# Copy number variation in the genomes of twelve natural isolates of *Caenorhabditis elegans*

**DOI:** 10.1186/1471-2164-11-62

**Published:** 2010-01-25

**Authors:** Jason S Maydan, Adam Lorch, Mark L Edgley, Stephane Flibotte, Donald G Moerman

**Affiliations:** 1 Department of Zoology, University of British Columbia, Vancouver, British Columbia V6T 1Z4, Canada; 2 Michael Smith Laboratories, University of British Columbia, Vancouver, British Columbia, V6T 1Z4, Canada; 3 Canada's Michael Smith Genome Sciences Centre, BC Cancer Agency, Vancouver, British Columbia V5Z 4S6, Canada

## Abstract

**Background:**

Copy number variation is an important component of genetic variation in higher eukaryotes. The extent of natural copy number variation in *C. elegans *is unknown outside of 2 highly divergent wild isolates and the canonical N2 Bristol strain.

**Results:**

We have used array comparative genomic hybridization (aCGH) to detect copy number variation in the genomes of 12 natural isolates of *Caenorhabditis elegans*. Deletions relative to the canonical N2 strain are more common in these isolates than duplications, and indels are enriched in multigene families on the autosome arms. Among the strains in our study, the Hawaiian and Madeiran strains (CB4856 and JU258) carry the largest number of deletions, followed by the Vancouver strain (KR314). Overall we detected 510 different deletions affecting 1136 genes, or over 5% of the genes in the canonical N2 genome. The indels we identified had a median length of 2.7 kb. Since many deletions are found in multiple isolates, deletion loci were used as markers to derive an unrooted tree to estimate genetic relatedness among the strains.

**Conclusion:**

Copy number variation is extensive in *C. elegans*, affecting over 5% of the genes in the genome. The deletions we have detected in natural isolates of *C. elegans *contribute significantly to the number of deletion alleles available to researchers. The relationships between strains are complex and different regions of the genome possess different genealogies due to recombination throughout the natural history of the species, which may not be apparent in studies utilizing smaller numbers of genetic markers.

## Background

Copy number variation is an important component of genetic diversity in both *Caenorhabditis elegans *[1] and humans [2-4] and has been associated with complex traits including autism spectrum disorder [5], mental retardation [6,7], and schizophrenia [8]. Genes involved in sensory perception, innate immunity and cell adhesion are overrepresented in copy number variants (CNVs) [1,2].

We have previously described extensive copy number variation relative to the Bristol N2 strain [9,10] in the genomes of two highly divergent isolates of *C. elegans*, CB4856 (Hawaii) and JU258 (Madeira) [1]. These observations were a by-product of our application of array comparative genomic hybridization (aCGH) to discover deletions in mutants of this nematode species. As our laboratory is part of the *C. elegans *Gene Knockout Consortium we are interested in methods that can help us identify gene deletions more efficiently. Our previous results for CB4856 and JU258 were encouraging, so we examined additional natural isolates to measure the extent of copy number variation in the genomes of less divergent strains. It is important to realize that the *C. elegans *Gene Knockout Consortium can deprioritize genes affected by deletions in natural isolates in our attempt to generate novel deletions in all genes. As many of the deletions we identify in this study are members of multigene families (see Results and Discussion) the Knockout Consortium can focus on obtaining deletions in members of these families that do not vary among wild type strains.

We also recognized that a large number of indel loci spread throughout the genome might allow us to more thoroughly characterize the relationships among strains, which have been difficult to ascertain due to the limited number of markers used in previous studies. Although *C. elegans *reproduces primarily through selfing of hermaphrodites, males allow rare outcrossing in the wild at an estimated rate of 1-2% [11-13]. The frequency of outcrossing varies among populations and has been estimated between 0.01% based on linkage disequilibrium measurements [11] to as high as 20% based on heterozygote frequencies [14]. Previous studies utilizing nuclear markers have identified some recombinant strains, but have been limited in their ability to precisely identify which regions of the genome have been exchanged due to the relatively small number of loci at hand [15,16]. More recently, SNP genotyping identified 41 haplotypes among 125 wild isolates [17], indicating that recombination has occurred more frequently than previously appreciated [15].

In this study we have used aCGH to detect copy number variation in 10 natural isolates of *C. elegans *in addition to the 2 isolates from our previous work [1]. We have chosen to use the term indel to refer to the deletions and duplications that we have detected, since many of them are < 1 kb in length and are thus not considered CNVs by some [3,18]. We use the term CNV only for larger aberrations. We use "deletion" to refer to a sequence absent in a natural isolate but present in N2, and "amplification" to refer to an increase in copy number relative to N2. An "amplification" detected in a natural isolate might alternatively represent the deletion of a duplicate copy in the N2 lineage. Only genes that are present in a single copy in the N2 genome are represented on our microarrays, therefore the presence of a gene (as in N2) cannot be reconstituted in an isolate by mutation or conversion from an ancestor carrying a deletion allele. Nevertheless, it is possible for a gene to reappear in a lineage as the result of outcrossing, recombining the intact gene into the lineage. Also, it is possible that extreme sequence divergence could be misinterpreted as a deletion in some cases (see Discussion).

We discovered 510 different deletions among 12 natural isolates, affecting over 5% of the genes in the canonical N2 genome. We found that recombination due to outcrossing in the natural history of the species has caused different portions of the genome to possess different genealogies, such that the relationships among strains are not well represented by a traditional bifurcating phylogenetic tree.

## Results

### aCGH reveals a bias favoring coding sequence deletions over coding sequence amplifications in *C. elegans*

We performed 10 new aCGH experiments utilizing our exon-centric whole genome microarray [1], which includes probes to 94% of the exons and 98% of the genes in the N2 reference genome. Each aCGH experiment compared a different natural isolate to N2. Table 1 summarizes the number and lengths of indels that were detected in each strain, including CB4856 and JU258 from our previous study [1], as well as the number of genes and pseudogenes that were deleted in each strain. It is important to note that nearly all of the indels detected in this study affect coding sequences. One "amplification" initially detected in all of the strains was subsequently identified by PCR and DNA sequencing as a 1788-bp deletion in our N2 strain (VC196), affecting exons 5 and 6 of *alh-2*, so that false amplification was ignored in all strains.

**Table 1:** Indels detected in twelve natural isolates of *C. elegans*.

Strain	Deletions	Amplifications	Median Indel Length	Mean Indel Length	Maximum Indel Length	Deleted Genes^a^	Deleted Pseudogenes^a^	Location
AB1	48	9	2481	7564	75120	147	26	Adelaide, Australia
CB3191	1	0	1262	1262	1262	1	0	Altadena, California, USA
CB4853	63	10	2460	8651	109400	237	16	Altadena, California, USA
CB4854	49	7	2240	6201	68860	122	20	Altadena, California, USA
CB4856	172	10	2885	7279	103200	517	91	Oahu, Hawaii, USA
CB4858	66	7	2481	7502	109400	211	16	Pasadena, California, USA
JU258	140	10	3271	12390	185700	671	117	Ribeiro Frio, Madeira, Portugal
JU263	68	6	2680	5080	68860	145	18	Le Blanc, France
JU322	66	12	2484	6270	70040	174	15	Merlet, France
KR314	124	6	2689	8674	118100	417	77	Vancouver, British Columbia, Canada
MY2	78	5	3485	10080	184100	301	44	Roxel, Munster, Germany
RW7000	8	2	2593	22600	116900	38	2	Bergerac, France
Overall	883^**b**^	84^**b**^	2700	8476	185700	1136^**c**^	216^**c**^	NA

^**a **^The number of deleted genes and pseudogenes includes those either wholly or partially deleted. ^**b **^The overall number of deletions and amplifications is the sum of those found in all strains, so indels found in more than one strain were counted multiple times. ^**c **^The overall number of deleted genes and pseudogenes is the number deleted in at least one of the 12 strains (genes deleted in more than one strain were counted only once).

We detected 883 deletions (510 distinguishable types) and 84 amplifications in the twelve natural isolates. Indel alleles were considered the same if they overlapped and both the leftmost and rightmost affected probes were within 3 probes of each other (see Methods - Strain relationships for details). Amplifications were less robustly detected than deletions due in part to the conservative criteria that we used (see Methods), but this alone does not account for the large preponderance of deletions in the strains. For example, relaxing the log_2 _fluorescence ratio of mutant:wild-type (log_2 _ratio) cutoff for amplifications from 1.0 to 0.9 or even 0.8 captured only a modest number of additional amplifications and did not affect the strong bias towards deletions in the strains (data not shown). The bias towards deletions is clearly evident in plots of log_2 _ratios on each chromosome (see Figure Six and Supplemental Figure One in [1]).

### Indel lengths

An ANOVA indicated that indel length varied significantly among the strains in our study (F = 1.8706, *P *= 0.0395), but not if RW7000 was excluded from the analysis (F = 1.4823, *P *= 0.1407). With just 8 deletions and 2 amplifications, the mean length of indels in RW7000 (22.6 kb) is greater than in the other strains, due largely to an 86-kb deletion on chromosome IV and a 117-kb duplication on chromosome V. To our knowledge, RW7000 is the only strain in our study with a high Tc1 transposon count [19], so transposon activity may have been more important in generating CNVs in this strain than in the others. The median indel length among all strains was 2.7 kb, with a mean of 8476 bp. Our ability to detect very small aberrations was restrained by the probe density on the arrays (The median distance between adjacent probes on the array is 289 bp, and we typically require at least 3 probes to be affected in order to call an indel. See Methods - aCGH). Indel length also varied significantly among chromosomes (F = 5.8772, *P *= 0.0000233), with larger indels on chromosomes V, IV and II. The mean size of indels on each chromosome ranged from 12.8 kb on chromosome V to just 1 kb on chromosome X. Furthermore, a Welch's two-sample *t*-test revealed a significant difference (*t *= 6.9256, *P *= 1.091 × 10^-11^) between the mean indel lengths on the autosome arms (as defined by Barnes et al. [20]) versus the autosome centers or the X chromosome (9505 bp and 3277 bp, respectively). This difference is not simply due to the small difference in the density of probes targeting the arms and the centers (see Methods).

### PCR validation of indel candidates

We performed PCR and gel electrophoresis to estimate the false positive and false negative rates of our aCGH indel predictions (Additional File 1: Table S1,). PCR primers were made to amplify 47 deletion loci and 7 amplification loci with predicted indel lengths between 250 - 2500 bp. This size range includes 40% of the indels that we predicted, and only 9% of predicted indels were smaller than 250 bp. Most indels detected in this study were larger than 2500 bp and are thus likely to have lower false positive and false negative rates than the loci that were tested. Among 96 deletion candidates tested at the 47 deletion loci, we observed 5 failed PCR reactions, 90 confirmed deletions and 1 apparent false positive (a 305-bp deletion candidate in JU258 affecting 4 probes), yielding a false positive rate of only 1% among the successful PCRs (Additional File 1: Table S1). For each deletion locus we also tested one isolate in which aCGH did not detect a deletion, and observed 13 failed PCR reactions, 31 confirmed negatives and 3 false negatives, giving a false positive rate of 9%, although it is possible that some of these false negatives affect non-coding sequences that are not probed by our microarrays and were thus undetectable by CGH. 6 of the 7 amplification candidates that we tested gave wild-type PCR fragments, suggesting that tandem copies of the amplified sequences are not found between the primer sequences that we used, perhaps because the additional copies exist elsewhere in the genome or because the amplifications extended beyond the primer sequences such that wild-type PCR products are still produced despite the amplifications. Interestingly, PCR indicated a deletion at a high-confidence amplification locus (8 probes affected with a mean log_2 _ratio of 1.3) in CB4854, suggesting that copies of the amplified sequence are found elsewhere in the genome and not between the PCR primer sites as in N2.

### Extensive copy number variation in the *C. elegans *genome allows even very closely related strains to be distinguished by aCGH

A single deletion was detected in CB3191 and subsequently confirmed by PCR and DNA sequencing. The deletion completely encompasses *math-15 *(sequenced deletion breakpoints are at chromosome coordinates II: 1866365 and II: 1868059). This illustrates the power of aCGH experiments to distinguish among strains, since CB3191 had previously appeared identical to N2 based on nearly complete mtDNA sequencing [15] and multilocus microsatellite genotyping [16]. Still, with only one deletion, CB3191 appears as similar to the canonical N2 genome as our laboratory N2 strain (VC196), at least in terms of the coding sequences targeted by our probes. This suggests the possibility that CB3191 is a laboratory contaminant or escapee (also suggested in [17]). In effect, the aCGH experiment comparing CB3191 to N2 served as a self-versus-self negative control and demonstrated that our whole genome array design, in itself, does not produce false positives.

CB4856 and JU258 were the most divergent strains relative to N2, with more indels and more genes deleted than any of the other strains. KR314 was the most divergent among the other strains and had more deletions on chromosome II than any other strain, including CB4856 and JU258, but curiously had no deletions at all on the left half of chromosome V (V-L) where 18 deletions were present in CB4856 and 23 in JU258. The absence of deletions on V-L suggests the possibility that KR314 reacquired an N2-like V-L through recombination. Interestingly, RW7000 was the least divergent strain other than the N2-like CB3191. This result contrasts with an earlier study stating that RW7000 appeared to be more divergent than 6 of our strains based on the number of alleles in 31 chemoreceptor genes [21].

### The distribution of indels in the genome and the overrepresentation of indels in particular gene families

**Figure 1:**
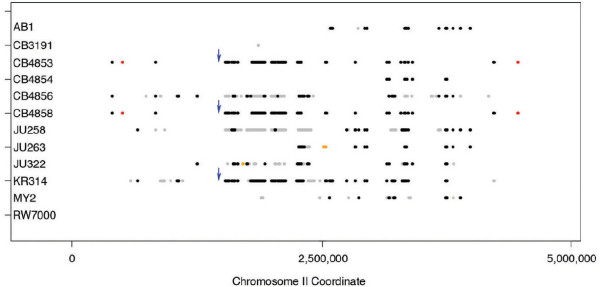
**Indels on the left arm of chromosome II in twelve natural isolates of *C. elegans***. Deletions unique to a strain are plotted in grey and deletions found in multiple strains are plotted in black. Amplified sequences present in only one strain are shown in orange and those found in multiple strains are shown in red. The actual position of amplified sequences in the genome is unknown. The position of amplifications shown here corresponds to the position of the single copy of that sequence in the N2 reference genome. Small indels are not shown to scale. The blue arrows indicate the site of a possible recombination event. KR314 shares alleles with CB4853 and CB4858 to the right of the arrows but not to the left.

Figure 1 plots all of the indels we detected in each strain on II-L. Additional File 2: Figure S1 plots a higher resolution figure of this type for the entire genome. Chi-square tests indicated that both the number of deletions and the number of amplifications varied significantly among chromosomes (*P *< 2.2 × 10^-16 ^and *P *= 1.281 × 10^-5^, respectively), after adjusting for the different number of probes targeting each chromosome. As previously described for CB4856 and JU258 [1], indels in *C. elegans *are strikingly more common on autosome arms than in the autosome centers or on the X chromosome. The autosome arms span just 38% of the probes on our Whole Genome microarray but include 83% of the indels that we identified (85% of the deletions and 64% of the amplifications).

Additional File 3: Table S2 lists all of the genes affected by the indels we detected. 1136 different genes were either wholly or partially deleted in at least one strain. The same gene families that we found to be overrepresented in indels in CB4856 and JU258 are also clearly overrepresented amongst indels in the new strains in this study, most notably the MATH-BTB, F-box, lectin and serpentine chemoreceptor gene families. All of these gene families cluster on the autosome arms [22].

### Relatedness inferences based on deletions shared by multiple natural isolates

**Figure 2:**
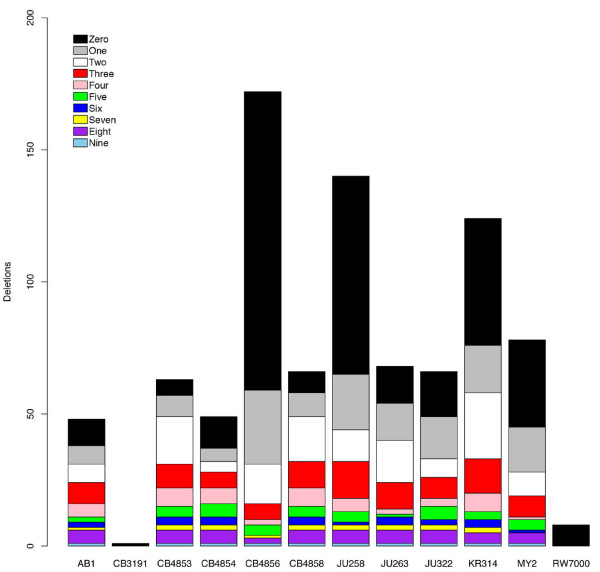
**The number of deletions in each of 12 natural isolates of *C. elegans *that are also present in other isolates**. The numbers of other isolates that carry the same deletions are indicated by the colors in the figure legend.

Many of the indels we detected were found in multiple strains (see Methods - Indel identification). Figure 2 shows the number of deletions found in each strain, and the number of other strains that carry the same deletions. The strains with the most deletions also have the highest proportion of unique deletions in our data set, excluding CB3191 and RW7000, which carry only unique deletions. By this measure, CB4856 was the most divergent strain, with 66% (113/172) of its deletions being unique among the 12 strains. This agrees closely with a study reporting that 70% of CB4856 SNPs were not present in nine other natural isolates [23]. Unique deletions comprise 64% (75/140) of the deletions in JU258, 42% (33/78) of the deletions in MY2, and 39% (48/124) of the deletions in KR314. In addition to carrying the most indels relative to N2 among the strains in our study, CB4856, JU258, MY2 and KR314 are also the least N2-like according to SNP genotypes [17].

**Figure 3:**
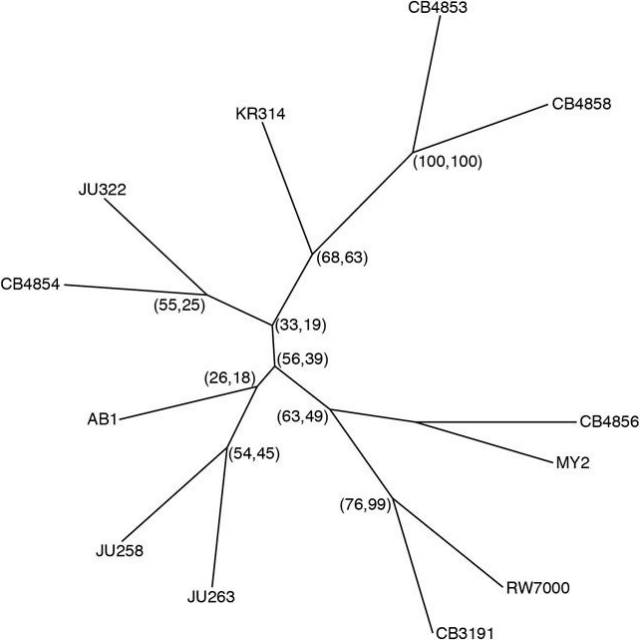
**Unrooted consensus tree for 12 natural isolates of *C. elegans***. The two numbers listed in parentheses next to each node are the percentage of trees among 1000 bootstrap replicates that included all strains distal from CB4856, under Camin-Sokal and Wagner parsimony, respectively. The tree should not be interpreted strictly as a phylogeny due to recombination between strains.

Table 2 displays the number of deletions shared by all pairs of strains. Deletions were used as markers to infer a strain phylogeny under both Camin-Sokal parsimony [24], which assumes that deletions are derived states and transitions to the presence of a deletion are more likely than transitions to the absence of a deletion, and Wagner parsimony [25,26], which considers the appearance or disappearance of deletions equally likely and does not presume ancestral states. Both parsimony methods gave the same consensus tree (Figure 3) based on 1000 bootstrap replicates drawn with replacement from the 510 deletion loci that we identified. We chose to exclude amplifications from this analysis because they were less robustly detected than deletions. Deletions were treated as independent characters despite the presence of linkage between loci. We detected significant multilocus linkage disequilibrium (standardized index of association (*I*_A_^S^) = 0.072, *P *< 0.001, see [27]) consistent with other studies [11,13,28]. It is important to note that because deletions are more common on the autosome arms, these regions of the genome factored heavily into the relationships that we inferred. Overall, close relationships were inferred between CB4853 and CB4858, and between CB3191 and RW7000 (which closely resembled N2). CB3191 and RW7000 were both very N2-like and shared no indels with any other strains in our study.

**Table 2:** Number of deletions shared by all strain pairs.

Strain	AB1	CB3191	CB4853	CB4854	CB4856	CB4858	JU258	JU263	JU322	KR314	MY2	RW7000
AB1	48^**a**^	0	14	20	12	13	20	18	12	21	13	0
CB3191	0	1^**a**^	0	0	0	0	0	0	0	0	0	0
CB4853	14	0	63^**a**^	24	12	52	16	13	23	34	12	0
CB4854	20	0	24	49^**a**^	13	21	15	15	23	19	11	0
CB4856	12	0	12	13	172^**a**^	13	17	17	18	18	16	0
CB4858	13	0	52	21	13	66^**a**^	18	17	23	34	11	0
JU258	20	0	16	15	17	18	140^**a**^	31	17	36	26	0
JU263	18	0	13	15	17	17	31	68^**a**^	18	26	15	0
JU322	12	0	23	23	18	23	17	18	66^**a**^	20	12	0
KR314	21	0	34	19	18	34	36	26	20	124^**a**^	14	0
MY2	13	0	12	11	16	11	26	15	12	14	78^**a**^	0
RW7000	0	0	0	0	0	0	0	0	0	0	0	8^**a**^

^**a **^The number listed for all comparisons between a strain and itself is simply the total number of deletions detected in that strain.

Different relationships are predicted by different regions of the genome due to the presence of recombination in the lineage, and this is reflected in the low bootstrap confidence at many of the nodes on the consensus tree. An example of a possible recombination event is indicated in Figure 1. AB1 was previously identified as a recombinant strain based on discrepancies between the phylogenies inferred by mitochondrial and nuclear marker data sets [15], and the bootstrap support at the node leading to the AB1 on our consensus tree is particularly low. While JU258 was most similar to JU263 on I-R, it shared more deletions with KR314 on II-L, III-L and V-R. JU258 and KR314 were identified as each other's closest relative on 27% of CS (32% W) trees inferred from all bootstrap replicates, and the group of JU258/KR314/JU263 was also fairly common (19% CS, 28% W). KR314 was more similar to CB4853 and CB4858 on chromosome II. Other groups with appreciable bootstrap support but not appearing on the consensus tree included AB1/CB4854 (32% CS, 20% W), CB4853/CB4858/CB4854/JU322 (21% CS, 29% W), MY2/JU258 (18% CS, 22% W) and MY2/JU258/JU263 (12% CS, 23% W).

## Discussion

### A bias favoring deletions affecting gene families involved in environmental responses and innate immunity

Genes thought to be involved in sensory perception and innate immunity are enriched in indels in both *C. elegans *[1] and humans [29]. These gene families include the *C. elegans *F-box and serpentine receptor chemoreceptor families, as well as genes involved in ubiquitination and secreted proteins possibly involved in innate immunity [22]. Chemoreceptor gene families have undergone significant expansion in *C. elegans *since its common ancestor with *Caenorhabditis briggsae *[30], including multiple rounds of tandem duplication in the *sra *and *srab *families [31], and appear prone to gene gains and losses. We found that indel lengths were larger on the autosome arms where homologous gene clusters of these same gene families are the most common [22]. Higher recombination rates [20] and the presence of homologous gene clusters probably predisposes autosome arms to non-allelic homologous recombination (NAHR) events, which tend to generate larger CNVs, whereas smaller indels are more likely created by non-homology based mechanisms [3,32]. Conversely, the increased frequency of SNPs in the autosome arms of natural isolates is probably not due to the increased rate of recombination, but rather to increased genetic hitchhiking and/or background selection in the autosome centers [33].

We chose to include pseudogenes in Additional File 3: Table S2 because many genes annotated as pseudogenes in the N2 strain probably have functional copies in other natural isolates, especially genes with a single defect in N2 (usually a premature stop codon or deletion [21]). The large number of deleted genes we detected suggests that N2 itself carries deletion alleles for many genes that are present in other natural isolates. These events remain undetected since only probes to N2 sequences are included on our microarrays.

A bias favoring deletions over insertions has previously been observed in patterns of pseudogene variation in *C. elegans *[34]. It has also been suggested that there may be a high rate of spontaneous deletion in the *C. elegans *genome [35], and perhaps even selection for small genome size [36]. We detected far more coding sequence deletions than amplifications in all of the natural isolates. This is the opposite of what is found in *Drosophila melanogaster *[37] and humans [3], and may at first seem puzzling since selection should tend to favor coding sequence amplifications over deletions. Non-allelic homologous recombination (NAHR) is one mechanism for creating copy number variation and it generates a mutational bias favoring deletions [38]. It is possible that the *C. elegans *mating system, consisting mainly of selfing, coupled with recurring population bottlenecks due to a demography including frequent extinction and recolonization events [13] producing founder effects, reduces the effective population size (*N*_e_) of *C. elegans *to a point where selection against deletions cannot overcome stochastic events and the force of genetic drift, which in turn leads to the fixation of more deletions than amplifications as a result of the mutational bias of NAHR. The worldwide *N*_e _of *C. elegans *has been estimated between 200 and 44,000 [39], which is orders of magnitude less than the *N*_e _of Drosophila [40] and possibly also less than the current human *N*_e _([41]) and the historic human *N*_e _of not more than perhaps 10,000-18,000 [41-43]. It is also possible that loss of gene function could be adaptive in some cases [44], such as for certain chemoreceptors in specific environments.

### New insights into complex strain relationships resulting from recombination and outcrossing in the *C. elegans *lineage

Different portions of the genome will possess different genealogies due to recombination. Trees are therefore inherently flawed in their generalized depiction of strain relationships when recombination has occurred between lineages, and should not be interpreted as phylogenies. Nevertheless, our results largely agree with other trees inferred from nuclear markers [15,16]. Strains that are close together on these trees generally share more deletions than do strains that are further apart, with a few bu but notable exceptions probably resulting from the limited number of loci available in earlier studies. For example, our results differ from the Haber *et al*. study [16] in which CB4858 and CB4854 appeared most closely related to one another, identical at 10/10 microsatellite loci. We found that CB4858 was much more closely related to CB4853, sharing 52/66 of its deletions with CB4853 (52/63 CB4853 deletions were found in CB4858) but sharing just 21/66 deletions with CB4854. CB4858 and CB4853 appeared identical on II-L and throughout chromosomes III and V (one small two-probe CB4858 deletion candidate on chromosome III did not quite meet our *P*-value cutoff in CB4853, but both probes showed log_2 _ratios < -2). Only 5 CB4853 deletions not found in CB4858 were present in any other strains, including 2 deletions on the X chromosome that were found in MY2, and 3 deletions on II-R that were present in CB4854. Four of the 8 deletions found in CB4858, but not in CB4853, were present in JU263 (on chromosomes I, II and X). CB4858 and CB4854 did share deletions in the regions of some of the Haber *et al*. microsatellites but not in all cases. For instance, CB4858 shared a deletion with CB4853 and CB4856, that is not found in CB4854, which is just 6 kb away from the microsatellite allele shared by CB4858 and CB4854 on II-L. This highlights the importance of using a large number of loci spread throughout the genome when estimating strain relatedness.

Another discrepancy between the relationships inferred in our study and those estimated by Haber *et al*. [16] involves JU263. Microsatellites did not reveal close relatedness between JU263 and JU258, but JU263 shared more deletions with JU258 (31/68) than with any other strain in our study. Results of SNP studies also suggest a closer relationship between these two strains [17]. JU263 was most similar to KR314 and CB4854 on V-R, and shared 26/68 deletions with KR314 overall. Although it does not appear on the consensus tree, a JU263/JU258/KR314 group appeared on 28% of W trees inferred from all bootstrap replicates. Chromosome III told yet another story, where JU263 shared 4/4 deletions on III-L with JU322, and none of these with any other strain.

Overall, CB4856 did not appear particularly closely related to any of the other strains. CB4856 shared the most deletions with KR314, JU322, JU258, JU263 and MY2 (18, 18, 17, 17, and 16, respectively), and slightly fewer with the remaining strains (but none with CB3191 and RW7000). However, specific regions of the CB4856 genome more closely resembled particular strains. For example, CB4856 and JU258 shared 5 deletions in common over a 10.6 Mb interval on chromosome V, but each shared only one deletion over the same interval with any other strain (MY2). CB4856, JU258 (and MY2 in the region of the deletion shared by all 3 strains) also share many CB4856 SNP alleles in this portion of the genome [17]. Still, CB4856 and JU258 were significantly diverged from one another in this region, which included 18 deletions unique to CB4856 and 10 deletions unique to JU258. Many other regions of similarity among different groups of strains are evident in Additional File 2: Figure S1 and Additional File 3: Table S2, illustrating that the relationships among strains are complicated due to recombination and outcrossing, probably to a greater extent than previously appreciated in studies utilizing fewer genetic markers [15,16]. Our study sheds further light on the extent of recombination in the natural history of the species because unlike the study by Rockman et al. [17], our study includes a great number of alleles not found in CB4856 (see Figure 2).

### Very common indels, mutation hotspots, and the possibility of extreme sequence divergence masquerading as deletions

Remarkably, we found 6.7-kb deletions (in CB4854, CB4856 and JU322) and duplications (in KR314 and MY2) that affected exactly the same 117 probes on chromosome III. These indels target the retrotransposon *retr-1*. The log_2 _ratios for these amplifications (particularly for MY2) indicated a roughly four-fold amplification, suggesting the presence of multiple copies of the retrotransposon.

Some deletions and amplifications were more common among the strains in our study than the allele observed in N2 (see Figures 1 and 2). For example, a 10-kb deletion on chromosome III was found in all strains except CB3191 and RW7000. This deletion affected 3 uncharacterized genes, Y75B8A.31, Y75B8A.32 and Y75B8A.34, and could possibly be of ancient origin. It is possible that some common indels have arisen by independent mutations, particularly if there are hotspots susceptible to mutation by NAHR [32] and/or subject to positive selection. However another study has shown that all of the strains in our study except CB3191 and RW7000 also share Hawaiian SNP alleles in precisely this region of chromosome III (see Supplemental Table Two in [17]), suggesting that the common deletion we detected is shared by descent. We also found several very common deletions on V-R (see Additional File 2: Figure S1 and Additional File 3: Table S2), which is a region rich in copy number variation.

Regions with very high sequence divergence could potentially produce log_2 _ratios negative enough to appear as deletions in aCGH data, but are unlikely to account for a sizeable proportion of the deletions that we detected. The false positive rate in our PCR validation of small deletion candidates was only 1%. On average, roughly 10% or more of the nucleotides in each of several adjacent 50-mer probes would need to be mutated in order to approach our log_2 _ratio cutoff for deletions [45]. This level of sequence variability in our probe sequences is particularly unlikely because our probes target coding sequences. On average, single nucleotide polymorphisms relative to N2 exist at 1/840 nucleotides in CB4856 [46,47] and 1/1500 nucleotides in CB4858 [48], but recent whole genome sequencing of the CB4856 genome has identified several regions of much higher sequence diversity (> 10×) that sometimes coincide with deletions identified in our aCGH data (David Spencer and Ryan Morin, personal communication). Nevertheless, one CB4856 deletion that we detected and an overlapping region of high sequence diversity have both been independently identified and confirmed, and are associated with the genetic incompatibility between CB4856 and N2 [49]. Reproductive isolation has allowed for the accumulation of sequence diversity in this region. Still, we cannot rule out that some of the deletions we report could be false positives resulting from regions of extreme sequence divergence. This is probably more likely in the most divergent strains. Genes in some of the gene families that are overrepresented amongst the deletions we report are known to be subject to positive selection for changes in amino acid sequence [22,50].

## Conclusion

We have shown that there is substantial copy number variation in coding sequences in the *C. elegans *genome. Indels are most common on the autosome arms, especially on chromosomes II and V. Deletions relative to N2 are much more common than amplifications. Over 5% of the annotated genes in the N2 genome overlap with indels in at least one of the 12 strains that we examined. This underestimates the copy number variation in the *C. elegans *genome because we examined only 12 natural isolates, our ability to detect very small indels is limited by the probe density on our microarrays, and we did not attempt to detect indels that do not affect exons. Approximately 26% of the *C. elegans *genome is intronic and 47% is intergenic sequence [10]. The indels that were detected should be useful in explaining natural phenotypic variation, particularly in chemosensation [51] and innate immunity [52]. aCGH is a powerful method to quickly obtain a large number of genetic markers throughout the genome, and has revealed complex relationships among wild *C. elegans *isolates resulting from recombination and outcrossing events throughout the natural history of the species. The naturally occurring deletions we have detected contribute significantly to the number of deletion alleles available to the *C. elegans *research community, and genes affected by these deletions can be deprioritized in efforts to generate novel mutations.

## Methods

### Strain selection, nematode culturing and DNA preparation

The N2 reference strain in all experiments was VC196, a subculture of N2 received from the Caenorhabditis Genetics Center (CGC) in 2002. RW7000 was acquired from the lab of Robert Waterston in 1987 and was submitted by that lab to the CGC in 1991. All other strains were received directly from the CGC and grown for a minimal number of generations prior to DNA preparation. We selected the strains in an attempt to sample a range of the microsatellite diversity observed by Haber et al. [16], but also included strains thought to be very closely related to each other in an attempt to better distinguish them with a larger number of loci. We also included JU322, which was not part of the Haber et al. study. We intentionally selected some strains suspected to be recombinant, including AB1 [15] and CB4854 [16], to test our ability to identify particular regions of the genome that have been exchanged as the result of outcrossing. Nematodes were grown as previously described [9] on 150-mm NGM agar plates seeded with *Escherichia coli *strain χ 1666. Nematode populations were grown to starvation, harvested by washing with M9 containing 0.01% Triton X-100, and washed an additional 7 times by centrifugation, removal of the supernatant by aspiration, resuspension and vortexing in M9/Triton-X100. After the final wash, DNA was prepared by standard phenol-chloroform extraction and ethanol precipitation as previously described [1].

### aCGH

Probes on our whole genome microarray were initially selected from build WS139 of the *C. elegans *genome [1]. The median distance between adjacent probes included on the microarray is 64 bp (66 bp in the arms and 63 bp in the centers), while the mean probe spacing is 260 bp (297 bp in the arms and 233 bp in the centers). Microarray manufacture, DNA fragmentation and labeling, sample hybridization and imaging, and fluorescence intensity measurement were performed by Roche NimbleGen, Inc. as previously described [1]. Log_2 _ratios (Natural Isolate/N2) were calculated and then normalized using the robust LOWESS regression [53] implemented in the R programming language [54] with a smooth spanner setting of f = 0.4. The microarray data have been deposited in NCBI's Gene Expression Omnibus [55] and are accessible through GEO Series accession number GSE19440 http://www.ncbi.nlm.nih.gov/geo/query/acc.cgi?acc=GSE19440.

### Indel identification

Indels were detected with a segmentation algorithm developed by S. Flibotte [1], briefly described here. The algorithm was written in the C programming language and employs a highly efficient bottom-up approach. The program assumes that log_2 _ratios for all probes are drawn from a normal distribution and begins by considering each probe as an individual segment. The algorithm performs *t*-tests for all possible mergers of adjacent segments to estimate the probability that the log_2 _ratios were drawn from samples with the same mean. These *P*-values are stored in a heap, prioritizing all possible mergers. The data structure is an ordered doubly linked list. The program makes the most likely merger of adjacent segments according to these *P*-values, calculates the new mean log_2 _ratio of the segment resulting from the merger, calculates *P*-values for subsequent mergers of the new segment with its neighbours and updates the heap. This procedure is repeated until the *P*-value of the next most likely merger is less than a critical value supplied by the user (typically 0.05). The program calculates a *P*-value for each remaining segment using a one-sample *t*-test. Segments are then labeled as candidate "amplifications" or "deletions" if their both their mean log_2 _ratios and *P*-values meet or exceed cutoff values supplied by the user. Segments that do not meet both of these criteria are labeled as "normal". If desired, the program can then merge all adjacent segments sharing the same label, recalculating the mean log_2 _ratios and *P*-values for the merged segments. The entire process takes just a few seconds for data sets of 380,000 probes like those used in this study.

Aberrant segments with a *P*-value ≤ 0.01 were called deletions if the mean log_2 _ratio of probes in the segment was ≤ -2, and called amplifications with a mean log_2 _ratio ≥ 1. Most indels that we classified as deletions are probably truly deletions as opposed to insertions in the N2 lineage, since we used probes targeting coding sequences that contain no non-unique 20-mers and no more than 70% homology to other genome sequences. A novel N2 gene arising by duplication and subsequent sequence divergence in the N2 lineage would have to accumulate many coding sequence mutations in order to pass our probe selection filters. N2 genes arising from recent duplications are probably among the 2% of genes not represented on our microarrays. Nonetheless, we cannot completely rule out the possibility that some indels affecting novel N2 genes could have been misclassified as deletions in the natural isolates.

*P*-values were not corrected for multiple tests, but a Bonferroni correction would not exclude many indels since a large majority of them have *P*-values far below the cutoff (91% of all indels have *P*-values < 0.001, and 83% have *P*-values < 0.0001). All indels affected 3 or more consecutive probes, with the exception of 10 deletions (~1% of the indels) that were detected by just 2 probes with very negative log_2 _ratios.

All aberrant segments were examined manually and some adjustments were made to fine-tune the selection of the leftmost and rightmost probes ("breakpoint probes") within the indels. Some segments were interrupted stretches of probes that did not give log_2 _ratios consistent with an indel and were manually split into multiple segments. After these adjustments, the mean log_2 _ratios and *P*-values were recalculated for all segments with one-sample *t*-tests using R to ensure that our cutoffs were still met. In most cases these adjustments further decreased the *P*-value of the indels.

Occasionally, probes flanking indels show unusual log_2 _ratios outside the normal range for unaffected probes [1]. This can sometimes make identification of the indel breakpoints less certain. For each indel, we identified "flanking probes" beyond the left and right breakpoint probes to demark the point at which more normal log_2 _ratios begin to consistently appear again (log_2 _ratios > -0.8 for deletions, and log_2 _ratios < 0.5 for amplifications). 78% of these flanking probes were adjacent to their corresponding breakpoint probes, and 90% were within three probes of indels.

### Validation of indel candidates by PCR and gel electrophoresis

Indel loci with minimum putative lengths of 250 - 2500 bp were chosen for validation to give PCR amplicons with convenient product sizes. PCR primers were designed to include sequences that aCGH suggested were not included in the indels, but it is possible that some indels may have extended into primer sequences (leading to failed PCR reactions). Loci on all six chromosomes were tested. For each indel locus we tested one or more natural isolates with putative indels within the PCR amplicon, one isolate with no apparent indel, and N2 DNA as a control. All reactions were run with annealing temperatures of 55°C and a 2-minute extension time, with no attempt to optimize PCR conditions. False negatives were considered to be any PCR product found in an isolate with no predicted indel that differed from the N2 band size, whether or not the band size was the same as that found in isolates with predicted indels.

### Chi-square tests, *t*-tests and Analysis of Variance (ANOVA)

All remaining chi-square tests, *t*-tests and ANOVA tests were done using R. For the chi-square tests, the expected number of indels on each chromosome was calculated based on the proportion of probes targeting that chromosome, which essentially corrects for differences in length and gene content among chromosomes. Indel lengths were measured from the middle of the left breakpoint probe to the middle of the right breakpoint probe. Indels found in more than one strain were counted multiple times.

### Affected genes

Probe coordinates were obtained by remapping probe sequences to the most recent genome data freeze (WS190) using MegaBLAST, which utilizes a greedy algorithm [56] to align DNA sequences. 24 probes on the array no longer had perfect sequence matches in the genome due to changes in the genome sequence from WS139 to WS190, but none of these probes were present in any of the indels that we detected.

In order to generate the list of genes affected by indels in Additional File 3: Table S2, we first extracted the start and stop coordinates for all genes in genome build WS190 from WormBase. From that list, we extracted only the genes that overlapped the coordinates spanned by the indel breakpoint probes. Genes completely contained within the region spanned by an indel were listed as entirely affected. Discrepancies between Additional File 3: Table S2 and the list of deleted genes in CB4856 and JU258 given in Maydan *et al*. (2007) are due to the adjustments we made to the indel breakpoints and because the genes listed in the previous study were extracted from an older genome build (WS150).

### Strain relationships

All deletion loci were treated as discrete presence-absence characters. Strains were considered to carry the same deletion allele if their respective deletions overlapped and both their left and right breakpoint probes were within 3 probes of each other, or in a small number of cases (where the breakpoint and flanking probes were more ambiguous) if the breakpoint probes in one strain fell within the region spanned by the flanking probes in another strain. Remarkably, most deletions found in multiple strains according to these criteria had exactly the same breakpoint probes, illustrating the reliability of the log_2 _ratios. The single case where we identified deletions and amplifications affecting the same probes could have been treated as a multi-state locus, but this was not done because we chose to exclude amplifications from this analysis.

Unrooted trees were inferred from the 510 deletion loci using Phylip 3.66 [57]. The most parsimonious trees were inferred under both Camin-Sokal and Wagner parsimony methods from 1000 bootstrap replicates drawn with replacement from the loci. An unrooted consensus tree was then inferred separately for each method. The true position of the root of the consensus tree is unknown.

### Linkage disequilibrium

We used LIAN 3.5 [27] to calculate a standardized index of association (*I*_A_^S^) based on the original formulae given by [58,59]. *I*_A_^S ^is 0 at linkage equilibrium. The program tested significance with a Monte Carlo simulation, resampling loci without replacement over 1000 iterations, in order to scramble their order and generate a null distribution of *I*_A_^S^. The *P*-value is the probability, under the null hypothesis of linkage equilibrium, of *I*_A_^S ^being greater than or equal to the value observed for our data set.

## Authors' contributions

JSM, MLE and DGM designed the experiments. JSM performed all experiments and data analyses, prepared the figures and tables and wrote the manuscript. AL assisted in preparing Table S2. MLE provided advice and helped to culture some of the nematodes. SF provided advice and designed the microarray and segmentation algorithm. DGM provided advice and interpretation of data and helped to write the manuscript. All authors have read and approved the final manuscript.

## Supplementary Material

Additional file 1 **Table S1**. Indel validations by PCR and gel electrophoresis. Click here for file

Additional file 2 **Figure S1**. Indels in 12 natural isolates of *C. elegans*. Click here for file

Additional file 3 **Table S2**. Genes affected by copy number variants in *C. elegans*. Click here for file
