# Supplementary material for: Copy number variation in the genomes of twelve natural isolates of Caenorhabditis elegans
Source: BMC Genomics. 2010 Jan 25;11:62. doi: 10.1186/1471-2164-11-62 (PMC2822765; doi:10.1186/1471-2164-11-62)
Supplement: Additional file 2 — Figure S1. Indels in 12 natural isolates of C. elegans. [file 1471-2164-11-62-S2.PDF]

Locus	Indel	Chromosome	Left Primer	LP coord	Right Primer	RP coord	WT Amplicon	Left Breakpoint	Right Breakpoint	Indel Length	Indel Strains Tested	False Positives	Negative Control	False Negatives	Failed PCR
1	D	I	gttcccagcaattccaaaaa	2482188	ccagaaatttttgcctcgaa	2485078	2891	2483194	2483482	289	JU258	None	CB4853	None	None
2	D	I	gcatgcaggagttgtttcaa	11147420	ccattgccattaccatttcc	11149155	1736	11147948	11148200	253	JU258, JU263, KR314	None	CB4854	None	None
3	D	I	tttattttcagacccgccac	11233095	tactgtcggtgccaatttca	11235554	2460	11234306	11235067	762	JU258, JU263, KR314	None	CB4856	None	CB4856
4	D	I	ctacccagtgagcttgcctc	11417973	gagttcactaggccaaagcg	11421234	3262	11418354	11419944	1591	CB4858, JU258, JU263	None	JU322	None	None
5	D	I	cacgaaactcgcaaattgaa	11634619	tgaaatggaatcttcgggag	11636155	1537	11635184	11635671	488	JU58, JU263	None	CB4858	None	None
6	D	I	atggaaatgaggacgtggaa	11744008	cacaacgctggatacattgc	11747247	3240	11744796	11745411	616	CB4858	None	JU258	None	CB4858, JU258
7	D	I	cattttcccgcaacttctgt	12072566	tttcgaatcaaggaagacgg	12074821	2256	12073111	12074693	1583	JU258, JU263, KR314	None	CB4853	None	None
8	D	I	agtgagcaaggcgagaatgt	12106505	ttgattgaacgctgtgaagg	12108428	1924	12107118	12108359	1242	CB4856	None	KR314	None	None
9	D	I	acccctggagtacacgacac	12758206	agagctccgccattacagaa	12759426	1221	12758660	12759217	558	CB4856, JU263, KR314	None	CB4854	None	None
10	D	I	gctcttgctaaggaacgtgg	13251366	cattgattgcacggatgaag	13253310	1945	13251795	13252249	455	CB4854, CB4856	None	CB4858	None	None
11	D	II	cacgcctaccgtagtttgct	2028982	cagaattgcatccgttcctt	2032597	3616	2030102	2031974	1873	CB4856, JU322	None	JU258	None	CB4856, JU258
12	D	II	caaaacacacttcccggact	2933226	aacttggatggttggtgagc	2935255	2030	2934689	2935105	417	JU258, JU263, KR314	None	CB4856	None	None
13	D	II	gagaaccgtgcaattttggt	2954725	ccgctgcgagtagagatacc	2956363	1639	2955107	2956099	993	JU258, JU263, KR314	None	JU322	None	None
14	D	II	tgcccattatccatgtcaga	3155643	gagatgtagaacccggcaaa	3158920	3278	3156114	3158199	2086	CB4853, CB4854, CB4858, JU322, KR314	None	CB4856	CB4856	None
15	D	II	ctcgggttggattctttgaa	3330946	gaaagttggatgattgggga	3333733	2788	3331375	3331990	616	CB4853, CB4858, JU322	None	KR314	KR314	None
16	D	II	ataggacattccgttgacgc	3406075	attcctccaactccctcgat	3408114	2040	3406578	3407569	992	CB4856	None	CB4853	None	None
17	D	II	tattgctgcaagttcatcgc	3824544	tctttcccgaatcagtggtc	3827364	2821	3825717	3826934	1218	JU322	None	CB4858	None	CB4858
18	D	II	ccatcgctgaagcacatcta	4228735	ccacggaaactaaccgaaaa	4230883	2149	4229046	4230619	1574	CB4853, CB4858	None	JU258	None	None
19	A	II	cccaatattttcacgggatg	6894770	gtctggatttggcattcgtt	6896239	1470	6895428	6895785	358	CB4854	NA	JU263	NA	None
20	D	II	ttgctcagcttttggcctat	10311762	cagtttgtggctgtctggaa	10313337	1576	10312319	10312904	586	KR314	None	JU322	None	None
21	D	II	gaaattccaggtcggacaaa	10467144	gcggaagttacggttaccaa	10468897	1754	10468472	10468797	326	CB4853, CB4854, JU322	None	KR314	None	None
22	D	II	agggtctcaaccgacatcag	10643136	accggcaatgttttctcaag	10644819	1684	10644116	10644561	446	CB4856, CB4858, JU263	None	CB4853	None	None
23	A	II	attgctcatcaaaaccggag	11386922	atcggatgagatttggttcg	11388193	1272	11387272	11387837	566	CB4853, JU322	NA	CB4854	NA	None
24	D	II	ggtgtccaaaactgcaaggt	11820040	gggcatcttcaaagaaccaa	11822089	2050	11820849	11821128	280	CB4858, JU263	None	CB4856	None	None
25	D	II	ttaaacgccattgtatgcca	12476205	attttatgcgagacgttggg	12477816	1612	12476605	12477344	740	CB4856	None	CB4858	None	None
26	A	II	ttttgagcagttttgttgcg	13155203	atttcgagtgtgaaaagccg	13156584	1382	13155506	13156513	1008	CB4854	NA	JU263	NA	None
27	A	II	aaagttttccagccgaatga	15084362	gtgtgatggaaggtgtcgtg	15085902	1541	15084647	15085167	521	CB4854, CB4856, CB4858, JU258, JU322, KR314	NA	CB4853	NA	None
28	A	III	acattcgcatcgtacaacca	115292	atcatcagtctgtggagccc	116497	1206	115810	116122	313	CB4853, CB4854, CB4856, CB4858, JU263, JU322, KR314	NA	JU258	NA	None
29	D	III	cagttggcagaagacgatca	1883889	tggggacgagtgtcaataca	1885239	1351	1884481	1885032	552	CB4853, CB4858, JU258, KR314	None	CB4854	None	None
30	D	III	atttcagtggcgtccagttt	2743919	aatacctacgtgcctgtgcc	2745629	1711	2744447	2745032	586	CB4853, CB4856, CB4858	None	JU258	None	None
31	D	III	ttgccaaggtttcaagctct	4399043	atcgatccgttccttctcct	4400046	1004	4399209	4399607	399	CB4853, CB4858	None	KR314	None	None
32	A	III	agcttcagccatgtcgattt	5343418	caaccggatgattatttggg	5345677	2260	5343902	5344232	331	CB4853, CB4854, CB4856, CB4858, JU258, KR314	NA	JU263	NA	None
33	D	IV	tcttgcaattgctatgccac	1246152	agctacccgtttttgggagt	1248062	1911	1246553	1247435	883	JU258	None	CB4853	None	None
34	D	IV	tgtgatattcggtgagccaa	1247391	agaaattcccgtctgtggtg	1250330	2940	1248520	1250055	1536	JU258	None	CB4858	None	CB4858
35	D	IV	gtcgtgtgattccggaaagt	2553480	taccaccaccaaagcaacaa	2556247	2768	2553855	2555752	1898	JU322	None	KR314	None	KR314
36	D	IV	cctccacctacaccaccatc	2593446	tggcgtttgtgtagtcttcg	2595670	2225	2593952	2594678	727	CB4856, JU322	None	CB4853	None	CB4853, JU322
37	D	IV	acagcctcgaaacaatttgg	4970461	actgcacatttttggccatt	4972921	2461	4971811	4972592	782	JU263	None	CB4854	None	None
38	D	IV	cgtgaagctgtcaaaaacca	14547050	cgttttctcaagaactgggg	14549889	2840	14547780	14549271	1492	KR314	None	CB4856	CB4856	None
39	D	IV	ttcaaacgtaaaccccttgc	16293866	cctttttcgtcttttgcagc	16296524	2659	16294716	16296254	1539	KR314	None	CB4858	None	CB4858
40	D	V	tgttggtctggaatgttgga	2153882	aaaatgcggataaacgcaac	2156164	2283	2154181	2155113	933	CB4856	None	JU258	None	CB4856, JU258
41	D	V	cttgaccttgtcgcaaatga	3996774	gaatacggaaacactgcgct	3999054	2281	3997385	3998697	1313	JU258	None	JU263	None	None
42	D	V	attatgccaacgagcctgtc	7376106	atgccgaataatcctgttgc	7377908	1803	7376559	7377511	953	CB4856	None	JU322	None	None
43	D	V	tgccatcttctcatttgtgc	10150876	tcaatcaaaatgcacacggt	10152839	1964	10151208	10152081	874	JU258	None	KR314	None	None
44	D	V	gattcgcgtcttgtttggtt	11549898	gggatgtgtttttcttgcgt	11552437	2540	11550329	11551245	917	JU258	None	CB4853	None	None
45	D	V	tggggcaaattattgctttc	16130682	ctggtggtctccaatgtgtg	16132108	1427	16131231	16131536	306	JU258	JU258	CB4854	None	None
46	D	V	gacctagtgggtccacgaaa	16905263	cttggaaagtgtcgcactga	16906938	1676	16905927	16906402	476	JU263	None	CB4856	None	None
47	D	V	tgaaagccaagtatgtcccc	18768071	tgcagaaatgggtgttttga	18770544	2474	18769793	18770400	608	CB4856	None	CB4858	None	CB4858
48	D	V	cagctccatcatcgatcctt	19236153	aacttggagaaccttccggt	19239449	3297	19236316	19238797	2482	CB4853, CB4854, CB4858, JU258, JU263, JU322, KR314	None	CB4856	None	CB4856
49	D	V	ccaattcccataaaaggggt	19873426	agtaggttcgcaacacgtcc	19875394	1969	19874266	19874840	575	CB4856	None	JU258	None	JU258
50	D	V	actaaccgatgtttgtccgc	20436774	tcaaagccaaatcactgctg	20438410	1637	20436883	20437811	929	CB4853, CB4854, CB4858, JU258, JU263, JU322, KR314	None	CB4856	None	None
51	D	X	ccgagtatcggtgtttcgtt	4370511	cgttgcagcatggtaagaga	4373106	2596	4371018	4371601	584	CB4856	None	JU263	None	None
52	D	X	atgtcgggtagtggtcgttc	8818158	ggattaatggcacgcatttc	8820581	2424	8819702	8820075	374	CB4854, KR314	None	JU322	None	JU322
53	D	X	ttaccgcagaatgagcactg	14154767	gaatcgaaacgaagctcagg	14156765	1999	14155765	14156181	417	CB4854	None	CB4853	None	CB4854
54	A	X	tgtgttgcaatgtggtgttg	17024664	gagcctcttcccagcctagt	17026193	1530	17025111	17025553	443	CB4853, JU322	NA	CB4854	NA	None
