# Supplementary material for: Copy number variation in the genomes of twelve natural isolates of Caenorhabditis elegans
Source: BMC Genomics. 2010 Jan 25;11:62. doi: 10.1186/1471-2164-11-62 (PMC2822765; doi:10.1186/1471-2164-11-62)
Supplement: Additional file 3 — Table S2. Genes affected by copy number variants in C. elegans. [file 1471-2164-11-62-S3.PDF]

CDS	Gene	Chromosome	Gene Start	Gene Stop	Indel	Entirely Affected	Strain	Left Flanking Probe	Left Breakpoint Probe	Right Breakpoint Probe	Right Flanking Probe	Indel Length
2RSSE.1	2RSSE.1	II	15268114	15273216	A	N	CB4853	15269858	15270698	15273138	NA	2441
3R5.1	3R5.1	III	13780108	13781013	D	N	AB1	13775144	13778160	13780800	NA	2641
3R5.1	3R5.1	III	13780108	13781013	D	N	CB4856	13775144	13778160	13780800	NA	2641
3R5.1	3R5.1	III	13780108	13781013	D	N	JU263	13775144	13778160	13780800	NA	2641
6R55.2	6R55.2	X	17712786	17714744	D	Y	CB4854	17701285	17702834	17718444	NA	15611
B0047.1a	bath-20	II	2040369	2041616	D	N	CB4853	2028436	2028592	2040432	2040665	11841
B0047.1a	bath-20	II	2040369	2041616	D	Y	CB4856	2033514	2034216	2052296	2052336	18081
B0047.1a	bath-20	II	2040369	2041616	D	N	CB4858	2028545	2030102	2040432	2040665	10331
B0047.1a	bath-20	II	2040369	2041616	D	N	JU322	2036215	2038081	2041183	2041287	3103
B0047.1a	bath-20	II	2040369	2041616	D	N	KR314	2028592	2029119	2040432	2040665	11314
B0047.2	btb-3	II	2043090	2044060	D	Y	CB4856	2033514	2034216	2052296	2052336	18081
B0047.2	btb-3	II	2043090	2044060	D	N	JU258	2043261	2043479	2044703	2044760	1225
B0047.3	bath-24	II	2044703	2045586	D	Y	CB4856	2033514	2034216	2052296	2052336	18081
B0047.3	bath-24	II	2044703	2045586	D	N	JU258	2043261	2043479	2044703	2044760	1225
B0047.3	bath-24	II	2044703	2045586	D	N	JU258	2045491	2045536	2050198	2051543	4663
B0047.4	math-1	II	2045964	2046657	D	Y	CB4856	2033514	2034216	2052296	2052336	18081
B0047.4	math-1	II	2045964	2046657	D	Y	JU258	2045491	2045536	2050198	2051543	4663
B0213.10	cyp-34A5	V	3955751	3957667	D	Y	JU258	3922016	3922055	3958835	3958879	36781
B0213.11	cyp-34A6	V	3958224	3961174	D	N	JU258	3922016	3922055	3958835	3958879	36781
B0213.9	str-247	V	3954024	3955445	D	Y	JU258	3922016	3922055	3958835	3958879	36781
B0281.1	B0281.1	II	2313068	2314928	D	Y	CB4856	2302120	2306782	2318165	2318664	11384
B0281.1	B0281.1	II	2313068	2314928	D	Y	JU258	2264403	2265489	2338778	2338818	73290
B0281.1	B0281.1	II	2313068	2314928	D	Y	JU263	2302181	2306782	2318165	2318664	11384
B0281.1	B0281.1	II	2313068	2314928	D	Y	JU322	2299972	2300284	2318165	2318664	17882
B0281.1	B0281.1	II	2313068	2314928	D	Y	KR314	2302181	2306782	2318165	2318664	11384
B0281.3	B0281.3	II	2307042	2308352	D	Y	CB4856	2302120	2306782	2318165	2318664	11384
B0281.3	B0281.3	II	2307042	2308352	D	Y	JU258	2264403	2265489	2338778	2338818	73290
B0281.3	B0281.3	II	2307042	2308352	D	Y	JU263	2302181	2306782	2318165	2318664	11384
B0281.3	B0281.3	II	2307042	2308352	D	Y	JU322	2299972	2300284	2318165	2318664	17882
B0281.3	B0281.3	II	2307042	2308352	D	Y	KR314	2302181	2306782	2318165	2318664	11384
B0281.4	B0281.4	II	2297939	2298794	D	N	CB4853	2291875	2294723	2298465	2299972	3743
B0281.4	B0281.4	II	2297939	2298794	D	N	CB4856	2296036	2297945	2300284	2300410	2340
B0281.4	B0281.4	II	2297939	2298794	D	N	CB4858	2291875	2294723	2298465	2299972	3743
B0281.4	B0281.4	II	2297939	2298794	D	Y	JU258	2264403	2265489	2338778	2338818	73290
B0281.4	B0281.4	II	2297939	2298794	D	N	JU263	2292053	2294723	2298465	2299972	3743
B0281.4	B0281.4	II	2297939	2298794	D	N	JU322	2256694	2256733	2298132	2298188	41400
B0281.4	B0281.4	II	2297939	2298794	D	N	KR314	2246518	2249981	2298370	2298409	48390
B0281.5a	B0281.5	II	2299943	2300883	D	N	CB4856	2296036	2297945	2300284	2300410	2340
B0281.5a	B0281.5	II	2299943	2300883	D	Y	JU258	2264403	2265489	2338778	2338818	73290
B0281.5a	B0281.5	II	2299943	2300883	D	N	JU322	2299972	2300284	2318165	2318664	17882
B0281.6	B0281.6	II	2301402	2302230	D	Y	JU258	2264403	2265489	2338778	2338818	73290
B0281.6	B0281.6	II	2301402	2302230	D	Y	JU322	2299972	2300284	2318165	2318664	17882
B0281.8	B0281.8	II	2311491	2312848	D	Y	CB4856	2302120	2306782	2318165	2318664	11384
B0281.8	B0281.8	II	2311491	2312848	D	Y	JU258	2264403	2265489	2338778	2338818	73290
B0281.8	B0281.8	II	2311491	2312848	D	Y	JU263	2302181	2306782	2318165	2318664	11384
B0281.8	B0281.8	II	2311491	2312848	D	Y	JU322	2299972	2300284	2318165	2318664	17882
B0281.8	B0281.8	II	2311491	2312848	D	Y	KR314	2302181	2306782	2318165	2318664	11384
B0462.1	B0462.1	V	18324987	18328756	D	N	AB1	18319652	18324928	18328420	18328537	3493
B0462.1	B0462.1	V	18324987	18328756	D	N	CB4853	18319652	18324928	18328537	18328597	3610
B0462.1	B0462.1	V	18324987	18328756	D	N	CB4854	18319652	18324928	18328537	18328597	3610
B0462.1	B0462.1	V	18324987	18328756	D	N	CB4858	18319652	18324928	18328479	18328597	3552
B0462.1	B0462.1	V	18324987	18328756	D	N	JU258	18319652	18324928	18328597	18329098	3670
B0462.1	B0462.1	V	18324987	18328756	D	N	JU263	18319652	18324928	18328479	18328597	3552
B0462.1	B0462.1	V	18324987	18328756	D	N	JU322	18319652	18324928	18328479	18328597	3552
B0462.1	B0462.1	V	18324987	18328756	D	N	KR314	18319652	18324928	18328420	18328597	3493
B0462.1	B0462.1	V	18324987	18328756	D	N	MY2	18319652	18324928	18328479	18328597	3552
B0478.1a	jnk-1	IV	6957625	6963709	A	N	AB1	6960809	6960952	6961863	6962134	912
B0524.2	B0524.2	III	1894091	1899845	A	N	AB1	1892026	1893285	1895451	1897252	2167
B0524.4	B0524.4	III	1883596	1886207	D	N	AB1	1884434	1884481	1885032	1885201	552

B0524.4	B0524.4	III	1883596	1886207	D	N	CB4853	1884434	1884481	1885032	1885201	552
B0524.4	B0524.4	III	1883596	1886207	D	N	CB4858	1884434	1884434	1885032	1885201	552
B0524.4	B0524.4	III	1883596	1886207	D	N	JU258	1884434	1884481	1885032	1885201	552
B0524.4	B0524.4	III	1883596	1886207	D	N	KR314	1884434	1884481	1884969	1885032	489
B0524.5	B0524.5	III	1891267	1893831	A	N	AB1	1892026	1893285	1895451	1897252	2167
C01A2.5	C01A2.5	I	13384740	13387072	D	N	AB1	13364145	13382552	13384948	13385127	2397
C01A2.5	C01A2.5	I	13384740	13387072	D	N	CB4854	13364145	13382552	13384948	13385127	2397
C01A2.6	C01A2.6	I	13379905	13384359	D	N	AB1	13364145	13382552	13384948	13385127	2397
C01A2.6	C01A2.6	I	13379905	13384359	D	N	CB4854	13364145	13382552	13384948	13385127	2397
C03H5.1	clec-10	II	402659	404238	D	N	CB4853	400056	402707	405205	406467	2499
C03H5.1	clec-10	II	402659	404238	D	N	CB4856	400056	402707	405205	406467	2499
C03H5.1	clec-10	II	402659	404238	D	N	CB4858	400056	402707	405205	406467	2499
C04E12.10	C04E12.10	V	3377534	3381922	D	N	CB4853	3304990	3306184	3380303	3381426	74120
C04E12.10	C04E12.10	V	3377534	3381922	D	Y	CB4856	3319416	3319804	3423047	3423415	103244
C04E12.10	C04E12.10	V	3377534	3381922	D	Y	JU258	3246578	3247858	3433564	3441667	185707
C04E12.10	C04E12.10	V	3377534	3381922	D	Y	MY2	3248872	3249051	3433136	3433521	184086
C04E12.11	C04E12.11	V	3386489	3388154	D	Y	CB4856	3319416	3319804	3423047	3423415	103244
C04E12.11	C04E12.11	V	3386489	3388154	D	Y	JU258	3246578	3247858	3433564	3441667	185707
C04E12.11	C04E12.11	V	3386489	3388154	D	Y	MY2	3248872	3249051	3433136	3433521	184086
C04E12.12	C04E12.12	V	3389328	3390950	D	Y	CB4856	3319416	3319804	3423047	3423415	103244
C04E12.12	C04E12.12	V	3389328	3390950	D	Y	JU258	3246578	3247858	3433564	3441667	185707
C04E12.12	C04E12.12	V	3389328	3390950	D	Y	MY2	3248872	3249051	3433136	3433521	184086
C04E12.2	C04E12.2	V	3369079	3369875	D	Y	CB4853	3304990	3306184	3380303	3381426	74120
C04E12.2	C04E12.2	V	3369079	3369875	D	Y	CB4856	3319416	3319804	3423047	3423415	103244
C04E12.2	C04E12.2	V	3369079	3369875	D	Y	JU258	3246578	3247858	3433564	3441667	185707
C04E12.2	C04E12.2	V	3369079	3369875	D	Y	MY2	3248872	3249051	3433136	3433521	184086
C04E12.4	C04E12.4	V	3362782	3369835	D	Y	CB4853	3304990	3306184	3380303	3381426	74120
C04E12.4	C04E12.4	V	3362782	3369835	D	Y	CB4856	3319416	3319804	3423047	3423415	103244
C04E12.4	C04E12.4	V	3362782	3369835	D	Y	JU258	3246578	3247858	3433564	3441667	185707
C04E12.4	C04E12.4	V	3362782	3369835	D	Y	MY2	3248872	3249051	3433136	3433521	184086
C04E12.5	C04E12.5	V	3358641	3361091	D	Y	CB4853	3304990	3306184	3380303	3381426	74120
C04E12.5	C04E12.5	V	3358641	3361091	D	Y	CB4856	3319416	3319804	3423047	3423415	103244
C04E12.5	C04E12.5	V	3358641	3361091	D	Y	JU258	3246578	3247858	3433564	3441667	185707
C04E12.5	C04E12.5	V	3358641	3361091	D	Y	MY2	3248872	3249051	3433136	3433521	184086
C04E12.6	C04E12.6	V	3354667	3355511	D	Y	CB4853	3304990	3306184	3380303	3381426	74120
C04E12.6	C04E12.6	V	3354667	3355511	D	Y	CB4856	3319416	3319804	3423047	3423415	103244
C04E12.6	C04E12.6	V	3354667	3355511	D	Y	JU258	3246578	3247858	3433564	3441667	185707
C04E12.6	C04E12.6	V	3354667	3355511	D	Y	MY2	3248872	3249051	3433136	3433521	184086
C04E12.7	scrm-3	V	3355978	3357191	D	Y	CB4853	3304990	3306184	3380303	3381426	74120
C04E12.7	scrm-3	V	3355978	3357191	D	Y	CB4856	3319416	3319804	3423047	3423415	103244
C04E12.7	scrm-3	V	3355978	3357191	D	Y	JU258	3246578	3247858	3433564	3441667	185707
C04E12.7	scrm-3	V	3355978	3357191	D	Y	MY2	3248872	3249051	3433136	3433521	184086
C04E12.8	srx-121	V	3370940	3372771	D	Y	CB4853	3304990	3306184	3380303	3381426	74120
C04E12.8	srx-121	V	3370940	3372771	D	Y	CB4856	3319416	3319804	3423047	3423415	103244
C04E12.8	srx-121	V	3370940	3372771	D	Y	JU258	3246578	3247858	3433564	3441667	185707
C04E12.8	srx-121	V	3370940	3372771	D	Y	MY2	3248872	3249051	3433136	3433521	184086
C04E12.9	srbc-2	V	3374360	3377074	D	Y	CB4853	3304990	3306184	3380303	3381426	74120
C04E12.9	srbc-2	V	3374360	3377074	D	Y	CB4856	3319416	3319804	3423047	3423415	103244
C04E12.9	srbc-2	V	3374360	3377074	D	Y	JU258	3246578	3247858	3433564	3441667	185707
C04E12.9	srbc-2	V	3374360	3377074	D	Y	MY2	3248872	3249051	3433136	3433521	184086
C05D12.2	C05D12.2	II	11418230	11423297	A	N	JU263	11419886	11420266	11427753	11427938	7488
C05D12.3a	C05D12.3	II	11424745	11430515	A	N	JU263	11419886	11420266	11427753	11427938	7488
C05E4.10	srj-25	V	734422	736273	D	Y	RW7000	733891	734013	737758	738006	3746
C05E4.11	srj-24	V	736805	738872	D	N	RW7000	733891	734013	737758	738006	3746
C05E4.14a	srh-2	V	731648	734281	D	N	RW7000	733891	734013	737758	738006	3746
C06C6.1	C06C6.1	V	16010683	16012085	A	Y	AB1	16003771	16004293	16024961	16025011	20669
C06C6.2	str-233	V	16008305	16009637	A	Y	AB1	16003771	16004293	16024961	16025011	20669
C06C6.3	str-231	V	16006277	16007578	A	Y	AB1	16003771	16004293	16024961	16025011	20669
C06C6.4	nhr-63	V	16002568	16004653	A	N	AB1	16003771	16004293	16024961	16025011	20669
C06C6.6	C06C6.6	V	15988727	15991580	D	N	CB4856	15990464	15991189	15994276	15994565	3088

C06C6.7	C06C6.7	V	15992179	15995580	D	N	CB4856	15990464	15991189	15994276	15994565	3088
C07D8.5	C07D8.5	X	7343995	7344499	D	N	AB1	7344290	7344339	7344441	7365655	103
C07D8.5	C07D8.5	X	7343995	7344499	D	N	CB4853	7344290	7344339	7344441	7365655	103
C07D8.5	C07D8.5	X	7343995	7344499	D	N	CB4854	7344290	7344339	7344441	7365655	103
C07D8.5	C07D8.5	X	7343995	7344499	D	N	CB4858	7344290	7344339	7344441	7365655	103
C07D8.5	C07D8.5	X	7343995	7344499	D	N	KR314	7344290	7344339	7344441	7365655	103
C07G3.3	str-227	V	3518187	3519799	D	N	JU258	3508805	3508995	3519006	3519723	10012
C07G3.3	str-227	V	3518187	3519799	D	N	MY2	3508889	3508995	3519006	3519723	10012
C07G3.4	str-226	V	3512679	3514341	D	Y	JU258	3508805	3508995	3519006	3519723	10012
C07G3.4	str-226	V	3512679	3514341	D	Y	MY2	3508889	3508995	3519006	3519723	10012
C07G3.5	str-228	V	3510176	3512036	D	Y	JU258	3508805	3508995	3519006	3519723	10012
C07G3.5	str-228	V	3510176	3512036	D	Y	MY2	3508889	3508995	3519006	3519723	10012
C07G3.6	str-225	V	3507820	3509249	D	N	JU258	3508805	3508995	3519006	3519723	10012
C07G3.6	str-225	V	3507820	3509249	D	N	MY2	3508889	3508995	3519006	3519723	10012
C08E3.10a	fbxa-158	II	1619898	1621265	D	Y	CB4853	1611460	1616122	1629408	1636623	13287
C08E3.10a	fbxa-158	II	1619898	1621265	D	Y	CB4856	1612848	1613097	1629408	1636623	16312
C08E3.10a	fbxa-158	II	1619898	1621265	D	Y	CB4858	1611460	1616122	1629408	1636623	13287
C08E3.10a	fbxa-158	II	1619898	1621265	D	Y	JU258	1612848	1612981	1629408	1636623	16428
C08E3.10a	fbxa-158	II	1619898	1621265	D	Y	JU322	1611460	1616122	1629408	1636623	13287
C08E3.10a	fbxa-158	II	1619898	1621265	D	Y	KR314	1613370	1613433	1629408	1636623	15976
C08E3.11	fbxa-159	II	1621646	1622851	D	Y	CB4853	1611460	1616122	1629408	1636623	13287
C08E3.11	fbxa-159	II	1621646	1622851	D	Y	CB4856	1612848	1613097	1629408	1636623	16312
C08E3.11	fbxa-159	II	1621646	1622851	D	Y	CB4858	1611460	1616122	1629408	1636623	13287
C08E3.11	fbxa-159	II	1621646	1622851	D	Y	JU258	1612848	1612981	1629408	1636623	16428
C08E3.11	fbxa-159	II	1621646	1622851	D	Y	JU322	1611460	1616122	1629408	1636623	13287
C08E3.11	fbxa-159	II	1621646	1622851	D	Y	KR314	1613370	1613433	1629408	1636623	15976
C08E3.12	fbxa-160	II	1623911	1624676	D	Y	CB4853	1611460	1616122	1629408	1636623	13287
C08E3.12	fbxa-160	II	1623911	1624676	D	Y	CB4856	1612848	1613097	1629408	1636623	16312
C08E3.12	fbxa-160	II	1623911	1624676	D	Y	CB4858	1611460	1616122	1629408	1636623	13287
C08E3.12	fbxa-160	II	1623911	1624676	D	Y	JU258	1612848	1612981	1629408	1636623	16428
C08E3.12	fbxa-160	II	1623911	1624676	D	Y	JU322	1611460	1616122	1629408	1636623	13287
C08E3.12	fbxa-160	II	1623911	1624676	D	Y	KR314	1613370	1613433	1629408	1636623	15976
C08E3.14	C08E3.14	II	1625970	1626887	D	Y	CB4853	1611460	1616122	1629408	1636623	13287
C08E3.14	C08E3.14	II	1625970	1626887	D	Y	CB4856	1612848	1613097	1629408	1636623	16312
C08E3.14	C08E3.14	II	1625970	1626887	D	Y	CB4858	1611460	1616122	1629408	1636623	13287
C08E3.14	C08E3.14	II	1625970	1626887	D	Y	JU258	1612848	1612981	1629408	1636623	16428
C08E3.14	C08E3.14	II	1625970	1626887	D	Y	JU322	1611460	1616122	1629408	1636623	13287
C08E3.14	C08E3.14	II	1625970	1626887	D	Y	KR314	1613370	1613433	1629408	1636623	15976
C08E3.15	C08E3.15	II	1627483	1627887	D	Y	CB4853	1611460	1616122	1629408	1636623	13287
C08E3.15	C08E3.15	II	1627483	1627887	D	Y	CB4856	1612848	1613097	1629408	1636623	16312
C08E3.15	C08E3.15	II	1627483	1627887	D	Y	CB4858	1611460	1616122	1629408	1636623	13287
C08E3.15	C08E3.15	II	1627483	1627887	D	Y	JU258	1612848	1612981	1629408	1636623	16428
C08E3.15	C08E3.15	II	1627483	1627887	D	Y	JU322	1611460	1616122	1629408	1636623	13287
C08E3.15	C08E3.15	II	1627483	1627887	D	Y	KR314	1613370	1613433	1629408	1636623	15976
C08E3.7	fbxa-164	II	1612838	1614063	D	N	CB4856	1612848	1613097	1629408	1636623	16312
C08E3.7	fbxa-164	II	1612838	1614063	D	N	JU258	1612848	1612981	1629408	1636623	16428
C08E3.7	fbxa-164	II	1612838	1614063	D	N	KR314	1613370	1613433	1629408	1636623	15976
C08E3.8	fbxa-165	II	1614797	1616467	D	N	CB4853	1611460	1616122	1629408	1636623	13287
C08E3.8	fbxa-165	II	1614797	1616467	D	Y	CB4856	1612848	1613097	1629408	1636623	16312
C08E3.8	fbxa-165	II	1614797	1616467	D	N	CB4858	1611460	1616122	1629408	1636623	13287
C08E3.8	fbxa-165	II	1614797	1616467	D	Y	JU258	1612848	1612981	1629408	1636623	16428
C08E3.8	fbxa-165	II	1614797	1616467	D	N	JU322	1611460	1616122	1629408	1636623	13287
C08E3.8	fbxa-165	II	1614797	1616467	D	Y	KR314	1613370	1613433	1629408	1636623	15976
C08E3.9	fbxa-166	II	1616916	1619008	D	Y	CB4853	1611460	1616122	1629408	1636623	13287
C08E3.9	fbxa-166	II	1616916	1619008	D	Y	CB4856	1612848	1613097	1629408	1636623	16312
C08E3.9	fbxa-166	II	1616916	1619008	D	Y	CB4858	1611460	1616122	1629408	1636623	13287
C08E3.9	fbxa-166	II	1616916	1619008	D	Y	JU258	1612848	1612981	1629408	1636623	16428
C08E3.9	fbxa-166	II	1616916	1619008	D	Y	JU322	1611460	1616122	1629408	1636623	13287
C08E3.9	fbxa-166	II	1616916	1619008	D	Y	KR314	1613370	1613433	1629408	1636623	15976
C08E8.3	C08E8.3	V	18350199	18352637	D	N	CB4856	18350149	18350897	18352552	18354995	1656

C08F1.1	math-2	II	1797855	1801380	D	Y	JU258	1750470	1750664	1822745	1822788	72082
C08F1.1	math-2	II	1797855	1801380	D	N	KR314	1799744	1800302	1822618	1822666	22317
C08F1.10	C08F1.10	II	1785270	1786280	D	N	CB4856	1762424	1762529	1785629	1785949	23101
C08F1.10	C08F1.10	II	1785270	1786280	D	Y	JU258	1750470	1750664	1822745	1822788	72082
C08F1.11	C08F1.11	II	1783458	1784445	D	Y	CB4856	1762424	1762529	1785629	1785949	23101
C08F1.11	C08F1.11	II	1783458	1784445	D	Y	JU258	1750470	1750664	1822745	1822788	72082
C08F1.3	fbxb-13	II	1789184	1790192	D	N	CB4856	1787564	1787878	1790054	1790108	2177
C08F1.3	fbxb-13	II	1789184	1790192	D	Y	JU258	1750470	1750664	1822745	1822788	72082
C08F1.3	fbxb-13	II	1789184	1790192	D	N	KR314	1781016	1787299	1790054	1790108	2756
C08F1.4a	math-3	II	1776313	1778529	D	Y	CB4856	1762424	1762529	1785629	1785949	23101
C08F1.4a	math-3	II	1776313	1778529	D	Y	JU258	1750470	1750664	1822745	1822788	72082
C08F1.4a	math-3	II	1776313	1778529	D	N	KR314	1766357	1767679	1777221	1778722	9543
C08F1.5a	math-4	II	1778775	1782310	D	Y	CB4856	1762424	1762529	1785629	1785949	23101
C08F1.5a	math-4	II	1778775	1782310	D	Y	JU258	1750470	1750664	1822745	1822788	72082
C08F1.6	C08F1.6	II	1787224	1788190	D	N	CB4856	1787564	1787878	1790054	1790108	2177
C08F1.6	C08F1.6	II	1787224	1788190	D	Y	JU258	1750470	1750664	1822745	1822788	72082
C08F1.6	C08F1.6	II	1787224	1788190	D	N	KR314	1781016	1787299	1790054	1790108	2756
C08F1.8	C08F1.8	II	1804160	1808297	D	Y	CB4853	1766357	1801449	1822360	1822666	20912
C08F1.8	C08F1.8	II	1804160	1808297	D	N	CB4856	1801303	1801449	1807444	1807483	5996
C08F1.8	C08F1.8	II	1804160	1808297	D	Y	CB4858	1766357	1801449	1822360	1822666	20912
C08F1.8	C08F1.8	II	1804160	1808297	D	Y	JU258	1750470	1750664	1822745	1822788	72082
C08F1.8	C08F1.8	II	1804160	1808297	D	Y	KR314	1799744	1800302	1822618	1822666	22317
C08G5.3	C08G5.3	II	743105	745134	D	N	CB4856	740854	743129	743412	743537	284
C09E7.8a	C09E7.8	III	6553515	6558515	D	Y	JU263	6551086	6551725	6559313	6562094	7589
C09E7.8a	C09E7.8	III	6553515	6558515	D	Y	JU322	6551086	6551725	6559313	6562094	7589
C09G12.9	tsg-101	IV	3510471	3514550	A	N	JU322	3513339	3513413	3514139	3514367	727
C10H11.3	ugt-25	I	4731605	4735280	D	N	CB4856	4729982	4730182	4732478	4732617	2297
C10H11.4	ugt-28	I	4727302	4730367	D	N	CB4856	4729982	4730182	4732478	4732617	2297
C16A11.3	C16A11.3	II	4229545	4234317	D	N	CB4853	4228960	4229046	4230619	4230713	1574
C16A11.3	C16A11.3	II	4229545	4234317	D	N	CB4858	4228960	4229046	4230619	4230713	1574
C16A11.3	C16A11.3	II	4229545	4234317	D	N	KR314	4230034	4230182	4230249	4230415	68
C16A11.4a	C16A11.4	II	4227040	4229126	D	N	CB4853	4228960	4229046	4230619	4230713	1574
C16A11.4a	C16A11.4	II	4227040	4229126	D	N	CB4858	4228960	4229046	4230619	4230713	1574
C16C4.1	C16C4.1	II	1894340	1894684	D	Y	CB4853	1823064	1825029	1934411	1934453	109383
C16C4.1	C16C4.1	II	1894340	1894684	D	Y	CB4856	1845546	1846078	1912838	1913611	66761
C16C4.1	C16C4.1	II	1894340	1894684	D	Y	CB4858	1823064	1825029	1934411	1937196	109383
C16C4.1	C16C4.1	II	1894340	1894684	D	Y	JU258	1860810	1862541	1934453	1937551	71913
C16C4.1	C16C4.1	II	1894340	1894684	D	Y	KR314	1823064	1825029	1934453	1937196	109425
C16C4.10	math-5	II	1881613	1882807	D	Y	CB4853	1823064	1825029	1934411	1934453	109383
C16C4.10	math-5	II	1881613	1882807	D	Y	CB4856	1845546	1846078	1912838	1913611	66761
C16C4.10	math-5	II	1881613	1882807	D	Y	CB4858	1823064	1825029	1934411	1937196	109383
C16C4.10	math-5	II	1881613	1882807	D	Y	JU258	1860810	1862541	1934453	1937551	71913
C16C4.10	math-5	II	1881613	1882807	D	Y	KR314	1823064	1825029	1934453	1937196	109425
C16C4.11	math-6	II	1883598	1884737	D	Y	CB4853	1823064	1825029	1934411	1934453	109383
C16C4.11	math-6	II	1883598	1884737	D	Y	CB4856	1845546	1846078	1912838	1913611	66761
C16C4.11	math-6	II	1883598	1884737	D	Y	CB4858	1823064	1825029	1934411	1937196	109383
C16C4.11	math-6	II	1883598	1884737	D	Y	JU258	1860810	1862541	1934453	1937551	71913
C16C4.11	math-6	II	1883598	1884737	D	Y	KR314	1823064	1825029	1934453	1937196	109425
C16C4.12	math-7	II	1885441	1886481	D	Y	CB4853	1823064	1825029	1934411	1934453	109383
C16C4.12	math-7	II	1885441	1886481	D	Y	CB4856	1845546	1846078	1912838	1913611	66761
C16C4.12	math-7	II	1885441	1886481	D	Y	CB4858	1823064	1825029	1934411	1937196	109383
C16C4.12	math-7	II	1885441	1886481	D	Y	JU258	1860810	1862541	1934453	1937551	71913
C16C4.12	math-7	II	1885441	1886481	D	Y	KR314	1823064	1825029	1934453	1937196	109425
C16C4.13	math-8	II	1887211	1889173	D	Y	CB4853	1823064	1825029	1934411	1934453	109383
C16C4.13	math-8	II	1887211	1889173	D	Y	CB4856	1845546	1846078	1912838	1913611	66761
C16C4.13	math-8	II	1887211	1889173	D	Y	CB4858	1823064	1825029	1934411	1937196	109383
C16C4.13	math-8	II	1887211	1889173	D	Y	JU258	1860810	1862541	1934453	1937551	71913
C16C4.13	math-8	II	1887211	1889173	D	Y	KR314	1823064	1825029	1934453	1937196	109425
C16C4.14	math-9	II	1889911	1890784	D	Y	CB4853	1823064	1825029	1934411	1934453	109383
C16C4.14	math-9	II	1889911	1890784	D	Y	CB4856	1845546	1846078	1912838	1913611	66761

C16C4.14	math-9	II	1889911	1890784	D	Y	CB4858	1823064	1825029	1934411	1937196	109383
C16C4.14	math-9	II	1889911	1890784	D	Y	JU258	1860810	1862541	1934453	1937551	71913
C16C4.14	math-9	II	1889911	1890784	D	Y	KR314	1823064	1825029	1934453	1937196	109425
C16C4.15	math-10	II	1871550	1873147	D	Y	CB4853	1823064	1825029	1934411	1934453	109383
C16C4.15	math-10	II	1871550	1873147	D	Y	CB4856	1845546	1846078	1912838	1913611	66761
C16C4.15	math-10	II	1871550	1873147	D	Y	CB4858	1823064	1825029	1934411	1937196	109383
C16C4.15	math-10	II	1871550	1873147	D	Y	JU258	1860810	1862541	1934453	1937551	71913
C16C4.15	math-10	II	1871550	1873147	D	Y	KR314	1823064	1825029	1934453	1937196	109425
C16C4.16	math-11	II	1874278	1875108	D	Y	CB4853	1823064	1825029	1934411	1934453	109383
C16C4.16	math-11	II	1874278	1875108	D	Y	CB4856	1845546	1846078	1912838	1913611	66761
C16C4.16	math-11	II	1874278	1875108	D	Y	CB4858	1823064	1825029	1934411	1937196	109383
C16C4.16	math-11	II	1874278	1875108	D	Y	JU258	1860810	1862541	1934453	1937551	71913
C16C4.16	math-11	II	1874278	1875108	D	Y	KR314	1823064	1825029	1934453	1937196	109425
C16C4.17	C16C4.17	II	1864734	1864886	D	Y	CB4853	1823064	1825029	1934411	1934453	109383
C16C4.17	C16C4.17	II	1864734	1864886	D	Y	CB4856	1845546	1846078	1912838	1913611	66761
C16C4.17	C16C4.17	II	1864734	1864886	D	Y	CB4858	1823064	1825029	1934411	1937196	109383
C16C4.17	C16C4.17	II	1864734	1864886	D	Y	JU258	1860810	1862541	1934453	1937551	71913
C16C4.17	C16C4.17	II	1864734	1864886	D	Y	KR314	1823064	1825029	1934453	1937196	109425
C16C4.3	math-13	II	1875960	1877239	D	Y	CB4853	1823064	1825029	1934411	1934453	109383
C16C4.3	math-13	II	1875960	1877239	D	Y	CB4856	1845546	1846078	1912838	1913611	66761
C16C4.3	math-13	II	1875960	1877239	D	Y	CB4858	1823064	1825029	1934411	1937196	109383
C16C4.3	math-13	II	1875960	1877239	D	Y	JU258	1860810	1862541	1934453	1937551	71913
C16C4.3	math-13	II	1875960	1877239	D	Y	KR314	1823064	1825029	1934453	1937196	109425
C16C4.4	math-14	II	1869450	1870692	D	Y	CB4853	1823064	1825029	1934411	1934453	109383
C16C4.4	math-14	II	1869450	1870692	D	Y	CB4856	1845546	1846078	1912838	1913611	66761
C16C4.4	math-14	II	1869450	1870692	D	Y	CB4858	1823064	1825029	1934411	1937196	109383
C16C4.4	math-14	II	1869450	1870692	D	Y	JU258	1860810	1862541	1934453	1937551	71913
C16C4.4	math-14	II	1869450	1870692	D	Y	KR314	1823064	1825029	1934453	1937196	109425
C16C4.5	math-15	II	1866394	1867785	D	N	CB3191	1866228	1866394	1867656	1869375	1263
C16C4.5	math-15	II	1866394	1867785	D	Y	CB4853	1823064	1825029	1934411	1934453	109383
C16C4.5	math-15	II	1866394	1867785	D	Y	CB4856	1845546	1846078	1912838	1913611	66761
C16C4.5	math-15	II	1866394	1867785	D	Y	CB4858	1823064	1825029	1934411	1937196	109383
C16C4.5	math-15	II	1866394	1867785	D	Y	JU258	1860810	1862541	1934453	1937551	71913
C16C4.5	math-15	II	1866394	1867785	D	Y	KR314	1823064	1825029	1934453	1937196	109425
C16C4.6	fbxb-98	II	1862636	1863784	D	Y	CB4853	1823064	1825029	1934411	1934453	109383
C16C4.6	fbxb-98	II	1862636	1863784	D	Y	CB4856	1845546	1846078	1912838	1913611	66761
C16C4.6	fbxb-98	II	1862636	1863784	D	Y	CB4858	1823064	1825029	1934411	1937196	109383
C16C4.6	fbxb-98	II	1862636	1863784	D	Y	JU258	1860810	1862541	1934453	1937551	71913
C16C4.6	fbxb-98	II	1862636	1863784	D	Y	KR314	1823064	1825029	1934453	1937196	109425
C16C4.7	C16C4.7	II	1858394	1861407	D	Y	CB4853	1823064	1825029	1934411	1934453	109383
C16C4.7	C16C4.7	II	1858394	1861407	D	Y	CB4856	1845546	1846078	1912838	1913611	66761
C16C4.7	C16C4.7	II	1858394	1861407	D	Y	CB4858	1823064	1825029	1934411	1937196	109383
C16C4.7	C16C4.7	II	1858394	1861407	D	Y	KR314	1823064	1825029	1934453	1937196	109425
C16C4.8	math-16	II	1877581	1878761	D	Y	CB4853	1823064	1825029	1934411	1934453	109383
C16C4.8	math-16	II	1877581	1878761	D	Y	CB4856	1845546	1846078	1912838	1913611	66761
C16C4.8	math-16	II	1877581	1878761	D	Y	CB4858	1823064	1825029	1934411	1937196	109383
C16C4.8	math-16	II	1877581	1878761	D	Y	JU258	1860810	1862541	1934453	1937551	71913
C16C4.8	math-16	II	1877581	1878761	D	Y	KR314	1823064	1825029	1934453	1937196	109425
C16C4.9	math-17	II	1879701	1880710	D	Y	CB4853	1823064	1825029	1934411	1934453	109383
C16C4.9	math-17	II	1879701	1880710	D	Y	CB4856	1845546	1846078	1912838	1913611	66761
C16C4.9	math-17	II	1879701	1880710	D	Y	CB4858	1823064	1825029	1934411	1937196	109383
C16C4.9	math-17	II	1879701	1880710	D	Y	JU258	1860810	1862541	1934453	1937551	71913
C16C4.9	math-17	II	1879701	1880710	D	Y	KR314	1823064	1825029	1934453	1937196	109425
C17B7.1	str-63	V	3351564	3352771	D	Y	CB4853	3304990	3306184	3380303	3381426	74120
C17B7.1	str-63	V	3351564	3352771	D	Y	CB4856	3319416	3319804	3423047	3423415	103244
C17B7.1	str-63	V	3351564	3352771	D	Y	JU258	3246578	3247858	3433564	3441667	185707
C17B7.1	str-63	V	3351564	3352771	D	Y	MY2	3248872	3249051	3433136	3433521	184086
C17B7.10	C17B7.10	V	3340177	3342743	D	Y	CB4853	3304990	3306184	3380303	3381426	74120
C17B7.10	C17B7.10	V	3340177	3342743	D	Y	CB4856	3319416	3319804	3423047	3423415	103244
C17B7.10	C17B7.10	V	3340177	3342743	D	Y	JU258	3246578	3247858	3433564	3441667	185707

C17B7.10	C17B7.10	V	3340177	3342743	D	Y	MY2	3248872	3249051	3433136	3433521	184086
C17B7.11	fbxa-65	V	3345342	3346482	D	Y	CB4853	3304990	3306184	3380303	3381426	74120
C17B7.11	fbxa-65	V	3345342	3346482	D	Y	CB4856	3319416	3319804	3423047	3423415	103244
C17B7.11	fbxa-65	V	3345342	3346482	D	Y	JU258	3246578	3247858	3433564	3441667	185707
C17B7.11	fbxa-65	V	3345342	3346482	D	Y	MY2	3248872	3249051	3433136	3433521	184086
C17B7.12	C17B7.12	V	3353261	3353919	D	Y	CB4853	3304990	3306184	3380303	3381426	74120
C17B7.12	C17B7.12	V	3353261	3353919	D	Y	CB4856	3319416	3319804	3423047	3423415	103244
C17B7.12	C17B7.12	V	3353261	3353919	D	Y	JU258	3246578	3247858	3433564	3441667	185707
C17B7.12	C17B7.12	V	3353261	3353919	D	Y	MY2	3248872	3249051	3433136	3433521	184086
C17B7.13	C17B7.13	V	3328654	3330422	D	Y	CB4853	3304990	3306184	3380303	3381426	74120
C17B7.13	C17B7.13	V	3328654	3330422	D	Y	CB4856	3319416	3319804	3423047	3423415	103244
C17B7.13	C17B7.13	V	3328654	3330422	D	Y	JU258	3246578	3247858	3433564	3441667	185707
C17B7.13	C17B7.13	V	3328654	3330422	D	Y	MY2	3248872	3249051	3433136	3433521	184086
C17B7.2	C17B7.2	V	3348628	3350965	D	Y	CB4853	3304990	3306184	3380303	3381426	74120
C17B7.2	C17B7.2	V	3348628	3350965	D	Y	CB4856	3319416	3319804	3423047	3423415	103244
C17B7.2	C17B7.2	V	3348628	3350965	D	Y	JU258	3246578	3247858	3433564	3441667	185707
C17B7.2	C17B7.2	V	3348628	3350965	D	Y	MY2	3248872	3249051	3433136	3433521	184086
C17B7.3	C17B7.3	V	3342802	3343863	D	Y	CB4853	3304990	3306184	3380303	3381426	74120
C17B7.3	C17B7.3	V	3342802	3343863	D	Y	CB4856	3319416	3319804	3423047	3423415	103244
C17B7.3	C17B7.3	V	3342802	3343863	D	Y	JU258	3246578	3247858	3433564	3441667	185707
C17B7.3	C17B7.3	V	3342802	3343863	D	Y	MY2	3248872	3249051	3433136	3433521	184086
C17B7.4	C17B7.4	V	3339346	3340029	D	Y	CB4853	3304990	3306184	3380303	3381426	74120
C17B7.4	C17B7.4	V	3339346	3340029	D	Y	CB4856	3319416	3319804	3423047	3423415	103244
C17B7.4	C17B7.4	V	3339346	3340029	D	Y	JU258	3246578	3247858	3433564	3441667	185707
C17B7.4	C17B7.4	V	3339346	3340029	D	Y	MY2	3248872	3249051	3433136	3433521	184086
C17B7.5	C17B7.5	V	3333639	3339003	D	Y	CB4853	3304990	3306184	3380303	3381426	74120
C17B7.5	C17B7.5	V	3333639	3339003	D	Y	CB4856	3319416	3319804	3423047	3423415	103244
C17B7.5	C17B7.5	V	3333639	3339003	D	Y	JU258	3246578	3247858	3433564	3441667	185707
C17B7.5	C17B7.5	V	3333639	3339003	D	Y	MY2	3248872	3249051	3433136	3433521	184086
C17B7.7	C17B7.7	V	3325450	3328341	D	Y	CB4853	3304990	3306184	3380303	3381426	74120
C17B7.7	C17B7.7	V	3325450	3328341	D	Y	CB4856	3319416	3319804	3423047	3423415	103244
C17B7.7	C17B7.7	V	3325450	3328341	D	Y	JU258	3246578	3247858	3433564	3441667	185707
C17B7.7	C17B7.7	V	3325450	3328341	D	Y	MY2	3248872	3249051	3433136	3433521	184086
C17B7.8	C17B7.8	V	3320746	3324110	D	Y	CB4853	3304990	3306184	3380303	3381426	74120
C17B7.8	C17B7.8	V	3320746	3324110	D	Y	CB4856	3319416	3319804	3423047	3423415	103244
C17B7.8	C17B7.8	V	3320746	3324110	D	Y	JU258	3246578	3247858	3433564	3441667	185707
C17B7.8	C17B7.8	V	3320746	3324110	D	Y	MY2	3248872	3249051	3433136	3433521	184086
C17B7.9	C17B7.9	V	3330913	3332111	D	Y	CB4853	3304990	3306184	3380303	3381426	74120
C17B7.9	C17B7.9	V	3330913	3332111	D	Y	CB4856	3319416	3319804	3423047	3423415	103244
C17B7.9	C17B7.9	V	3330913	3332111	D	Y	JU258	3246578	3247858	3433564	3441667	185707
C17B7.9	C17B7.9	V	3330913	3332111	D	Y	MY2	3248872	3249051	3433136	3433521	184086
C17E7.1	nhr-156	V	3906126	3906932	D	N	JU258	3906298	3906677	3917411	3917762	10735
C17F4.10	srz-67	II	3235951	3237133	D	N	CB4856	3224933	3224997	3237080	3240493	12084
C17F4.10	srz-67	II	3235951	3237133	D	Y	JU258	3195432	3196064	3249275	3250319	53212
C17F4.10	srz-67	II	3235951	3237133	D	N	KR314	3224933	3224997	3237080	3241072	12084
C17F4.10	srz-67	II	3235951	3237133	D	N	MY2	3224933	3225304	3237080	3240493	11777
C17F4.11	C17F4.11	II	3237471	3238052	D	Y	JU258	3195432	3196064	3249275	3250319	53212
C17F4.12	C17F4.12	II	3243687	3244151	D	Y	JU258	3195432	3196064	3249275	3250319	53212
C17F4.2	C17F4.2	II	3248687	3249383	D	N	JU258	3195432	3196064	3249275	3250319	53212
C17F4.3	C17F4.3	II	3240540	3241722	D	Y	JU258	3195432	3196064	3249275	3250319	53212
C17F4.4	srh-297	II	3228880	3230517	D	Y	CB4856	3224933	3224997	3237080	3240493	12084
C17F4.4	srh-297	II	3228880	3230517	D	Y	JU258	3195432	3196064	3249275	3250319	53212
C17F4.4	srh-297	II	3228880	3230517	D	Y	KR314	3224933	3224997	3237080	3241072	12084
C17F4.4	srh-297	II	3228880	3230517	D	Y	MY2	3224933	3225304	3237080	3240493	11777
C17F4.5	C17F4.5	II	3246316	3248114	D	Y	JU258	3195432	3196064	3249275	3250319	53212
C17F4.7	C17F4.7	II	3245079	3246011	D	Y	JU258	3195432	3196064	3249275	3250319	53212
C17F4.8	C17F4.8	II	3230872	3234808	D	Y	CB4856	3224933	3224997	3237080	3240493	12084
C17F4.8	C17F4.8	II	3230872	3234808	D	Y	JU258	3195432	3196064	3249275	3250319	53212
C17F4.8	C17F4.8	II	3230872	3234808	D	Y	KR314	3224933	3224997	3237080	3241072	12084
C17F4.8	C17F4.8	II	3230872	3234808	D	Y	MY2	3224933	3225304	3237080	3240493	11777

C17H1.10	C17H1.10	I	13133636	13136144	D	Y	AB1	13109530	13111296	13153623	13155415	42328
C17H1.10	C17H1.10	I	13133636	13136144	D	Y	CB4854	13108633	13111296	13153623	13155415	42328
C17H1.13	C17H1.13	I	13124069	13125672	D	Y	AB1	13109530	13111296	13153623	13155415	42328
C17H1.13	C17H1.13	I	13124069	13125672	D	Y	CB4854	13108633	13111296	13153623	13155415	42328
C17H1.2	C17H1.2	I	13102779	13103047	D	Y	AB1	13084717	13084921	13104191	13107776	19271
C17H1.4	C17H1.4	I	13110522	13112418	D	N	AB1	13109530	13111296	13153623	13155415	42328
C17H1.4	C17H1.4	I	13110522	13112418	D	N	CB4854	13108633	13111296	13153623	13155415	42328
C17H1.5	C17H1.5	I	13113625	13116422	D	Y	AB1	13109530	13111296	13153623	13155415	42328
C17H1.5	C17H1.5	I	13113625	13116422	D	Y	CB4854	13108633	13111296	13153623	13155415	42328
C17H1.6	C17H1.6	I	13130796	13132452	D	Y	AB1	13109530	13111296	13153623	13155415	42328
C17H1.6	C17H1.6	I	13130796	13132452	D	Y	CB4854	13108633	13111296	13153623	13155415	42328
C17H1.7	C17H1.7	I	13136574	13138509	D	Y	AB1	13109530	13111296	13153623	13155415	42328
C17H1.7	C17H1.7	I	13136574	13138509	D	Y	CB4854	13108633	13111296	13153623	13155415	42328
C17H1.8	C17H1.8	I	13128458	13130158	D	Y	AB1	13109530	13111296	13153623	13155415	42328
C17H1.8	C17H1.8	I	13128458	13130158	D	Y	CB4854	13108633	13111296	13153623	13155415	42328
C17H1.9	C17H1.9	I	13121080	13123501	D	Y	AB1	13109530	13111296	13153623	13155415	42328
C17H1.9	C17H1.9	I	13121080	13123501	D	Y	CB4854	13108633	13111296	13153623	13155415	42328
C18D4.2a	fbxa-136	V	17521129	17527446	A	N	JU258	17510210	17521313	17545755	17547052	24443
C18D4.2a	fbxa-136	V	17521129	17527446	D	Y	KR314	17490847	17491246	17588401	17589546	97156
C18D4.3	C18D4.3	V	17532320	17532753	D	N	CB4856	17530511	17531341	17532669	17535562	1329
C18D4.3	C18D4.3	V	17532320	17532753	A	Y	JU258	17510210	17521313	17545755	17547052	24443
C18D4.3	C18D4.3	V	17532320	17532753	D	Y	KR314	17490847	17491246	17588401	17589546	97156
C18D4.4	C18D4.4	V	17530470	17531477	D	N	CB4856	17530511	17531341	17532669	17535562	1329
C18D4.4	C18D4.4	V	17530470	17531477	A	Y	JU258	17510210	17521313	17545755	17547052	24443
C18D4.4	C18D4.4	V	17530470	17531477	D	Y	KR314	17490847	17491246	17588401	17589546	97156
C18D4.5	srz-25	V	17540562	17542239	A	Y	JU258	17510210	17521313	17545755	17547052	24443
C18D4.5	srz-25	V	17540562	17542239	D	Y	KR314	17490847	17491246	17588401	17589546	97156
C18D4.6a	C18D4.6	V	17535799	17538340	A	Y	JU258	17510210	17521313	17545755	17547052	24443
C18D4.6a	C18D4.6	V	17535799	17538340	D	Y	KR314	17490847	17491246	17588401	17589546	97156
C18D4.8	C18D4.8	V	17544142	17546952	A	N	JU258	17510210	17521313	17545755	17547052	24443
C18D4.8	C18D4.8	V	17544142	17546952	D	Y	KR314	17490847	17491246	17588401	17589546	97156
C18D4.9	srz-90	V	17527996	17529877	A	Y	JU258	17510210	17521313	17545755	17547052	24443
C18D4.9	srz-90	V	17527996	17529877	D	Y	KR314	17490847	17491246	17588401	17589546	97156
C18H2.4	C18H2.4	III	7694618	7700308	D	N	CB4858	7694100	7694471	7694639	7694727	169
C24F3.5	abt-1	IV	10206164	10214851	D	N	CB4856	10207201	10207465	10208525	10208706	1061
C25A11.1	C25A11.1	X	9119711	9121588	D	N	CB4853	9116477	9119985	9120114	9120306	130
C25F9.1	srw-85	V	19429352	19431651	D	Y	AB1	19389840	19395538	19470658	19470740	75121
C25F9.1	srw-85	V	19429352	19431651	D	Y	CB4853	19389791	19399677	19470658	19470740	70982
C25F9.1	srw-85	V	19429352	19431651	D	Y	CB4854	19395932	19400613	19469469	19470658	68857
C25F9.1	srw-85	V	19429352	19431651	D	Y	CB4856	19400192	19400613	19469168	19470658	68556
C25F9.1	srw-85	V	19429352	19431651	D	Y	CB4858	19395467	19400613	19469469	19470740	68857
C25F9.1	srw-85	V	19429352	19431651	D	Y	JU258	19389840	19390251	19469469	19480048	79219
C25F9.1	srw-85	V	19429352	19431651	D	Y	JU263	19398882	19400613	19469469	19470658	68857
C25F9.1	srw-85	V	19429352	19431651	D	Y	JU322	19400192	19400613	19470658	19470740	70046
C25F9.1	srw-85	V	19429352	19431651	D	Y	KR314	19395467	19395538	19440602	19441749	45065
C25F9.1	srw-85	V	19429352	19431651	D	Y	MY2	19389840	19390251	19469469	19470740	79219
C25F9.10	C25F9.10	V	19405690	19406358	D	Y	AB1	19389840	19395538	19470658	19470740	75121
C25F9.10	C25F9.10	V	19405690	19406358	D	Y	CB4853	19389791	19399677	19470658	19470740	70982
C25F9.10	C25F9.10	V	19405690	19406358	D	Y	CB4854	19395932	19400613	19469469	19470658	68857
C25F9.10	C25F9.10	V	19405690	19406358	D	Y	CB4856	19400192	19400613	19469168	19470658	68556
C25F9.10	C25F9.10	V	19405690	19406358	D	Y	CB4858	19395467	19400613	19469469	19470740	68857
C25F9.10	C25F9.10	V	19405690	19406358	D	Y	JU258	19389840	19390251	19469469	19480048	79219
C25F9.10	C25F9.10	V	19405690	19406358	D	Y	JU263	19398882	19400613	19469469	19470658	68857
C25F9.10	C25F9.10	V	19405690	19406358	D	Y	JU322	19400192	19400613	19470658	19470740	70046
C25F9.10	C25F9.10	V	19405690	19406358	D	Y	KR314	19395467	19395538	19440602	19441749	45065
C25F9.10	C25F9.10	V	19405690	19406358	D	Y	MY2	19389840	19390251	19469469	19470740	79219
C25F9.11	C25F9.11	V	19432409	19433023	D	Y	AB1	19389840	19395538	19470658	19470740	75121
C25F9.11	C25F9.11	V	19432409	19433023	D	Y	CB4853	19389791	19399677	19470658	19470740	70982
C25F9.11	C25F9.11	V	19432409	19433023	D	Y	CB4854	19395932	19400613	19469469	19470658	68857
C25F9.11	C25F9.11	V	19432409	19433023	D	Y	CB4856	19400192	19400613	19469168	19470658	68556

C25F9.11	C25F9.11	V	19432409	19433023	D	Y	CB4858	19395467	19400613	19469469	19470740	68857
C25F9.11	C25F9.11	V	19432409	19433023	D	Y	JU258	19389840	19390251	19469469	19480048	79219
C25F9.11	C25F9.11	V	19432409	19433023	D	Y	JU263	19398882	19400613	19469469	19470658	68857
C25F9.11	C25F9.11	V	19432409	19433023	D	Y	JU322	19400192	19400613	19470658	19470740	70046
C25F9.11	C25F9.11	V	19432409	19433023	D	Y	KR314	19395467	19395538	19440602	19441749	45065
C25F9.11	C25F9.11	V	19432409	19433023	D	Y	MY2	19389840	19390251	19469469	19470740	79219
C25F9.12	C25F9.12	V	19402445	19402913	D	Y	AB1	19389840	19395538	19470658	19470740	75121
C25F9.12	C25F9.12	V	19402445	19402913	D	Y	CB4853	19389791	19399677	19470658	19470740	70982
C25F9.12	C25F9.12	V	19402445	19402913	D	Y	CB4854	19395932	19400613	19469469	19470658	68857
C25F9.12	C25F9.12	V	19402445	19402913	D	Y	CB4856	19400192	19400613	19469168	19470658	68556
C25F9.12	C25F9.12	V	19402445	19402913	D	Y	CB4858	19395467	19400613	19469469	19470740	68857
C25F9.12	C25F9.12	V	19402445	19402913	D	Y	JU258	19389840	19390251	19469469	19480048	79219
C25F9.12	C25F9.12	V	19402445	19402913	D	Y	JU263	19398882	19400613	19469469	19470658	68857
C25F9.12	C25F9.12	V	19402445	19402913	D	Y	JU322	19400192	19400613	19470658	19470740	70046
C25F9.12	C25F9.12	V	19402445	19402913	D	Y	KR314	19395467	19395538	19440602	19441749	45065
C25F9.12	C25F9.12	V	19402445	19402913	D	Y	MY2	19389840	19390251	19469469	19470740	79219
C25F9.13	C25F9.13	V	19397792	19398946	D	Y	AB1	19389840	19395538	19470658	19470740	75121
C25F9.13	C25F9.13	V	19397792	19398946	D	Y	JU258	19389840	19390251	19469469	19480048	79219
C25F9.13	C25F9.13	V	19397792	19398946	D	Y	KR314	19395467	19395538	19440602	19441749	45065
C25F9.13	C25F9.13	V	19397792	19398946	D	Y	MY2	19389840	19390251	19469469	19470740	79219
C25F9.14	C25F9.14	V	19436538	19436994	D	Y	AB1	19389840	19395538	19470658	19470740	75121
C25F9.14	C25F9.14	V	19436538	19436994	D	Y	CB4853	19389791	19399677	19470658	19470740	70982
C25F9.14	C25F9.14	V	19436538	19436994	D	Y	CB4854	19395932	19400613	19469469	19470658	68857
C25F9.14	C25F9.14	V	19436538	19436994	D	Y	CB4856	19400192	19400613	19469168	19470658	68556
C25F9.14	C25F9.14	V	19436538	19436994	D	Y	CB4858	19395467	19400613	19469469	19470740	68857
C25F9.14	C25F9.14	V	19436538	19436994	D	Y	JU258	19389840	19390251	19469469	19480048	79219
C25F9.14	C25F9.14	V	19436538	19436994	D	Y	JU263	19398882	19400613	19469469	19470658	68857
C25F9.14	C25F9.14	V	19436538	19436994	D	Y	JU322	19400192	19400613	19470658	19470740	70046
C25F9.14	C25F9.14	V	19436538	19436994	D	Y	KR314	19395467	19395538	19440602	19441749	45065
C25F9.14	C25F9.14	V	19436538	19436994	D	Y	MY2	19389840	19390251	19469469	19470740	79219
C25F9.2	C25F9.2	V	19421435	19426738	D	Y	AB1	19389840	19395538	19470658	19470740	75121
C25F9.2	C25F9.2	V	19421435	19426738	D	Y	CB4853	19389791	19399677	19470658	19470740	70982
C25F9.2	C25F9.2	V	19421435	19426738	D	Y	CB4854	19395932	19400613	19469469	19470658	68857
C25F9.2	C25F9.2	V	19421435	19426738	D	Y	CB4856	19400192	19400613	19469168	19470658	68556
C25F9.2	C25F9.2	V	19421435	19426738	D	Y	CB4858	19395467	19400613	19469469	19470740	68857
C25F9.2	C25F9.2	V	19421435	19426738	D	Y	JU258	19389840	19390251	19469469	19480048	79219
C25F9.2	C25F9.2	V	19421435	19426738	D	Y	JU263	19398882	19400613	19469469	19470658	68857
C25F9.2	C25F9.2	V	19421435	19426738	D	Y	JU322	19400192	19400613	19470658	19470740	70046
C25F9.2	C25F9.2	V	19421435	19426738	D	Y	KR314	19395467	19395538	19440602	19441749	45065
C25F9.2	C25F9.2	V	19421435	19426738	D	Y	MY2	19389840	19390251	19469469	19470740	79219
C25F9.4	C25F9.4	V	19411317	19413845	D	Y	AB1	19389840	19395538	19470658	19470740	75121
C25F9.4	C25F9.4	V	19411317	19413845	D	Y	CB4853	19389791	19399677	19470658	19470740	70982
C25F9.4	C25F9.4	V	19411317	19413845	D	Y	CB4854	19395932	19400613	19469469	19470658	68857
C25F9.4	C25F9.4	V	19411317	19413845	D	Y	CB4856	19400192	19400613	19469168	19470658	68556
C25F9.4	C25F9.4	V	19411317	19413845	D	Y	CB4858	19395467	19400613	19469469	19470740	68857
C25F9.4	C25F9.4	V	19411317	19413845	D	Y	JU258	19389840	19390251	19469469	19480048	79219
C25F9.4	C25F9.4	V	19411317	19413845	D	Y	JU263	19398882	19400613	19469469	19470658	68857
C25F9.4	C25F9.4	V	19411317	19413845	D	Y	JU322	19400192	19400613	19470658	19470740	70046
C25F9.4	C25F9.4	V	19411317	19413845	D	Y	KR314	19395467	19395538	19440602	19441749	45065
C25F9.4	C25F9.4	V	19411317	19413845	D	Y	MY2	19389840	19390251	19469469	19470740	79219
C25F9.5	C25F9.5	V	19407703	19410804	D	Y	AB1	19389840	19395538	19470658	19470740	75121
C25F9.5	C25F9.5	V	19407703	19410804	D	Y	CB4853	19389791	19399677	19470658	19470740	70982
C25F9.5	C25F9.5	V	19407703	19410804	D	Y	CB4854	19395932	19400613	19469469	19470658	68857
C25F9.5	C25F9.5	V	19407703	19410804	D	Y	CB4856	19400192	19400613	19469168	19470658	68556
C25F9.5	C25F9.5	V	19407703	19410804	D	Y	CB4858	19395467	19400613	19469469	19470740	68857
C25F9.5	C25F9.5	V	19407703	19410804	D	Y	JU258	19389840	19390251	19469469	19480048	79219
C25F9.5	C25F9.5	V	19407703	19410804	D	Y	JU263	19398882	19400613	19469469	19470658	68857
C25F9.5	C25F9.5	V	19407703	19410804	D	Y	JU322	19400192	19400613	19470658	19470740	70046
C25F9.5	C25F9.5	V	19407703	19410804	D	Y	KR314	19395467	19395538	19440602	19441749	45065
C25F9.5	C25F9.5	V	19407703	19410804	D	Y	MY2	19389840	19390251	19469469	19470740	79219

C25F9.6	C25F9.6	V	19404963	19405305	D	Y	AB1	19389840	19395538	19470658	19470740	75121
C25F9.6	C25F9.6	V	19404963	19405305	D	Y	CB4853	19389791	19399677	19470658	19470740	70982
C25F9.6	C25F9.6	V	19404963	19405305	D	Y	CB4854	19395932	19400613	19469469	19470658	68857
C25F9.6	C25F9.6	V	19404963	19405305	D	Y	CB4856	19400192	19400613	19469168	19470658	68556
C25F9.6	C25F9.6	V	19404963	19405305	D	Y	CB4858	19395467	19400613	19469469	19470740	68857
C25F9.6	C25F9.6	V	19404963	19405305	D	Y	JU258	19389840	19390251	19469469	19480048	79219
C25F9.6	C25F9.6	V	19404963	19405305	D	Y	JU263	19398882	19400613	19469469	19470658	68857
C25F9.6	C25F9.6	V	19404963	19405305	D	Y	JU322	19400192	19400613	19470658	19470740	70046
C25F9.6	C25F9.6	V	19404963	19405305	D	Y	KR314	19395467	19395538	19440602	19441749	45065
C25F9.6	C25F9.6	V	19404963	19405305	D	Y	MY2	19389840	19390251	19469469	19470740	79219
C25F9.7	srw-86	V	19400106	19400679	D	Y	AB1	19389840	19395538	19470658	19470740	75121
C25F9.7	srw-86	V	19400106	19400679	D	Y	CB4853	19389791	19399677	19470658	19470740	70982
C25F9.7	srw-86	V	19400106	19400679	D	N	CB4854	19395932	19400613	19469469	19470658	68857
C25F9.7	srw-86	V	19400106	19400679	D	N	CB4856	19400192	19400613	19469168	19470658	68556
C25F9.7	srw-86	V	19400106	19400679	D	N	CB4858	19395467	19400613	19469469	19470740	68857
C25F9.7	srw-86	V	19400106	19400679	D	Y	JU258	19389840	19390251	19469469	19480048	79219
C25F9.7	srw-86	V	19400106	19400679	D	N	JU263	19398882	19400613	19469469	19470658	68857
C25F9.7	srw-86	V	19400106	19400679	D	N	JU322	19400192	19400613	19470658	19470740	70046
C25F9.7	srw-86	V	19400106	19400679	D	Y	KR314	19395467	19395538	19440602	19441749	45065
C25F9.7	srw-86	V	19400106	19400679	D	Y	MY2	19389840	19390251	19469469	19470740	79219
C25F9.8	C25F9.8	V	19395019	19396573	D	N	AB1	19389840	19395538	19470658	19470740	75121
C25F9.8	C25F9.8	V	19395019	19396573	D	N	CB4854	19395467	19395538	19395797	19395932	260
C25F9.8	C25F9.8	V	19395019	19396573	D	Y	JU258	19389840	19390251	19469469	19480048	79219
C25F9.8	C25F9.8	V	19395019	19396573	D	N	KR314	19395467	19395538	19440602	19441749	45065
C25F9.8	C25F9.8	V	19395019	19396573	D	Y	MY2	19389840	19390251	19469469	19470740	79219
C25F9.9	C25F9.9	V	19416597	19417858	D	Y	AB1	19389840	19395538	19470658	19470740	75121
C25F9.9	C25F9.9	V	19416597	19417858	D	Y	CB4853	19389791	19399677	19470658	19470740	70982
C25F9.9	C25F9.9	V	19416597	19417858	D	Y	CB4854	19395932	19400613	19469469	19470658	68857
C25F9.9	C25F9.9	V	19416597	19417858	D	Y	CB4856	19400192	19400613	19469168	19470658	68556
C25F9.9	C25F9.9	V	19416597	19417858	D	Y	CB4858	19395467	19400613	19469469	19470740	68857
C25F9.9	C25F9.9	V	19416597	19417858	D	Y	JU258	19389840	19390251	19469469	19480048	79219
C25F9.9	C25F9.9	V	19416597	19417858	D	Y	JU263	19398882	19400613	19469469	19470658	68857
C25F9.9	C25F9.9	V	19416597	19417858	D	Y	JU322	19400192	19400613	19470658	19470740	70046
C25F9.9	C25F9.9	V	19416597	19417858	D	Y	KR314	19395467	19395538	19440602	19441749	45065
C25F9.9	C25F9.9	V	19416597	19417858	D	Y	MY2	19389840	19390251	19469469	19470740	79219
C26E6.5	fsn-1	III	4937301	4939415	A	N	CB4856	4937361	4937582	4938274	4938318	693
C27C7.3	nhr-74	I	11429062	11432054	D	N	MY2	11426071	11428999	11431301	11448826	2303
C27D6.8	srb-3	II	5165297	5166820	D	N	JU322	5165397	5165593	5166076	5166624	484
C27D6.8	srb-3	II	5165297	5166820	D	N	KR314	5165462	5165593	5166076	5166624	484
C29F3.1	ech-1	V	15357672	15360714	A	Y	RW7000	15282709	15282820	15399718	15400303	116899
C29F3.2	wrt-8	V	15342061	15344548	A	Y	RW7000	15282709	15282820	15399718	15400303	116899
C29F3.3	C29F3.3	V	15346700	15347364	A	Y	RW7000	15282709	15282820	15399718	15400303	116899
C29F3.4	clec-231	V	15349425	15350638	A	Y	RW7000	15282709	15282820	15399718	15400303	116899
C29F3.5	clec-230	V	15347831	15349120	A	Y	RW7000	15282709	15282820	15399718	15400303	116899
C29F3.6	srx-58	V	15352550	15353838	A	Y	RW7000	15282709	15282820	15399718	15400303	116899
C29F3.7a	C29F3.7	V	15354809	15357548	A	Y	RW7000	15282709	15282820	15399718	15400303	116899
C29F9.11	fbxa-58	III	109321	111044	A	Y	CB4854	96918	107519	116018	116122	8500
C29F9.11	fbxa-58	III	109321	111044	A	Y	JU263	96918	107519	116068	116207	8550
C29F9.11	fbxa-58	III	109321	111044	A	Y	JU322	96918	107519	116161	116207	8643
C29F9.11	fbxa-58	III	109321	111044	A	Y	KR314	107519	109257	116068	12185670	6812
C29F9.12	C29F9.12	III	121734	122019	D	Y	CB4856	118554	118609	124332	124405	5724
C29F9.3a	C29F9.3	III	124777	126462	D	N	CB4856	124453	124588	126369	130238	1782
C29F9.4	C29F9.4	III	120491	124640	D	N	CB4856	118554	118609	124332	124405	5724
C29F9.4	C29F9.4	III	120491	124640	D	N	CB4856	124453	124588	126369	130238	1782
C29F9.5	C29F9.5	III	117706	118766	D	N	CB4856	118554	118609	124332	124405	5724
C29F9.6	C29F9.6	III	115363	116618	A	N	AB1	109257	115702	115964	116068	263
C29F9.6	C29F9.6	III	115363	116618	A	N	CB4853	109257	115702	116207	116252	506
C29F9.6	C29F9.6	III	115363	116618	A	N	CB4854	96918	107519	116018	116122	8500
C29F9.6	C29F9.6	III	115363	116618	A	N	CB4856	115742	115810	116122	116207	313
C29F9.6	C29F9.6	III	115363	116618	A	N	CB4858	109257	115702	116122	116207	421

C29F9.6	C29F9.6	III	115363	116618	A	N	JU263	96918	107519	116068	116207	8550
C29F9.6	C29F9.6	III	115363	116618	A	N	JU322	96918	107519	116161	116207	8643
C29F9.6	C29F9.6	III	115363	116618	A	N	KR314	107519	109257	116068	12185670	6812
C29F9.6	C29F9.6	III	115363	116618	A	N	MY2	109257	115702	116068	116207	367
C29G2.1	C29G2.1	V	2591175	2591757	D	Y	CB4856	2584378	2590342	2591821	2592627	1480
C29G2.2	C29G2.2	V	2590392	2590820	D	Y	CB4856	2584378	2590342	2591821	2592627	1480
C30G4.2	C30G4.2	X	17044032	17045220	D	N	CB4853	17044585	17044721	17045233	17046206	513
C30G4.2	C30G4.2	X	17044032	17045220	D	N	MY2	17044585	17044721	17045233	17046206	513
C31A11.7	C31A11.7	V	16309902	16314725	D	N	RW7000	16311799	16313991	16314664	16315286	674
C31B8.4	C31B8.4	V	2896636	2898416	D	N	CB4856	2897866	2897979	2899538	2899658	1560
C31B8.6	str-46	V	2898435	2899966	D	N	CB4856	2897866	2897979	2899538	2899658	1560
C31G12.2	clec-245	V	18186076	18188225	D	N	JU258	18186176	18186552	18247813	18248607	61262
C32H11.1	C32H11.1	IV	12912759	12914925	D	Y	RW7000	12881617	12881730	12968210	12968283	86481
C32H11.10	dod-21	IV	12936721	12937917	D	Y	RW7000	12881617	12881730	12968210	12968283	86481
C32H11.11	C32H11.11	IV	12938197	12939040	D	Y	RW7000	12881617	12881730	12968210	12968283	86481
C32H11.12	dod-24	IV	12940111	12941337	D	Y	RW7000	12881617	12881730	12968210	12968283	86481
C32H11.13	dct-19	IV	12942688	12944495	D	Y	RW7000	12881617	12881730	12968210	12968283	86481
C32H11.2	srz-15	IV	12916012	12917262	D	Y	RW7000	12881617	12881730	12968210	12968283	86481
C32H11.3	C32H11.3	IV	12918580	12920189	D	Y	RW7000	12881617	12881730	12968210	12968283	86481
C32H11.4	C32H11.4	IV	12921276	12923259	D	Y	RW7000	12881617	12881730	12968210	12968283	86481
C32H11.5	C32H11.5	IV	12923494	12924347	D	Y	RW7000	12881617	12881730	12968210	12968283	86481
C32H11.6	C32H11.6	IV	12925898	12926705	D	Y	RW7000	12881617	12881730	12968210	12968283	86481
C32H11.7a	C32H11.7	IV	12930676	12931483	D	Y	RW7000	12881617	12881730	12968210	12968283	86481
C32H11.8	C32H11.8	IV	12933897	12934740	D	Y	RW7000	12881617	12881730	12968210	12968283	86481
C32H11.9	C32H11.9	IV	12935020	12936240	D	Y	RW7000	12881617	12881730	12968210	12968283	86481
C33B4.3a	shn-1	II	11384511	11390778	A	N	CB4853	11387195	11387272	11387837	11387883	566
C33B4.3a	shn-1	II	11384511	11390778	A	N	JU322	11387195	11387272	11387837	11387883	566
C33D9.6	C33D9.6	IV	8775415	8778127	D	N	JU263	8775178	8775715	8775799	8775901	85
C35D6.1	srh-228	IV	16339625	16341334	D	Y	CB4856	16280625	16286565	16345044	16347072	58480
C35D6.10	srz-71	IV	16332845	16334421	D	Y	CB4856	16280625	16286565	16345044	16347072	58480
C35D6.2	srh-227	IV	16342450	16344159	D	Y	CB4856	16280625	16286565	16345044	16347072	58480
C35D6.3	C35D6.3	IV	16347128	16347390	D	N	KR314	16341964	16345044	16347314	16350519	2271
C35D6.9a	srz-38	IV	16335297	16336463	D	Y	CB4856	16280625	16286565	16345044	16347072	58480
C38D9.1	fbxa-171	V	17563177	17565970	D	Y	KR314	17490847	17491246	17588401	17589546	97156
C38D9.2	C38D9.2	V	17566406	17571570	D	N	JU258	17567676	17568820	17591172	17592462	22353
C38D9.2	C38D9.2	V	17566406	17571570	D	Y	KR314	17490847	17491246	17588401	17589546	97156
C38D9.4	fbxa-133	V	17584760	17585843	D	Y	JU258	17567676	17568820	17591172	17592462	22353
C38D9.4	fbxa-133	V	17584760	17585843	D	Y	KR314	17490847	17491246	17588401	17589546	97156
C38D9.5	C38D9.5	V	17586819	17591942	D	N	JU258	17567676	17568820	17591172	17592462	22353
C38D9.5	C38D9.5	V	17586819	17591942	D	N	KR314	17490847	17491246	17588401	17589546	97156
C38D9.6	fbxa-172	V	17559471	17561579	D	Y	KR314	17490847	17491246	17588401	17589546	97156
C38D9.7	fbxa-174	V	17555821	17558444	D	Y	KR314	17490847	17491246	17588401	17589546	97156
C38D9.8	C38D9.8	V	17551473	17552783	D	Y	KR314	17490847	17491246	17588401	17589546	97156
C38D9.9	fbxa-176	V	17549688	17551163	D	Y	KR314	17490847	17491246	17588401	17589546	97156
C40A11.10	C40A11.10	II	2136555	2137301	D	Y	CB4853	2103403	2103518	2138338	2138410	34821
C40A11.10	C40A11.10	II	2136555	2137301	D	Y	CB4858	2103403	2103518	2138338	2138410	34821
C40A11.10	C40A11.10	II	2136555	2137301	D	Y	JU258	2063261	2063759	2138338	2138578	74580
C40A11.10	C40A11.10	II	2136555	2137301	D	Y	KR314	2103403	2103518	2138338	2138410	34821
C40A11.4	C40A11.4	II	2137326	2138952	D	N	CB4853	2103403	2103518	2138338	2138410	34821
C40A11.4	C40A11.4	II	2137326	2138952	D	N	CB4858	2103403	2103518	2138338	2138410	34821
C40A11.4	C40A11.4	II	2137326	2138952	D	N	JU258	2063261	2063759	2138338	2138578	74580
C40A11.4	C40A11.4	II	2137326	2138952	D	N	KR314	2103403	2103518	2138338	2138410	34821
C40A11.5	C40A11.5	II	2133667	2135705	D	Y	CB4853	2103403	2103518	2138338	2138410	34821
C40A11.5	C40A11.5	II	2133667	2135705	D	Y	CB4858	2103403	2103518	2138338	2138410	34821
C40A11.5	C40A11.5	II	2133667	2135705	D	Y	JU258	2063261	2063759	2138338	2138578	74580
C40A11.5	C40A11.5	II	2133667	2135705	D	Y	KR314	2103403	2103518	2138338	2138410	34821
C40D2.1	math-19	II	1998862	1999659	D	Y	CB4856	1995283	1995557	2022712	2025902	27156
C40D2.2	math-20	II	1996531	1998515	D	Y	CB4856	1995283	1995557	2022712	2025902	27156
C40D2.3	math-21	II	1994048	1995657	D	N	CB4856	1995283	1995557	2022712	2025902	27156
C40D2.4	C40D2.4	II	1999744	2000821	D	N	CB4853	1959059	1999941	2012475	2013142	12535

C40D2.4	C40D2.4	II	1999744	2000821	D	Y	CB4856	1995283	1995557	2022712	2025902	27156
C40D2.4	C40D2.4	II	1999744	2000821	D	N	CB4858	1997733	1999941	2012475	2013103	12535
C40D2.4	C40D2.4	II	1999744	2000821	D	Y	JU258	1997733	1999695	2026507	2027864	26813
C40D2.4	C40D2.4	II	1999744	2000821	D	Y	KR314	1997733	1999695	2012475	2013142	12781
C42C1.7	C42C1.7	IV	12293212	12301250	D	N	AB1	12300628	12300711	12300806	12301050	96
C43D7.4	C43D7.4	V	19321865	19322451	D	Y	AB1	19316331	19318486	19322875	19329554	4390
C43D7.4	C43D7.4	V	19321865	19322451	D	Y	CB4856	19316331	19318486	19322875	19329554	4390
C43D7.5	sdz-6	V	19320588	19321167	D	Y	AB1	19316331	19318486	19322875	19329554	4390
C43D7.5	sdz-6	V	19320588	19321167	D	Y	CB4856	19316331	19318486	19322875	19329554	4390
C43D7.7	C43D7.7	V	19318997	19319424	D	Y	AB1	19316331	19318486	19322875	19329554	4390
C43D7.7	C43D7.7	V	19318997	19319424	D	Y	CB4856	19316331	19318486	19322875	19329554	4390
C44B7.11	C44B7.11	II	6894142	6897468	A	N	CB4854	6895360	6895428	6895785	6895829	358
C45H4.9	srbc-23	V	2153574	2155112	D	N	CB4856	2154142	2154181	2155113	2155473	933
C46F9.1	math-22	II	1905431	1906061	D	Y	CB4853	1823064	1825029	1934411	1934453	109383
C46F9.1	math-22	II	1905431	1906061	D	Y	CB4856	1845546	1846078	1912838	1913611	66761
C46F9.1	math-22	II	1905431	1906061	D	Y	CB4858	1823064	1825029	1934411	1937196	109383
C46F9.1	math-22	II	1905431	1906061	D	Y	JU258	1860810	1862541	1934453	1937551	71913
C46F9.1	math-22	II	1905431	1906061	D	Y	KR314	1823064	1825029	1934453	1937196	109425
C46F9.1	math-22	II	1905431	1906061	D	Y	MY2	1894685	1895971	1909021	1910290	13051
C46F9.2	math-23	II	1902283	1904448	D	Y	CB4853	1823064	1825029	1934411	1934453	109383
C46F9.2	math-23	II	1902283	1904448	D	Y	CB4856	1845546	1846078	1912838	1913611	66761
C46F9.2	math-23	II	1902283	1904448	D	Y	CB4858	1823064	1825029	1934411	1937196	109383
C46F9.2	math-23	II	1902283	1904448	D	Y	JU258	1860810	1862541	1934453	1937551	71913
C46F9.2	math-23	II	1902283	1904448	D	Y	KR314	1823064	1825029	1934453	1937196	109425
C46F9.2	math-23	II	1902283	1904448	D	Y	MY2	1894685	1895971	1909021	1910290	13051
C46F9.3	math-24	II	1900245	1901923	D	Y	CB4853	1823064	1825029	1934411	1934453	109383
C46F9.3	math-24	II	1900245	1901923	D	Y	CB4856	1845546	1846078	1912838	1913611	66761
C46F9.3	math-24	II	1900245	1901923	D	Y	CB4858	1823064	1825029	1934411	1937196	109383
C46F9.3	math-24	II	1900245	1901923	D	Y	JU258	1860810	1862541	1934453	1937551	71913
C46F9.3	math-24	II	1900245	1901923	D	Y	KR314	1823064	1825029	1934453	1937196	109425
C46F9.3	math-24	II	1900245	1901923	D	Y	MY2	1894685	1895971	1909021	1910290	13051
C46F9.4	math-25	II	1896064	1897760	D	Y	CB4853	1823064	1825029	1934411	1934453	109383
C46F9.4	math-25	II	1896064	1897760	D	Y	CB4856	1845546	1846078	1912838	1913611	66761
C46F9.4	math-25	II	1896064	1897760	D	Y	CB4858	1823064	1825029	1934411	1937196	109383
C46F9.4	math-25	II	1896064	1897760	D	Y	JU258	1860810	1862541	1934453	1937551	71913
C46F9.4	math-25	II	1896064	1897760	D	Y	KR314	1823064	1825029	1934453	1937196	109425
C46F9.4	math-25	II	1896064	1897760	D	Y	MY2	1894685	1895971	1909021	1910290	13051
C47A10.2	srh-283	V	17765315	17766747	D	N	MY2	17765315	17766115	17767516	17769373	1402
C47A10.3	srh-284	V	17767516	17769588	D	N	MY2	17765315	17766115	17767516	17769373	1402
C47A10.9	srh-287	V	17800805	17801873	D	N	JU258	17801700	17801810	17820902	17822222	19093
C48B4.9	C48B4.9	III	9562437	9563221	D	N	CB4854	9561239	9562439	9563153	9563515	715
C49G7.1	C49G7.1	V	4056150	4059051	D	N	CB4856	4057454	4057613	4060464	4060700	2852
C49G7.1	C49G7.1	V	4056150	4059051	D	N	JU258	4055038	4056166	4060003	4060061	3838
C50H11.1	C50H11.1	V	3089073	3091259	D	N	JU258	3080323	3080362	3090528	3090580	10167
C50H11.14	srt-5	V	3079448	3080891	D	N	JU258	3071569	3078021	3079707	3079920	1687
C50H11.14	srt-5	V	3079448	3080891	D	N	JU258	3080323	3080362	3090528	3090580	10167
C50H11.2	srt-8	V	3086559	3087985	D	Y	JU258	3080323	3080362	3090528	3090580	10167
C50H11.3	srt-71	V	3083644	3085214	D	Y	JU258	3080323	3080362	3090528	3090580	10167
C50H11.4	srt-7	V	3081770	3083026	D	Y	JU258	3080323	3080362	3090528	3090580	10167
C50H11.5	srt-9	V	3078071	3079299	D	Y	JU258	3071569	3078021	3079707	3079920	1687
C51E3.2	srsx-27	V	10150701	10152130	D	N	JU258	10151081	10151208	10152081	10152554	874
C52E2.1	fbxb-95	II	1854396	1855329	D	Y	CB4853	1823064	1825029	1934411	1934453	109383
C52E2.1	fbxb-95	II	1854396	1855329	D	Y	CB4856	1845546	1846078	1912838	1913611	66761
C52E2.1	fbxb-95	II	1854396	1855329	D	Y	CB4858	1823064	1825029	1934411	1937196	109383
C52E2.1	fbxb-95	II	1854396	1855329	D	Y	JU258	1823064	1825029	1855280	1860255	30252
C52E2.1	fbxb-95	II	1854396	1855329	D	Y	KR314	1823064	1825029	1934453	1937196	109425
C52E2.2	C52E2.2	II	1852810	1853076	D	Y	CB4853	1823064	1825029	1934411	1934453	109383
C52E2.2	C52E2.2	II	1852810	1853076	D	Y	CB4856	1845546	1846078	1912838	1913611	66761
C52E2.2	C52E2.2	II	1852810	1853076	D	Y	CB4858	1823064	1825029	1934411	1937196	109383
C52E2.2	C52E2.2	II	1852810	1853076	D	Y	JU258	1823064	1825029	1855280	1860255	30252

C52E2.2	C52E2.2	II	1852810	1853076	D	Y	KR314	1823064	1825029	1934453	1937196	109425
C52E2.3	C52E2.3	II	1851246	1852485	D	Y	CB4853	1823064	1825029	1934411	1934453	109383
C52E2.3	C52E2.3	II	1851246	1852485	D	Y	CB4856	1845546	1846078	1912838	1913611	66761
C52E2.3	C52E2.3	II	1851246	1852485	D	Y	CB4858	1823064	1825029	1934411	1937196	109383
C52E2.3	C52E2.3	II	1851246	1852485	D	Y	JU258	1823064	1825029	1855280	1860255	30252
C52E2.3	C52E2.3	II	1851246	1852485	D	Y	KR314	1823064	1825029	1934453	1937196	109425
C52E2.4	C52E2.4	II	1843192	1846186	D	Y	CB4853	1823064	1825029	1934411	1934453	109383
C52E2.4	C52E2.4	II	1843192	1846186	D	N	CB4856	1845546	1846078	1912838	1913611	66761
C52E2.4	C52E2.4	II	1843192	1846186	D	Y	CB4858	1823064	1825029	1934411	1937196	109383
C52E2.4	C52E2.4	II	1843192	1846186	D	Y	JU258	1823064	1825029	1855280	1860255	30252
C52E2.4	C52E2.4	II	1843192	1846186	D	Y	KR314	1823064	1825029	1934453	1937196	109425
C52E2.5	C52E2.5	II	1840978	1842945	D	Y	CB4853	1823064	1825029	1934411	1934453	109383
C52E2.5	C52E2.5	II	1840978	1842945	D	N	CB4856	1819944	1820301	1842836	1843613	22536
C52E2.5	C52E2.5	II	1840978	1842945	D	Y	CB4858	1823064	1825029	1934411	1937196	109383
C52E2.5	C52E2.5	II	1840978	1842945	D	Y	JU258	1823064	1825029	1855280	1860255	30252
C52E2.5	C52E2.5	II	1840978	1842945	D	N	JU322	1822140	1822245	1841617	1842274	19373
C52E2.5	C52E2.5	II	1840978	1842945	D	Y	KR314	1823064	1825029	1934453	1937196	109425
C52E2.6	fbxb-97	II	1846593	1848156	D	Y	CB4853	1823064	1825029	1934411	1934453	109383
C52E2.6	fbxb-97	II	1846593	1848156	D	Y	CB4856	1845546	1846078	1912838	1913611	66761
C52E2.6	fbxb-97	II	1846593	1848156	D	Y	CB4858	1823064	1825029	1934411	1937196	109383
C52E2.6	fbxb-97	II	1846593	1848156	D	Y	JU258	1823064	1825029	1855280	1860255	30252
C52E2.6	fbxb-97	II	1846593	1848156	D	Y	KR314	1823064	1825029	1934453	1937196	109425
C52E2.7	fbxb-96	II	1849432	1850400	D	Y	CB4853	1823064	1825029	1934411	1934453	109383
C52E2.7	fbxb-96	II	1849432	1850400	D	Y	CB4856	1845546	1846078	1912838	1913611	66761
C52E2.7	fbxb-96	II	1849432	1850400	D	Y	CB4858	1823064	1825029	1934411	1937196	109383
C52E2.7	fbxb-96	II	1849432	1850400	D	Y	JU258	1823064	1825029	1855280	1860255	30252
C52E2.7	fbxb-96	II	1849432	1850400	D	Y	KR314	1823064	1825029	1934453	1937196	109425
C52E2.8	C52E2.8	II	1855621	1857799	D	Y	CB4853	1823064	1825029	1934411	1934453	109383
C52E2.8	C52E2.8	II	1855621	1857799	D	Y	CB4856	1845546	1846078	1912838	1913611	66761
C52E2.8	C52E2.8	II	1855621	1857799	D	Y	CB4858	1823064	1825029	1934411	1937196	109383
C52E2.8	C52E2.8	II	1855621	1857799	D	Y	KR314	1823064	1825029	1934453	1937196	109425
C53B7.7	C53B7.7	X	6861044	6863368	D	N	JU263	6862972	6863034	6863185	6863297	152
C53B7.7	C53B7.7	X	6861044	6863368	D	N	JU322	6862871	6863034	6863185	6863297	152
C54D10.12	C54D10.12	V	12434028	12436929	D	Y	CB4856	12433370	12433622	12442121	12445198	8500
C54D10.6	srh-25	V	12432342	12433794	D	N	CB4856	12433370	12433622	12442121	12445198	8500
C54D10.7a	dct-3	V	12437476	12443739	D	N	CB4856	12433370	12433622	12442121	12445198	8500
C55A1.5	str-23	V	15644849	15646452	D	N	MY2	15642541	15643515	15646382	15648010	2868
cTel54X.1	fbxa-6	III	1271	2917	D	N	CB4856	NA	1272	2941	8706	1670
cTel55X.1a	cTel55X.1	X	17714878	17718626	D	N	CB4854	17701285	17702834	17718444	NA	15611
D1005.3	D1005.3	X	1452691	1454024	A	N	JU322	1452059	1452747	1453261	1453308	515
D1065.3	D1065.3	V	4059817	4062078	D	N	CB4856	4057454	4057613	4060464	4060700	2852
D1065.3	D1065.3	V	4059817	4062078	D	N	JU258	4055038	4056166	4060003	4060061	3838
D2062.9	sri-77	II	2603209	2605420	D	Y	AB1	2587455	2588254	2610765	2611484	22512
D2062.9	sri-77	II	2603209	2605420	D	Y	KR314	2585541	2588254	2610765	2611484	22512
E01G4.5	E01G4.5	II	13472339	13473831	D	N	CB4853	13472164	13472520	13473569	13482264	1050
E01G4.5	E01G4.5	II	13472339	13473831	D	N	CB4854	13472164	13472520	13473569	13482264	1050
E01G4.5	E01G4.5	II	13472339	13473831	D	N	CB4856	13472164	13472520	13476967	13482264	4448
E01G4.5	E01G4.5	II	13472339	13473831	D	N	CB4858	13472164	13472520	13476967	13482264	4448
E01G4.5	E01G4.5	II	13472339	13473831	D	N	JU258	13472164	13472520	13476967	13482264	4448
E03G2.3	mec-5	X	15943929	15948873	A	N	AB1	15937744	15943966	15948329	15948643	4364
E03H4.12	E03H4.12	I	12435715	12437274	D	N	CB4856	12434823	12436234	12437028	12439886	795
E03H4.2	E03H4.2	I	12403133	12404478	D	Y	MY2	12345894	12346085	12421673	12430881	75589
E03H4.3	E03H4.3	I	12405514	12406935	D	Y	MY2	12345894	12346085	12421673	12430881	75589
E03H4.4	E03H4.4	I	12407553	12411424	D	Y	MY2	12345894	12346085	12421673	12430881	75589
E03H4.5	E03H4.5	I	12413798	12415734	D	Y	MY2	12345894	12346085	12421673	12430881	75589
E03H4.6	nhr-174	I	12417375	12418789	D	Y	MY2	12345894	12346085	12421673	12430881	75589
E03H4.7	E03H4.7	I	12421228	12424400	D	N	MY2	12345894	12346085	12421673	12430881	75589
F07E5.1	fbxb-6	II	2064463	2068972	D	N	CB4853	2068600	2068808	2097680	2103041	28873
F07E5.1	fbxb-6	II	2064463	2068972	D	N	CB4858	2068600	2068808	2097680	2103041	28873
F07E5.1	fbxb-6	II	2064463	2068972	D	Y	JU258	2063261	2063759	2138338	2138578	74580

F07E5.1	fbxb-6	II	2064463	2068972	D	N	JU322	2065424	2068458	2081749	2076295	13292
F07E5.1	fbxb-6	II	2064463	2068972	D	N	KR314	2068600	2068808	2099883	2102260	31076
F07E5.2	fbxb-35	II	2051670	2052746	D	N	CB4856	2033514	2034216	2052296	2052336	18081
F07E5.4	F07E5.4	II	2050024	2051680	D	Y	CB4856	2033514	2034216	2052296	2052336	18081
F07E5.4	F07E5.4	II	2050024	2051680	D	N	JU258	2045491	2045536	2050198	2051543	4663
F07E5.6	fbxb-36	II	2059786	2063845	D	N	CB4853	2059007	2059853	2061763	2063261	1911
F07E5.6	fbxb-36	II	2059786	2063845	D	N	CB4856	2059853	2060365	2061804	2063261	1440
F07E5.6	fbxb-36	II	2059786	2063845	D	N	CB4858	2059007	2059853	2061763	2063261	1911
F07E5.6	fbxb-36	II	2059786	2063845	D	N	JU258	2063261	2063759	2138338	2138578	74580
F07E5.6	fbxb-36	II	2059786	2063845	D	N	KR314	2059007	2059853	2061804	2063261	1952
F07E5.7	F07E5.7	II	2070702	2071517	D	Y	CB4853	2068600	2068808	2097680	2103041	28873
F07E5.7	F07E5.7	II	2070702	2071517	D	Y	CB4858	2068600	2068808	2097680	2103041	28873
F07E5.7	F07E5.7	II	2070702	2071517	D	Y	JU258	2063261	2063759	2138338	2138578	74580
F07E5.7	F07E5.7	II	2070702	2071517	D	Y	JU322	2065424	2068458	2081749	2076295	13292
F07E5.7	F07E5.7	II	2070702	2071517	D	Y	KR314	2068600	2068808	2099883	2102260	31076
F07E5.8	F07E5.8	II	2072362	2075831	D	Y	CB4853	2068600	2068808	2097680	2103041	28873
F07E5.8	F07E5.8	II	2072362	2075831	D	Y	CB4858	2068600	2068808	2097680	2103041	28873
F07E5.8	F07E5.8	II	2072362	2075831	D	Y	JU258	2063261	2063759	2138338	2138578	74580
F07E5.8	F07E5.8	II	2072362	2075831	D	Y	JU322	2065424	2068458	2081749	2076295	13292
F07E5.8	F07E5.8	II	2072362	2075831	D	Y	KR314	2068600	2068808	2099883	2102260	31076
F07E5.9	F07E5.9	II	2076292	2079407	D	Y	CB4853	2068600	2068808	2097680	2103041	28873
F07E5.9	F07E5.9	II	2076292	2079407	D	Y	CB4858	2068600	2068808	2097680	2103041	28873
F07E5.9	F07E5.9	II	2076292	2079407	D	Y	JU258	2063261	2063759	2138338	2138578	74580
F07E5.9	F07E5.9	II	2076292	2079407	D	Y	JU322	2065424	2068458	2081749	2076295	13292
F07E5.9	F07E5.9	II	2076292	2079407	D	Y	KR314	2068600	2068808	2099883	2102260	31076
F07G6.3	F07G6.3	X	1723088	1724884	D	N	JU322	1720425	1723182	1723661	1727364	480
F08D12.10	sdz-9	II	2772389	2773612	D	N	MY2	2772574	2772680	2772870	2773022	191
F08D12.8	fbxb-105	II	2769794	2770888	D	N	JU258	2770245	2770338	2770461	2770511	124
F08E10.1	srh-235	V	17470190	17471631	D	Y	CB4856	17435729	17435768	17492477	17492521	56710
F08E10.1	srh-235	V	17470190	17471631	D	Y	JU258	17417977	17418058	17499690	17506165	81633
F08E10.1	srh-235	V	17470190	17471631	D	Y	KR314	17360012	17371630	17489726	17490760	118097
F08E10.1	srh-235	V	17470190	17471631	D	Y	MY2	17435729	17435768	17489726	17490760	53959
F08E10.2	srbc-61	V	17476757	17478075	D	Y	CB4856	17435729	17435768	17492477	17492521	56710
F08E10.2	srbc-61	V	17476757	17478075	D	Y	JU258	17417977	17418058	17499690	17506165	81633
F08E10.2	srbc-61	V	17476757	17478075	D	Y	KR314	17360012	17371630	17489726	17490760	118097
F08E10.2	srbc-61	V	17476757	17478075	D	Y	MY2	17435729	17435768	17489726	17490760	53959
F08E10.3	srh-123	V	17473966	17475438	D	Y	CB4856	17435729	17435768	17492477	17492521	56710
F08E10.3	srh-123	V	17473966	17475438	D	Y	JU258	17417977	17418058	17499690	17506165	81633
F08E10.3	srh-123	V	17473966	17475438	D	Y	KR314	17360012	17371630	17489726	17490760	118097
F08E10.3	srh-123	V	17473966	17475438	D	Y	MY2	17435729	17435768	17489726	17490760	53959
F08E10.6	srh-111	V	17484012	17485145	D	Y	CB4856	17435729	17435768	17492477	17492521	56710
F08E10.6	srh-111	V	17484012	17485145	D	Y	JU258	17417977	17418058	17499690	17506165	81633
F08E10.6	srh-111	V	17484012	17485145	D	Y	KR314	17360012	17371630	17489726	17490760	118097
F08E10.6	srh-111	V	17484012	17485145	D	Y	MY2	17435729	17435768	17489726	17490760	53959
F08E10.7	scl-24	V	17486398	17487172	D	Y	CB4856	17435729	17435768	17492477	17492521	56710
F08E10.7	scl-24	V	17486398	17487172	D	Y	JU258	17417977	17418058	17499690	17506165	81633
F08E10.7	scl-24	V	17486398	17487172	D	Y	KR314	17360012	17371630	17489726	17490760	118097
F08E10.7	scl-24	V	17486398	17487172	D	Y	MY2	17435729	17435768	17489726	17490760	53959
F09C6.10	F09C6.10	V	16915342	16916148	D	Y	CB4856	16912457	16912533	16921721	16922242	9189
F09C6.8	nhr-262	V	16904882	16907256	D	N	JU263	16905390	16905927	16906402	16906592	476
F09C6.9	nhr-116	V	16912203	16915319	D	N	CB4856	16912457	16912533	16921721	16922242	9189
F10E7.1	F10E7.1	II	7126696	7128865	D	Y	CB4856	7120261	7121599	7130232	7131644	8634
F10E7.11	F10E7.11	II	7120528	7121721	D	N	CB4856	7120261	7121599	7130232	7131644	8634
F10E7.2	F10E7.2	II	7122181	7124325	D	Y	CB4856	7120261	7121599	7130232	7131644	8634
F10E7.3	F10E7.3	II	7124596	7125257	D	Y	CB4856	7120261	7121599	7130232	7131644	8634
F11A5.16	F11A5.16	V	16210925	16211427	D	Y	JU258	16200953	16203778	16214890	16214948	11113
F11A5.6	F11A5.6	V	16203913	16204059	D	Y	CB4856	16200953	16203778	16209877	16214948	6100
F11A5.6	F11A5.6	V	16203913	16204059	D	Y	JU258	16200953	16203778	16214890	16214948	11113
F11A5.6	F11A5.6	V	16203913	16204059	D	Y	MY2	16200953	16203778	16209877	16214948	6100
F11A5.7	F11A5.7	V	16205161	16206791	D	Y	CB4856	16200953	16203778	16209877	16214948	6100

F11A5.7	F11A5.7	V	16205161	16206791	D	Y	JU258	16200953	16203778	16214890	16214948	11113
F11A5.7	F11A5.7	V	16205161	16206791	D	Y	MY2	16200953	16203778	16209877	16214948	6100
F11A5.8	F11A5.8	V	16206916	16209876	D	Y	CB4856	16200953	16203778	16209877	16214948	6100
F11A5.8	F11A5.8	V	16206916	16209876	D	Y	JU258	16200953	16203778	16214890	16214948	11113
F11A5.8	F11A5.8	V	16206916	16209876	D	Y	MY2	16200953	16203778	16209877	16214948	6100
F11A5.9	F11A5.9	V	16214863	16217672	D	N	JU258	16200953	16203778	16214890	16214948	11113
F11D11.1	clec-255	V	18769732	18770688	D	N	CB4856	18768316	18769793	18770400	18770448	608
F11D11.1	clec-255	V	18769732	18770688	D	N	JU258	18751971	18752576	18769922	18770085	17347
F11D11.1	clec-255	V	18769732	18770688	D	N	JU258	18770198	18770400	18776232	18776304	5833
F11D11.12	F11D11.12	V	18749544	18750656	D	N	AB1	18747013	18750255	18750461	18751341	207
F11D11.12	F11D11.12	V	18749544	18750656	D	N	CB4854	18747013	18750255	18750461	18751341	207
F11D11.12	F11D11.12	V	18749544	18750656	D	N	JU258	18747013	18750255	18750461	18751380	207
F11D11.12	F11D11.12	V	18749544	18750656	D	N	JU263	18747013	18750255	18750461	18751341	207
F11D11.12	F11D11.12	V	18749544	18750656	D	N	KR314	18747013	18750255	18750461	18751341	207
F11D11.3	F11D11.3	V	18757947	18760234	D	Y	AB1	18751971	18752576	18768216	18769880	15641
F11D11.3	F11D11.3	V	18757947	18760234	D	Y	CB4854	18751971	18752576	18768216	18769793	15641
F11D11.3	F11D11.3	V	18757947	18760234	D	Y	JU258	18751971	18752576	18769922	18770085	17347
F11D11.3	F11D11.3	V	18757947	18760234	D	Y	JU263	18751971	18752576	18768316	18769793	15741
F11D11.3	F11D11.3	V	18757947	18760234	D	Y	KR314	18751971	18752576	18768216	18769880	15641
F11D11.4	F11D11.4	V	18761343	18763491	D	Y	AB1	18751971	18752576	18768216	18769880	15641
F11D11.4	F11D11.4	V	18761343	18763491	D	Y	CB4854	18751971	18752576	18768216	18769793	15641
F11D11.4	F11D11.4	V	18761343	18763491	D	Y	JU258	18751971	18752576	18769922	18770085	17347
F11D11.4	F11D11.4	V	18761343	18763491	D	Y	JU263	18751971	18752576	18768316	18769793	15741
F11D11.4	F11D11.4	V	18761343	18763491	D	Y	KR314	18751971	18752576	18768216	18769880	15641
F11D11.5	clec-254	V	18753465	18754393	D	Y	AB1	18751971	18752576	18768216	18769880	15641
F11D11.5	clec-254	V	18753465	18754393	D	Y	CB4854	18751971	18752576	18768216	18769793	15641
F11D11.5	clec-254	V	18753465	18754393	D	Y	JU258	18751971	18752576	18769922	18770085	17347
F11D11.5	clec-254	V	18753465	18754393	D	Y	JU263	18751971	18752576	18768316	18769793	15741
F11D11.5	clec-254	V	18753465	18754393	D	Y	KR314	18751971	18752576	18768216	18769880	15641
F11D11.6	F11D11.6	V	18752447	18753101	D	N	AB1	18751971	18752576	18768216	18769880	15641
F11D11.6	F11D11.6	V	18752447	18753101	D	N	CB4854	18751971	18752576	18768216	18769793	15641
F11D11.6	F11D11.6	V	18752447	18753101	D	N	JU258	18751971	18752576	18769922	18770085	17347
F11D11.6	F11D11.6	V	18752447	18753101	D	N	JU263	18751971	18752576	18768316	18769793	15741
F11D11.6	F11D11.6	V	18752447	18753101	D	N	KR314	18751971	18752576	18768216	18769880	15641
F12E12.10	fbxb-90	II	3747778	3748790	D	N	AB1	3748451	3748499	3757270	3757743	8772
F12E12.10	fbxb-90	II	3747778	3748790	D	N	CB4854	3748451	3748499	3757270	3757743	8772
F12E12.10	fbxb-90	II	3747778	3748790	D	N	CB4856	3748451	3748582	3757270	3757743	8689
F12E12.10	fbxb-90	II	3747778	3748790	D	N	JU258	3748451	3748499	3759197	3760463	10699
F12E12.10	fbxb-90	II	3747778	3748790	D	N	JU263	3748538	3748582	3757270	3757743	8689
F12E12.10	fbxb-90	II	3747778	3748790	D	N	JU322	3748451	3748499	3759029	3760463	10531
F12E12.10	fbxb-90	II	3747778	3748790	D	N	KR314	3748451	3748499	3759029	3759158	10531
F12E12.10	fbxb-90	II	3747778	3748790	D	N	MY2	3748358	3748499	3759029	3760463	10531
F12E12.7	sdz-11	II	3751283	3752272	D	Y	AB1	3748451	3748499	3757270	3757743	8772
F12E12.7	sdz-11	II	3751283	3752272	D	Y	CB4854	3748451	3748499	3757270	3757743	8772
F12E12.7	sdz-11	II	3751283	3752272	D	Y	CB4856	3748451	3748582	3757270	3757743	8689
F12E12.7	sdz-11	II	3751283	3752272	D	Y	JU258	3748451	3748499	3759197	3760463	10699
F12E12.7	sdz-11	II	3751283	3752272	D	Y	JU263	3748538	3748582	3757270	3757743	8689
F12E12.7	sdz-11	II	3751283	3752272	D	Y	JU322	3748451	3748499	3759029	3760463	10531
F12E12.7	sdz-11	II	3751283	3752272	D	Y	KR314	3748451	3748499	3759029	3759158	10531
F12E12.7	sdz-11	II	3751283	3752272	D	Y	MY2	3748358	3748499	3759029	3760463	10531
F12E12.8	fbxb-91	II	3749063	3750713	D	Y	AB1	3748451	3748499	3757270	3757743	8772
F12E12.8	fbxb-91	II	3749063	3750713	D	Y	CB4854	3748451	3748499	3757270	3757743	8772
F12E12.8	fbxb-91	II	3749063	3750713	D	Y	CB4856	3748451	3748582	3757270	3757743	8689
F12E12.8	fbxb-91	II	3749063	3750713	D	Y	JU258	3748451	3748499	3759197	3760463	10699
F12E12.8	fbxb-91	II	3749063	3750713	D	Y	JU263	3748538	3748582	3757270	3757743	8689
F12E12.8	fbxb-91	II	3749063	3750713	D	Y	JU322	3748451	3748499	3759029	3760463	10531
F12E12.8	fbxb-91	II	3749063	3750713	D	Y	KR314	3748451	3748499	3759029	3759158	10531
F12E12.8	fbxb-91	II	3749063	3750713	D	Y	MY2	3748358	3748499	3759029	3760463	10531
F14D2.1	bath-27	II	3360216	3361349	D	Y	CB4856	3334109	3335696	3363341	3363381	27646
F14D2.11	F14D2.11	II	3341374	3341762	D	Y	AB1	3334109	3335696	3355966	3359702	20271

F14D2.11	F14D2.11	II	3341374	3341762	D	Y	CB4853	3334109	3335696	3342056	3343105	6361
F14D2.11	F14D2.11	II	3341374	3341762	D	Y	CB4854	3334109	3335696	3342056	3343105	6361
F14D2.11	F14D2.11	II	3341374	3341762	D	Y	CB4856	3334109	3335696	3363341	3363381	27646
F14D2.11	F14D2.11	II	3341374	3341762	D	Y	CB4858	3334109	3335696	3342056	3343105	6361
F14D2.11	F14D2.11	II	3341374	3341762	D	Y	JU258	3305367	3306370	3353651	3369831	47282
F14D2.11	F14D2.11	II	3341374	3341762	D	Y	JU263	3334109	3335696	3355966	3359208	20271
F14D2.11	F14D2.11	II	3341374	3341762	D	Y	JU322	3334109	3335696	3342056	3343105	6361
F14D2.11	F14D2.11	II	3341374	3341762	D	Y	KR314	3306283	3309390	3355966	3359208	46577
F14D2.12	bath-30	II	3345275	3346366	D	Y	AB1	3334109	3335696	3355966	3359702	20271
F14D2.12	bath-30	II	3345275	3346366	D	Y	CB4856	3334109	3335696	3363341	3363381	27646
F14D2.12	bath-30	II	3345275	3346366	D	Y	JU258	3305367	3306370	3353651	3369831	47282
F14D2.12	bath-30	II	3345275	3346366	D	Y	JU263	3334109	3335696	3355966	3359208	20271
F14D2.12	bath-30	II	3345275	3346366	D	Y	KR314	3306283	3309390	3355966	3359208	46577
F14D2.13a	bath-28	II	3343732	3344962	D	Y	AB1	3334109	3335696	3355966	3359702	20271
F14D2.13a	bath-28	II	3343732	3344962	D	Y	CB4856	3334109	3335696	3363341	3363381	27646
F14D2.13a	bath-28	II	3343732	3344962	D	Y	JU258	3305367	3306370	3353651	3369831	47282
F14D2.13a	bath-28	II	3343732	3344962	D	Y	JU263	3334109	3335696	3355966	3359208	20271
F14D2.13a	bath-28	II	3343732	3344962	D	Y	KR314	3306283	3309390	3355966	3359208	46577
F14D2.14	F14D2.14	II	3346815	3347510	D	Y	AB1	3334109	3335696	3355966	3359702	20271
F14D2.14	F14D2.14	II	3346815	3347510	D	Y	CB4856	3334109	3335696	3363341	3363381	27646
F14D2.14	F14D2.14	II	3346815	3347510	D	Y	JU258	3305367	3306370	3353651	3369831	47282
F14D2.14	F14D2.14	II	3346815	3347510	D	Y	JU263	3334109	3335696	3355966	3359208	20271
F14D2.14	F14D2.14	II	3346815	3347510	D	Y	KR314	3306283	3309390	3355966	3359208	46577
F14D2.15	F14D2.15	II	3342388	3343532	D	Y	AB1	3334109	3335696	3355966	3359702	20271
F14D2.15	F14D2.15	II	3342388	3343532	D	Y	CB4856	3334109	3335696	3363341	3363381	27646
F14D2.15	F14D2.15	II	3342388	3343532	D	Y	JU258	3305367	3306370	3353651	3369831	47282
F14D2.15	F14D2.15	II	3342388	3343532	D	Y	JU263	3334109	3335696	3355966	3359208	20271
F14D2.15	F14D2.15	II	3342388	3343532	D	Y	KR314	3306283	3309390	3355966	3359208	46577
F14D2.2	F14D2.2	II	3359141	3361172	D	N	CB4854	3355966	3359208	3360123	3361904	916
F14D2.2	F14D2.2	II	3359141	3361172	D	Y	CB4856	3334109	3335696	3363341	3363381	27646
F14D2.2	F14D2.2	II	3359141	3361172	D	N	JU322	3355966	3359208	3360123	3361904	916
F14D2.4a	bath-29	II	3350254	3358965	D	N	AB1	3334109	3335696	3355966	3359702	20271
F14D2.4a	bath-29	II	3350254	3358965	D	Y	CB4856	3334109	3335696	3363341	3363381	27646
F14D2.4a	bath-29	II	3350254	3358965	D	N	JU258	3305367	3306370	3353651	3369831	47282
F14D2.4a	bath-29	II	3350254	3358965	D	N	JU263	3334109	3335696	3355966	3359208	20271
F14D2.4a	bath-29	II	3350254	3358965	D	N	KR314	3306283	3309390	3355966	3359208	46577
F14D2.5	F14D2.5	II	3340384	3341209	D	Y	AB1	3334109	3335696	3355966	3359702	20271
F14D2.5	F14D2.5	II	3340384	3341209	D	Y	CB4853	3334109	3335696	3342056	3343105	6361
F14D2.5	F14D2.5	II	3340384	3341209	D	Y	CB4854	3334109	3335696	3342056	3343105	6361
F14D2.5	F14D2.5	II	3340384	3341209	D	Y	CB4856	3334109	3335696	3363341	3363381	27646
F14D2.5	F14D2.5	II	3340384	3341209	D	Y	CB4858	3334109	3335696	3342056	3343105	6361
F14D2.5	F14D2.5	II	3340384	3341209	D	Y	JU258	3305367	3306370	3353651	3369831	47282
F14D2.5	F14D2.5	II	3340384	3341209	D	Y	JU263	3334109	3335696	3355966	3359208	20271
F14D2.5	F14D2.5	II	3340384	3341209	D	Y	JU322	3334109	3335696	3342056	3343105	6361
F14D2.5	F14D2.5	II	3340384	3341209	D	Y	KR314	3306283	3309390	3355966	3359208	46577
F14D2.6	F14D2.6	II	3329712	3334200	D	N	CB4853	3331067	3331375	3331990	3333618	616
F14D2.6	F14D2.6	II	3329712	3334200	D	N	CB4858	3331067	3331375	3333574	3333618	2200
F14D2.6	F14D2.6	II	3329712	3334200	D	Y	JU258	3305367	3306370	3353651	3369831	47282
F14D2.6	F14D2.6	II	3329712	3334200	D	N	JU322	3305367	3309390	3330254	3330712	20865
F14D2.6	F14D2.6	II	3329712	3334200	D	N	JU322	3330765	3330916	3333301	3333618	2386
F14D2.6	F14D2.6	II	3329712	3334200	D	Y	KR314	3306283	3309390	3355966	3359208	46577
F14D2.7	F14D2.7	II	3335686	3338651	D	N	AB1	3334109	3335696	3355966	3359702	20271
F14D2.7	F14D2.7	II	3335686	3338651	D	N	CB4853	3334109	3335696	3342056	3343105	6361
F14D2.7	F14D2.7	II	3335686	3338651	D	N	CB4854	3334109	3335696	3342056	3343105	6361
F14D2.7	F14D2.7	II	3335686	3338651	D	N	CB4856	3334109	3335696	3363341	3363381	27646
F14D2.7	F14D2.7	II	3335686	3338651	D	N	CB4858	3334109	3335696	3342056	3343105	6361
F14D2.7	F14D2.7	II	3335686	3338651	D	Y	JU258	3305367	3306370	3353651	3369831	47282
F14D2.7	F14D2.7	II	3335686	3338651	D	N	JU263	3334109	3335696	3355966	3359208	20271
F14D2.7	F14D2.7	II	3335686	3338651	D	N	JU322	3334109	3335696	3342056	3343105	6361
F14D2.7	F14D2.7	II	3335686	3338651	D	Y	KR314	3306283	3309390	3355966	3359208	46577

F14D2.8	F14D2.8	II	3347569	3349865	D	Y	AB1	3334109	3335696	3355966	3359702	20271
F14D2.8	F14D2.8	II	3347569	3349865	D	Y	CB4856	3334109	3335696	3363341	3363381	27646
F14D2.8	F14D2.8	II	3347569	3349865	D	Y	JU258	3305367	3306370	3353651	3369831	47282
F14D2.8	F14D2.8	II	3347569	3349865	D	Y	JU263	3334109	3335696	3355966	3359208	20271
F14D2.8	F14D2.8	II	3347569	3349865	D	Y	KR314	3306283	3309390	3355966	3359208	46577
F14D2.9	F14D2.9	II	3352496	3356298	D	N	AB1	3334109	3335696	3355966	3359702	20271
F14D2.9	F14D2.9	II	3352496	3356298	D	Y	CB4856	3334109	3335696	3363341	3363381	27646
F14D2.9	F14D2.9	II	3352496	3356298	D	N	JU258	3305367	3306370	3353651	3369831	47282
F14D2.9	F14D2.9	II	3352496	3356298	D	N	JU263	3334109	3335696	3355966	3359208	20271
F14D2.9	F14D2.9	II	3352496	3356298	D	N	KR314	3306283	3309390	3355966	3359208	46577
F14F8.6	srw-44	V	16680253	16682628	D	N	CB4856	16679243	16680253	16684769	16685304	4517
F14F8.6	srw-44	V	16680253	16682628	D	Y	CB4856	16679243	16680253	16684769	16685304	4517
F14F8.7	srw-36	V	16683158	16684850	D	N	CB4856	16679243	16680253	16684769	16685304	4517
F15A4.8a	F15A4.8	II	12475253	12478832	D	N	CB4856	12476375	12476605	12477344	12477394	740
F15D4.1	btf-1	II	13200079	13215613	A	N	JU322	13210367	13212767	13213527	13213571	761
F15D4.5	F15D4.5	II	13245338	13247552	D	N	JU263	13242954	13245442	13259068	13260802	13627
F15D4.5	F15D4.5	II	13245338	13247552	D	N	KR314	13242954	13245442	13246470	13246631	1029
F15D4.5	F15D4.5	II	13245338	13247552	D	N	KR314	13246917	13246981	13259068	13260802	12088
F15D4.6	F15D4.6	II	13249116	13249762	D	Y	JU263	13242954	13245442	13259068	13260802	13627
F15D4.6	F15D4.6	II	13249116	13249762	D	Y	KR314	13246917	13246981	13259068	13260802	12088
F15E11.12	F15E11.12	V	2326679	2327262	D	N	JU258	2317083	2319179	2327141	2334409	7963
F15E11.12	F15E11.12	V	2326679	2327262	D	N	JU263	2317083	2319179	2327141	2334409	7963
F15E11.12	F15E11.12	V	2326679	2327262	D	N	MY2	2317083	2319179	2327141	2334409	7963
F15E11.14	F15E11.14	V	2323014	2323524	D	Y	JU258	2317083	2319179	2327141	2334409	7963
F15E11.14	F15E11.14	V	2323014	2323524	D	Y	JU263	2317083	2319179	2327141	2334409	7963
F15E11.14	F15E11.14	V	2323014	2323524	D	Y	MY2	2317083	2319179	2327141	2334409	7963
F15E11.15a	F15E11.15	V	2324510	2326847	D	Y	JU258	2317083	2319179	2327141	2334409	7963
F15E11.15a	F15E11.15	V	2324510	2326847	D	Y	JU263	2317083	2319179	2327141	2334409	7963
F15E11.15a	F15E11.15	V	2324510	2326847	D	Y	MY2	2317083	2319179	2327141	2334409	7963
F15H9.1	F15H9.1	I	12157141	12158702	D	N	JU258	12154324	12156903	12158631	12162368	1729
F15H9.1	F15H9.1	I	12157141	12158702	D	N	JU263	12154324	12156903	12158553	12158592	1651
F15H9.1	F15H9.1	I	12157141	12158702	D	N	KR314	12154324	12156903	12158553	12158592	1651
F16G10.10	F16G10.10	II	2389583	2391064	D	Y	JU258	2365825	2366015	2397267	2398258	31253
F16G10.10	F16G10.10	II	2389583	2391064	D	Y	KR314	2380008	2380144	2396061	2397342	15918
F16G10.11	F16G10.11	II	2391646	2393922	D	Y	JU258	2365825	2366015	2397267	2398258	31253
F16G10.11	F16G10.11	II	2391646	2393922	D	Y	KR314	2380008	2380144	2396061	2397342	15918
F16G10.13	F16G10.13	II	2394917	2395965	D	Y	JU258	2365825	2366015	2397267	2398258	31253
F16G10.13	F16G10.13	II	2394917	2395965	D	Y	KR314	2380008	2380144	2396061	2397342	15918
F16G10.14	F16G10.14	II	2397202	2398985	D	N	JU258	2365825	2366015	2397267	2398258	31253
F16G10.2	F16G10.2	II	2375274	2375992	D	Y	JU258	2365825	2366015	2397267	2398258	31253
F16G10.2	F16G10.2	II	2375274	2375992	D	Y	JU263	2365966	2366015	2377842	2379591	11828
F16G10.2	F16G10.2	II	2375274	2375992	D	Y	KR314	2374108	2375219	2377842	2379727	2624
F16G10.3	F16G10.3	II	2372391	2374182	D	N	CB4856	2356815	2357665	2374108	2375282	16444
F16G10.3	F16G10.3	II	2372391	2374182	D	Y	JU258	2365825	2366015	2397267	2398258	31253
F16G10.3	F16G10.3	II	2372391	2374182	D	Y	JU263	2365966	2366015	2377842	2379591	11828
F16G10.3	F16G10.3	II	2372391	2374182	D	N	JU322	2356815	2357665	2374108	2375282	16444
F16G10.4	F16G10.4	II	2369257	2370090	D	Y	CB4856	2356815	2357665	2374108	2375282	16444
F16G10.4	F16G10.4	II	2369257	2370090	D	Y	JU258	2365825	2366015	2397267	2398258	31253
F16G10.4	F16G10.4	II	2369257	2370090	D	Y	JU263	2365966	2366015	2377842	2379591	11828
F16G10.4	F16G10.4	II	2369257	2370090	D	Y	JU322	2356815	2357665	2374108	2375282	16444
F16G10.4	F16G10.4	II	2369257	2370090	D	N	KR314	2365825	2366015	2370011	2372450	3997
F16G10.5	F16G10.5	II	2367503	2368683	D	Y	CB4856	2356815	2357665	2374108	2375282	16444
F16G10.5	F16G10.5	II	2367503	2368683	D	Y	JU258	2365825	2366015	2397267	2398258	31253
F16G10.5	F16G10.5	II	2367503	2368683	D	Y	JU263	2365966	2366015	2377842	2379591	11828
F16G10.5	F16G10.5	II	2367503	2368683	D	Y	JU322	2356815	2357665	2374108	2375282	16444
F16G10.5	F16G10.5	II	2367503	2368683	D	Y	KR314	2365825	2366015	2370011	2372450	3997
F16G10.6	F16G10.6	II	2377200	2377852	D	Y	JU258	2365825	2366015	2397267	2398258	31253
F16G10.6	F16G10.6	II	2377200	2377852	D	N	JU263	2365966	2366015	2377842	2379591	11828
F16G10.6	F16G10.6	II	2377200	2377852	D	N	KR314	2374108	2375219	2377842	2379727	2624
F16G10.7	F16G10.7	II	2379580	2380275	D	Y	JU258	2365825	2366015	2397267	2398258	31253

F16G10.7	F16G10.7	II	2379580	2380275	D	N	KR314	2380008	2380144	2396061	2397342	15918
F16G10.8	F16G10.8	II	2381950	2383435	D	Y	JU258	2365825	2366015	2397267	2398258	31253
F16G10.8	F16G10.8	II	2381950	2383435	D	Y	KR314	2380008	2380144	2396061	2397342	15918
F16G10.9	F16G10.9	II	2384187	2386710	D	Y	JU258	2365825	2366015	2397267	2398258	31253
F16G10.9	F16G10.9	II	2384187	2386710	D	Y	KR314	2380008	2380144	2396061	2397342	15918
F16H6.1	clec-42	V	18191408	18194132	D	Y	JU258	18186176	18186552	18247813	18248607	61262
F16H6.10	F16H6.10	V	18222763	18224412	D	N	CB4856	18223164	18224107	18235017	18235065	10911
F16H6.10	F16H6.10	V	18222763	18224412	D	Y	JU258	18186176	18186552	18247813	18248607	61262
F16H6.2	clec-246	V	18189525	18191012	D	Y	JU258	18186176	18186552	18247813	18248607	61262
F16H6.3	F16H6.3	V	18195271	18197828	D	N	CB4856	18194034	18197535	18219539	18221632	22005
F16H6.3	F16H6.3	V	18195271	18197828	D	Y	JU258	18186176	18186552	18247813	18248607	61262
F16H6.4	F16H6.4	V	18198859	18200719	D	Y	CB4856	18194034	18197535	18219539	18221632	22005
F16H6.4	F16H6.4	V	18198859	18200719	D	Y	JU258	18186176	18186552	18247813	18248607	61262
F16H6.5	F16H6.5	V	18201794	18203370	D	Y	CB4856	18194034	18197535	18219539	18221632	22005
F16H6.5	F16H6.5	V	18201794	18203370	D	Y	JU258	18186176	18186552	18247813	18248607	61262
F16H6.6	F16H6.6	V	18205611	18205961	D	Y	CB4856	18194034	18197535	18219539	18221632	22005
F16H6.6	F16H6.6	V	18205611	18205961	D	Y	JU258	18186176	18186552	18247813	18248607	61262
F16H6.7	F16H6.7	V	18207147	18209050	D	Y	CB4856	18194034	18197535	18219539	18221632	22005
F16H6.7	F16H6.7	V	18207147	18209050	D	Y	JU258	18186176	18186552	18247813	18248607	61262
F16H6.8	F16H6.8	V	18213642	18216240	D	Y	CB4856	18194034	18197535	18219539	18221632	22005
F16H6.8	F16H6.8	V	18213642	18216240	D	Y	JU258	18186176	18186552	18247813	18248607	61262
F16H6.9	F16H6.9	V	18217049	18222264	D	N	CB4856	18194034	18197535	18219539	18221632	22005
F16H6.9	F16H6.9	V	18217049	18222264	D	Y	JU258	18186176	18186552	18247813	18248607	61262
F18A12.3	F18A12.3	II	3407142	3409769	D	N	CB4856	3406285	3406578	3407569	3407934	992
F18A12.4	F18A12.4	II	3403914	3406783	D	N	CB4856	3406285	3406578	3407569	3407934	992
F18A12.7	F18A12.7	II	3411420	3414464	D	N	CB4853	3413189	3413782	3414011	3415684	230
F18A12.7	F18A12.7	II	3411420	3414464	D	N	CB4854	3413189	3413782	3414011	3414219	230
F18A12.7	F18A12.7	II	3411420	3414464	D	N	CB4858	3413189	3413782	3414011	3414465	230
F18A12.7	F18A12.7	II	3411420	3414464	D	N	JU322	3413189	3413782	3414011	3415684	230
F18C5.10	F18C5.10	II	6559220	6561409	A	N	JU258	6557781	6557847	6561319	6563692	3473
F18C5.2	wrn-1	II	6554926	6559210	A	N	JU258	6557781	6557847	6561319	6563692	3473
F19B10.1	F19B10.1	II	3683171	3684539	D	Y	JU258	3672184	3672299	3693900	3694195	21602
F19B10.10	F19B10.10	II	3677529	3681409	D	N	AB1	3676046	3677609	3679721	3679768	2113
F19B10.10	F19B10.10	II	3677529	3681409	D	N	CB4856	3676046	3677609	3679471	3679768	1863
F19B10.10	F19B10.10	II	3677529	3681409	D	Y	JU258	3672184	3672299	3693900	3694195	21602
F19B10.11	F19B10.11	II	3651250	3654270	D	N	CB4856	3646235	3651258	3654207	3655642	2950
F19B10.2	F19B10.2	II	3675618	3676722	D	Y	JU258	3672184	3672299	3693900	3694195	21602
F19B10.9	sea-1	II	3671910	3673584	D	N	JU258	3672184	3672299	3693900	3694195	21602
F19B2.3	srw-39	V	20164683	20167429	D	Y	AB1	20153948	20154082	20167559	20179231	13478
F19B2.3	srw-39	V	20164683	20167429	D	Y	CB4856	20156386	20156759	20167559	20179231	10801
F19B2.3	srw-39	V	20164683	20167429	D	Y	MY2	20156820	20158156	20182007	20183240	23852
F19B2.5	F19B2.5	V	20158035	20158756	D	Y	AB1	20153948	20154082	20167559	20179231	13478
F19B2.5	F19B2.5	V	20158035	20158756	D	Y	CB4856	20156386	20156759	20167559	20179231	10801
F19B2.5	F19B2.5	V	20158035	20158756	D	N	MY2	20156820	20158156	20182007	20183240	23852
F19B2.6	F19B2.6	V	20153914	20156888	D	N	AB1	20153948	20154082	20167559	20179231	13478
F19B2.6	F19B2.6	V	20153914	20156888	D	N	CB4856	20133405	20133764	20156235	20156347	22472
F19B2.6	F19B2.6	V	20153914	20156888	D	N	CB4856	20156386	20156759	20167559	20179231	10801
F19B2.7	F19B2.7	V	20149985	20150830	D	Y	AB1	20133405	20133764	20151489	20153948	17726
F19B2.7	F19B2.7	V	20149985	20150830	D	Y	CB4856	20133405	20133764	20156235	20156347	22472
F19B2.7	F19B2.7	V	20149985	20150830	D	Y	MY2	20133405	20133764	20151489	20154392	17726
F19B2.8	srz-16	V	20179231	20182067	D	N	MY2	20156820	20158156	20182007	20183240	23852
F19C7.3	F19C7.3	IV	4597641	4598975	D	N	AB1	4589984	4594956	4597790	4599318	2835
F19C7.3	F19C7.3	IV	4597641	4598975	D	N	JU258	4589984	4594956	4597790	4599318	2835
F19C7.3	F19C7.3	IV	4597641	4598975	D	N	JU322	4589984	4594956	4597790	4599318	2835
F19C7.3	F19C7.3	IV	4597641	4598975	D	N	MY2	4589984	4594956	4597790	4599318	2835
F19C7.5	F19C7.5	IV	4595949	4596669	D	Y	AB1	4589984	4594956	4597790	4599318	2835
F19C7.5	F19C7.5	IV	4595949	4596669	D	Y	JU258	4589984	4594956	4597790	4599318	2835
F19C7.5	F19C7.5	IV	4595949	4596669	D	Y	JU322	4589984	4594956	4597790	4599318	2835
F19C7.5	F19C7.5	IV	4595949	4596669	D	Y	MY2	4589984	4594956	4597790	4599318	2835
F19C7.6	F19C7.6	IV	4595043	4595521	D	Y	AB1	4589984	4594956	4597790	4599318	2835

F19C7.6	F19C7.6	IV	4595043	4595521	D	Y	JU258	4589984	4594956	4597790	4599318	2835
F19C7.6	F19C7.6	IV	4595043	4595521	D	Y	JU322	4589984	4594956	4597790	4599318	2835
F19C7.6	F19C7.6	IV	4595043	4595521	D	Y	MY2	4589984	4594956	4597790	4599318	2835
F20B4.2	F20B4.2	X	17703768	17704781	D	Y	CB4854	17701285	17702834	17718444	NA	15611
F20B4.3	F20B4.3	X	17705314	17706753	D	Y	CB4854	17701285	17702834	17718444	NA	15611
F20B4.4	F20B4.4	X	17706792	17707424	D	Y	CB4854	17701285	17702834	17718444	NA	15611
F20E11.1	srz-48	V	17458501	17460129	D	Y	CB4856	17435729	17435768	17492477	17492521	56710
F20E11.1	srz-48	V	17458501	17460129	D	Y	JU258	17417977	17418058	17499690	17506165	81633
F20E11.1	srz-48	V	17458501	17460129	D	Y	KR314	17360012	17371630	17489726	17490760	118097
F20E11.1	srz-48	V	17458501	17460129	D	Y	MY2	17435729	17435768	17489726	17490760	53959
F20E11.10	srh-203	V	17454463	17456265	D	Y	CB4856	17435729	17435768	17492477	17492521	56710
F20E11.10	srh-203	V	17454463	17456265	D	Y	JU258	17417977	17418058	17499690	17506165	81633
F20E11.10	srh-203	V	17454463	17456265	D	Y	KR314	17360012	17371630	17489726	17490760	118097
F20E11.10	srh-203	V	17454463	17456265	D	Y	MY2	17435729	17435768	17489726	17490760	53959
F20E11.12	srh-154	V	17440524	17441742	D	Y	CB4856	17435729	17435768	17492477	17492521	56710
F20E11.12	srh-154	V	17440524	17441742	D	Y	JU258	17417977	17418058	17499690	17506165	81633
F20E11.12	srh-154	V	17440524	17441742	D	Y	KR314	17360012	17371630	17489726	17490760	118097
F20E11.12	srh-154	V	17440524	17441742	D	Y	MY2	17435729	17435768	17489726	17490760	53959
F20E11.15	srbc-27	V	17431171	17432469	D	Y	JU258	17417977	17418058	17499690	17506165	81633
F20E11.15	srbc-27	V	17431171	17432469	D	Y	KR314	17360012	17371630	17489726	17490760	118097
F20E11.17	F20E11.17	V	17468909	17469219	D	Y	CB4856	17435729	17435768	17492477	17492521	56710
F20E11.17	F20E11.17	V	17468909	17469219	D	Y	JU258	17417977	17418058	17499690	17506165	81633
F20E11.17	F20E11.17	V	17468909	17469219	D	Y	KR314	17360012	17371630	17489726	17490760	118097
F20E11.17	F20E11.17	V	17468909	17469219	D	Y	MY2	17435729	17435768	17489726	17490760	53959
F20E11.2	srsx-2	V	17436953	17438385	D	Y	CB4856	17435729	17435768	17492477	17492521	56710
F20E11.2	srsx-2	V	17436953	17438385	D	Y	JU258	17417977	17418058	17499690	17506165	81633
F20E11.2	srsx-2	V	17436953	17438385	D	Y	KR314	17360012	17371630	17489726	17490760	118097
F20E11.2	srsx-2	V	17436953	17438385	D	Y	MY2	17435729	17435768	17489726	17490760	53959
F20E11.4	str-200	V	17456587	17458137	D	Y	CB4856	17435729	17435768	17492477	17492521	56710
F20E11.4	str-200	V	17456587	17458137	D	Y	JU258	17417977	17418058	17499690	17506165	81633
F20E11.4	str-200	V	17456587	17458137	D	Y	KR314	17360012	17371630	17489726	17490760	118097
F20E11.4	str-200	V	17456587	17458137	D	Y	MY2	17435729	17435768	17489726	17490760	53959
F20E11.5	F20E11.5	V	17460781	17462400	D	Y	CB4856	17435729	17435768	17492477	17492521	56710
F20E11.5	F20E11.5	V	17460781	17462400	D	Y	JU258	17417977	17418058	17499690	17506165	81633
F20E11.5	F20E11.5	V	17460781	17462400	D	Y	KR314	17360012	17371630	17489726	17490760	118097
F20E11.5	F20E11.5	V	17460781	17462400	D	Y	MY2	17435729	17435768	17489726	17490760	53959
F20E11.6	srw-72	V	17467019	17468558	D	Y	CB4856	17435729	17435768	17492477	17492521	56710
F20E11.6	srw-72	V	17467019	17468558	D	Y	JU258	17417977	17418058	17499690	17506165	81633
F20E11.6	srw-72	V	17467019	17468558	D	Y	KR314	17360012	17371630	17489726	17490760	118097
F20E11.6	srw-72	V	17467019	17468558	D	Y	MY2	17435729	17435768	17489726	17490760	53959
F20E11.7	F20E11.7	V	17464546	17466698	D	Y	CB4856	17435729	17435768	17492477	17492521	56710
F20E11.7	F20E11.7	V	17464546	17466698	D	Y	JU258	17417977	17418058	17499690	17506165	81633
F20E11.7	F20E11.7	V	17464546	17466698	D	Y	KR314	17360012	17371630	17489726	17490760	118097
F20E11.7	F20E11.7	V	17464546	17466698	D	Y	MY2	17435729	17435768	17489726	17490760	53959
F21E9.2	F21E9.2	X	1328865	1329936	A	N	JU322	1327414	1328895	1329130	1329189	236
F22D6.1	kin-14	I	7075919	7079481	D	N	JU258	7076209	7076680	7076760	7076871	81
F22G12.1	F22G12.1	I	13139120	13141777	D	Y	AB1	13109530	13111296	13153623	13155415	42328
F22G12.1	F22G12.1	I	13139120	13141777	D	Y	CB4854	13108633	13111296	13153623	13155415	42328
F22G12.3	F22G12.3	I	13152584	13153675	D	N	AB1	13109530	13111296	13153623	13155415	42328
F22G12.3	F22G12.3	I	13152584	13153675	D	N	CB4854	13108633	13111296	13153623	13155415	42328
F22G12.7	F22G12.7	I	13143189	13145582	D	Y	AB1	13109530	13111296	13153623	13155415	42328
F22G12.7	F22G12.7	I	13143189	13145582	D	Y	CB4854	13108633	13111296	13153623	13155415	42328
F22G12.8	F22G12.8	I	13145588	13146441	D	Y	AB1	13109530	13111296	13153623	13155415	42328
F22G12.8	F22G12.8	I	13145588	13146441	D	Y	CB4854	13108633	13111296	13153623	13155415	42328
F26F2.1	F26F2.1	V	20554468	20557283	D	Y	CB4856	20553700	20554380	20570014	20570116	15635
F26F2.1	F26F2.1	V	20554468	20557283	D	Y	MY2	20553656	20554380	20570014	20570116	15635
F26F2.2	F26F2.2	V	20558050	20560054	D	Y	CB4856	20553700	20554380	20570014	20570116	15635
F26F2.2	F26F2.2	V	20558050	20560054	D	Y	MY2	20553656	20554380	20570014	20570116	15635
F26F2.3	F26F2.3	V	20560798	20561929	D	Y	CB4856	20553700	20554380	20570014	20570116	15635
F26F2.3	F26F2.3	V	20560798	20561929	D	Y	MY2	20553656	20554380	20570014	20570116	15635

F26F2.4	F26F2.4	V	20565104	20565384	D	Y	CB4856	20553700	20554380	20570014	20570116	15635
F26F2.4	F26F2.4	V	20565104	20565384	D	Y	MY2	20553656	20554380	20570014	20570116	15635
F26F2.5	F26F2.5	V	20569105	20569388	D	Y	CB4856	20553700	20554380	20570014	20570116	15635
F26F2.5	F26F2.5	V	20569105	20569388	D	Y	MY2	20553656	20554380	20570014	20570116	15635
F26F2.6	clec-263	V	20570013	20573024	D	N	CB4856	20553700	20554380	20570014	20570116	15635
F26F2.6	clec-263	V	20570013	20573024	D	N	MY2	20553656	20554380	20570014	20570116	15635
F26F2.9	F26F2.9	V	20562963	20563304	D	Y	CB4856	20553700	20554380	20570014	20570116	15635
F26F2.9	F26F2.9	V	20562963	20563304	D	Y	MY2	20553656	20554380	20570014	20570116	15635
F27B3.5	F27B3.5	III	6559039	6562835	D	N	JU263	6551086	6551725	6559313	6562094	7589
F27B3.5	F27B3.5	III	6559039	6562835	D	N	JU322	6551086	6551725	6559313	6562094	7589
F27C1.3	F27C1.3	I	5426360	5427319	D	N	JU258	5426467	5426663	5426746	5426860	84
F27C1.6	F27C1.6	I	5421317	5428460	D	N	JU258	5426467	5426663	5426746	5426860	84
F27E5.5	F27E5.5	II	10128176	10131746	D	N	MY2	10126217	10126262	10128945	10129579	2684
F27E5.8	F27E5.8	II	10125820	10127377	D	N	MY2	10126217	10126262	10128945	10129579	2684
F28A10.3	F28A10.3	II	840573	841119	D	N	CB4853	838135	838206	841065	844307	2860
F28A10.3	F28A10.3	II	840573	841119	D	N	CB4856	838135	838206	841065	844307	2860
F28A10.3	F28A10.3	II	840573	841119	D	N	CB4858	838135	838206	841065	844307	2860
F28A10.3	F28A10.3	II	840573	841119	D	N	JU258	838423	840605	840825	840979	221
F28A10.8	F28A10.8	II	837815	838476	D	N	CB4853	838135	838206	841065	844307	2860
F28A10.8	F28A10.8	II	837815	838476	D	N	CB4856	838135	838206	841065	844307	2860
F28A10.8	F28A10.8	II	837815	838476	D	N	CB4858	838135	838206	841065	844307	2860
F29A7.3	F29A7.3	II	2753299	2753859	D	N	JU258	2753459	2753523	2753890	2756021	368
F29A7.3	F29A7.3	II	2753299	2753859	D	N	KR314	2753611	2753783	2753890	2756021	108
F31D5.4	F31D5.4	II	4164480	4194452	D	N	CB4856	4175763	4176391	4178872	4180162	2482
F31E9.2	srg-44	V	17328765	17330578	D	N	CB4856	17328959	17329847	17337901	17338060	8055
F31E9.2	srg-44	V	17328765	17330578	D	Y	JU258	17229936	17238425	17347568	17348479	109144
F31E9.3	F31E9.3	V	17322811	17324486	D	Y	CB4856	17301021	17301143	17325589	17325981	24447
F31E9.3	F31E9.3	V	17322811	17324486	D	Y	JU258	17229936	17238425	17347568	17348479	109144
F31E9.4	sdz-16	V	17326422	17327935	D	Y	JU258	17229936	17238425	17347568	17348479	109144
F31E9.5	srz-58	V	17318115	17319483	D	Y	CB4856	17301021	17301143	17325589	17325981	24447
F31E9.5	srz-58	V	17318115	17319483	D	Y	JU258	17229936	17238425	17347568	17348479	109144
F31E9.6	F31E9.6	V	17313594	17315093	D	Y	CB4856	17301021	17301143	17325589	17325981	24447
F31E9.6	F31E9.6	V	17313594	17315093	D	Y	JU258	17229936	17238425	17347568	17348479	109144
F31F6.2	F31F6.2	X	14869527	14870596	D	N	JU322	14869527	14870030	14871465	14872824	1436
F32A11.2	hpr-17	II	13153369	13156955	A	N	CB4854	13155451	13155506	13156513	13156552	1008
F33E11.2	F33E11.2	V	296599	300009	D	N	AB1	298647	298710	298858	299807	149
F33E2.4	F33E2.4	I	12582275	12583288	D	N	CB4856	12582983	12583116	12583292	12585418	177
F33H12.1	F33H12.1	II	2600554	2601780	D	Y	AB1	2587455	2588254	2610765	2611484	22512
F33H12.1	F33H12.1	II	2600554	2601780	D	Y	KR314	2585541	2588254	2610765	2611484	22512
F33H12.2	sri-39	II	2587505	2589640	D	N	AB1	2587455	2588254	2610765	2611484	22512
F33H12.2	sri-39	II	2587505	2589640	D	N	KR314	2585541	2588254	2610765	2611484	22512
F33H12.4	sri-74	II	2580902	2583007	D	N	KR314	2575755	2580748	2581712	2582462	965
F33H12.4	sri-74	II	2580902	2583007	D	N	MY2	2575755	2580748	2582462	2583008	1715
F33H12.6	F33H12.6	II	2594053	2598904	D	Y	AB1	2587455	2588254	2610765	2611484	22512
F33H12.6	F33H12.6	II	2594053	2598904	D	Y	KR314	2585541	2588254	2610765	2611484	22512
F33H2.1	dog-1	I	15017266	15022967	A	Y	JU258	15016389	15016450	15028313	15028966	11864
F33H2.2	F33H2.2	I	15023127	15027739	A	Y	JU258	15016389	15016450	15028313	15028966	11864
F33H2.6	F33H2.6	I	15014838	15016582	A	N	JU258	15016389	15016450	15028313	15028966	11864
F33H2.7	set-10	I	15027704	15030537	A	N	JU258	15016389	15016450	15028313	15028966	11864
F35B3.5a	pqn-34	X	17021738	17030136	A	N	JU322	17025048	17025111	17025553	17026164	443
F35E2.3	F35E2.3	I	11734418	11735926	D	N	CB4856	11726745	11734437	11735578	11736811	1142
F35E2.5	F35E2.5	I	11741605	11743941	D	N	JU258	11741852	11741982	11742133	11742239	152
F35E2.5	F35E2.5	I	11741605	11743941	D	N	JU263	11741852	11741982	11742133	11742239	152
F35F10.1	F35F10.1	V	3316382	3318025	D	Y	CB4853	3304990	3306184	3380303	3381426	74120
F35F10.1	F35F10.1	V	3316382	3318025	D	Y	JU258	3246578	3247858	3433564	3441667	185707
F35F10.1	F35F10.1	V	3316382	3318025	D	Y	MY2	3248872	3249051	3433136	3433521	184086
F35F10.10	F35F10.10	V	3302413	3307361	D	N	CB4853	3304990	3306184	3380303	3381426	74120
F35F10.10	F35F10.10	V	3302413	3307361	D	Y	JU258	3246578	3247858	3433564	3441667	185707
F35F10.10	F35F10.10	V	3302413	3307361	D	Y	MY2	3248872	3249051	3433136	3433521	184086
F35F10.11	F35F10.11	V	3310131	3312115	D	Y	CB4853	3304990	3306184	3380303	3381426	74120

F35F10.11	F35F10.11	V	3310131	3312115	D	Y	JU258	3246578	3247858	3433564	3441667	185707
F35F10.11	F35F10.11	V	3310131	3312115	D	Y	MY2	3248872	3249051	3433136	3433521	184086
F35F10.12	F35F10.12	V	3313346	3315514	D	Y	CB4853	3304990	3306184	3380303	3381426	74120
F35F10.12	F35F10.12	V	3313346	3315514	D	Y	JU258	3246578	3247858	3433564	3441667	185707
F35F10.12	F35F10.12	V	3313346	3315514	D	Y	MY2	3248872	3249051	3433136	3433521	184086
F35F10.13	F35F10.13	V	3318206	3319060	D	Y	CB4853	3304990	3306184	3380303	3381426	74120
F35F10.13	F35F10.13	V	3318206	3319060	D	Y	JU258	3246578	3247858	3433564	3441667	185707
F35F10.13	F35F10.13	V	3318206	3319060	D	Y	MY2	3248872	3249051	3433136	3433521	184086
F35F10.14	F35F10.14	V	3319488	3319673	D	Y	CB4853	3304990	3306184	3380303	3381426	74120
F35F10.14	F35F10.14	V	3319488	3319673	D	Y	JU258	3246578	3247858	3433564	3441667	185707
F35F10.14	F35F10.14	V	3319488	3319673	D	Y	MY2	3248872	3249051	3433136	3433521	184086
F35F10.2	srbc-3	V	3308467	3309687	D	Y	CB4853	3304990	3306184	3380303	3381426	74120
F35F10.2	srbc-3	V	3308467	3309687	D	Y	JU258	3246578	3247858	3433564	3441667	185707
F35F10.2	srbc-3	V	3308467	3309687	D	Y	MY2	3248872	3249051	3433136	3433521	184086
F35F10.4	F35F10.4	V	3290833	3295121	D	Y	JU258	3246578	3247858	3433564	3441667	185707
F35F10.4	F35F10.4	V	3290833	3295121	D	Y	MY2	3248872	3249051	3433136	3433521	184086
F35F10.5	F35F10.5	V	3285020	3286203	D	Y	CB4856	3276151	3276987	3286204	3286430	9218
F35F10.5	F35F10.5	V	3285020	3286203	D	Y	JU258	3246578	3247858	3433564	3441667	185707
F35F10.5	F35F10.5	V	3285020	3286203	D	Y	MY2	3248872	3249051	3433136	3433521	184086
F35F10.6	F35F10.6	V	3288075	3289112	D	Y	JU258	3246578	3247858	3433564	3441667	185707
F35F10.6	F35F10.6	V	3288075	3289112	D	Y	MY2	3248872	3249051	3433136	3433521	184086
F35F10.7	F35F10.7	V	3286430	3287398	D	Y	JU258	3246578	3247858	3433564	3441667	185707
F35F10.7	F35F10.7	V	3286430	3287398	D	Y	MY2	3248872	3249051	3433136	3433521	184086
F35F10.8	srx-122	V	3296106	3297862	D	Y	JU258	3246578	3247858	3433564	3441667	185707
F35F10.8	srx-122	V	3296106	3297862	D	Y	MY2	3248872	3249051	3433136	3433521	184086
F35F10.9	srbc-1	V	3298883	3301996	D	Y	JU258	3246578	3247858	3433564	3441667	185707
F35F10.9	srbc-1	V	3298883	3301996	D	Y	MY2	3248872	3249051	3433136	3433521	184086
F36H5.1	math-26	II	1771650	1774958	D	Y	CB4856	1762424	1762529	1785629	1785949	23101
F36H5.1	math-26	II	1771650	1774958	D	Y	JU258	1750470	1750664	1822745	1822788	72082
F36H5.1	math-26	II	1771650	1774958	D	Y	JU322	1762424	1763379	1776263	1776507	12885
F36H5.1	math-26	II	1771650	1774958	D	Y	KR314	1766357	1767679	1777221	1778722	9543
F36H5.10	F36H5.10	II	1756823	1757764	D	N	CB4856	1750470	1750664	1757479	1757674	6816
F36H5.10	F36H5.10	II	1756823	1757764	D	Y	JU258	1750470	1750664	1822745	1822788	72082
F36H5.10	F36H5.10	II	1756823	1757764	D	N	JU322	1750423	1750664	1757479	1757674	6816
F36H5.11	fbxb-12	II	1753516	1756167	D	Y	CB4856	1750470	1750664	1757479	1757674	6816
F36H5.11	fbxb-12	II	1753516	1756167	D	Y	JU258	1750470	1750664	1822745	1822788	72082
F36H5.11	fbxb-12	II	1753516	1756167	D	Y	JU322	1750423	1750664	1757479	1757674	6816
F36H5.2a	math-27	II	1767807	1771050	D	Y	CB4856	1762424	1762529	1785629	1785949	23101
F36H5.2a	math-27	II	1767807	1771050	D	Y	JU258	1750470	1750664	1822745	1822788	72082
F36H5.2a	math-27	II	1767807	1771050	D	Y	JU322	1762424	1763379	1776263	1776507	12885
F36H5.2a	math-27	II	1767807	1771050	D	Y	KR314	1766357	1767679	1777221	1778722	9543
F36H5.3a	math-28	II	1763375	1766558	D	Y	CB4856	1762424	1762529	1785629	1785949	23101
F36H5.3a	math-28	II	1763375	1766558	D	Y	JU258	1750470	1750664	1822745	1822788	72082
F36H5.3a	math-28	II	1763375	1766558	D	N	JU322	1762424	1763379	1776263	1776507	12885
F36H5.4	F36H5.4	II	1761842	1762909	D	N	CB4856	1762424	1762529	1785629	1785949	23101
F36H5.4	F36H5.4	II	1761842	1762909	D	Y	JU258	1750470	1750664	1822745	1822788	72082
F36H5.5	fbxb-53	II	1758355	1760670	D	Y	JU258	1750470	1750664	1822745	1822788	72082
F36H5.8	F36H5.8	II	1748381	1751600	D	N	CB4856	1750470	1750664	1757479	1757674	6816
F36H5.8	F36H5.8	II	1748381	1751600	D	N	JU258	1750470	1750664	1822745	1822788	72082
F36H5.8	F36H5.8	II	1748381	1751600	D	N	JU322	1750423	1750664	1757479	1757674	6816
F36H5.9	F36H5.9	II	1752752	1753093	D	Y	CB4856	1750470	1750664	1757479	1757674	6816
F36H5.9	F36H5.9	II	1752752	1753093	D	Y	JU258	1750470	1750664	1822745	1822788	72082
F36H5.9	F36H5.9	II	1752752	1753093	D	Y	JU322	1750423	1750664	1757479	1757674	6816
F37B1.1	gst-24	II	13610351	13611250	A	N	MY2	13609634	13610426	13617819	13622946	7394
F37B1.2	gst-12	II	13616670	13617818	A	Y	MY2	13609634	13610426	13617819	13622946	7394
F37B1.3	gst-14	II	13614759	13616006	A	Y	MY2	13609634	13610426	13617819	13622946	7394
F37B1.4	gst-15	II	13611739	13613051	A	Y	MY2	13609634	13610426	13617819	13622946	7394
F38A1.10	clec-165	IV	1245399	1246872	D	N	JU258	1246489	1246553	1247435	1247971	883
F38A1.11	F38A1.11	IV	1247386	1249025	D	N	AB1	1247435	1247971	1248151	1248285	181
F38A1.11	F38A1.11	IV	1247386	1249025	D	N	JU258	1246489	1246553	1247435	1247971	883

F38A1.11	F38A1.11	IV	1247386	1249025	D	N	JU258	1248151	1248520	1250055	1250134	1536
F38A1.13	F38A1.13	IV	1265228	1265848	D	Y	CB4856	1264194	1264451	1266422	1266889	1972
F38A1.14	clec-169	IV	1261326	1264543	D	N	CB4856	1264194	1264451	1266422	1266889	1972
F38A1.4	clec-167	IV	1253339	1255732	D	N	CB4856	1254584	1254887	1259417	1261358	4531
F38A1.5	clec-166	IV	1249018	1251448	D	N	JU258	1248151	1248520	1250055	1250134	1536
F38A1.7	clec-168	IV	1257083	1259344	D	Y	CB4856	1254584	1254887	1259417	1261358	4531
F38B7.3	F38B7.3	V	11549279	11551525	D	N	JU258	11550127	11550329	11551245	11552425	917
F39C12.3a	tsp-14	X	4866654	4874627	D	N	RW7000	4868135	4868875	4870910	4874285	2036
F39E9.1	F39E9.1	II	3315879	3320354	D	Y	JU258	3305367	3306370	3353651	3369831	47282
F39E9.1	F39E9.1	II	3315879	3320354	D	Y	JU322	3305367	3309390	3330254	3330712	20865
F39E9.1	F39E9.1	II	3315879	3320354	D	Y	KR314	3306283	3309390	3355966	3359208	46577
F39E9.11	btb-17	II	3295410	3296578	D	N	CB4853	3289051	3295431	3300545	3305367	5115
F39E9.11	btb-17	II	3295410	3296578	D	N	CB4858	3288968	3295431	3300545	3305367	5115
F39E9.12	btb-18	II	3298391	3300672	D	N	CB4853	3289051	3295431	3300545	3305367	5115
F39E9.12	btb-18	II	3298391	3300672	D	N	CB4858	3288968	3295431	3300545	3305367	5115
F39E9.2	btb-16	II	3296904	3297980	D	Y	CB4853	3289051	3295431	3300545	3305367	5115
F39E9.2	btb-16	II	3296904	3297980	D	Y	CB4858	3288968	3295431	3300545	3305367	5115
F39E9.6	F39E9.6	II	3310283	3311925	D	Y	JU258	3305367	3306370	3353651	3369831	47282
F39E9.6	F39E9.6	II	3310283	3311925	D	Y	JU322	3305367	3309390	3330254	3330712	20865
F39E9.6	F39E9.6	II	3310283	3311925	D	Y	KR314	3306283	3309390	3355966	3359208	46577
F39E9.7	F39E9.7	II	3306027	3307113	D	N	JU258	3305367	3306370	3353651	3369831	47282
F40B1.1	bath-13	II	2520966	2522045	A	N	JU263	2521204	2521667	2546368	2547362	24702
F40D4.11	srh-102	V	17162869	17165478	D	N	MY2	17157174	17159848	17163333	17163407	3486
F40D4.12	F40D4.12	V	17159843	17162449	D	N	MY2	17157174	17159848	17163333	17163407	3486
F40E12.2	F40E12.2	II	3610403	3615346	D	N	CB4856	3611192	3611431	3612505	3612555	1075
F40F12.3	F40F12.3	III	9918541	9919964	D	N	MY2	9915171	9918783	9924106	9926330	5324
F40H7.12	F40H7.12	II	3687785	3688685	D	Y	JU258	3672184	3672299	3693900	3694195	21602
F40H7.4	srx-101	II	3689461	3690740	D	Y	JU258	3672184	3672299	3693900	3694195	21602
F40H7.5	srx-102	II	3693395	3694758	D	N	JU258	3672184	3672299	3693900	3694195	21602
F42G2.2	F42G2.2	II	2422208	2425344	D	N	KR314	2412625	2414455	2422861	2423208	8407
F42G2.2	F42G2.2	II	2422208	2425344	D	N	KR314	2424323	2424642	2424844	2425280	203
F42G2.3	F42G2.3	II	2419473	2420795	D	Y	KR314	2412625	2414455	2422861	2423208	8407
F42G2.5	F42G2.5	II	2416412	2417972	D	N	CB4856	2415510	2416359	2417871	2419440	1513
F42G2.5	F42G2.5	II	2416412	2417972	D	Y	KR314	2412625	2414455	2422861	2423208	8407
F42G2.6	F42G2.6	II	2426407	2429945	D	N	KR314	2425410	2426571	2427254	2427327	684
F42G2.7	F42G2.7	II	2418638	2419006	D	Y	KR314	2412625	2414455	2422861	2423208	8407
F42G2.8	fbxa-4	II	2414217	2415449	D	N	KR314	2412625	2414455	2422861	2423208	8407
F43C11.1	F43C11.1	II	2357096	2358072	D	N	CB4856	2356815	2357665	2374108	2375282	16444
F43C11.1	F43C11.1	II	2357096	2358072	D	N	JU322	2356815	2357665	2374108	2375282	16444
F43C11.10	F43C11.10	II	2350262	2351658	D	Y	JU258	2339835	2345193	2355695	2361160	10503
F43C11.10	F43C11.10	II	2350262	2351658	D	Y	JU263	2343688	2344994	2353289	2353399	8296
F43C11.11	F43C11.11	II	2358802	2361438	D	Y	CB4856	2356815	2357665	2374108	2375282	16444
F43C11.11	F43C11.11	II	2358802	2361438	D	Y	JU322	2356815	2357665	2374108	2375282	16444
F43C11.12	F43C11.12	II	2365670	2366064	D	Y	CB4856	2356815	2357665	2374108	2375282	16444
F43C11.12	F43C11.12	II	2365670	2366064	D	N	JU258	2365825	2366015	2397267	2398258	31253
F43C11.12	F43C11.12	II	2365670	2366064	D	N	JU263	2365966	2366015	2377842	2379591	11828
F43C11.12	F43C11.12	II	2365670	2366064	D	Y	JU322	2356815	2357665	2374108	2375282	16444
F43C11.12	F43C11.12	II	2365670	2366064	D	N	KR314	2365825	2366015	2370011	2372450	3997
F43C11.2	F43C11.2	II	2354777	2355744	D	Y	JU258	2339835	2345193	2355695	2361160	10503
F43C11.3	F43C11.3	II	2353316	2354376	D	Y	JU258	2339835	2345193	2355695	2361160	10503
F43C11.4	F43C11.4	II	2347016	2350031	D	Y	JU258	2339835	2345193	2355695	2361160	10503
F43C11.4	F43C11.4	II	2347016	2350031	D	Y	JU263	2343688	2344994	2353289	2353399	8296
F43C11.5	F43C11.5	II	2345119	2346029	D	N	JU258	2339835	2345193	2355695	2361160	10503
F43C11.5	F43C11.5	II	2345119	2346029	D	Y	JU263	2343688	2344994	2353289	2353399	8296
F43C11.7	F43C11.7	II	2335934	2337692	D	Y	JU258	2264403	2265489	2338778	2338818	73290
F43C11.7	F43C11.7	II	2335934	2337692	D	N	JU263	2334311	2335868	2337667	2337979	1800
F43C11.8	F43C11.8	II	2332521	2334339	D	Y	JU258	2264403	2265489	2338778	2338818	73290
F43C11.9	F43C11.9	II	2337913	2340747	D	N	JU258	2264403	2265489	2338778	2338818	73290
F43G6.11a	F43G6.11	II	11798953	11803841	D	N	CB4853	11799827	11802353	11803101	11804039	749
F43G6.11a	F43G6.11	II	11798953	11803841	D	N	CB4854	11799827	11802353	11803101	11804039	749

F43G6.11a	F43G6.11	II	11798953	11803841	D	N	JU322	11799827	11802353	11803101	11804039	749
F43G6.11a	F43G6.11	II	11798953	11803841	D	N	KR314	11799827	11802353	11806629	11806769	4277
F43G6.11a	F43G6.11	II	11798953	11803841	D	N	MY2	11799827	11802353	11806629	11806769	4277
F43G6.6	F43G6.6	II	11806483	11808721	D	N	KR314	11799827	11802353	11806629	11806769	4277
F43G6.6	F43G6.6	II	11806483	11808721	D	N	MY2	11799827	11802353	11806629	11806769	4277
F44E2.2a	retr-1	III	8853710	8861292	D	N	CB4854	8850388	8853742	8860468	8865128	6727
F44E2.2a	retr-1	III	8853710	8861292	D	N	CB4856	8850388	8853742	8860468	8865128	6727
F44E2.2a	retr-1	III	8853710	8861292	D	N	JU263	8850388	8853742	8859219	8859258	5478
F44E2.2a	retr-1	III	8853710	8861292	D	N	JU322	8850388	8853742	8860468	8865128	6727
F44E2.2a	retr-1	III	8853710	8861292	A	N	KR314	8850388	8853742	8860468	8865128	6727
F44E2.2a	retr-1	III	8853710	8861292	A	N	MY2	8850388	8853742	8860468	8865128	6727
F44E2.2b	retr-1	III	8853710	8861292	D	N	JU263	8850388	8853742	8859219	8859258	5478
F44E5.2	F44E5.2	II	11766688	11773955	D	N	CB4853	11765971	11766615	11769033	11771805	2419
F44E5.2	F44E5.2	II	11766688	11773955	D	N	CB4854	11765971	11766615	11769033	11771805	2419
F44E5.2	F44E5.2	II	11766688	11773955	D	N	CB4858	11765971	11766615	11769033	11771805	2419
F44E5.2	F44E5.2	II	11766688	11773955	D	N	JU322	11765971	11766615	11769033	11771805	2419
F44G3.12	fbxa-142	V	16129677	16130946	D	Y	CB4856	16129474	16129524	16131739	16131780	2216
F44G3.8	fbxa-144	V	16131157	16133256	D	N	CB4856	16129474	16129524	16131739	16131780	2216
F44G3.8	fbxa-144	V	16131157	16133256	D	N	JU258	16130987	16131231	16131536	16131582	306
F45C12.12	btb-7	II	1713261	1714230	A	N	JU322	1711111	1711157	1713678	1713841	2522
F45C12.8	fbxa-184	II	1710262	1711420	A	N	JU322	1711111	1711157	1713678	1713841	2522
F45D11.10	F45D11.10	II	988743	991080	D	N	KR314	989111	990118	990201	990304	84
F46B6.10	F46B6.10	V	9801435	9802000	D	N	RW7000	9797916	9801637	9804523	9805433	2887
F46B6.11	sru-39	V	9803448	9804579	D	N	RW7000	9797916	9801637	9804523	9805433	2887
F46F2.4	F46F2.4	X	15257518	15258932	D	N	CB4854	15246290	15250730	15257691	15257840	6962
F47B7.5	F47B7.5	X	3767780	3768802	D	N	JU258	3766106	3766679	3768392	3768543	1714
F47D2.6	srt-38	V	4269472	4271344	D	N	CB4856	4269683	4269991	4272768	4281647	2778
F47F6.5	clec-119	II	1255598	1258359	D	N	CB4856	1256565	1256643	1256767	1256834	125
F47F6.5	clec-119	II	1255598	1258359	D	N	JU322	1256565	1256643	1256767	1256834	125
F47H4.1	F47H4.1	V	17348635	17350144	D	N	CB4856	17338277	17339572	17348931	17349125	9360
F47H4.10	skr-5	V	17347890	17348677	D	Y	CB4856	17338277	17339572	17348931	17349125	9360
F47H4.11	fbxa-134	V	17350336	17352548	D	N	CB4856	17350451	17350545	17360012	17371630	9468
F47H4.11	fbxa-134	V	17350336	17352548	D	N	JU258	17350490	17350545	17360012	17371630	9468
F47H4.2	F47H4.2	V	17353293	17360180	D	N	CB4856	17350451	17350545	17360012	17371630	9468
F47H4.2	F47H4.2	V	17353293	17360180	D	N	JU258	17350490	17350545	17360012	17371630	9468
F47H4.4	fbxa-185	V	17331608	17335514	D	Y	CB4856	17328959	17329847	17337901	17338060	8055
F47H4.4	fbxa-185	V	17331608	17335514	D	Y	JU258	17229936	17238425	17347568	17348479	109144
F47H4.6	fbxa-186	V	17336350	17338344	D	N	CB4856	17328959	17329847	17337901	17338060	8055
F47H4.6	fbxa-186	V	17336350	17338344	D	Y	JU258	17229936	17238425	17347568	17348479	109144
F47H4.7	fbxa-187	V	17339049	17342750	D	N	CB4856	17338277	17339572	17348931	17349125	9360
F47H4.7	fbxa-187	V	17339049	17342750	D	Y	JU258	17229936	17238425	17347568	17348479	109144
F47H4.8	fbxa-188	V	17342904	17344870	D	Y	CB4856	17338277	17339572	17348931	17349125	9360
F47H4.8	fbxa-188	V	17342904	17344870	D	Y	JU258	17229936	17238425	17347568	17348479	109144
F47H4.9	fbxa-189	V	17345196	17347677	D	Y	CB4856	17338277	17339572	17348931	17349125	9360
F47H4.9	fbxa-189	V	17345196	17347677	D	N	JU258	17229936	17238425	17347568	17348479	109144
F48F5.1	F48F5.1	V	20435580	20439836	D	N	AB1	20436827	20436883	20437811	20437885	929
F48F5.1	F48F5.1	V	20435580	20439836	D	N	CB4853	20436827	20436883	20437811	20437885	929
F48F5.1	F48F5.1	V	20435580	20439836	D	N	CB4854	20436827	20436883	20437811	20437885	929
F48F5.1	F48F5.1	V	20435580	20439836	A	N	CB4856	20426693	20435644	20439262	20439301	3619
F48F5.1	F48F5.1	V	20435580	20439836	D	N	CB4858	20436827	20436883	20437811	20437885	929
F48F5.1	F48F5.1	V	20435580	20439836	D	N	JU258	20436827	20436883	20437811	20437885	929
F48F5.1	F48F5.1	V	20435580	20439836	D	N	JU263	20436827	20436883	20437811	20437885	929
F48F5.1	F48F5.1	V	20435580	20439836	D	N	JU322	20436827	20436883	20437811	20437885	929
F48F5.1	F48F5.1	V	20435580	20439836	D	N	KR314	20436827	20436883	20437753	20437885	871
F48G7.6	srt-17	V	623926	625023	D	N	AB1	624520	624559	624684	624728	126
F49B2.2	fbxb-67	I	14297276	14298252	D	N	JU258	14289500	14297224	14297628	14297783	405
F49C5.6	str-223	II	12564098	12565856	D	N	JU258	12546379	12564016	12565222	12565415	1207
F49F1.10	F49F1.10	IV	4138401	4139115	D	Y	AB1	4123592	4124569	4166385	4168062	41817
F49F1.10	F49F1.10	IV	4138401	4139115	D	Y	CB4854	4123592	4124569	4166385	4168062	41817
F49F1.10	F49F1.10	IV	4138401	4139115	D	Y	CB4856	4123592	4124569	4166385	4168062	41817

F49F1.10	F49F1.10	IV	4138401	4139115	D	Y	JU258	4123592	4124569	4166385	4168062	41817
F49F1.10	F49F1.10	IV	4138401	4139115	D	Y	JU322	4123592	4124569	4166385	4168062	41817
F49F1.10	F49F1.10	IV	4138401	4139115	D	Y	MY2	4123592	4124569	4166385	4168062	41817
F49F1.11	F49F1.11	IV	4139678	4141400	D	Y	AB1	4123592	4124569	4166385	4168062	41817
F49F1.11	F49F1.11	IV	4139678	4141400	D	Y	CB4854	4123592	4124569	4166385	4168062	41817
F49F1.11	F49F1.11	IV	4139678	4141400	D	Y	CB4856	4123592	4124569	4166385	4168062	41817
F49F1.11	F49F1.11	IV	4139678	4141400	D	Y	JU258	4123592	4124569	4166385	4168062	41817
F49F1.11	F49F1.11	IV	4139678	4141400	D	Y	JU322	4123592	4124569	4166385	4168062	41817
F49F1.11	F49F1.11	IV	4139678	4141400	D	Y	MY2	4123592	4124569	4166385	4168062	41817
F49F1.12	F49F1.12	IV	4141736	4143012	D	Y	AB1	4123592	4124569	4166385	4168062	41817
F49F1.12	F49F1.12	IV	4141736	4143012	D	Y	CB4854	4123592	4124569	4166385	4168062	41817
F49F1.12	F49F1.12	IV	4141736	4143012	D	Y	CB4856	4123592	4124569	4166385	4168062	41817
F49F1.12	F49F1.12	IV	4141736	4143012	D	Y	JU258	4123592	4124569	4166385	4168062	41817
F49F1.12	F49F1.12	IV	4141736	4143012	D	Y	JU322	4123592	4124569	4166385	4168062	41817
F49F1.12	F49F1.12	IV	4141736	4143012	D	Y	MY2	4123592	4124569	4166385	4168062	41817
F49F1.7	F49F1.7	IV	4123521	4125750	D	N	AB1	4123592	4124569	4166385	4168062	41817
F49F1.7	F49F1.7	IV	4123521	4125750	D	N	CB4854	4123592	4124569	4166385	4168062	41817
F49F1.7	F49F1.7	IV	4123521	4125750	D	N	CB4856	4123592	4124569	4166385	4168062	41817
F49F1.7	F49F1.7	IV	4123521	4125750	D	N	JU258	4123592	4124569	4166385	4168062	41817
F49F1.7	F49F1.7	IV	4123521	4125750	D	N	JU322	4123592	4124569	4166385	4168062	41817
F49F1.7	F49F1.7	IV	4123521	4125750	D	N	MY2	4123592	4124569	4166385	4168062	41817
F49F1.8	F49F1.8	IV	4130496	4134624	D	Y	AB1	4123592	4124569	4166385	4168062	41817
F49F1.8	F49F1.8	IV	4130496	4134624	D	Y	CB4854	4123592	4124569	4166385	4168062	41817
F49F1.8	F49F1.8	IV	4130496	4134624	D	Y	CB4856	4123592	4124569	4166385	4168062	41817
F49F1.8	F49F1.8	IV	4130496	4134624	D	Y	JU258	4123592	4124569	4166385	4168062	41817
F49F1.8	F49F1.8	IV	4130496	4134624	D	Y	JU322	4123592	4124569	4166385	4168062	41817
F49F1.8	F49F1.8	IV	4130496	4134624	D	Y	MY2	4123592	4124569	4166385	4168062	41817
F49F1.9	F49F1.9	IV	4136841	4137560	D	Y	AB1	4123592	4124569	4166385	4168062	41817
F49F1.9	F49F1.9	IV	4136841	4137560	D	Y	CB4854	4123592	4124569	4166385	4168062	41817
F49F1.9	F49F1.9	IV	4136841	4137560	D	Y	CB4856	4123592	4124569	4166385	4168062	41817
F49F1.9	F49F1.9	IV	4136841	4137560	D	Y	JU258	4123592	4124569	4166385	4168062	41817
F49F1.9	F49F1.9	IV	4136841	4137560	D	Y	JU322	4123592	4124569	4166385	4168062	41817
F49F1.9	F49F1.9	IV	4136841	4137560	D	Y	MY2	4123592	4124569	4166385	4168062	41817
F49H6.11	srz-94	V	17033187	17035919	D	Y	JU258	17030051	17030090	17040742	17041452	10653
F52C6.10	bath-7	II	1920018	1921106	D	Y	CB4853	1823064	1825029	1934411	1934453	109383
F52C6.10	bath-7	II	1920018	1921106	D	N	CB4856	1914552	1916627	1920897	1921622	4271
F52C6.10	bath-7	II	1920018	1921106	D	Y	CB4858	1823064	1825029	1934411	1937196	109383
F52C6.10	bath-7	II	1920018	1921106	D	Y	JU258	1860810	1862541	1934453	1937551	71913
F52C6.10	bath-7	II	1920018	1921106	D	Y	KR314	1823064	1825029	1934453	1937196	109425
F52C6.11	bath-2	II	1921619	1922746	D	Y	CB4853	1823064	1825029	1934411	1934453	109383
F52C6.11	bath-2	II	1921619	1922746	D	N	CB4856	1922456	1922525	1934453	1937196	11929
F52C6.11	bath-2	II	1921619	1922746	D	Y	CB4858	1823064	1825029	1934411	1937196	109383
F52C6.11	bath-2	II	1921619	1922746	D	Y	JU258	1860810	1862541	1934453	1937551	71913
F52C6.11	bath-2	II	1921619	1922746	D	Y	KR314	1823064	1825029	1934453	1937196	109425
F52C6.12	F52C6.12	II	1929367	1929954	D	Y	CB4853	1823064	1825029	1934411	1934453	109383
F52C6.12	F52C6.12	II	1929367	1929954	D	Y	CB4856	1922456	1922525	1934453	1937196	11929
F52C6.12	F52C6.12	II	1929367	1929954	D	Y	CB4858	1823064	1825029	1934411	1937196	109383
F52C6.12	F52C6.12	II	1929367	1929954	D	Y	JU258	1860810	1862541	1934453	1937551	71913
F52C6.12	F52C6.12	II	1929367	1929954	D	Y	KR314	1823064	1825029	1934453	1937196	109425
F52C6.13	F52C6.13	II	1930191	1930748	D	Y	CB4853	1823064	1825029	1934411	1934453	109383
F52C6.13	F52C6.13	II	1930191	1930748	D	Y	CB4856	1922456	1922525	1934453	1937196	11929
F52C6.13	F52C6.13	II	1930191	1930748	D	Y	CB4858	1823064	1825029	1934411	1937196	109383
F52C6.13	F52C6.13	II	1930191	1930748	D	Y	JU258	1860810	1862541	1934453	1937551	71913
F52C6.13	F52C6.13	II	1930191	1930748	D	Y	KR314	1823064	1825029	1934453	1937196	109425
F52C6.14	F52C6.14	II	1931146	1934508	D	N	CB4853	1823064	1825029	1934411	1934453	109383
F52C6.14	F52C6.14	II	1931146	1934508	D	N	CB4856	1922456	1922525	1934453	1937196	11929
F52C6.14	F52C6.14	II	1931146	1934508	D	N	CB4858	1823064	1825029	1934411	1937196	109383
F52C6.14	F52C6.14	II	1931146	1934508	D	N	JU258	1860810	1862541	1934453	1937551	71913
F52C6.14	F52C6.14	II	1931146	1934508	D	N	JU322	1915879	1931151	1934411	1937323	3261
F52C6.14	F52C6.14	II	1931146	1934508	D	N	KR314	1823064	1825029	1934453	1937196	109425

F52C6.2	F52C6.2	II	1926472	1927249	D	Y	CB4853	1823064	1825029	1934411	1934453	109383
F52C6.2	F52C6.2	II	1926472	1927249	D	Y	CB4856	1922456	1922525	1934453	1937196	11929
F52C6.2	F52C6.2	II	1926472	1927249	D	Y	CB4858	1823064	1825029	1934411	1937196	109383
F52C6.2	F52C6.2	II	1926472	1927249	D	Y	JU258	1860810	1862541	1934453	1937551	71913
F52C6.2	F52C6.2	II	1926472	1927249	D	Y	KR314	1823064	1825029	1934453	1937196	109425
F52C6.3	F52C6.3	II	1924769	1925698	D	Y	CB4853	1823064	1825029	1934411	1934453	109383
F52C6.3	F52C6.3	II	1924769	1925698	D	Y	CB4856	1922456	1922525	1934453	1937196	11929
F52C6.3	F52C6.3	II	1924769	1925698	D	Y	CB4858	1823064	1825029	1934411	1937196	109383
F52C6.3	F52C6.3	II	1924769	1925698	D	Y	JU258	1860810	1862541	1934453	1937551	71913
F52C6.3	F52C6.3	II	1924769	1925698	D	Y	KR314	1823064	1825029	1934453	1937196	109425
F52C6.4	F52C6.4	II	1922868	1924259	D	Y	CB4853	1823064	1825029	1934411	1934453	109383
F52C6.4	F52C6.4	II	1922868	1924259	D	Y	CB4856	1922456	1922525	1934453	1937196	11929
F52C6.4	F52C6.4	II	1922868	1924259	D	Y	CB4858	1823064	1825029	1934411	1937196	109383
F52C6.4	F52C6.4	II	1922868	1924259	D	Y	JU258	1860810	1862541	1934453	1937551	71913
F52C6.4	F52C6.4	II	1922868	1924259	D	Y	KR314	1823064	1825029	1934453	1937196	109425
F52C6.5	math-30	II	1908163	1909737	D	Y	CB4853	1823064	1825029	1934411	1934453	109383
F52C6.5	math-30	II	1908163	1909737	D	Y	CB4856	1845546	1846078	1912838	1913611	66761
F52C6.5	math-30	II	1908163	1909737	D	Y	CB4858	1823064	1825029	1934411	1937196	109383
F52C6.5	math-30	II	1908163	1909737	D	Y	JU258	1860810	1862541	1934453	1937551	71913
F52C6.5	math-30	II	1908163	1909737	D	Y	KR314	1823064	1825029	1934453	1937196	109425
F52C6.5	math-30	II	1908163	1909737	D	N	MY2	1894685	1895971	1909021	1910290	13051
F52C6.6	math-31	II	1912472	1912837	D	Y	CB4853	1823064	1825029	1934411	1934453	109383
F52C6.6	math-31	II	1912472	1912837	D	Y	CB4856	1845546	1846078	1912838	1913611	66761
F52C6.6	math-31	II	1912472	1912837	D	Y	CB4858	1823064	1825029	1934411	1937196	109383
F52C6.6	math-31	II	1912472	1912837	D	Y	JU258	1860810	1862541	1934453	1937551	71913
F52C6.6	math-31	II	1912472	1912837	D	Y	KR314	1823064	1825029	1934453	1937196	109425
F52C6.8	bath-4	II	1916800	1917954	D	Y	CB4853	1823064	1825029	1934411	1934453	109383
F52C6.8	bath-4	II	1916800	1917954	D	Y	CB4856	1914552	1916627	1920897	1921622	4271
F52C6.8	bath-4	II	1916800	1917954	D	Y	CB4858	1823064	1825029	1934411	1937196	109383
F52C6.8	bath-4	II	1916800	1917954	D	Y	JU258	1860810	1862541	1934453	1937551	71913
F52C6.8	bath-4	II	1916800	1917954	D	Y	KR314	1823064	1825029	1934453	1937196	109425
F53C3.7	F53C3.7	II	3894834	3896232	D	N	AB1	3892164	3894857	3895590	3895653	734
F53C3.7	F53C3.7	II	3894834	3896232	D	N	CB4856	3895694	3895848	3896002	3896154	155
F53C3.7	F53C3.7	II	3894834	3896232	D	N	JU258	3892164	3894857	3895590	3895694	734
F53C3.7	F53C3.7	II	3894834	3896232	D	N	MY2	3892164	3894857	3895590	3895848	734
F53E10.5	F53E10.5	V	2596019	2598929	D	Y	JU263	2594164	2595371	2600395	2602332	5025
F53G2.2	F53G2.2	II	2481939	2485903	D	N	KR314	2483730	2483769	2485904	2488656	2136
F53G2.2	F53G2.2	II	2481939	2485903	D	N	MY2	2480529	2480964	2482497	2482827	1534
F53G2.3	F53G2.3	II	2480400	2481313	D	N	MY2	2480529	2480964	2482497	2482827	1534
F53G2.8	F53G2.8	II	2483408	2483870	D	N	KR314	2483730	2483769	2485904	2488656	2136
F53H1.1	F53H1.1	IV	1297258	1309644	A	N	JU322	1293374	1297648	1297834	1299000	187
F53H4.6	F53H4.6	X	15852856	15860522	D	N	AB1	15853145	15853252	15853587	15853730	336
F54A3.1	F54A3.1	II	2249408	2253961	D	N	KR314	2246518	2249981	2298370	2298409	48390
F54C1.1	F54C1.1	I	5008789	5013374	D	N	RW7000	5012231	5012340	5013315	5014361	976
F54D10.1	skr-15	II	3824140	3824773	D	N	MY2	3821205	3824228	3824419	3824528	192
F54D10.7	F54D10.7	II	3820145	3821278	D	N	AB1	3820245	3820396	3821205	3824183	810
F54D10.7	F54D10.7	II	3820145	3821278	D	N	CB4856	3818398	3820396	3821205	3824183	810
F54D10.7	F54D10.7	II	3820145	3821278	D	N	JU258	3820245	3820324	3821205	3824183	882
F54D10.7	F54D10.7	II	3820145	3821278	D	N	JU263	3820245	3820396	3821205	3824183	810
F54D10.8	F54D10.8	II	3825706	3827872	D	N	JU322	3824633	3825717	3826934	3827258	1218
F54E2.1	F54E2.1	V	2810426	2812000	D	N	JU258	2801027	2810838	2811036	2811122	199
F54E2.1	F54E2.1	V	2810426	2812000	D	N	JU258	2811563	2811604	2820099	2820229	8496
F54E2.5	F54E2.5	V	2812530	2815931	D	Y	JU258	2811563	2811604	2820099	2820229	8496
F54E2.6	srt-34	V	2816882	2818216	D	Y	JU258	2811563	2811604	2820099	2820229	8496
F54F11.2	F54F11.2	II	13507727	13516387	A	N	CB4853	13513407	13513515	13526754	13526822	13240
F54F11.2	F54F11.2	II	13507727	13516387	A	N	CB4858	13513407	13513449	13526754	13527016	13306
F54F11.3	sre-45	II	13517820	13522833	A	Y	CB4853	13513407	13513515	13526754	13526822	13240
F54F11.3	sre-45	II	13517820	13522833	A	Y	CB4858	13513407	13513449	13526754	13527016	13306
F55B11.5	F55B11.5	IV	14427661	14428397	D	N	JU258	14424954	14427724	14428073	14428748	350
F55G11.1	F55G11.1	IV	12945001	12946546	D	Y	RW7000	12881617	12881730	12968210	12968283	86481

F55G11.10	F55G11.10	IV	12948739	12950226	D	Y	RW7000	12881617	12881730	12968210	12968283	86481
F55G11.2	F55G11.2	IV	12967418	12969258	D	N	RW7000	12881617	12881730	12968210	12968283	86481
F55G11.5	dod-22	IV	12965356	12967287	D	Y	RW7000	12881617	12881730	12968210	12968283	86481
F55G11.6	F55G11.6	IV	12957999	12959563	D	Y	RW7000	12881617	12881730	12968210	12968283	86481
F55G11.7	F55G11.7	IV	12960492	12962089	D	Y	RW7000	12881617	12881730	12968210	12968283	86481
F55G11.8	F55G11.8	IV	12962777	12964592	D	Y	RW7000	12881617	12881730	12968210	12968283	86481
F55G11.9	abt-3	IV	12950286	12957097	D	Y	RW7000	12881617	12881730	12968210	12968283	86481
F56B3.9	F56B3.9	IV	771603	772797	D	N	JU263	768408	772183	772715	773088	533
F56B6.5a	uvt-6	X	3528800	3531814	D	N	RW7000	3515754	3528806	3531106	3531550	2301
F56C3.5	F56C3.5	X	1362812	1363993	D	N	JU258	1362033	1362883	1363724	1363804	842
F56G4.6	F56G4.6	I	11364345	11374024	D	N	MY2	11364460	11364959	11370480	11372027	5522
F57A10.1	str-9	V	15758748	15760391	D	Y	JU258	15730588	15730913	15760733	15761000	29821
F57A10.3	haf-3	V	15760419	15766150	D	N	JU258	15730588	15730913	15760733	15761000	29821
F57A10.6	nhr-283	V	15774920	15776615	D	N	JU258	15774953	15774995	15775112	15775613	118
F57C7.2a	nhx-5	X	10576890	10580984	A	N	AB1	10575095	10577059	10583794	10585063	6736
F57C7.4	F57C7.4	X	10580992	10589468	A	N	AB1	10575095	10577059	10583794	10585063	6736
F57E7.1	F57E7.1	V	16481578	16481972	D	Y	CB4856	16479069	16481528	16484015	16484949	2488
F57E7.2	F57E7.2	V	16482392	16483568	D	Y	CB4856	16479069	16481528	16484015	16484949	2488
F57E7.3	srh-139	V	16483953	16485354	D	N	CB4856	16479069	16481528	16484015	16484949	2488
F57F4.4	F57F4.4	V	6401352	6408767	D	N	RW7000	6401901	6407581	6408264	6408860	684
F57G4.5	F57G4.5	V	17641904	17642814	D	N	CB4856	17640926	17641964	17644684	17649136	2721
F57G4.5	F57G4.5	V	17641904	17642814	D	Y	JU258	17639843	17640926	17658110	17658389	17185
F57G4.6	F57G4.6	V	17642973	17643876	D	Y	CB4856	17640926	17641964	17644684	17649136	2721
F57G4.6	F57G4.6	V	17642973	17643876	D	Y	JU258	17639843	17640926	17658110	17658389	17185
F57G4.8	fbxa-192	V	17640695	17650489	D	N	CB4856	17640926	17641964	17644684	17649136	2721
F57G4.8	fbxa-192	V	17640695	17650489	D	N	JU258	17639843	17640926	17658110	17658389	17185
F57G4.9	F57G4.9	V	17643850	17644733	D	Y	CB4856	17640926	17641964	17644684	17649136	2721
F57G4.9	F57G4.9	V	17643850	17644733	D	Y	JU258	17639843	17640926	17658110	17658389	17185
F57G9.4	sre-29	II	12399314	12401806	D	N	CB4858	12400679	12401389	12401743	12404875	355
F57G9.4	sre-29	II	12399314	12401806	D	N	JU322	12400679	12401389	12401743	12404875	355
F58E1.10	F58E1.10	II	1689699	1691080	D	Y	JU258	1686130	1686928	1695276	1698050	8349
F58E1.11	F58E1.11	II	1691560	1692908	D	Y	JU258	1686130	1686928	1695276	1698050	8349
F58E1.12	F58E1.12	II	1693299	1694618	D	N	CB4856	1693760	1693815	1695276	1697848	1462
F58E1.12	F58E1.12	II	1693299	1694618	D	Y	JU258	1686130	1686928	1695276	1698050	8349
F58E1.13	F58E1.13	II	1694857	1695830	D	N	CB4856	1693760	1693815	1695276	1697848	1462
F58E1.13	F58E1.13	II	1694857	1695830	D	N	JU258	1686130	1686928	1695276	1698050	8349
F58E1.8	fbxb-18	II	1686080	1687793	D	N	JU258	1686130	1686928	1695276	1698050	8349
F58E1.9	fbxb-19	II	1688238	1689175	D	Y	JU258	1686130	1686928	1695276	1698050	8349
F58F12.1	F58F12.1	II	6383594	6384641	A	N	JU263	6373804	6383554	6384414	6387426	861
F59A1.12	F59A1.12	V	17664725	17667743	D	N	CB4856	17664070	17664910	17666513	17666869	1604
F59A1.13	F59A1.13	V	17687230	17690915	D	N	JU263	17685977	17687230	17690042	17692292	2813
F59A1.7	fbxa-108	V	17656918	17658130	D	N	JU258	17639843	17640926	17658110	17658389	17185
F59A1.8	fbxa-129	V	17655548	17656621	D	Y	CB4856	17650071	17653548	17656683	17657164	3136
F59A1.8	fbxa-129	V	17655548	17656621	D	Y	JU258	17639843	17640926	17658110	17658389	17185
F59A1.9	fbxa-193	V	17653449	17655248	D	N	CB4856	17650071	17653548	17656683	17657164	3136
F59A1.9	fbxa-193	V	17653449	17655248	D	Y	JU258	17639843	17640926	17658110	17658389	17185
F59A7.11	F59A7.11	V	2013418	2013860	D	Y	CB4854	2006917	2008669	2045822	2049093	37154
F59A7.12	F59A7.12	V	2030177	2030407	D	Y	CB4854	2006917	2008669	2045822	2049093	37154
F59A7.2	F59A7.2	V	2015838	2016538	D	Y	CB4854	2006917	2008669	2045822	2049093	37154
F59A7.3	srab-13	V	2024703	2027887	D	Y	CB4854	2006917	2008669	2045822	2049093	37154
F59A7.4	hil-6	V	2030657	2031475	D	Y	CB4854	2006917	2008669	2045822	2049093	37154
F59A7.4	hil-6	V	2030657	2031475	D	N	CB4856	2027804	2030835	2035594	2038195	4760
F59A7.4	hil-6	V	2030657	2031475	D	N	JU263	2027804	2030835	2035594	2038195	4760
F59A7.4	hil-6	V	2030657	2031475	D	N	MY2	2027804	2030835	2035594	2038195	4760
F59A7.5a	F59A7.5	V	2022612	2024661	D	Y	CB4854	2006917	2008669	2045822	2049093	37154
F59A7.7	F59A7.7	V	2012206	2013333	D	Y	CB4854	2006917	2008669	2045822	2049093	37154
F59A7.8	F59A7.8	V	2008690	2011526	D	Y	CB4854	2006917	2008669	2045822	2049093	37154
F59A7.8	F59A7.8	V	2008690	2011526	D	Y	CB4856	2005570	2006917	2011818	2013518	4902
F59A7.8	F59A7.8	V	2008690	2011526	D	N	JU258	2009255	2009484	2011818	2013518	2335
F59A7.8	F59A7.8	V	2008690	2011526	D	Y	JU263	2005570	2006917	2011818	2013518	4902

F59A7.8	F59A7.8	V	2008690	2011526	D	Y	MY2	2005570	2006917	2011818	2013518	4902
F59E12.6a	F59E12.6	II	5626281	5629675	D	N	AB1	5621467	5624981	5629367	5633406	4387
F59E12.6a	F59E12.6	II	5626281	5629675	D	N	CB4856	5624981	5627241	5629367	5633406	2127
F59E12.6a	F59E12.6	II	5626281	5629675	D	N	MY2	5621467	5624981	5629367	5633406	4387
F59F4.2	F59F4.2	X	15841493	15842293	D	N	JU258	15841561	15841895	15842116	15842571	222
F59H6.1	bath-19	II	2035788	2039387	D	Y	CB4853	2028436	2028592	2040432	2040665	11841
F59H6.1	bath-19	II	2035788	2039387	D	Y	CB4856	2033514	2034216	2052296	2052336	18081
F59H6.1	bath-19	II	2035788	2039387	D	Y	CB4858	2028545	2030102	2040432	2040665	10331
F59H6.1	bath-19	II	2035788	2039387	D	N	JU258	2030893	2031287	2039156	2040432	7870
F59H6.1	bath-19	II	2035788	2039387	D	N	JU322	2036215	2038081	2041183	2041287	3103
F59H6.1	bath-19	II	2035788	2039387	D	Y	KR314	2028592	2029119	2040432	2040665	11314
F59H6.10	bath-3	II	2029326	2030464	D	Y	CB4853	2028436	2028592	2040432	2040665	11841
F59H6.10	bath-3	II	2029326	2030464	D	Y	CB4856	2026211	2026291	2032761	2032962	6471
F59H6.10	bath-3	II	2029326	2030464	D	N	CB4858	2028545	2030102	2040432	2040665	10331
F59H6.10	bath-3	II	2029326	2030464	D	N	JU322	2029119	2030102	2031974	2032572	1873
F59H6.10	bath-3	II	2029326	2030464	D	Y	KR314	2028592	2029119	2040432	2040665	11314
F59H6.11	bath-5	II	2030840	2031939	D	Y	CB4853	2028436	2028592	2040432	2040665	11841
F59H6.11	bath-5	II	2030840	2031939	D	Y	CB4856	2026211	2026291	2032761	2032962	6471
F59H6.11	bath-5	II	2030840	2031939	D	Y	CB4858	2028545	2030102	2040432	2040665	10331
F59H6.11	bath-5	II	2030840	2031939	D	N	JU258	2030893	2031287	2039156	2040432	7870
F59H6.11	bath-5	II	2030840	2031939	D	Y	JU322	2029119	2030102	2031974	2032572	1873
F59H6.11	bath-5	II	2030840	2031939	D	Y	KR314	2028592	2029119	2040432	2040665	11314
F59H6.12	btb-4	II	2032494	2033218	D	Y	CB4853	2028436	2028592	2040432	2040665	11841
F59H6.12	btb-4	II	2032494	2033218	D	N	CB4856	2026211	2026291	2032761	2032962	6471
F59H6.12	btb-4	II	2032494	2033218	D	Y	CB4858	2028545	2030102	2040432	2040665	10331
F59H6.12	btb-4	II	2032494	2033218	D	Y	JU258	2030893	2031287	2039156	2040432	7870
F59H6.12	btb-4	II	2032494	2033218	D	Y	KR314	2028592	2029119	2040432	2040665	11314
F59H6.2	F59H6.2	II	2019655	2022442	D	Y	CB4853	2013182	2019648	2026507	2027531	6860
F59H6.2	F59H6.2	II	2019655	2022442	D	Y	CB4856	1995283	1995557	2022712	2025902	27156
F59H6.2	F59H6.2	II	2019655	2022442	D	Y	CB4858	2013182	2019648	2026507	2027531	6860
F59H6.2	F59H6.2	II	2019655	2022442	D	Y	JU258	1997733	1999695	2026507	2027864	26813
F59H6.2	F59H6.2	II	2019655	2022442	D	Y	KR314	2013182	2019648	2026507	2027531	6860
F59H6.3	F59H6.3	II	2018022	2018861	D	Y	CB4856	1995283	1995557	2022712	2025902	27156
F59H6.3	F59H6.3	II	2018022	2018861	D	Y	JU258	1997733	1999695	2026507	2027864	26813
F59H6.4	math-32	II	2010651	2013387	D	N	CB4853	1959059	1999941	2012475	2013142	12535
F59H6.4	math-32	II	2010651	2013387	D	Y	CB4856	1995283	1995557	2022712	2025902	27156
F59H6.4	math-32	II	2010651	2013387	D	N	CB4858	1997733	1999941	2012475	2013103	12535
F59H6.4	math-32	II	2010651	2013387	D	Y	JU258	1997733	1999695	2026507	2027864	26813
F59H6.4	math-32	II	2010651	2013387	D	N	KR314	1997733	1999695	2012475	2013142	12781
F59H6.5	F59H6.5	II	2003378	2008251	D	Y	CB4853	1959059	1999941	2012475	2013142	12535
F59H6.5	F59H6.5	II	2003378	2008251	D	Y	CB4856	1995283	1995557	2022712	2025902	27156
F59H6.5	F59H6.5	II	2003378	2008251	D	Y	CB4858	1997733	1999941	2012475	2013103	12535
F59H6.5	F59H6.5	II	2003378	2008251	D	Y	JU258	1997733	1999695	2026507	2027864	26813
F59H6.5	F59H6.5	II	2003378	2008251	D	Y	KR314	1997733	1999695	2012475	2013142	12781
F59H6.6	F59H6.6	II	2011488	2012473	D	Y	CB4853	1959059	1999941	2012475	2013142	12535
F59H6.6	F59H6.6	II	2011488	2012473	D	Y	CB4856	1995283	1995557	2022712	2025902	27156
F59H6.6	F59H6.6	II	2011488	2012473	D	Y	CB4858	1997733	1999941	2012475	2013103	12535
F59H6.6	F59H6.6	II	2011488	2012473	D	Y	JU258	1997733	1999695	2026507	2027864	26813
F59H6.6	F59H6.6	II	2011488	2012473	D	Y	KR314	1997733	1999695	2012475	2013142	12781
F59H6.7	cya-2	II	2022879	2023303	D	Y	CB4853	2013182	2019648	2026507	2027531	6860
F59H6.7	cya-2	II	2022879	2023303	D	Y	CB4858	2013182	2019648	2026507	2027531	6860
F59H6.7	cya-2	II	2022879	2023303	D	Y	JU258	1997733	1999695	2026507	2027864	26813
F59H6.7	cya-2	II	2022879	2023303	D	Y	KR314	2013182	2019648	2026507	2027531	6860
F59H6.8	bath-21	II	2025643	2026611	D	N	CB4853	2013182	2019648	2026507	2027531	6860
F59H6.8	bath-21	II	2025643	2026611	D	N	CB4856	2026211	2026291	2032761	2032962	6471
F59H6.8	bath-21	II	2025643	2026611	D	N	CB4858	2013182	2019648	2026507	2027531	6860
F59H6.8	bath-21	II	2025643	2026611	D	N	JU258	1997733	1999695	2026507	2027864	26813
F59H6.8	bath-21	II	2025643	2026611	D	N	KR314	2013182	2019648	2026507	2027531	6860
F59H6.9	bath-1	II	2027625	2028813	D	N	CB4853	2028436	2028592	2040432	2040665	11841
F59H6.9	bath-1	II	2027625	2028813	D	Y	CB4856	2026211	2026291	2032761	2032962	6471

H02K04.1	clec-229	V	15340253	15341273	A	Y	RW7000	15282709	15282820	15399718	15400303	116899
H02K04.2	clec-228	V	15338545	15339377	A	Y	RW7000	15282709	15282820	15399718	15400303	116899
H04J21.1	H04J21.1	III	2366872	2372643	D	N	CB4853	2369706	2369745	2371697	2371829	1953
H04J21.1	H04J21.1	III	2366872	2372643	D	N	CB4854	2369291	2369996	2371697	2371829	1702
H04J21.1	H04J21.1	III	2366872	2372643	D	N	CB4858	2369706	2370066	2371697	2371829	1632
H04J21.1	H04J21.1	III	2366872	2372643	D	N	JU258	2369417	2369667	2371697	2371829	2031
H04J21.1	H04J21.1	III	2366872	2372643	D	N	JU322	2369417	2370066	2371697	2371829	1632
H04J21.1	H04J21.1	III	2366872	2372643	D	N	KR314	2369667	2369745	2371697	2371829	1953
H05B21.2	srh-248	V	2954403	2955441	D	Y	JU258	2934122	2936892	2956596	2958453	19705
H05B21.2	srh-248	V	2954403	2955441	D	N	MY2	2934122	2936892	2955369	2956596	18478
H11L12.1	H11L12.1	X	17710399	17711587	D	Y	CB4854	17701285	17702834	17718444	NA	15611
H16D19.1	clec-13	I	12635161	12636531	D	Y	JU258	12627134	12633945	12642554	12643233	8610
H16D19.1	clec-13	I	12635161	12636531	D	Y	JU263	12633798	12633945	12642554	12642930	8610
H16D19.1	clec-13	I	12635161	12636531	D	Y	KR314	12633798	12633945	12642554	12642742	8610
H16D19.3	H16D19.3	I	12638719	12640474	D	Y	JU258	12627134	12633945	12642554	12643233	8610
H16D19.3	H16D19.3	I	12638719	12640474	D	Y	JU263	12633798	12633945	12642554	12642930	8610
H16D19.3	H16D19.3	I	12638719	12640474	D	Y	KR314	12633798	12633945	12642554	12642742	8610
H16D19.4	H16D19.4	I	12640727	12644416	D	N	CB4856	12643293	12643842	12644032	12644367	191
H16D19.4	H16D19.4	I	12640727	12644416	D	N	JU258	12627134	12633945	12642554	12643233	8610
H16D19.4	H16D19.4	I	12640727	12644416	D	N	JU258	12643293	12643842	12644032	12644367	191
H16D19.4	H16D19.4	I	12640727	12644416	D	N	JU263	12633798	12633945	12642554	12642930	8610
H16D19.4	H16D19.4	I	12640727	12644416	D	N	JU263	12643293	12643842	12644032	12644367	191
H16D19.4	H16D19.4	I	12640727	12644416	D	N	KR314	12633798	12633945	12642554	12642742	8610
H16D19.4	H16D19.4	I	12640727	12644416	D	N	KR314	12643293	12643842	12644032	12644367	191
H25P06.4	H25P06.4	I	11305812	11309592	D	N	KR314	11297160	11299293	11307482	11311827	8190
H27D07.2	srw-141	V	2937310	2938596	D	Y	JU258	2934122	2936892	2956596	2958453	19705
H27D07.2	srw-141	V	2937310	2938596	D	Y	MY2	2934122	2936892	2955369	2956596	18478
H27D07.3	srw-143	V	2939602	2941504	D	Y	JU258	2934122	2936892	2956596	2958453	19705
H27D07.3	srw-143	V	2939602	2941504	D	Y	MY2	2934122	2936892	2955369	2956596	18478
H27D07.4	srw-137	V	2942570	2943958	D	Y	JU258	2934122	2936892	2956596	2958453	19705
H27D07.4	srw-137	V	2942570	2943958	D	Y	MY2	2934122	2936892	2955369	2956596	18478
H27D07.5	srw-122	V	2950711	2952201	D	Y	JU258	2934122	2936892	2956596	2958453	19705
H27D07.5	srw-122	V	2950711	2952201	D	Y	MY2	2934122	2936892	2955369	2956596	18478
H27D07.6	srh-87	V	2944378	2945681	D	Y	JU258	2934122	2936892	2956596	2958453	19705
H27D07.6	srh-87	V	2944378	2945681	D	Y	MY2	2934122	2936892	2955369	2956596	18478
K01A12.3	K01A12.3	X	8620510	8625129	D	N	JU263	8623388	8623804	8623888	8624010	85
K02E7.10	K02E7.10	II	1065572	1067004	D	Y	CB4856	1058490	1058886	1071402	1074143	12517
K02E7.10	K02E7.10	II	1065572	1067004	D	Y	KR314	1050030	1056548	1072536	1074041	15989
K02E7.12	K02E7.12	II	1070646	1071496	D	N	CB4856	1058490	1058886	1071402	1074143	12517
K02E7.12	K02E7.12	II	1070646	1071496	D	Y	KR314	1050030	1056548	1072536	1074041	15989
K02E7.4	K02E7.4	II	1072412	1074733	D	N	KR314	1050030	1056548	1072536	1074041	15989
K02E7.6	K02E7.6	II	1057189	1059286	D	N	CB4856	1058490	1058886	1071402	1074143	12517
K02E7.6	K02E7.6	II	1057189	1059286	D	Y	KR314	1050030	1056548	1072536	1074041	15989
K02E7.9	btb-10	II	1059719	1060808	D	Y	CB4856	1058490	1058886	1071402	1074143	12517
K02E7.9	btb-10	II	1059719	1060808	D	Y	KR314	1050030	1056548	1072536	1074041	15989
K02F6.2	K02F6.2	II	2552200	2553506	D	N	CB4853	2549621	2552229	2553573	2557860	1345
K02F6.2	K02F6.2	II	2552200	2553506	D	N	CB4858	2549365	2552229	2553573	2557860	1345
K02F6.2	K02F6.2	II	2552200	2553506	D	N	KR314	2549621	2552229	2553573	2557860	1345
K02F6.3	K02F6.3	II	2546290	2549515	A	N	JU263	2521204	2521667	2546368	2547362	24702
K02F6.4	K02F6.4	II	2538495	2543921	D	N	CB4853	2532529	2539799	2540924	2541722	1126
K02F6.4	K02F6.4	II	2538495	2543921	D	N	CB4858	2538544	2539707	2540924	2542892	1218
K02F6.4	K02F6.4	II	2538495	2543921	A	Y	JU263	2521204	2521667	2546368	2547362	24702
K02F6.4	K02F6.4	II	2538495	2543921	D	N	KR314	2538544	2539707	2540924	2541722	1218
K02F6.5	K02F6.5	II	2535418	2536251	A	Y	JU263	2521204	2521667	2546368	2547362	24702
K02F6.6	K02F6.6	II	2529293	2531477	A	Y	JU263	2521204	2521667	2546368	2547362	24702
K02F6.7	K02F6.7	II	2525235	2529245	A	Y	JU263	2521204	2521667	2546368	2547362	24702
K02F6.8	K02F6.8	II	2533446	2534279	A	Y	JU263	2521204	2521667	2546368	2547362	24702
K03D3.1	srz-74	IV	16329419	16331019	D	Y	CB4856	16280625	16286565	16345044	16347072	58480
K03D3.10	rac-2	IV	16308185	16309789	D	Y	CB4856	16280625	16286565	16345044	16347072	58480
K03D3.11	srz-104	IV	16324002	16324933	D	Y	CB4856	16280625	16286565	16345044	16347072	58480

K03D3.12	srz-105	IV	16325132	16326561	D	Y	CB4856	16280625	16286565	16345044	16347072	58480
K03D3.14	K03D3.14	IV	16331325	16332571	D	Y	CB4856	16280625	16286565	16345044	16347072	58480
K03D3.2	K03D3.2	IV	16328095	16328574	D	Y	CB4856	16280625	16286565	16345044	16347072	58480
K03D3.4	srz-61	IV	16321126	16323155	D	Y	CB4856	16280625	16286565	16345044	16347072	58480
K03D3.5	K03D3.5	IV	16320099	16320466	D	Y	CB4856	16280625	16286565	16345044	16347072	58480
K03D7.11	srw-32	V	17508760	17510181	D	Y	KR314	17490847	17491246	17588401	17589546	97156
K03D7.2	srw-31	V	17506556	17508473	D	Y	KR314	17490847	17491246	17588401	17589546	97156
K03D7.4	srh-261	V	17497386	17498941	D	N	CB4856	17493378	17493733	17498290	17499690	4558
K03D7.4	srh-261	V	17497386	17498941	D	Y	JU258	17417977	17418058	17499690	17506165	81633
K03D7.4	srh-261	V	17497386	17498941	D	Y	KR314	17490847	17491246	17588401	17589546	97156
K03D7.4	srh-261	V	17497386	17498941	D	N	MY2	17490847	17493832	17498290	17499690	4459
K03D7.6	srh-118	V	17494273	17496138	D	Y	CB4856	17493378	17493733	17498290	17499690	4558
K03D7.6	srh-118	V	17494273	17496138	D	Y	JU258	17417977	17418058	17499690	17506165	81633
K03D7.6	srh-118	V	17494273	17496138	D	Y	KR314	17490847	17491246	17588401	17589546	97156
K03D7.6	srh-118	V	17494273	17496138	D	Y	MY2	17490847	17493832	17498290	17499690	4459
K03D7.7	fbxa-102	V	17491622	17493799	D	N	CB4856	17435729	17435768	17492477	17492521	56710
K03D7.7	fbxa-102	V	17491622	17493799	D	N	CB4856	17493378	17493733	17498290	17499690	4558
K03D7.7	fbxa-102	V	17491622	17493799	D	Y	JU258	17417977	17418058	17499690	17506165	81633
K03D7.7	fbxa-102	V	17491622	17493799	D	Y	KR314	17490847	17491246	17588401	17589546	97156
K03D7.8	K03D7.8	V	17489469	17491576	D	Y	CB4856	17435729	17435768	17492477	17492521	56710
K03D7.8	K03D7.8	V	17489469	17491576	D	Y	JU258	17417977	17418058	17499690	17506165	81633
K03D7.8	K03D7.8	V	17489469	17491576	D	N	KR314	17360012	17371630	17489726	17490760	118097
K03D7.8	K03D7.8	V	17489469	17491576	D	N	KR314	17490847	17491246	17588401	17589546	97156
K03D7.8	K03D7.8	V	17489469	17491576	D	N	MY2	17435729	17435768	17489726	17490760	53959
K03D7.9	K03D7.9	V	17487313	17488800	D	Y	CB4856	17435729	17435768	17492477	17492521	56710
K03D7.9	K03D7.9	V	17487313	17488800	D	Y	JU258	17417977	17418058	17499690	17506165	81633
K03D7.9	K03D7.9	V	17487313	17488800	D	Y	KR314	17360012	17371630	17489726	17490760	118097
K03D7.9	K03D7.9	V	17487313	17488800	D	Y	MY2	17435729	17435768	17489726	17490760	53959
K03H1.10	K03H1.10	III	9923071	9924690	D	N	MY2	9915171	9918783	9924106	9926330	5324
K03H9.3	K03H9.3	II	6423063	6424762	D	N	MY2	6410141	6423063	6424243	6424287	1181
K05C4.3	K05C4.3	I	14748457	14753125	D	N	CB4854	14747605	14748742	14753256	14755360	4515
K05C4.9	K05C4.9	I	14726865	14730706	D	N	AB1	14725980	14726868	14731782	14732867	4915
K05C4.9	K05C4.9	I	14726865	14730706	D	N	CB4854	14725980	14726868	14731782	14732867	4915
K05C4.9	K05C4.9	I	14726865	14730706	D	N	CB4856	14725980	14726868	14731782	14732867	4915
K05C4.9	K05C4.9	I	14726865	14730706	D	N	KR314	14725980	14726868	14731782	14732867	4915
K05F6.1	fbxb-49	II	1568673	1573306	D	Y	CB4853	1552779	1553074	1573984	1575006	20911
K05F6.1	fbxb-49	II	1568673	1573306	D	Y	CB4856	1547460	1548926	1574076	1574708	25151
K05F6.1	fbxb-49	II	1568673	1573306	D	Y	CB4858	1552779	1552850	1574708	1575006	21859
K05F6.1	fbxb-49	II	1568673	1573306	D	Y	JU258	1534143	1535351	1593891	1593940	58541
K05F6.1	fbxb-49	II	1568673	1573306	D	Y	KR314	1552779	1552850	1573984	1574076	21135
K05F6.10	K05F6.10	II	1574563	1576121	D	N	CB4858	1552779	1552850	1574708	1575006	21859
K05F6.10	K05F6.10	II	1574563	1576121	D	Y	JU258	1534143	1535351	1593891	1593940	58541
K05F6.11	K05F6.11	II	1540934	1542724	D	Y	CB4853	1534583	1535351	1548926	1549814	13576
K05F6.11	K05F6.11	II	1540934	1542724	D	Y	CB4856	1537134	1537855	1547066	1547149	9212
K05F6.11	K05F6.11	II	1540934	1542724	D	Y	CB4858	1534521	1535351	1547819	1549474	12469
K05F6.11	K05F6.11	II	1540934	1542724	D	Y	JU258	1534143	1535351	1593891	1593940	58541
K05F6.11	K05F6.11	II	1540934	1542724	D	Y	KR314	1534583	1535351	1547819	1548926	12469
K05F6.12	K05F6.12	II	1545931	1546997	D	Y	CB4853	1534583	1535351	1548926	1549814	13576
K05F6.12	K05F6.12	II	1545931	1546997	D	Y	CB4856	1537134	1537855	1547066	1547149	9212
K05F6.12	K05F6.12	II	1545931	1546997	D	Y	CB4858	1534521	1535351	1547819	1549474	12469
K05F6.12	K05F6.12	II	1545931	1546997	D	Y	JU258	1534143	1535351	1593891	1593940	58541
K05F6.12	K05F6.12	II	1545931	1546997	D	Y	KR314	1534583	1535351	1547819	1548926	12469
K05F6.2	fbxb-50	II	1556585	1563218	D	Y	CB4853	1552779	1553074	1573984	1575006	20911
K05F6.2	fbxb-50	II	1556585	1563218	D	Y	CB4856	1547460	1548926	1574076	1574708	25151
K05F6.2	fbxb-50	II	1556585	1563218	D	Y	CB4858	1552779	1552850	1574708	1575006	21859
K05F6.2	fbxb-50	II	1556585	1563218	D	Y	JU258	1534143	1535351	1593891	1593940	58541
K05F6.2	fbxb-50	II	1556585	1563218	D	N	JU322	1555783	1556419	1557088	1559751	670
K05F6.2	fbxb-50	II	1556585	1563218	D	Y	KR314	1552779	1552850	1573984	1574076	21135
K05F6.3	fbxb-51	II	1550881	1553308	D	N	CB4853	1552779	1553074	1573984	1575006	20911
K05F6.3	fbxb-51	II	1550881	1553308	D	Y	CB4856	1547460	1548926	1574076	1574708	25151

K05F6.3	fbxb-51	II	1550881	1553308	D	N	CB4858	1552779	1552850	1574708	1575006	21859
K05F6.3	fbxb-51	II	1550881	1553308	D	Y	JU258	1534143	1535351	1593891	1593940	58541
K05F6.3	fbxb-51	II	1550881	1553308	D	N	KR314	1552779	1552850	1573984	1574076	21135
K05F6.4	K05F6.4	II	1543506	1545417	D	Y	CB4853	1534583	1535351	1548926	1549814	13576
K05F6.4	K05F6.4	II	1543506	1545417	D	Y	CB4856	1537134	1537855	1547066	1547149	9212
K05F6.4	K05F6.4	II	1543506	1545417	D	Y	CB4858	1534521	1535351	1547819	1549474	12469
K05F6.4	K05F6.4	II	1543506	1545417	D	Y	JU258	1534143	1535351	1593891	1593940	58541
K05F6.4	K05F6.4	II	1543506	1545417	D	Y	KR314	1534583	1535351	1547819	1548926	12469
K05F6.5	fbxb-44	II	1538897	1539888	D	Y	CB4853	1534583	1535351	1548926	1549814	13576
K05F6.5	fbxb-44	II	1538897	1539888	D	Y	CB4856	1537134	1537855	1547066	1547149	9212
K05F6.5	fbxb-44	II	1538897	1539888	D	Y	CB4858	1534521	1535351	1547819	1549474	12469
K05F6.5	fbxb-44	II	1538897	1539888	D	Y	JU258	1534143	1535351	1593891	1593940	58541
K05F6.5	fbxb-44	II	1538897	1539888	D	Y	KR314	1534583	1535351	1547819	1548926	12469
K05F6.6	fbxb-52	II	1547066	1549958	D	N	CB4853	1534583	1535351	1548926	1549814	13576
K05F6.6	fbxb-52	II	1547066	1549958	D	N	CB4856	1537134	1537855	1547066	1547149	9212
K05F6.6	fbxb-52	II	1547066	1549958	D	N	CB4856	1547460	1548926	1574076	1574708	25151
K05F6.6	fbxb-52	II	1547066	1549958	D	N	CB4858	1534521	1535351	1547819	1549474	12469
K05F6.6	fbxb-52	II	1547066	1549958	D	Y	JU258	1534143	1535351	1593891	1593940	58541
K05F6.6	fbxb-52	II	1547066	1549958	D	N	KR314	1534583	1535351	1547819	1548926	12469
K05F6.7	fbxb-54	II	1555263	1556313	D	Y	CB4853	1552779	1553074	1573984	1575006	20911
K05F6.7	fbxb-54	II	1555263	1556313	D	Y	CB4856	1547460	1548926	1574076	1574708	25151
K05F6.7	fbxb-54	II	1555263	1556313	D	Y	CB4858	1552779	1552850	1574708	1575006	21859
K05F6.7	fbxb-54	II	1555263	1556313	D	Y	JU258	1534143	1535351	1593891	1593940	58541
K05F6.7	fbxb-54	II	1555263	1556313	D	Y	KR314	1552779	1552850	1573984	1574076	21135
K05F6.8	K05F6.8	II	1567330	1567752	D	Y	CB4853	1552779	1553074	1573984	1575006	20911
K05F6.8	K05F6.8	II	1567330	1567752	D	Y	CB4856	1547460	1548926	1574076	1574708	25151
K05F6.8	K05F6.8	II	1567330	1567752	D	Y	CB4858	1552779	1552850	1574708	1575006	21859
K05F6.8	K05F6.8	II	1567330	1567752	D	Y	JU258	1534143	1535351	1593891	1593940	58541
K05F6.8	K05F6.8	II	1567330	1567752	D	Y	KR314	1552779	1552850	1573984	1574076	21135
K05F6.9	fbxb-46	II	1570088	1571133	D	Y	CB4853	1552779	1553074	1573984	1575006	20911
K05F6.9	fbxb-46	II	1570088	1571133	D	Y	CB4856	1547460	1548926	1574076	1574708	25151
K05F6.9	fbxb-46	II	1570088	1571133	D	Y	CB4858	1552779	1552850	1574708	1575006	21859
K05F6.9	fbxb-46	II	1570088	1571133	D	Y	JU258	1534143	1535351	1593891	1593940	58541
K05F6.9	fbxb-46	II	1570088	1571133	D	Y	KR314	1552779	1552850	1573984	1574076	21135
K07C6.10	srx-67	V	3916824	3918431	D	N	JU258	3906298	3906677	3917411	3917762	10735
K07C6.11	srx-68	V	3912794	3914476	D	Y	JU258	3906298	3906677	3917411	3917762	10735
K07C6.2	cyp-35B3	V	3945338	3947029	D	Y	JU258	3922016	3922055	3958835	3958879	36781
K07C6.3	cyp-35B2	V	3942200	3944442	D	Y	JU258	3922016	3922055	3958835	3958879	36781
K07C6.4	cyp-35B1	V	3939112	3941169	D	Y	JU258	3922016	3922055	3958835	3958879	36781
K07C6.5	cyp-35A5	V	3936359	3938486	D	Y	JU258	3922016	3922055	3958835	3958879	36781
K07C6.6	srx-63	V	3931611	3933379	D	Y	JU258	3922016	3922055	3958835	3958879	36781
K07C6.7	srx-64	V	3929508	3930663	D	Y	JU258	3922016	3922055	3958835	3958879	36781
K07C6.8	srx-65	V	3921920	3922949	D	N	JU258	3922016	3922055	3958835	3958879	36781
K07E12.1a	dig-1	III	6746246	6794701	A	N	RW7000	6772349	6776518	6785881	6785932	9364
K07E8.3	sdz-24	II	658675	659873	D	N	JU258	658863	658958	660209	660337	1252
K07E8.3	sdz-24	II	658675	659873	D	N	KR314	658958	658999	660097	660209	1099
K07E8.9	sri-45	II	660097	661802	D	N	JU258	658863	658958	660209	660337	1252
K07E8.9	sri-45	II	660097	661802	D	N	KR314	658958	658999	660097	660209	1099
K08D10.10	K08D10.10	IV	4154391	4157125	D	Y	AB1	4123592	4124569	4166385	4168062	41817
K08D10.10	K08D10.10	IV	4154391	4157125	D	Y	CB4854	4123592	4124569	4166385	4168062	41817
K08D10.10	K08D10.10	IV	4154391	4157125	D	Y	CB4856	4123592	4124569	4166385	4168062	41817
K08D10.10	K08D10.10	IV	4154391	4157125	D	Y	JU258	4123592	4124569	4166385	4168062	41817
K08D10.10	K08D10.10	IV	4154391	4157125	D	Y	JU322	4123592	4124569	4166385	4168062	41817
K08D10.10	K08D10.10	IV	4154391	4157125	D	Y	MY2	4123592	4124569	4166385	4168062	41817
K08D10.7	scrm-8	IV	4164696	4166169	D	Y	AB1	4123592	4124569	4166385	4168062	41817
K08D10.7	scrm-8	IV	4164696	4166169	D	Y	CB4854	4123592	4124569	4166385	4168062	41817
K08D10.7	scrm-8	IV	4164696	4166169	D	Y	CB4856	4123592	4124569	4166385	4168062	41817
K08D10.7	scrm-8	IV	4164696	4166169	D	Y	JU258	4123592	4124569	4166385	4168062	41817
K08D10.7	scrm-8	IV	4164696	4166169	D	Y	JU322	4123592	4124569	4166385	4168062	41817
K08D10.7	scrm-8	IV	4164696	4166169	D	Y	MY2	4123592	4124569	4166385	4168062	41817

K08D10.8	scrm-5	IV	4162510	4164466	D	Y	AB1	4123592	4124569	4166385	4168062	41817
K08D10.8	scrm-5	IV	4162510	4164466	D	Y	CB4854	4123592	4124569	4166385	4168062	41817
K08D10.8	scrm-5	IV	4162510	4164466	D	Y	CB4856	4123592	4124569	4166385	4168062	41817
K08D10.8	scrm-5	IV	4162510	4164466	D	Y	JU258	4123592	4124569	4166385	4168062	41817
K08D10.8	scrm-5	IV	4162510	4164466	D	Y	JU322	4123592	4124569	4166385	4168062	41817
K08D10.8	scrm-5	IV	4162510	4164466	D	Y	MY2	4123592	4124569	4166385	4168062	41817
K08D10.9	K08D10.9	IV	4158184	4161782	D	Y	AB1	4123592	4124569	4166385	4168062	41817
K08D10.9	K08D10.9	IV	4158184	4161782	D	Y	CB4854	4123592	4124569	4166385	4168062	41817
K08D10.9	K08D10.9	IV	4158184	4161782	D	Y	CB4856	4123592	4124569	4166385	4168062	41817
K08D10.9	K08D10.9	IV	4158184	4161782	D	Y	JU258	4123592	4124569	4166385	4168062	41817
K08D10.9	K08D10.9	IV	4158184	4161782	D	Y	JU322	4123592	4124569	4166385	4168062	41817
K08D10.9	K08D10.9	IV	4158184	4161782	D	Y	MY2	4123592	4124569	4166385	4168062	41817
K08D8.1	K08D8.1	IV	12892594	12894323	D	Y	RW7000	12881617	12881730	12968210	12968283	86481
K08D8.2	K08D8.2	IV	12903086	12903731	D	Y	RW7000	12881617	12881730	12968210	12968283	86481
K08D8.3	K08D8.3	IV	12903944	12906593	D	Y	RW7000	12881617	12881730	12968210	12968283	86481
K08D8.4a	K08D8.4	IV	12907438	12909967	D	Y	RW7000	12881617	12881730	12968210	12968283	86481
K08D8.5	K08D8.5	IV	12910616	12912105	D	Y	RW7000	12881617	12881730	12968210	12968283	86481
K08D8.6	K08D8.6	IV	12887109	12890477	D	Y	RW7000	12881617	12881730	12968210	12968283	86481
K08E3.8	mdt-29	III	13773336	13778776	D	N	AB1	13775144	13778160	13780800	NA	2641
K08E3.8	mdt-29	III	13773336	13778776	D	N	CB4856	13775144	13778160	13780800	NA	2641
K08E3.8	mdt-29	III	13773336	13778776	D	N	JU263	13775144	13778160	13780800	NA	2641
K08G2.8	srh-293	V	15866113	15867307	D	N	CB4856	15862579	15863316	15867153	15867258	3838
K08G2.9	srh-299	V	15863316	15864432	D	N	CB4856	15862579	15863316	15867153	15867258	3838
K08G2.9	srh-299	V	15863316	15864432	D	Y	CB4856	15862579	15863316	15867153	15867258	3838
K09D9.10	srx-62	V	3994765	3995923	D	Y	JU258	3989748	3992640	3996008	3997126	3369
K09D9.11	K09D9.11	V	3997109	4001450	D	N	JU258	3997200	3997385	3998697	3998962	1313
K09D9.11	K09D9.11	V	3997109	4001450	D	N	JU258	3999379	3999575	4006785	4007056	7211
K09D9.12	K09D9.12	V	4002312	4005168	D	Y	JU258	3999379	3999575	4006785	4007056	7211
K09D9.8	srh-8	V	4005552	4007502	D	N	JU258	3999379	3999575	4006785	4007056	7211
K09D9.9	K09D9.9	V	3992640	3994124	D	N	JU258	3989748	3992640	3996008	3997126	3369
K09D9.9	K09D9.9	V	3992640	3994124	D	Y	JU258	3989748	3992640	3996008	3997126	3369
K09F6.10	K09F6.10	II	2287334	2290078	D	N	CB4853	2250227	2257254	2289446	2290944	32193
K09F6.10	K09F6.10	II	2287334	2290078	D	Y	CB4856	2263611	2275111	2291077	2291711	15967
K09F6.10	K09F6.10	II	2287334	2290078	D	N	CB4858	2249981	2256954	2289446	2291077	32493
K09F6.10	K09F6.10	II	2287334	2290078	D	Y	JU258	2264403	2265489	2338778	2338818	73290
K09F6.10	K09F6.10	II	2287334	2290078	D	Y	JU263	2265489	2270554	2291117	2291230	20564
K09F6.10	K09F6.10	II	2287334	2290078	D	Y	JU322	2256694	2256733	2298132	2298188	41400
K09F6.10	K09F6.10	II	2287334	2290078	D	Y	KR314	2246518	2249981	2298370	2298409	48390
K09F6.11	K09F6.11	II	2280140	2280581	D	Y	CB4853	2250227	2257254	2289446	2290944	32193
K09F6.11	K09F6.11	II	2280140	2280581	D	Y	CB4856	2263611	2275111	2291077	2291711	15967
K09F6.11	K09F6.11	II	2280140	2280581	D	Y	CB4858	2249981	2256954	2289446	2291077	32493
K09F6.11	K09F6.11	II	2280140	2280581	D	Y	JU258	2264403	2265489	2338778	2338818	73290
K09F6.11	K09F6.11	II	2280140	2280581	D	Y	JU263	2265489	2270554	2291117	2291230	20564
K09F6.11	K09F6.11	II	2280140	2280581	D	Y	JU322	2256694	2256733	2298132	2298188	41400
K09F6.11	K09F6.11	II	2280140	2280581	D	Y	KR314	2246518	2249981	2298370	2298409	48390
K09F6.2	K09F6.2	II	2267064	2270354	D	Y	CB4853	2250227	2257254	2289446	2290944	32193
K09F6.2	K09F6.2	II	2267064	2270354	D	Y	CB4858	2249981	2256954	2289446	2291077	32493
K09F6.2	K09F6.2	II	2267064	2270354	D	Y	JU258	2264403	2265489	2338778	2338818	73290
K09F6.2	K09F6.2	II	2267064	2270354	D	Y	JU322	2256694	2256733	2298132	2298188	41400
K09F6.2	K09F6.2	II	2267064	2270354	D	Y	KR314	2246518	2249981	2298370	2298409	48390
K09F6.3	K09F6.3	II	2256307	2261102	D	N	CB4853	2250227	2257254	2289446	2290944	32193
K09F6.3	K09F6.3	II	2256307	2261102	D	N	CB4858	2249981	2256954	2289446	2291077	32493
K09F6.3	K09F6.3	II	2256307	2261102	D	N	JU322	2256694	2256733	2298132	2298188	41400
K09F6.3	K09F6.3	II	2256307	2261102	D	Y	KR314	2246518	2249981	2298370	2298409	48390
K09F6.4	K09F6.4	II	2262198	2265436	D	Y	CB4853	2250227	2257254	2289446	2290944	32193
K09F6.4	K09F6.4	II	2262198	2265436	D	Y	CB4858	2249981	2256954	2289446	2291077	32493
K09F6.4	K09F6.4	II	2262198	2265436	D	Y	JU322	2256694	2256733	2298132	2298188	41400
K09F6.4	K09F6.4	II	2262198	2265436	D	Y	KR314	2246518	2249981	2298370	2298409	48390
K09F6.5	K09F6.5	II	2272063	2273385	D	Y	CB4853	2250227	2257254	2289446	2290944	32193
K09F6.5	K09F6.5	II	2272063	2273385	D	Y	CB4858	2249981	2256954	2289446	2291077	32493

K09F6.5	K09F6.5	II	2272063	2273385	D	Y	JU258	2264403	2265489	2338778	2338818	73290
K09F6.5	K09F6.5	II	2272063	2273385	D	Y	JU263	2265489	2270554	2291117	2291230	20564
K09F6.5	K09F6.5	II	2272063	2273385	D	Y	JU322	2256694	2256733	2298132	2298188	41400
K09F6.5	K09F6.5	II	2272063	2273385	D	Y	KR314	2246518	2249981	2298370	2298409	48390
K09F6.6	K09F6.6	II	2274877	2279147	D	Y	CB4853	2250227	2257254	2289446	2290944	32193
K09F6.6	K09F6.6	II	2274877	2279147	D	N	CB4856	2263611	2275111	2291077	2291711	15967
K09F6.6	K09F6.6	II	2274877	2279147	D	Y	CB4858	2249981	2256954	2289446	2291077	32493
K09F6.6	K09F6.6	II	2274877	2279147	D	Y	JU258	2264403	2265489	2338778	2338818	73290
K09F6.6	K09F6.6	II	2274877	2279147	D	Y	JU263	2265489	2270554	2291117	2291230	20564
K09F6.6	K09F6.6	II	2274877	2279147	D	Y	JU322	2256694	2256733	2298132	2298188	41400
K09F6.6	K09F6.6	II	2274877	2279147	D	Y	KR314	2246518	2249981	2298370	2298409	48390
K09F6.7	K09F6.7	II	2290918	2292128	D	N	CB4856	2263611	2275111	2291077	2291711	15967
K09F6.7	K09F6.7	II	2290918	2292128	D	Y	JU258	2264403	2265489	2338778	2338818	73290
K09F6.7	K09F6.7	II	2290918	2292128	D	N	JU263	2265489	2270554	2291117	2291230	20564
K09F6.7	K09F6.7	II	2290918	2292128	D	Y	JU322	2256694	2256733	2298132	2298188	41400
K09F6.7	K09F6.7	II	2290918	2292128	D	Y	KR314	2246518	2249981	2298370	2298409	48390
K09F6.8	K09F6.8	II	2294694	2296104	D	N	CB4853	2291875	2294723	2298465	2299972	3743
K09F6.8	K09F6.8	II	2294694	2296104	D	N	CB4858	2291875	2294723	2298465	2299972	3743
K09F6.8	K09F6.8	II	2294694	2296104	D	Y	JU258	2264403	2265489	2338778	2338818	73290
K09F6.8	K09F6.8	II	2294694	2296104	D	N	JU263	2292053	2294723	2298465	2299972	3743
K09F6.8	K09F6.8	II	2294694	2296104	D	Y	JU322	2256694	2256733	2298132	2298188	41400
K09F6.8	K09F6.8	II	2294694	2296104	D	Y	KR314	2246518	2249981	2298370	2298409	48390
K09F6.9	K09F6.9	II	2280679	2282877	D	Y	CB4853	2250227	2257254	2289446	2290944	32193
K09F6.9	K09F6.9	II	2280679	2282877	D	Y	CB4856	2263611	2275111	2291077	2291711	15967
K09F6.9	K09F6.9	II	2280679	2282877	D	Y	CB4858	2249981	2256954	2289446	2291077	32493
K09F6.9	K09F6.9	II	2280679	2282877	D	Y	JU258	2264403	2265489	2338778	2338818	73290
K09F6.9	K09F6.9	II	2280679	2282877	D	Y	JU263	2265489	2270554	2291117	2291230	20564
K09F6.9	K09F6.9	II	2280679	2282877	D	Y	JU322	2256694	2256733	2298132	2298188	41400
K09F6.9	K09F6.9	II	2280679	2282877	D	Y	KR314	2246518	2249981	2298370	2298409	48390
K10C9.3	K10C9.3	V	1061597	1067861	D	N	JU258	1048364	1050243	1061730	1062015	11488
K10C9.3	K10C9.3	V	1061597	1067861	D	N	JU258	1062015	1062600	1062870	1063016	271
K10C9.4	K10C9.4	V	1050128	1053016	D	N	JU258	1048364	1050243	1061730	1062015	11488
K10C9.6	str-67	V	1062590	1064593	D	N	JU258	1062015	1062600	1062870	1063016	271
K10C9.8	str-224	V	1056550	1058989	D	Y	JU258	1048364	1050243	1061730	1062015	11488
K10C9.9	K10C9.9	V	1054606	1055775	D	Y	JU258	1048364	1050243	1061730	1062015	11488
K10G4.10	K10G4.10	V	17273492	17274985	D	Y	JU258	17229936	17238425	17347568	17348479	109144
K10G4.2	srw-47	V	17260642	17262048	D	N	CB4856	17225924	17226138	17261395	17261770	35258
K10G4.2	srw-47	V	17260642	17262048	D	Y	JU258	17229936	17238425	17347568	17348479	109144
K10G4.3	K10G4.3	V	17264034	17264921	D	Y	JU258	17229936	17238425	17347568	17348479	109144
K10G4.5	K10G4.5	V	17298309	17301506	D	N	CB4856	17301021	17301143	17325589	17325981	24447
K10G4.5	K10G4.5	V	17298309	17301506	D	Y	JU258	17229936	17238425	17347568	17348479	109144
K10G4.9	srw-30	V	17287351	17288634	D	Y	JU258	17229936	17238425	17347568	17348479	109144
M01G12.12	rrf-2	I	12098674	12105967	D	N	CB4856	12103762	12104037	12105917	12106851	1881
M01G12.12	rrf-2	I	12098674	12105967	D	N	MY2	12103388	12103872	12105917	12106851	2046
M01G12.14	M01G12.14	I	12106902	12108448	D	N	CB4856	12107194	12107260	12107508	12111325	249
M01G12.14	M01G12.14	I	12106902	12108448	D	N	MY2	12106902	12107118	12108359	12108409	1242
M04C3.1a	M04C3.1	V	19437920	19445532	D	Y	AB1	19389840	19395538	19470658	19470740	75121
M04C3.1a	M04C3.1	V	19437920	19445532	D	Y	CB4853	19389791	19399677	19470658	19470740	70982
M04C3.1a	M04C3.1	V	19437920	19445532	D	Y	CB4854	19395932	19400613	19469469	19470658	68857
M04C3.1a	M04C3.1	V	19437920	19445532	D	Y	CB4856	19400192	19400613	19469168	19470658	68556
M04C3.1a	M04C3.1	V	19437920	19445532	D	Y	CB4858	19395467	19400613	19469469	19470740	68857
M04C3.1a	M04C3.1	V	19437920	19445532	D	Y	JU258	19389840	19390251	19469469	19480048	79219
M04C3.1a	M04C3.1	V	19437920	19445532	D	Y	JU263	19398882	19400613	19469469	19470658	68857
M04C3.1a	M04C3.1	V	19437920	19445532	D	Y	JU322	19400192	19400613	19470658	19470740	70046
M04C3.1a	M04C3.1	V	19437920	19445532	D	N	KR314	19395467	19395538	19440602	19441749	45065
M04C3.1a	M04C3.1	V	19437920	19445532	D	Y	MY2	19389840	19390251	19469469	19470740	79219
M04C3.2	M04C3.2	V	19445858	19448865	D	Y	AB1	19389840	19395538	19470658	19470740	75121
M04C3.2	M04C3.2	V	19445858	19448865	D	Y	CB4853	19389791	19399677	19470658	19470740	70982
M04C3.2	M04C3.2	V	19445858	19448865	D	Y	CB4854	19395932	19400613	19469469	19470658	68857
M04C3.2	M04C3.2	V	19445858	19448865	D	Y	CB4856	19400192	19400613	19469168	19470658	68556

M04C3.2	M04C3.2	V	19445858	19448865	D	Y	CB4858	19395467	19400613	19469469	19470740	68857
M04C3.2	M04C3.2	V	19445858	19448865	D	Y	JU258	19389840	19390251	19469469	19480048	79219
M04C3.2	M04C3.2	V	19445858	19448865	D	Y	JU263	19398882	19400613	19469469	19470658	68857
M04C3.2	M04C3.2	V	19445858	19448865	D	Y	JU322	19400192	19400613	19470658	19470740	70046
M04C3.2	M04C3.2	V	19445858	19448865	D	N	KR314	19446045	19446704	19470658	19470740	23955
M04C3.2	M04C3.2	V	19445858	19448865	D	Y	MY2	19389840	19390251	19469469	19470740	79219
M04C3.3	M04C3.3	V	19444118	19445541	D	Y	AB1	19389840	19395538	19470658	19470740	75121
M04C3.3	M04C3.3	V	19444118	19445541	D	Y	CB4853	19389791	19399677	19470658	19470740	70982
M04C3.3	M04C3.3	V	19444118	19445541	D	Y	CB4854	19395932	19400613	19469469	19470658	68857
M04C3.3	M04C3.3	V	19444118	19445541	D	Y	CB4856	19400192	19400613	19469168	19470658	68556
M04C3.3	M04C3.3	V	19444118	19445541	D	Y	CB4858	19395467	19400613	19469469	19470740	68857
M04C3.3	M04C3.3	V	19444118	19445541	D	Y	JU258	19389840	19390251	19469469	19480048	79219
M04C3.3	M04C3.3	V	19444118	19445541	D	Y	JU263	19398882	19400613	19469469	19470658	68857
M04C3.3	M04C3.3	V	19444118	19445541	D	Y	JU322	19400192	19400613	19470658	19470740	70046
M04C3.3	M04C3.3	V	19444118	19445541	D	Y	MY2	19389840	19390251	19469469	19470740	79219
M199.3	clec-188	IV	15115530	15120202	D	N	KR314	15117600	15117718	15117911	15120494	194
M28.4	M28.4	II	10643625	10646868	D	N	CB4856	10643874	10644116	10644561	10644694	446
M28.4	M28.4	II	10643625	10646868	D	N	CB4858	10643967	10644116	10644561	10644694	446
M28.4	M28.4	II	10643625	10646868	D	N	JU263	10643967	10644116	10644561	10644694	446
ncRNA	21ur-1216	IV	16901559	16901579	D	Y	JU258	16894642	16896609	16903176	16916026	6568
ncRNA	21ur-1243	IV	16341666	16341686	D	Y	CB4856	16280625	16286565	16345044	16347072	58480
ncRNA	21ur-1606	IV	14549308	14549328	D	Y	JU258	14547120	14547780	14550230	14551095	2451
ncRNA	21ur-1803	IV	16335361	16335381	D	Y	CB4856	16280625	16286565	16345044	16347072	58480
ncRNA	21ur-2108	IV	16344041	16344061	D	Y	CB4856	16280625	16286565	16345044	16347072	58480
ncRNA	21ur-2300	IV	17047529	17047549	A	Y	KR314	17043517	17044193	17048260	17053640	4068
ncRNA	21ur-2329	IV	16897102	16897122	D	Y	JU258	16894642	16896609	16903176	16916026	6568
ncRNA	21ur-2515	IV	16346265	16346285	D	Y	KR314	16341964	16345044	16347314	16350519	2271
ncRNA	21ur-2627	IV	16830966	16830986	D	Y	KR314	16827992	16829881	16832313	16832365	2433
ncRNA	21ur-2711	IV	17045980	17046000	A	Y	KR314	17043517	17044193	17048260	17053640	4068
ncRNA	21ur-3031	IV	16341698	16341718	D	Y	CB4856	16280625	16286565	16345044	16347072	58480
ncRNA	21ur-3173	IV	14161937	14161957	D	Y	KR314	14160103	14160518	14161975	14165714	1458
ncRNA	21ur-318	IV	16830295	16830315	D	Y	KR314	16827992	16829881	16832313	16832365	2433
ncRNA	21ur-3372	IV	15946323	15946343	D	Y	JU258	15941399	15944081	15947436	15949714	3356
ncRNA	21ur-353	IV	15946071	15946091	D	Y	JU258	15941399	15944081	15947436	15949714	3356
ncRNA	21ur-3635	IV	15945844	15945864	D	Y	JU258	15941399	15944081	15947436	15949714	3356
ncRNA	21ur-3743	IV	15945743	15945763	D	Y	JU258	15941399	15944081	15947436	15949714	3356
ncRNA	21ur-397	IV	16831272	16831292	D	Y	KR314	16827992	16829881	16832313	16832365	2433
ncRNA	21ur-4067	IV	16831273	16831293	D	Y	KR314	16827992	16829881	16832313	16832365	2433
ncRNA	21ur-4229	IV	15946590	15946610	D	Y	JU258	15941399	15944081	15947436	15949714	3356
ncRNA	21ur-428	IV	16830011	16830031	D	Y	KR314	16827992	16829881	16832313	16832365	2433
ncRNA	21ur-4408	IV	14160975	14160995	D	Y	KR314	14160103	14160518	14161975	14165714	1458
ncRNA	21ur-4453	IV	16897504	16897524	D	Y	JU258	16894642	16896609	16903176	16916026	6568
ncRNA	21ur-4606	IV	16898266	16898286	D	Y	JU258	16894642	16896609	16903176	16916026	6568
ncRNA	21ur-4911	IV	15944918	15944938	D	Y	JU258	15941399	15944081	15947436	15949714	3356
ncRNA	21ur-52	IV	16902940	16902960	D	Y	JU258	16894642	16896609	16903176	16916026	6568
ncRNA	21ur-5294	IV	16897566	16897586	D	Y	JU258	16894642	16896609	16903176	16916026	6568
ncRNA	21ur-572	IV	16898397	16898417	D	Y	JU258	16894642	16896609	16903176	16916026	6568
ncRNA	21ur-738	IV	14549668	14549688	D	Y	JU258	14547120	14547780	14550230	14551095	2451
pseudogene	B0281.7	II	2310072	2311075	D	Y	CB4856	2302120	2306782	2318165	2318664	11384
pseudogene	B0281.7	II	2310072	2311075	D	Y	JU258	2264403	2265489	2338778	2338818	73290
pseudogene	B0281.7	II	2310072	2311075	D	Y	JU263	2302181	2306782	2318165	2318664	11384
pseudogene	B0281.7	II	2310072	2311075	D	Y	JU322	2299972	2300284	2318165	2318664	17882
pseudogene	B0281.7	II	2310072	2311075	D	Y	KR314	2302181	2306782	2318165	2318664	11384
pseudogene	bath-11	II	1913607	1916378	D	Y	CB4853	1823064	1825029	1934411	1934453	109383
pseudogene	bath-11	II	1913607	1916378	D	Y	CB4858	1823064	1825029	1934411	1937196	109383
pseudogene	bath-11	II	1913607	1916378	D	Y	JU258	1860810	1862541	1934453	1937551	71913
pseudogene	bath-11	II	1913607	1916378	D	Y	KR314	1823064	1825029	1934453	1937196	109425
pseudogene	bath-14	II	2048069	2049187	D	Y	CB4856	2033514	2034216	2052296	2052336	18081
pseudogene	bath-14	II	2048069	2049187	D	Y	JU258	2045491	2045536	2050198	2051543	4663
pseudogene	bath-18	II	2522565	2524476	A	Y	JU263	2521204	2521667	2546368	2547362	24702

pseudogene	bath-22	II	1927880	1928735	D	Y	CB4853	1823064	1825029	1934411	1934453	109383
pseudogene	bath-22	II	1927880	1928735	D	Y	CB4856	1922456	1922525	1934453	1937196	11929
pseudogene	bath-22	II	1927880	1928735	D	Y	CB4858	1823064	1825029	1934411	1937196	109383
pseudogene	bath-22	II	1927880	1928735	D	Y	JU258	1860810	1862541	1934453	1937551	71913
pseudogene	bath-22	II	1927880	1928735	D	Y	KR314	1823064	1825029	1934453	1937196	109425
pseudogene	bath-6	II	1918321	1919441	D	Y	CB4853	1823064	1825029	1934411	1934453	109383
pseudogene	bath-6	II	1918321	1919441	D	Y	CB4856	1914552	1916627	1920897	1921622	4271
pseudogene	bath-6	II	1918321	1919441	D	Y	CB4858	1823064	1825029	1934411	1937196	109383
pseudogene	bath-6	II	1918321	1919441	D	Y	JU258	1860810	1862541	1934453	1937551	71913
pseudogene	bath-6	II	1918321	1919441	D	Y	KR314	1823064	1825029	1934453	1937196	109425
pseudogene	bath-8	II	3380283	3381398	D	Y	MY2	3378382	3379217	3394898	3395853	15682
pseudogene	C06C6.10	V	16004694	16004914	A	Y	AB1	16003771	16004293	16024961	16025011	20669
pseudogene	C17H1.1	I	13117617	13120312	D	Y	AB1	13109530	13111296	13153623	13155415	42328
pseudogene	C17H1.1	I	13117617	13120312	D	Y	CB4854	13108633	13111296	13153623	13155415	42328
pseudogene	C17H1.12	I	13127140	13127864	D	Y	AB1	13109530	13111296	13153623	13155415	42328
pseudogene	C17H1.12	I	13127140	13127864	D	Y	CB4854	13108633	13111296	13153623	13155415	42328
pseudogene	C18D4.10	V	17532788	17535335	A	Y	JU258	17510210	17521313	17545755	17547052	24443
pseudogene	C18D4.10	V	17532788	17535335	D	Y	KR314	17490847	17491246	17588401	17589546	97156
pseudogene	C18D4.t1	V	17542413	17542496	A	Y	JU258	17510210	17521313	17545755	17547052	24443
pseudogene	C18D4.t1	V	17542413	17542496	D	Y	KR314	17490847	17491246	17588401	17589546	97156
pseudogene	C25F9.15	V	19418390	19418877	D	Y	AB1	19389840	19395538	19470658	19470740	75121
pseudogene	C25F9.15	V	19418390	19418877	D	Y	CB4853	19389791	19399677	19470658	19470740	70982
pseudogene	C25F9.15	V	19418390	19418877	D	Y	CB4854	19395932	19400613	19469469	19470658	68857
pseudogene	C25F9.15	V	19418390	19418877	D	Y	CB4856	19400192	19400613	19469168	19470658	68556
pseudogene	C25F9.15	V	19418390	19418877	D	Y	CB4858	19395467	19400613	19469469	19470740	68857
pseudogene	C25F9.15	V	19418390	19418877	D	Y	JU258	19389840	19390251	19469469	19480048	79219
pseudogene	C25F9.15	V	19418390	19418877	D	Y	JU263	19398882	19400613	19469469	19470658	68857
pseudogene	C25F9.15	V	19418390	19418877	D	Y	JU322	19400192	19400613	19470658	19470740	70046
pseudogene	C25F9.15	V	19418390	19418877	D	Y	KR314	19395467	19395538	19440602	19441749	45065
pseudogene	C25F9.15	V	19418390	19418877	D	Y	MY2	19389840	19390251	19469469	19470740	79219
pseudogene	C25F9.t1	V	19396953	19397025	D	Y	AB1	19389840	19395538	19470658	19470740	75121
pseudogene	C25F9.t1	V	19396953	19397025	D	Y	JU258	19389840	19390251	19469469	19480048	79219
pseudogene	C25F9.t1	V	19396953	19397025	D	Y	KR314	19395467	19395538	19440602	19441749	45065
pseudogene	C25F9.t1	V	19396953	19397025	D	Y	MY2	19389840	19390251	19469469	19470740	79219
pseudogene	C25F9.t2	V	19396659	19396731	D	Y	AB1	19389840	19395538	19470658	19470740	75121
pseudogene	C25F9.t2	V	19396659	19396731	D	Y	JU258	19389840	19390251	19469469	19480048	79219
pseudogene	C25F9.t2	V	19396659	19396731	D	Y	KR314	19395467	19395538	19440602	19441749	45065
pseudogene	C25F9.t2	V	19396659	19396731	D	Y	MY2	19389840	19390251	19469469	19470740	79219
pseudogene	C25F9.t3	V	19394745	19394814	D	Y	JU258	19389840	19390251	19469469	19480048	79219
pseudogene	C25F9.t3	V	19394745	19394814	D	Y	MY2	19389840	19390251	19469469	19470740	79219
pseudogene	C25F9.t4	V	19398552	19398622	D	Y	AB1	19389840	19395538	19470658	19470740	75121
pseudogene	C25F9.t4	V	19398552	19398622	D	Y	JU258	19389840	19390251	19469469	19480048	79219
pseudogene	C25F9.t4	V	19398552	19398622	D	Y	KR314	19395467	19395538	19440602	19441749	45065
pseudogene	C25F9.t4	V	19398552	19398622	D	Y	MY2	19389840	19390251	19469469	19470740	79219
pseudogene	C25F9.t5	V	19431255	19431327	D	Y	AB1	19389840	19395538	19470658	19470740	75121
pseudogene	C25F9.t5	V	19431255	19431327	D	Y	CB4853	19389791	19399677	19470658	19470740	70982
pseudogene	C25F9.t5	V	19431255	19431327	D	Y	CB4854	19395932	19400613	19469469	19470658	68857
pseudogene	C25F9.t5	V	19431255	19431327	D	Y	CB4856	19400192	19400613	19469168	19470658	68556
pseudogene	C25F9.t5	V	19431255	19431327	D	Y	CB4858	19395467	19400613	19469469	19470740	68857
pseudogene	C25F9.t5	V	19431255	19431327	D	Y	JU258	19389840	19390251	19469469	19480048	79219
pseudogene	C25F9.t5	V	19431255	19431327	D	Y	JU263	19398882	19400613	19469469	19470658	68857
pseudogene	C25F9.t5	V	19431255	19431327	D	Y	JU322	19400192	19400613	19470658	19470740	70046
pseudogene	C25F9.t5	V	19431255	19431327	D	Y	KR314	19395467	19395538	19440602	19441749	45065
pseudogene	C25F9.t5	V	19431255	19431327	D	Y	MY2	19389840	19390251	19469469	19470740	79219
pseudogene	C27C7.9	I	11430143	11430170	D	Y	MY2	11426071	11428999	11431301	11448826	2303
pseudogene	C32H11.7	IV	12930676	12931483	D	Y	RW7000	12881617	12881730	12968210	12968283	86481
pseudogene	C35D6.12	IV	16338522	16339058	D	Y	CB4856	16280625	16286565	16345044	16347072	58480
pseudogene	C35D6.13	IV	16337235	16338398	D	Y	CB4856	16280625	16286565	16345044	16347072	58480
pseudogene	C35D6.6	IV	16336970	16337088	D	Y	CB4856	16280625	16286565	16345044	16347072	58480
pseudogene	C35D6.7	IV	16338467	16338588	D	Y	CB4856	16280625	16286565	16345044	16347072	58480

pseudogene	clec-14	I	12637015	12638407	D	Y	JU258	12627134	12633945	12642554	12643233	8610
pseudogene	clec-14	I	12637015	12638407	D	Y	JU263	12633798	12633945	12642554	12642930	8610
pseudogene	clec-14	I	12637015	12638407	D	Y	KR314	12633798	12633945	12642554	12642742	8610
pseudogene	F07E5.10	II	2077023	2077903	D	Y	CB4853	2068600	2068808	2097680	2103041	28873
pseudogene	F07E5.10	II	2077023	2077903	D	Y	CB4858	2068600	2068808	2097680	2103041	28873
pseudogene	F07E5.10	II	2077023	2077903	D	Y	JU258	2063261	2063759	2138338	2138578	74580
pseudogene	F07E5.10	II	2077023	2077903	D	Y	JU322	2065424	2068458	2081749	2076295	13292
pseudogene	F07E5.10	II	2077023	2077903	D	Y	KR314	2068600	2068808	2099883	2102260	31076
pseudogene	F11A5.18	V	16204400	16204828	D	Y	CB4856	16200953	16203778	16209877	16214948	6100
pseudogene	F11A5.18	V	16204400	16204828	D	Y	JU258	16200953	16203778	16214890	16214948	11113
pseudogene	F11A5.18	V	16204400	16204828	D	Y	MY2	16200953	16203778	16209877	16214948	6100
pseudogene	F11D11.15	V	18755415	18756725	D	Y	AB1	18751971	18752576	18768216	18769880	15641
pseudogene	F11D11.15	V	18755415	18756725	D	Y	CB4854	18751971	18752576	18768216	18769793	15641
pseudogene	F11D11.15	V	18755415	18756725	D	Y	JU258	18751971	18752576	18769922	18770085	17347
pseudogene	F11D11.15	V	18755415	18756725	D	Y	JU263	18751971	18752576	18768316	18769793	15741
pseudogene	F11D11.15	V	18755415	18756725	D	Y	KR314	18751971	18752576	18768216	18769880	15641
pseudogene	F11D11.16	V	18756811	18757643	D	Y	AB1	18751971	18752576	18768216	18769880	15641
pseudogene	F11D11.16	V	18756811	18757643	D	Y	CB4854	18751971	18752576	18768216	18769793	15641
pseudogene	F11D11.16	V	18756811	18757643	D	Y	JU258	18751971	18752576	18769922	18770085	17347
pseudogene	F11D11.16	V	18756811	18757643	D	Y	JU263	18751971	18752576	18768316	18769793	15741
pseudogene	F11D11.16	V	18756811	18757643	D	Y	KR314	18751971	18752576	18768216	18769880	15641
pseudogene	F11D11.17	V	18764202	18764446	D	Y	AB1	18751971	18752576	18768216	18769880	15641
pseudogene	F11D11.17	V	18764202	18764446	D	Y	CB4854	18751971	18752576	18768216	18769793	15641
pseudogene	F11D11.17	V	18764202	18764446	D	Y	JU258	18751971	18752576	18769922	18770085	17347
pseudogene	F11D11.17	V	18764202	18764446	D	Y	JU263	18751971	18752576	18768316	18769793	15741
pseudogene	F11D11.17	V	18764202	18764446	D	Y	KR314	18751971	18752576	18768216	18769880	15641
pseudogene	F11D11.18	V	18769207	18769451	D	Y	JU258	18751971	18752576	18769922	18770085	17347
pseudogene	F11D11.19	V	18767719	18768379	D	N	AB1	18751971	18752576	18768216	18769880	15641
pseudogene	F11D11.19	V	18767719	18768379	D	N	CB4854	18751971	18752576	18768216	18769793	15641
pseudogene	F11D11.19	V	18767719	18768379	D	Y	JU258	18751971	18752576	18769922	18770085	17347
pseudogene	F11D11.19	V	18767719	18768379	D	N	JU263	18751971	18752576	18768316	18769793	15741
pseudogene	F11D11.19	V	18767719	18768379	D	N	KR314	18751971	18752576	18768216	18769880	15641
pseudogene	F14D2.16	II	3328354	3328376	D	Y	JU258	3305367	3306370	3353651	3369831	47282
pseudogene	F14D2.16	II	3328354	3328376	D	Y	JU322	3305367	3309390	3330254	3330712	20865
pseudogene	F14D2.16	II	3328354	3328376	D	Y	KR314	3306283	3309390	3355966	3359208	46577
pseudogene	F19B2.10	V	20171489	20173103	D	Y	MY2	20156820	20158156	20182007	20183240	23852
pseudogene	F19B2.11	V	20144186	20145723	D	Y	AB1	20133405	20133764	20151489	20153948	17726
pseudogene	F19B2.11	V	20144186	20145723	D	Y	CB4856	20133405	20133764	20156235	20156347	22472
pseudogene	F19B2.11	V	20144186	20145723	D	Y	MY2	20133405	20133764	20151489	20154392	17726
pseudogene	F21D9.6	V	19242842	19244950	D	N	CB4856	19240392	19240532	19244840	19247309	4309
pseudogene	F28B1.11	V	17040708	17041930	D	N	JU258	17030051	17030090	17040742	17041452	10653
pseudogene	F31E9.10	V	17330659	17330849	D	Y	CB4856	17328959	17329847	17337901	17338060	8055
pseudogene	F31E9.10	V	17330659	17330849	D	Y	JU258	17229936	17238425	17347568	17348479	109144
pseudogene	F31E9.8	V	17324875	17326136	D	N	CB4856	17301021	17301143	17325589	17325981	24447
pseudogene	F31E9.8	V	17324875	17326136	D	Y	JU258	17229936	17238425	17347568	17348479	109144
pseudogene	F31E9.9	V	17323926	17324209	D	Y	CB4856	17301021	17301143	17325589	17325981	24447
pseudogene	F31E9.9	V	17323926	17324209	D	Y	JU258	17229936	17238425	17347568	17348479	109144
pseudogene	F31E9.t1	V	17317847	17317930	D	Y	CB4856	17301021	17301143	17325589	17325981	24447
pseudogene	F31E9.t1	V	17317847	17317930	D	Y	JU258	17229936	17238425	17347568	17348479	109144
pseudogene	F40F12.8	III	9921097	9921789	D	Y	MY2	9915171	9918783	9924106	9926330	5324
pseudogene	F44F1.1	I	13250507	13252890	D	N	CB4854	13251574	13251795	13252249	13252476	455
pseudogene	F44F1.1	I	13250507	13252890	D	N	CB4856	13251574	13251795	13252249	13252476	455
pseudogene	F46F2.1	X	15250730	15253798	D	N	CB4854	15246290	15250730	15257691	15257840	6962
pseudogene	F46F2.1	X	15250730	15253798	D	Y	CB4854	15246290	15250730	15257691	15257840	6962
pseudogene	F46F2.1	X	15250730	15253798	D	N	CB4858	15246290	15250730	15253216	15253266	2487
pseudogene	F46F2.1	X	15250730	15253798	D	N	JU258	15246072	15250730	15253216	15253266	2487
pseudogene	F46F2.1	X	15250730	15253798	D	N	JU263	15246290	15250730	15253216	15253266	2487
pseudogene	F46F2.1	X	15250730	15253798	D	N	JU322	15246290	15250730	15253216	15253266	2487
pseudogene	F47H4.5	V	17332983	17333610	D	Y	CB4856	17328959	17329847	17337901	17338060	8055
pseudogene	F47H4.5	V	17332983	17333610	D	Y	JU258	17229936	17238425	17347568	17348479	109144

pseudogene	F49F1.13	IV	4143310	4144339	D	Y	AB1	4123592	4124569	4166385	4168062	41817
pseudogene	F49F1.13	IV	4143310	4144339	D	Y	CB4854	4123592	4124569	4166385	4168062	41817
pseudogene	F49F1.13	IV	4143310	4144339	D	Y	CB4856	4123592	4124569	4166385	4168062	41817
pseudogene	F49F1.13	IV	4143310	4144339	D	Y	JU258	4123592	4124569	4166385	4168062	41817
pseudogene	F49F1.13	IV	4143310	4144339	D	Y	JU322	4123592	4124569	4166385	4168062	41817
pseudogene	F49F1.13	IV	4143310	4144339	D	Y	MY2	4123592	4124569	4166385	4168062	41817
pseudogene	F57G4.7	V	17645461	17648681	D	Y	JU258	17639843	17640926	17658110	17658389	17185
pseudogene	F59A1.5	V	17650790	17652949	D	Y	JU258	17639843	17640926	17658110	17658389	17185
pseudogene	F59A1.t6	V	17688401	17688473	D	Y	JU263	17685977	17687230	17690042	17692292	2813
pseudogene	F59A7.10	V	2007360	2007709	D	Y	CB4856	2005570	2006917	2011818	2013518	4902
pseudogene	F59A7.10	V	2007360	2007709	D	Y	JU263	2005570	2006917	2011818	2013518	4902
pseudogene	F59A7.10	V	2007360	2007709	D	Y	MY2	2005570	2006917	2011818	2013518	4902
pseudogene	fbxa-173	V	17562188	17563082	D	Y	KR314	17490847	17491246	17588401	17589546	97156
pseudogene	fbxa-175	V	17547350	17549255	D	Y	KR314	17490847	17491246	17588401	17589546	97156
pseudogene	fbxa-180	V	17319564	17322412	D	Y	CB4856	17301021	17301143	17325589	17325981	24447
pseudogene	fbxa-180	V	17319564	17322412	D	Y	JU258	17229936	17238425	17347568	17348479	109144
pseudogene	fbxa-207	V	16919751	16921292	D	Y	CB4856	16912457	16912533	16921721	16922242	9189
pseudogene	fbxa-217	I	11903724	11905138	D	N	MY2	11904097	11904241	11905065	11907398	825
pseudogene	fbxa-70	III	960255	961334	D	Y	JU258	948244	949229	973819	976540	24591
pseudogene	fbxa-87	V	16128106	16129573	D	N	CB4856	16129474	16129524	16131739	16131780	2216
pseudogene	fbxb-27	II	1646600	1647571	D	N	CB4856	1646825	1647203	1649806	1650120	2604
pseudogene	fbxb-27	II	1646600	1647571	D	Y	JU258	1645645	1645716	1660932	1663710	15217
pseudogene	H25P06.3	I	11307161	11307829	D	N	KR314	11297160	11299293	11307482	11311827	8190
pseudogene	K02E7.5	II	1062870	1063214	D	Y	CB4856	1058490	1058886	1071402	1074143	12517
pseudogene	K02E7.5	II	1062870	1063214	D	Y	KR314	1050030	1056548	1072536	1074041	15989
pseudogene	K03D7.1	V	17514773	17519262	D	Y	KR314	17490847	17491246	17588401	17589546	97156
pseudogene	K03D7.3	V	17499011	17504988	D	N	JU258	17417977	17418058	17499690	17506165	81633
pseudogene	K03D7.3	V	17499011	17504988	D	Y	KR314	17490847	17491246	17588401	17589546	97156
pseudogene	K03D7.5	V	17496512	17497253	D	Y	CB4856	17493378	17493733	17498290	17499690	4558
pseudogene	K03D7.5	V	17496512	17497253	D	Y	JU258	17417977	17418058	17499690	17506165	81633
pseudogene	K03D7.5	V	17496512	17497253	D	Y	KR314	17490847	17491246	17588401	17589546	97156
pseudogene	K03D7.5	V	17496512	17497253	D	Y	MY2	17490847	17493832	17498290	17499690	4459
pseudogene	K10G4.11	V	17291294	17291683	D	Y	JU258	17229936	17238425	17347568	17348479	109144
pseudogene	K10G4.12	V	17288770	17289080	D	Y	JU258	17229936	17238425	17347568	17348479	109144
pseudogene	K10G4.13	V	17294381	17297327	D	Y	JU258	17229936	17238425	17347568	17348479	109144
pseudogene	K10G4.4	V	17278335	17285137	D	N	CB4856	17264834	17277557	17284376	17294972	6820
pseudogene	K10G4.4	V	17278335	17285137	D	Y	JU258	17229936	17238425	17347568	17348479	109144
pseudogene	M04C3.4	V	19450205	19450793	D	Y	AB1	19389840	19395538	19470658	19470740	75121
pseudogene	M04C3.4	V	19450205	19450793	D	Y	CB4853	19389791	19399677	19470658	19470740	70982
pseudogene	M04C3.4	V	19450205	19450793	D	Y	CB4854	19395932	19400613	19469469	19470658	68857
pseudogene	M04C3.4	V	19450205	19450793	D	Y	CB4856	19400192	19400613	19469168	19470658	68556
pseudogene	M04C3.4	V	19450205	19450793	D	Y	CB4858	19395467	19400613	19469469	19470740	68857
pseudogene	M04C3.4	V	19450205	19450793	D	Y	JU258	19389840	19390251	19469469	19480048	79219
pseudogene	M04C3.4	V	19450205	19450793	D	Y	JU263	19398882	19400613	19469469	19470658	68857
pseudogene	M04C3.4	V	19450205	19450793	D	Y	JU322	19400192	19400613	19470658	19470740	70046
pseudogene	M04C3.4	V	19450205	19450793	D	Y	KR314	19446045	19446704	19470658	19470740	23955
pseudogene	M04C3.4	V	19450205	19450793	D	Y	MY2	19389840	19390251	19469469	19470740	79219
pseudogene	math-12	II	1890853	1891701	D	Y	CB4853	1823064	1825029	1934411	1934453	109383
pseudogene	math-12	II	1890853	1891701	D	Y	CB4856	1845546	1846078	1912838	1913611	66761
pseudogene	math-12	II	1890853	1891701	D	Y	CB4858	1823064	1825029	1934411	1937196	109383
pseudogene	math-12	II	1890853	1891701	D	Y	JU258	1860810	1862541	1934453	1937551	71913
pseudogene	math-12	II	1890853	1891701	D	Y	KR314	1823064	1825029	1934453	1937196	109425
pseudogene	mir-257	V	17140652	17140673	D	Y	CB4856	17116985	17140588	17140688	17142183	101
pseudogene	R04E5.t3	X	8819864	8819935	D	Y	AB1	8818444	8819702	8820075	8820225	374
pseudogene	R04E5.t3	X	8819864	8819935	D	Y	CB4854	8819702	8819702	8820075	8820225	374
pseudogene	R04E5.t3	X	8819864	8819935	D	Y	KR314	8818444	8819702	8820075	8820225	374
pseudogene	R08H2.15	V	15363163	15363398	A	Y	RW7000	15282709	15282820	15399718	15400303	116899
pseudogene	R08H2.16	V	15380520	15380825	A	Y	RW7000	15282709	15282820	15399718	15400303	116899
pseudogene	R09D1.4	II	9446010	9447625	D	N	KR314	9447204	9447341	9448316	9448356	976
pseudogene	R09D1.9	II	9462457	9464013	D	N	CB4853	9456556	9461666	9462702	9462878	1037

pseudogene	R09D1.9	II	9462457	9464013	D	N	CB4858	9456556	9461666	9462702	9462878	1037
pseudogene	R09D1.9	II	9462457	9464013	D	N	KR314	9462954	9463030	9464015	9464549	986
pseudogene	R09D1.9	II	9462457	9464013	D	N	MY2	9456556	9461666	9462702	9462878	1037
pseudogene	R10E8.4	V	18246773	18249428	D	N	JU258	18186176	18186552	18247813	18248607	61262
pseudogene	R10E8.7	V	18231600	18237188	D	N	CB4856	18223164	18224107	18235017	18235065	10911
pseudogene	R10E8.7	V	18231600	18237188	D	Y	JU258	18186176	18186552	18247813	18248607	61262
pseudogene	rac-3	IV	16311039	16319663	D	Y	CB4856	16280625	16286565	16345044	16347072	58480
pseudogene	rac-3	IV	16311039	16319663	D	N	JU258	16311929	16312660	16318032	16318912	5373
pseudogene	rrn-2.1	I	15064288	15064440	A	Y	JU258	15059572	15064238	15067425	NA	3188
pseudogene	rrn-2.1	I	15064288	15064440	A	Y	KR314	15059572	15064238	15067425	NA	3188
pseudogene	rrn-3.1	I	15064825	15068333	A	N	JU258	15059572	15064238	15067425	NA	3188
pseudogene	rrn-3.1	I	15064825	15068333	A	N	KR314	15059572	15064238	15067425	NA	3188
pseudogene	sdz-29	II	2123688	2129282	D	Y	CB4853	2103403	2103518	2138338	2138410	34821
pseudogene	sdz-29	II	2123688	2129282	D	N	CB4856	2122184	2123890	2126592	2126908	2703
pseudogene	sdz-29	II	2123688	2129282	D	Y	CB4858	2103403	2103518	2138338	2138410	34821
pseudogene	sdz-29	II	2123688	2129282	D	Y	JU258	2063261	2063759	2138338	2138578	74580
pseudogene	sdz-29	II	2123688	2129282	D	N	JU322	2123890	2124560	2128567	2131308	4008
pseudogene	sdz-29	II	2123688	2129282	D	Y	KR314	2103403	2103518	2138338	2138410	34821
pseudogene	srab-15	V	787433	789890	D	N	JU258	788174	789364	789489	789814	126
pseudogene	srbc-28	V	17432961	17435177	D	Y	JU258	17417977	17418058	17499690	17506165	81633
pseudogene	srbc-28	V	17432961	17435177	D	Y	KR314	17360012	17371630	17489726	17490760	118097
pseudogene	srbc-4	V	3383922	3385143	D	Y	CB4856	3319416	3319804	3423047	3423415	103244
pseudogene	srbc-4	V	3383922	3385143	D	Y	JU258	3246578	3247858	3433564	3441667	185707
pseudogene	srbc-4	V	3383922	3385143	D	Y	MY2	3248872	3249051	3433136	3433521	184086
pseudogene	srg-43	V	17553837	17555742	D	Y	KR314	17490847	17491246	17588401	17589546	97156
pseudogene	srh-106	V	15363580	15364989	A	Y	RW7000	15282709	15282820	15399718	15400303	116899
pseudogene	srh-107	V	15365274	15366801	A	Y	RW7000	15282709	15282820	15399718	15400303	116899
pseudogene	srh-108	V	15367135	15368265	A	Y	RW7000	15282709	15282820	15399718	15400303	116899
pseudogene	srh-110	V	17478300	17479353	D	Y	CB4856	17435729	17435768	17492477	17492521	56710
pseudogene	srh-110	V	17478300	17479353	D	Y	JU258	17417977	17418058	17499690	17506165	81633
pseudogene	srh-110	V	17478300	17479353	D	Y	KR314	17360012	17371630	17489726	17490760	118097
pseudogene	srh-110	V	17478300	17479353	D	Y	MY2	17435729	17435768	17489726	17490760	53959
pseudogene	srh-114	V	17472286	17473446	D	Y	CB4856	17435729	17435768	17492477	17492521	56710
pseudogene	srh-114	V	17472286	17473446	D	Y	JU258	17417977	17418058	17499690	17506165	81633
pseudogene	srh-114	V	17472286	17473446	D	Y	KR314	17360012	17371630	17489726	17490760	118097
pseudogene	srh-114	V	17472286	17473446	D	Y	MY2	17435729	17435768	17489726	17490760	53959
pseudogene	srh-117	V	17271698	17272856	D	Y	JU258	17229936	17238425	17347568	17348479	109144
pseudogene	srh-121	V	17398422	17399364	D	Y	KR314	17360012	17371630	17489726	17490760	118097
pseudogene	srh-156	V	17450776	17452099	D	Y	CB4856	17435729	17435768	17492477	17492521	56710
pseudogene	srh-156	V	17450776	17452099	D	Y	JU258	17417977	17418058	17499690	17506165	81633
pseudogene	srh-156	V	17450776	17452099	D	Y	KR314	17360012	17371630	17489726	17490760	118097
pseudogene	srh-156	V	17450776	17452099	D	Y	MY2	17435729	17435768	17489726	17490760	53959
pseudogene	srh-157	V	17448300	17449558	D	Y	CB4856	17435729	17435768	17492477	17492521	56710
pseudogene	srh-157	V	17448300	17449558	D	Y	JU258	17417977	17418058	17499690	17506165	81633
pseudogene	srh-157	V	17448300	17449558	D	Y	KR314	17360012	17371630	17489726	17490760	118097
pseudogene	srh-157	V	17448300	17449558	D	Y	MY2	17435729	17435768	17489726	17490760	53959
pseudogene	srh-158	V	17442549	17445636	D	Y	CB4856	17435729	17435768	17492477	17492521	56710
pseudogene	srh-158	V	17442549	17445636	D	Y	JU258	17417977	17418058	17499690	17506165	81633
pseudogene	srh-158	V	17442549	17445636	D	Y	KR314	17360012	17371630	17489726	17490760	118097
pseudogene	srh-158	V	17442549	17445636	D	Y	MY2	17435729	17435768	17489726	17490760	53959
pseudogene	srh-160	V	17446316	17447604	D	Y	CB4856	17435729	17435768	17492477	17492521	56710
pseudogene	srh-160	V	17446316	17447604	D	Y	JU258	17417977	17418058	17499690	17506165	81633
pseudogene	srh-160	V	17446316	17447604	D	Y	KR314	17360012	17371630	17489726	17490760	118097
pseudogene	srh-160	V	17446316	17447604	D	Y	MY2	17435729	17435768	17489726	17490760	53959
pseudogene	srh-161	V	17452644	17453773	D	Y	CB4856	17435729	17435768	17492477	17492521	56710
pseudogene	srh-161	V	17452644	17453773	D	Y	JU258	17417977	17418058	17499690	17506165	81633
pseudogene	srh-161	V	17452644	17453773	D	Y	KR314	17360012	17371630	17489726	17490760	118097
pseudogene	srh-161	V	17452644	17453773	D	Y	MY2	17435729	17435768	17489726	17490760	53959
pseudogene	srh-175	V	17439079	17440340	D	Y	CB4856	17435729	17435768	17492477	17492521	56710
pseudogene	srh-175	V	17439079	17440340	D	Y	JU258	17417977	17418058	17499690	17506165	81633

pseudogene	srh-175	V	17439079	17440340	D	Y	KR314	17360012	17371630	17489726	17490760	118097
pseudogene	srh-175	V	17439079	17440340	D	Y	MY2	17435729	17435768	17489726	17490760	53959
pseudogene	srh-224	IV	16341585	16345402	D	N	CB4856	16280625	16286565	16345044	16347072	58480
pseudogene	srh-224	IV	16341585	16345402	D	N	KR314	16341964	16345044	16347314	16350519	2271
pseudogene	srh-253	V	17480818	17481822	D	Y	CB4856	17435729	17435768	17492477	17492521	56710
pseudogene	srh-253	V	17480818	17481822	D	Y	JU258	17417977	17418058	17499690	17506165	81633
pseudogene	srh-253	V	17480818	17481822	D	Y	KR314	17360012	17371630	17489726	17490760	118097
pseudogene	srh-253	V	17480818	17481822	D	Y	MY2	17435729	17435768	17489726	17490760	53959
pseudogene	srh-262	V	17275857	17278238	D	N	CB4856	17264834	17277557	17284376	17294972	6820
pseudogene	srh-262	V	17275857	17278238	D	Y	JU258	17229936	17238425	17347568	17348479	109144
pseudogene	srh-294	V	15865062	15865785	D	Y	CB4856	15862579	15863316	15867153	15867258	3838
pseudogene	srh-96	V	17419124	17420266	D	Y	JU258	17417977	17418058	17499690	17506165	81633
pseudogene	srh-96	V	17419124	17420266	D	Y	KR314	17360012	17371630	17489726	17490760	118097
pseudogene	sri-58	II	3187281	3188823	D	Y	MY2	3180783	3180993	3196064	3196905	15072
pseudogene	sri-76	II	2606775	2608559	D	Y	AB1	2587455	2588254	2610765	2611484	22512
pseudogene	sri-76	II	2606775	2608559	D	Y	KR314	2585541	2588254	2610765	2611484	22512
pseudogene	srt-60	V	4272019	4273315	D	N	CB4856	4269683	4269991	4272768	4281647	2778
pseudogene	srw-125	V	2997091	2997916	D	Y	CB4856	2994908	2996205	2998538	2998914	2334
pseudogene	srw-126	V	2953192	2954288	D	Y	JU258	2934122	2936892	2956596	2958453	19705
pseudogene	srw-126	V	2953192	2954288	D	Y	MY2	2934122	2936892	2955369	2956596	18478
pseudogene	srw-128	V	2946812	2948415	D	Y	JU258	2934122	2936892	2956596	2958453	19705
pseudogene	srw-128	V	2946812	2948415	D	Y	MY2	2934122	2936892	2955369	2956596	18478
pseudogene	srw-37	V	17028853	17030417	D	N	CB4853	17029351	17029756	17029796	17029847	41
pseudogene	srw-37	V	17028853	17030417	D	N	JU258	17030051	17030090	17040742	17041452	10653
pseudogene	srw-45	V	17511500	17519824	D	Y	KR314	17490847	17491246	17588401	17589546	97156
pseudogene	srw-46	V	17292182	17293697	D	Y	JU258	17229936	17238425	17347568	17348479	109144
pseudogene	srw-70	V	15328632	15330040	A	Y	RW7000	15282709	15282820	15399718	15400303	116899
pseudogene	srx-100	II	3691778	3693225	D	Y	JU258	3672184	3672299	3693900	3694195	21602
pseudogene	srx-11	V	7914400	7915708	D	N	CB4856	7912455	7914630	7915161	7915612	532
pseudogene	srx-49	V	15299166	15300382	A	Y	RW7000	15282709	15282820	15399718	15400303	116899
pseudogene	srx-52	V	17402492	17404154	D	Y	KR314	17360012	17371630	17489726	17490760	118097
pseudogene	srx-57	V	7376391	7377724	D	N	CB4856	7376413	7376559	7377511	7377756	953
pseudogene	srx-61	V	3909083	3910930	D	Y	JU258	3906298	3906677	3917411	3917762	10735
pseudogene	srx-69	V	3923360	3924318	D	Y	JU258	3922016	3922055	3958835	3958879	36781
pseudogene	srx-70	V	3933945	3934935	D	Y	JU258	3922016	3922055	3958835	3958879	36781
pseudogene	srx-94	V	3622371	3623685	D	N	JU258	3620750	3622989	3623920	3625014	932
pseudogene	srx-94	V	3622371	3623685	D	Y	MY2	3620750	3622287	3623920	3625014	1634
pseudogene	srx-99	II	3685946	3687127	D	Y	JU258	3672184	3672299	3693900	3694195	21602
pseudogene	srz-21	IV	16326678	16327925	D	Y	CB4856	16280625	16286565	16345044	16347072	58480
pseudogene	srz-26	V	17316319	17317687	D	Y	CB4856	17301021	17301143	17325589	17325981	24447
pseudogene	srz-26	V	17316319	17317687	D	Y	JU258	17229936	17238425	17347568	17348479	109144
pseudogene	srz-33	V	20170376	20178144	D	Y	MY2	20156820	20158156	20182007	20183240	23852
pseudogene	srz-34	V	20168209	20169387	D	Y	MY2	20156820	20158156	20182007	20183240	23852
pseudogene	srz-35	V	19834791	19836860	A	Y	MY2	19825429	19833649	19846181	19849102	12533
pseudogene	srz-36	V	20162697	20164671	D	Y	AB1	20153948	20154082	20167559	20179231	13478
pseudogene	srz-36	V	20162697	20164671	D	Y	CB4856	20156386	20156759	20167559	20179231	10801
pseudogene	srz-36	V	20162697	20164671	D	Y	MY2	20156820	20158156	20182007	20183240	23852
pseudogene	srz-39	IV	16306272	16306799	D	Y	CB4856	16280625	16286565	16345044	16347072	58480
pseudogene	srz-40	IV	16291948	16293030	D	Y	CB4856	16280625	16286565	16345044	16347072	58480
pseudogene	srz-41	IV	16300548	16301706	D	Y	CB4856	16280625	16286565	16345044	16347072	58480
pseudogene	srz-50	V	17406274	17407471	D	Y	KR314	17360012	17371630	17489726	17490760	118097
pseudogene	srz-51	V	17404579	17405791	D	Y	KR314	17360012	17371630	17489726	17490760	118097
pseudogene	srz-57	V	17542686	17544075	A	Y	JU258	17510210	17521313	17545755	17547052	24443
pseudogene	srz-57	V	17542686	17544075	D	Y	KR314	17490847	17491246	17588401	17589546	97156
pseudogene	srz-68	II	3238164	3240038	D	Y	JU258	3195432	3196064	3249275	3250319	53212
pseudogene	srz-73	IV	16289518	16290795	D	Y	CB4856	16280625	16286565	16345044	16347072	58480
pseudogene	srz-89	V	3947350	3948937	D	Y	JU258	3922016	3922055	3958835	3958879	36781
pseudogene	srz-92	V	17030707	17032741	D	Y	JU258	17030051	17030090	17040742	17041452	10653
pseudogene	srz-93	V	17036379	17037831	D	Y	JU258	17030051	17030090	17040742	17041452	10653
pseudogene	str-21	II	1791984	1792965	D	Y	JU258	1750470	1750664	1822745	1822788	72082

pseudogene	str-22	II	1802110	1803305	D	Y	CB4853	1766357	1801449	1822360	1822666	20912
pseudogene	str-22	II	1802110	1803305	D	Y	CB4856	1801303	1801449	1807444	1807483	5996
pseudogene	str-22	II	1802110	1803305	D	Y	CB4858	1766357	1801449	1822360	1822666	20912
pseudogene	str-22	II	1802110	1803305	D	Y	JU258	1750470	1750664	1822745	1822788	72082
pseudogene	str-22	II	1802110	1803305	D	Y	KR314	1799744	1800302	1822618	1822666	22317
pseudogene	str-34	V	4477547	4479177	D	N	CB4854	4454913	4471278	4477553	4480754	6276
pseudogene	str-35	V	4474829	4476982	D	Y	CB4854	4454913	4471278	4477553	4480754	6276
pseudogene	str-58	V	3253468	3254589	D	Y	JU258	3246578	3247858	3433564	3441667	185707
pseudogene	str-58	V	3253468	3254589	D	Y	MY2	3248872	3249051	3433136	3433521	184086
pseudogene	T02G6.9	I	11822936	11823359	D	Y	CB4856	11819980	11820557	11827573	11827651	7017
pseudogene	T03E6.12	V	16582360	16582638	D	Y	CB4856	16581129	16581424	16583891	16586754	2468
pseudogene	T10H4.1	V	15284218	15285527	A	Y	RW7000	15282709	15282820	15399718	15400303	116899
pseudogene	T19C9.10	V	17237358	17237584	D	Y	CB4856	17225924	17226138	17261395	17261770	35258
pseudogene	T19C9.11	V	17235095	17235546	D	Y	CB4856	17225924	17226138	17261395	17261770	35258
pseudogene	T19C9.6	V	17229523	17229958	D	Y	CB4856	17225924	17226138	17261395	17261770	35258
pseudogene	T19C9.9	V	17234297	17234598	D	Y	CB4856	17225924	17226138	17261395	17261770	35258
pseudogene	T23D5.13	V	15747014	15747455	D	Y	JU258	15730588	15730913	15760733	15761000	29821
pseudogene	T23D5.14	V	15745378	15745626	D	Y	JU258	15730588	15730913	15760733	15761000	29821
pseudogene	T23D5.15	V	15745852	15746060	D	Y	JU258	15730588	15730913	15760733	15761000	29821
pseudogene	T23D5.16	V	15752827	15753216	D	Y	JU258	15730588	15730913	15760733	15761000	29821
pseudogene	T23D5.5	V	15736994	15739633	D	Y	JU258	15730588	15730913	15760733	15761000	29821
pseudogene	T26H2.12	V	19232250	19233651	A	Y	CB4856	19228374	19230781	19237263	19238432	6483
pseudogene	T27C5.15	V	17399438	17399968	D	Y	KR314	17360012	17371630	17489726	17490760	118097
pseudogene	T27C5.16	V	17407942	17408351	D	Y	KR314	17360012	17371630	17489726	17490760	118097
pseudogene	T27C5.17	V	17408405	17408611	D	Y	KR314	17360012	17371630	17489726	17490760	118097
pseudogene	T27C5.6	V	17412570	17413066	D	Y	KR314	17360012	17371630	17489726	17490760	118097
pseudogene	T28F3.7	IV	17302197	17304325	D	N	KR314	17302790	17302829	17302955	17302994	127
pseudogene	W02A11.6	I	12754080	12755115	D	Y	CB4856	12750598	12751464	12758513	12758603	7050
pseudogene	W03H1.t1	X	1775484	1775556	D	Y	CB4856	1747004	1752189	1778099	1781395	25911
pseudogene	Y113G7B.26	V	20220244	20220846	D	N	AB1	20219626	20220579	20224266	20229051	3688
pseudogene	Y113G7B.26	V	20220244	20220846	D	N	CB4853	20214424	20220767	20224266	20228945	3500
pseudogene	Y113G7B.26	V	20220244	20220846	D	N	CB4854	20214424	20220767	20224266	20228945	3500
pseudogene	Y113G7B.26	V	20220244	20220846	D	Y	CB4856	20214424	20217225	20224266	20228945	7042
pseudogene	Y113G7B.26	V	20220244	20220846	D	N	CB4858	20214424	20220579	20224266	20228945	3688
pseudogene	Y113G7B.26	V	20220244	20220846	D	N	JU258	20219626	20220767	20227256	20228945	6490
pseudogene	Y113G7B.26	V	20220244	20220846	D	N	JU263	20219626	20220579	20224266	20228945	3688
pseudogene	Y113G7B.26	V	20220244	20220846	D	N	JU322	20214424	20220767	20227256	20228945	6490
pseudogene	Y113G7B.26	V	20220244	20220846	D	Y	KR314	20214424	20217225	20224266	20228945	7042
pseudogene	Y116A8C.48	IV	17142638	17142938	D	Y	JU258	17131081	17134566	17145165	17146720	10600
pseudogene	Y116A8C.53	IV	17137553	17138018	D	Y	JU258	17131081	17134566	17145165	17146720	10600
pseudogene	Y17D7C.5	V	18709076	18709323	D	N	KR314	18708143	18709091	18711469	18714705	2379
pseudogene	Y26D4A.15	I	13073825	13075112	D	Y	AB1	13071050	13072099	13081538	13081763	9440
pseudogene	Y26D4A.16	I	13075110	13075369	D	Y	AB1	13071050	13072099	13081538	13081763	9440
pseudogene	Y26D4A.18	I	13078465	13078868	D	Y	AB1	13071050	13072099	13081538	13081763	9440
pseudogene	Y26D4A.19	I	13080178	13080489	D	Y	AB1	13071050	13072099	13081538	13081763	9440
pseudogene	Y26D4A.20	I	13085275	13086342	D	Y	AB1	13084717	13084921	13104191	13107776	19271
pseudogene	Y43F8A.t1	V	19393484	19393556	D	Y	JU258	19389840	19390251	19469469	19480048	79219
pseudogene	Y43F8A.t1	V	19393484	19393556	D	Y	MY2	19389840	19390251	19469469	19470740	79219
pseudogene	Y43F8B.17	V	19484799	19485836	D	Y	JU258	19480130	19480251	19492283	19494156	12033
pseudogene	Y43F8B.17	V	19484799	19485836	D	Y	KR314	19480130	19480251	19492283	19494156	12033
pseudogene	Y43F8B.7	V	19490010	19491194	D	Y	JU258	19480130	19480251	19492283	19494156	12033
pseudogene	Y43F8B.7	V	19490010	19491194	D	Y	KR314	19480130	19480251	19492283	19494156	12033
pseudogene	Y43F8B.8	V	19483601	19483859	D	Y	JU258	19480130	19480251	19492283	19494156	12033
pseudogene	Y43F8B.8	V	19483601	19483859	D	Y	KR314	19480130	19480251	19492283	19494156	12033
pseudogene	Y46G5A.41	II	12754518	12754785	D	Y	AB1	12749888	12754432	12755646	12756261	1215
pseudogene	Y46G5A.41	II	12754518	12754785	D	Y	CB4853	12749888	12754432	12755646	12756261	1215
pseudogene	Y46G5A.41	II	12754518	12754785	D	Y	CB4854	12749888	12754432	12755646	12756261	1215
pseudogene	Y46G5A.41	II	12754518	12754785	D	Y	CB4856	12749101	12749888	12757363	12760568	7476
pseudogene	Y46G5A.41	II	12754518	12754785	D	Y	JU258	12746108	12746571	12757363	12760568	10793
pseudogene	Y46G5A.41	II	12754518	12754785	D	Y	JU263	12749101	12754432	12757363	12760568	2932

pseudogene	Y46G5A.41	II	12754518	12754785	D	Y	JU322	12749101	12749888	12757363	12760568	7476
pseudogene	Y46G5A.41	II	12754518	12754785	D	Y	KR314	12749888	12754432	12755646	12756261	1215
pseudogene	Y46G5A.41	II	12754518	12754785	D	Y	MY2	12746108	12746571	12757363	12760568	10793
pseudogene	Y46G5A.42	II	12749001	12749289	D	Y	JU258	12746108	12746571	12757363	12760568	10793
pseudogene	Y46G5A.42	II	12749001	12749289	D	Y	MY2	12746108	12746571	12757363	12760568	10793
pseudogene	Y46G5A.6	II	12743699	12746207	D	N	JU263	12743578	12743815	12746622	12749013	2808
pseudogene	Y47H9C.14	I	11897397	11898511	D	N	CB4856	11896805	11897641	11903091	11903854	5451
pseudogene	Y47H9C.14	I	11897397	11898511	D	N	CB4858	11896805	11897641	11900122	11902248	2482
pseudogene	Y47H9C.14	I	11897397	11898511	D	N	JU258	11896805	11897641	11903091	11903854	5451
pseudogene	Y47H9C.14	I	11897397	11898511	D	N	JU263	11896805	11897641	11903091	11903578	5451
pseudogene	Y47H9C.14	I	11897397	11898511	D	N	MY2	11896805	11897641	11903091	11903578	5451
pseudogene	Y51A2A.13	V	18282960	18283129	D	Y	JU258	18277830	18282879	18287431	18289554	4553
pseudogene	Y51A2A.13	V	18282960	18283129	D	Y	MY2	18277830	18282879	18287431	18289554	4553
pseudogene	Y51A2A.14	V	18296393	18297331	D	Y	CB4856	18283066	18285026	18300599	18318725	15574
pseudogene	Y51A2A.2	V	18287272	18288357	D	Y	AB1	18283066	18285026	18291314	18292896	6289
pseudogene	Y51A2A.2	V	18287272	18288357	D	Y	CB4853	18283066	18285026	18291314	18292896	6289
pseudogene	Y51A2A.2	V	18287272	18288357	D	Y	CB4854	18283066	18285026	18291314	18292896	6289
pseudogene	Y51A2A.2	V	18287272	18288357	D	Y	CB4856	18283066	18285026	18300599	18318725	15574
pseudogene	Y51A2A.2	V	18287272	18288357	D	Y	CB4858	18283066	18285026	18291314	18292896	6289
pseudogene	Y51A2A.2	V	18287272	18288357	D	N	JU258	18277830	18282879	18287431	18289554	4553
pseudogene	Y51A2A.2	V	18287272	18288357	D	Y	JU263	18283066	18285026	18291314	18292896	6289
pseudogene	Y51A2A.2	V	18287272	18288357	D	Y	JU322	18283066	18285026	18291314	18292896	6289
pseudogene	Y51A2A.2	V	18287272	18288357	D	Y	KR314	18283066	18285026	18291314	18292896	6289
pseudogene	Y51A2A.2	V	18287272	18288357	D	N	MY2	18277830	18282879	18287431	18289554	4553
pseudogene	Y51A2A.3	V	18289523	18290638	D	Y	AB1	18283066	18285026	18291314	18292896	6289
pseudogene	Y51A2A.3	V	18289523	18290638	D	Y	CB4853	18283066	18285026	18291314	18292896	6289
pseudogene	Y51A2A.3	V	18289523	18290638	D	Y	CB4854	18283066	18285026	18291314	18292896	6289
pseudogene	Y51A2A.3	V	18289523	18290638	D	Y	CB4856	18283066	18285026	18300599	18318725	15574
pseudogene	Y51A2A.3	V	18289523	18290638	D	Y	CB4858	18283066	18285026	18291314	18292896	6289
pseudogene	Y51A2A.3	V	18289523	18290638	D	Y	JU263	18283066	18285026	18291314	18292896	6289
pseudogene	Y51A2A.3	V	18289523	18290638	D	Y	JU322	18283066	18285026	18291314	18292896	6289
pseudogene	Y51A2A.3	V	18289523	18290638	D	Y	KR314	18283066	18285026	18291314	18292896	6289
pseudogene	Y53F4B.43	II	14988433	14991212	D	N	KR314	14983264	14983789	14991178	14994915	7390
pseudogene	Y53H1A.6	I	11235377	11235586	D	N	JU258	11233192	11234306	11235432	11235536	1127
pseudogene	Y53H1A.6	I	11235377	11235586	D	N	KR314	11233192	11234306	11235432	11235536	1127
pseudogene	Y61B8A.3	V	17250238	17252152	D	Y	CB4856	17225924	17226138	17261395	17261770	35258
pseudogene	Y61B8A.3	V	17250238	17252152	D	Y	JU258	17229936	17238425	17347568	17348479	109144
pseudogene	Y61B8B.2	V	17305750	17307703	D	Y	CB4856	17301021	17301143	17325589	17325981	24447
pseudogene	Y61B8B.2	V	17305750	17307703	D	Y	JU258	17229936	17238425	17347568	17348479	109144
pseudogene	Y68A4A.4	V	17195353	17196373	D	Y	CB4856	17190486	17190902	17197119	17197300	6218
pseudogene	Y68A4A.5	V	17192999	17198971	D	N	CB4856	17190486	17190902	17197119	17197300	6218
pseudogene	Y68A4B.4	V	17242316	17242545	D	Y	CB4856	17225924	17226138	17261395	17261770	35258
pseudogene	Y68A4B.4	V	17242316	17242545	D	Y	JU258	17229936	17238425	17347568	17348479	109144
pseudogene	Y69A2AR.11	IV	2584774	2587685	D	Y	CB4856	2571288	2572089	2588387	2593325	16299
pseudogene	Y69A2AR.11	IV	2584774	2587685	D	Y	JU258	2556903	2563486	2594976	2600114	31491
pseudogene	Y69A2AR.11	IV	2584774	2587685	D	Y	JU322	2570796	2572089	2588387	2592669	16299
pseudogene	Y75D11A.t2	X	1764250	1764323	D	Y	CB4856	1747004	1752189	1778099	1781395	25911
pseudogene	Y75D11A.t2	X	1764250	1764323	D	Y	JU322	1747004	1752189	1774025	1777351	21837
pseudogene	Y7A9C.1	IV	16294114	16298439	D	Y	CB4856	16280625	16286565	16345044	16347072	58480
pseudogene	Y7A9C.1	IV	16294114	16298439	D	N	KR314	16294202	16294716	16296254	16296323	1539
pseudogene	Y7A9C.11	IV	16293159	16293442	D	Y	CB4856	16280625	16286565	16345044	16347072	58480
pseudogene	Y7A9C.12	IV	16293579	16293883	D	Y	CB4856	16280625	16286565	16345044	16347072	58480
pseudogene	Y7A9C.13	IV	16298773	16298972	D	Y	CB4856	16280625	16286565	16345044	16347072	58480
pseudogene	Y7A9C.2	IV	16303574	16306193	D	Y	CB4856	16280625	16286565	16345044	16347072	58480
pseudogene	Y94A7B.12	V	17813572	17813882	D	Y	CB4856	17811878	17812669	17833335	17836695	20667
pseudogene	Y94A7B.12	V	17813572	17813882	D	Y	JU258	17801700	17801810	17820902	17822222	19093
pseudogene	Y94A7B.12	V	17813572	17813882	D	Y	MY2	17811918	17812669	17833335	17836695	20667
pseudogene	ZC239.1	II	3227253	3228290	D	Y	CB4856	3224933	3224997	3237080	3240493	12084
pseudogene	ZC239.1	II	3227253	3228290	D	Y	JU258	3195432	3196064	3249275	3250319	53212
pseudogene	ZC239.1	II	3227253	3228290	D	Y	KR314	3224933	3224997	3237080	3241072	12084

pseudogene	ZC239.1	II	3227253	3228290	D	Y	MY2	3224933	3225304	3237080	3240493	11777
pseudogene	ZK1037.12	V	15310096	15310272	A	Y	RW7000	15282709	15282820	15399718	15400303	116899
pseudogene	ZK1037.2	V	15312958	15314206	A	Y	RW7000	15282709	15282820	15399718	15400303	116899
pseudogene	ZK666.13	II	10479841	10480002	A	Y	CB4858	10477044	10477549	10490009	10490052	12461
pseudogene	ZK666.13	II	10479841	10480002	A	Y	JU322	10477044	10477549	10490009	10490052	12461
pseudogene	ZK896.3	IV	12883476	12886096	D	Y	RW7000	12881617	12881730	12968210	12968283	86481
R03D7.2	R03D7.2	II	10932710	10935062	D	N	JU263	10933488	10933557	10933646	10933687	90
R03D7.2	R03D7.2	II	10932710	10935062	D	N	JU322	10933488	10933557	10933646	10933687	90
R03D7.2	R03D7.2	II	10932710	10935062	D	N	KR314	10933488	10933557	10933646	10933687	90
R03E1.4	R03E1.4	X	14155037	14156935	D	N	CB4854	14155096	14155765	14156181	14156568	417
R03H10.7	R03H10.7	II	4175543	4178921	D	N	CB4856	4175763	4176391	4178872	4180162	2482
R05A10.7	R05A10.7	IV	14160373	14162626	D	N	KR314	14160103	14160518	14161975	14165714	1458
R05C11.2	R05C11.2	IV	2057877	2059110	D	N	CB4856	2058104	2058317	2058554	2058693	238
R06B10.1	R06B10.1	III	966641	972012	D	Y	JU258	948244	949229	973819	976540	24591
R06B10.2	R06B10.2	III	973298	978524	D	N	JU258	948244	949229	973819	976540	24591
R07C12.1	R07C12.1	IV	4152540	4154022	D	Y	AB1	4123592	4124569	4166385	4168062	41817
R07C12.1	R07C12.1	IV	4152540	4154022	D	Y	CB4854	4123592	4124569	4166385	4168062	41817
R07C12.1	R07C12.1	IV	4152540	4154022	D	Y	CB4856	4123592	4124569	4166385	4168062	41817
R07C12.1	R07C12.1	IV	4152540	4154022	D	Y	JU258	4123592	4124569	4166385	4168062	41817
R07C12.1	R07C12.1	IV	4152540	4154022	D	Y	JU322	4123592	4124569	4166385	4168062	41817
R07C12.1	R07C12.1	IV	4152540	4154022	D	Y	MY2	4123592	4124569	4166385	4168062	41817
R07C12.2	R07C12.2	IV	4148128	4150057	D	Y	AB1	4123592	4124569	4166385	4168062	41817
R07C12.2	R07C12.2	IV	4148128	4150057	D	Y	CB4854	4123592	4124569	4166385	4168062	41817
R07C12.2	R07C12.2	IV	4148128	4150057	D	Y	CB4856	4123592	4124569	4166385	4168062	41817
R07C12.2	R07C12.2	IV	4148128	4150057	D	Y	JU258	4123592	4124569	4166385	4168062	41817
R07C12.2	R07C12.2	IV	4148128	4150057	D	Y	JU322	4123592	4124569	4166385	4168062	41817
R07C12.2	R07C12.2	IV	4148128	4150057	D	Y	MY2	4123592	4124569	4166385	4168062	41817
R07C12.3	R07C12.3	IV	4144544	4147250	D	Y	AB1	4123592	4124569	4166385	4168062	41817
R07C12.3	R07C12.3	IV	4144544	4147250	D	Y	CB4854	4123592	4124569	4166385	4168062	41817
R07C12.3	R07C12.3	IV	4144544	4147250	D	Y	CB4856	4123592	4124569	4166385	4168062	41817
R07C12.3	R07C12.3	IV	4144544	4147250	D	Y	JU258	4123592	4124569	4166385	4168062	41817
R07C12.3	R07C12.3	IV	4144544	4147250	D	Y	JU322	4123592	4124569	4166385	4168062	41817
R07C12.3	R07C12.3	IV	4144544	4147250	D	Y	MY2	4123592	4124569	4166385	4168062	41817
R07C12.4	R07C12.4	IV	4150746	4152318	D	Y	AB1	4123592	4124569	4166385	4168062	41817
R07C12.4	R07C12.4	IV	4150746	4152318	D	Y	CB4854	4123592	4124569	4166385	4168062	41817
R07C12.4	R07C12.4	IV	4150746	4152318	D	Y	CB4856	4123592	4124569	4166385	4168062	41817
R07C12.4	R07C12.4	IV	4150746	4152318	D	Y	JU258	4123592	4124569	4166385	4168062	41817
R07C12.4	R07C12.4	IV	4150746	4152318	D	Y	JU322	4123592	4124569	4166385	4168062	41817
R07C12.4	R07C12.4	IV	4150746	4152318	D	Y	MY2	4123592	4124569	4166385	4168062	41817
R07C3.10	R07C3.10	II	909890	911598	D	Y	KR314	898611	899953	912358	912496	12406
R07C3.11	R07C3.11	II	907541	908787	D	Y	KR314	898611	899953	912358	912496	12406
R07C3.12	clec-44	II	919581	922708	D	N	KR314	914929	915056	921010	923894	5955
R07C3.13	R07C3.13	II	926537	927865	D	Y	KR314	925050	925195	930141	931534	4947
R07C3.14	R07C3.14	II	929491	930190	D	Y	KR314	925050	925195	930141	931534	4947
R07C3.15	R07C3.15	II	917630	919423	D	Y	KR314	914929	915056	921010	923894	5955
R07C3.5	R07C3.5	II	931190	932333	D	N	JU258	930141	931534	931848	931947	315
R07C3.7	R07C3.7	II	916057	917474	D	Y	KR314	914929	915056	921010	923894	5955
R07C3.8	R07C3.8	II	913686	915213	D	N	KR314	912720	912907	913697	914170	791
R07C3.8	R07C3.8	II	913686	915213	D	N	KR314	914929	915056	921010	923894	5955
R07C3.9	R07C3.9	II	912009	913036	D	N	KR314	898611	899953	912358	912496	12406
R07C3.9	R07C3.9	II	912009	913036	D	N	KR314	912720	912907	913697	914170	791
R07E5.6	R07E5.6	III	4398784	4400914	D	N	CB4853	4399160	4399209	4399607	4399822	399
R07E5.6	R07E5.6	III	4398784	4400914	D	N	CB4858	4399160	4399209	4399607	4399879	399
R08H2.1	dhs-23	V	15381073	15382281	A	Y	RW7000	15282709	15282820	15399718	15400303	116899
R08H2.10	R08H2.10	V	15373652	15374089	A	Y	RW7000	15282709	15282820	15399718	15400303	116899
R08H2.13	str-102	V	15385009	15386629	A	Y	RW7000	15282709	15282820	15399718	15400303	116899
R08H2.2	str-88	V	15382415	15383815	A	Y	RW7000	15282709	15282820	15399718	15400303	116899
R08H2.3	srh-170	V	15386899	15388846	A	Y	RW7000	15282709	15282820	15399718	15400303	116899
R08H2.4	srh-251	V	15378343	15379893	A	Y	RW7000	15282709	15282820	15399718	15400303	116899
R08H2.5	srh-122	V	15376477	15377979	A	Y	RW7000	15282709	15282820	15399718	15400303	116899

R08H2.7	srh-52	V	15374876	15376299	A	Y	RW7000	15282709	15282820	15399718	15400303	116899
R08H2.8	R08H2.8	V	15370843	15372067	A	Y	RW7000	15282709	15282820	15399718	15400303	116899
R08H2.9	nhr-269	V	15368446	15370191	A	Y	RW7000	15282709	15282820	15399718	15400303	116899
R09D1.5	R09D1.5	II	9448196	9449589	D	N	KR314	9447204	9447341	9448316	9448356	976
R09D1.6	R09D1.6	II	9452621	9454204	D	N	CB4854	9452204	9453833	9456556	9461666	2724
R09D1.6	R09D1.6	II	9452621	9454204	D	N	JU322	9452204	9453833	9456556	9461666	2724
R09D1.7	R09D1.7	II	9455337	9456537	D	Y	CB4854	9452204	9453833	9456556	9461666	2724
R09D1.7	R09D1.7	II	9455337	9456537	D	Y	JU322	9452204	9453833	9456556	9461666	2724
R09D1.8	R09D1.8	II	9460048	9461892	D	N	CB4853	9456556	9461666	9462702	9462878	1037
R09D1.8	R09D1.8	II	9460048	9461892	D	N	CB4858	9456556	9461666	9462702	9462878	1037
R09D1.8	R09D1.8	II	9460048	9461892	D	N	MY2	9456556	9461666	9462702	9462878	1037
R10E8.1	R10E8.1	V	18242327	18243912	D	Y	JU258	18186176	18186552	18247813	18248607	61262
R10E8.3	R10E8.3	V	18234495	18236722	D	N	CB4856	18223164	18224107	18235017	18235065	10911
R10E8.3	R10E8.3	V	18234495	18236722	D	Y	JU258	18186176	18186552	18247813	18248607	61262
R10E8.8	R10E8.8	V	18238211	18240694	D	Y	JU258	18186176	18186552	18247813	18248607	61262
R160.6	R160.6	X	4371011	4373186	D	N	CB4856	4370756	4371018	4371601	4372946	584
R52.1	sdz-28	II	2131081	2132400	D	Y	CB4853	2103403	2103518	2138338	2138410	34821
R52.1	sdz-28	II	2131081	2132400	D	Y	CB4858	2103403	2103518	2138338	2138410	34821
R52.1	sdz-28	II	2131081	2132400	D	Y	JU258	2063261	2063759	2138338	2138578	74580
R52.1	sdz-28	II	2131081	2132400	D	Y	KR314	2103403	2103518	2138338	2138410	34821
R52.2	R52.2	II	2125628	2128687	D	Y	CB4853	2103403	2103518	2138338	2138410	34821
R52.2	R52.2	II	2125628	2128687	D	N	CB4856	2122184	2123890	2126592	2126908	2703
R52.2	R52.2	II	2125628	2128687	D	Y	CB4858	2103403	2103518	2138338	2138410	34821
R52.2	R52.2	II	2125628	2128687	D	Y	JU258	2063261	2063759	2138338	2138578	74580
R52.2	R52.2	II	2125628	2128687	D	N	JU322	2123890	2124560	2128567	2131308	4008
R52.2	R52.2	II	2125628	2128687	D	Y	KR314	2103403	2103518	2138338	2138410	34821
R52.3	math-35	II	2104525	2106867	D	Y	CB4853	2103403	2103518	2138338	2138410	34821
R52.3	math-35	II	2104525	2106867	D	Y	CB4858	2103403	2103518	2138338	2138410	34821
R52.3	math-35	II	2104525	2106867	D	Y	JU258	2063261	2063759	2138338	2138578	74580
R52.3	math-35	II	2104525	2106867	D	Y	KR314	2103403	2103518	2138338	2138410	34821
R52.4	R52.4	II	2107198	2108396	D	Y	CB4853	2103403	2103518	2138338	2138410	34821
R52.4	R52.4	II	2107198	2108396	D	Y	CB4858	2103403	2103518	2138338	2138410	34821
R52.4	R52.4	II	2107198	2108396	D	Y	JU258	2063261	2063759	2138338	2138578	74580
R52.4	R52.4	II	2107198	2108396	D	Y	KR314	2103403	2103518	2138338	2138410	34821
R52.5	R52.5	II	2108788	2110044	D	Y	CB4853	2103403	2103518	2138338	2138410	34821
R52.5	R52.5	II	2108788	2110044	D	Y	CB4858	2103403	2103518	2138338	2138410	34821
R52.5	R52.5	II	2108788	2110044	D	Y	JU258	2063261	2063759	2138338	2138578	74580
R52.5	R52.5	II	2108788	2110044	D	Y	KR314	2103403	2103518	2138338	2138410	34821
R52.6	R52.6	II	2112275	2113049	D	Y	CB4853	2103403	2103518	2138338	2138410	34821
R52.6	R52.6	II	2112275	2113049	D	Y	CB4858	2103403	2103518	2138338	2138410	34821
R52.6	R52.6	II	2112275	2113049	D	Y	JU258	2063261	2063759	2138338	2138578	74580
R52.6	R52.6	II	2112275	2113049	D	Y	KR314	2103403	2103518	2138338	2138410	34821
R52.7	srh-195	II	2114632	2116895	D	Y	CB4853	2103403	2103518	2138338	2138410	34821
R52.7	srh-195	II	2114632	2116895	D	Y	CB4858	2103403	2103518	2138338	2138410	34821
R52.7	srh-195	II	2114632	2116895	D	Y	JU258	2063261	2063759	2138338	2138578	74580
R52.7	srh-195	II	2114632	2116895	D	Y	KR314	2103403	2103518	2138338	2138410	34821
R52.8	math-36	II	2117277	2120171	D	Y	CB4853	2103403	2103518	2138338	2138410	34821
R52.8	math-36	II	2117277	2120171	D	Y	CB4858	2103403	2103518	2138338	2138410	34821
R52.8	math-36	II	2117277	2120171	D	Y	JU258	2063261	2063759	2138338	2138578	74580
R52.8	math-36	II	2117277	2120171	D	Y	KR314	2103403	2103518	2138338	2138410	34821
R52.9	math-37	II	2120739	2122182	D	Y	CB4853	2103403	2103518	2138338	2138410	34821
R52.9	math-37	II	2120739	2122182	D	Y	CB4858	2103403	2103518	2138338	2138410	34821
R52.9	math-37	II	2120739	2122182	D	Y	JU258	2063261	2063759	2138338	2138578	74580
R52.9	math-37	II	2120739	2122182	D	Y	KR314	2103403	2103518	2138338	2138410	34821
T01G5.6	T01G5.6	V	15113757	15114068	D	N	MY2	15113321	15113662	15113908	15116823	247
T02G6.6	T02G6.6	I	11819529	11822579	D	N	CB4856	11819980	11820557	11827573	11827651	7017
T02G6.7	T02G6.7	I	11827539	11828609	D	N	CB4856	11819980	11820557	11827573	11827651	7017
T02G6.7	T02G6.7	I	11827539	11828609	D	N	JU258	11822485	11827573	11828672	11830555	1100
T02G6.7	T02G6.7	I	11827539	11828609	D	N	JU263	11822485	11827573	11828672	11830555	1100
T03D3.4	srh-36	V	2819580	2821667	D	N	JU258	2811563	2811604	2820099	2820229	8496

T03E6.2	T03E6.2	V	16581009	16583147	D	N	CB4856	16581129	16581424	16583891	16586754	2468
T03E6.4	str-150	V	16583245	16585146	D	N	CB4856	16581129	16581424	16583891	16586754	2468
T04F3.1	T04F3.1	V	11740208	11754003	D	N	CB4856	11746905	11747545	11747708	11748599	164
T06D4.1a	T06D4.1	II	3381831	3383948	D	Y	MY2	3378382	3379217	3394898	3395853	15682
T06D4.2	T06D4.2	II	3384057	3386106	D	Y	MY2	3378382	3379217	3394898	3395853	15682
T06D4.3	T06D4.3	II	3388275	3391581	D	Y	MY2	3378382	3379217	3394898	3395853	15682
T06D4.4	T06D4.4	II	3391959	3395026	D	N	MY2	3378382	3379217	3394898	3395853	15682
T06E6.1	T06E6.1	V	15393094	15394931	A	Y	RW7000	15282709	15282820	15399718	15400303	116899
T06E6.15	T06E6.15	V	15398915	15399767	A	Y	RW7000	15282709	15282820	15399718	15400303	116899
T06E6.2a	cyb-3	V	15395672	15397325	A	Y	RW7000	15282709	15282820	15399718	15400303	116899
T06E6.3	fbxa-199	V	15397361	15398804	A	Y	RW7000	15282709	15282820	15399718	15400303	116899
T07D3.1	fbxa-200	II	904452	906045	D	Y	KR314	898611	899953	912358	912496	12406
T07D3.2	T07D3.2	II	901938	903621	D	Y	KR314	898611	899953	912358	912496	12406
T07D3.3	T07D3.3	II	893869	897385	D	N	CB4856	885580	885958	894076	896181	8119
T07D3.3	T07D3.3	II	893869	897385	D	Y	KR314	890566	890611	898191	898230	7581
T07D3.4	T07D3.4	II	888311	890715	D	Y	CB4856	885580	885958	894076	896181	8119
T07D3.4	T07D3.4	II	888311	890715	D	N	KR314	885580	885958	888366	888617	2409
T07D3.4	T07D3.4	II	888311	890715	D	N	KR314	890566	890611	898191	898230	7581
T07D3.5	T07D3.5	II	885490	887036	D	N	CB4856	885580	885958	894076	896181	8119
T07D3.5	T07D3.5	II	885490	887036	D	N	KR314	885580	885958	888366	888617	2409
T07D3.9a	T07D3.9	II	898065	900743	D	N	KR314	890566	890611	898191	898230	7581
T07D3.9a	T07D3.9	II	898065	900743	D	N	KR314	898611	899953	912358	912496	12406
T07H3.1	bath-47	II	1601794	1604614	D	N	CB4853	1599345	1599389	1602642	1604234	3254
T07H3.1	bath-47	II	1601794	1604614	D	N	CB4858	1599345	1599389	1602642	1604234	3254
T07H3.1	bath-47	II	1601794	1604614	D	N	JU258	1599162	1599389	1604148	1609098	4760
T07H3.1	bath-47	II	1601794	1604614	D	N	KR314	1599345	1599389	1602642	1604234	3254
T07H3.2	bath-46	II	1598034	1599477	D	N	CB4853	1599345	1599389	1602642	1604234	3254
T07H3.2	bath-46	II	1598034	1599477	D	N	CB4858	1599345	1599389	1602642	1604234	3254
T07H3.2	bath-46	II	1598034	1599477	D	N	JU258	1594186	1594744	1598815	1599162	4072
T07H3.2	bath-46	II	1598034	1599477	D	N	JU258	1599162	1599389	1604148	1609098	4760
T07H3.2	bath-46	II	1598034	1599477	D	N	KR314	1599345	1599389	1602642	1604234	3254
T07H3.3a	math-38	II	1577484	1579977	D	Y	JU258	1534143	1535351	1593891	1593940	58541
T07H3.4	clec-21	II	1582881	1584245	D	Y	JU258	1534143	1535351	1593891	1593940	58541
T07H3.4	clec-21	II	1582881	1584245	D	N	KR314	1579659	1580007	1583344	1583420	3338
T07H3.4	clec-21	II	1582881	1584245	D	N	KR314	1583852	1583891	1594186	1594744	10296
T07H3.5	clec-20	II	1593485	1595242	D	N	JU258	1534143	1535351	1593891	1593940	58541
T07H3.5	clec-20	II	1593485	1595242	D	N	JU258	1594186	1594744	1598815	1599162	4072
T07H3.5	clec-20	II	1593485	1595242	D	N	KR314	1583852	1583891	1594186	1594744	10296
T07H3.6	bath-26	II	1599476	1600719	D	Y	CB4853	1599345	1599389	1602642	1604234	3254
T07H3.6	bath-26	II	1599476	1600719	D	Y	CB4858	1599345	1599389	1602642	1604234	3254
T07H3.6	bath-26	II	1599476	1600719	D	Y	JU258	1599162	1599389	1604148	1609098	4760
T07H3.6	bath-26	II	1599476	1600719	D	Y	KR314	1599345	1599389	1602642	1604234	3254
T07H3.7	T07H3.7	II	1588547	1589950	D	Y	JU258	1534143	1535351	1593891	1593940	58541
T07H3.7	T07H3.7	II	1588547	1589950	D	Y	KR314	1583852	1583891	1594186	1594744	10296
T08E11.1	T08E11.1	II	1836515	1840295	D	Y	CB4853	1823064	1825029	1934411	1934453	109383
T08E11.1	T08E11.1	II	1836515	1840295	D	Y	CB4856	1819944	1820301	1842836	1843613	22536
T08E11.1	T08E11.1	II	1836515	1840295	D	Y	CB4858	1823064	1825029	1934411	1937196	109383
T08E11.1	T08E11.1	II	1836515	1840295	D	Y	JU258	1823064	1825029	1855280	1860255	30252
T08E11.1	T08E11.1	II	1836515	1840295	D	Y	JU322	1822140	1822245	1841617	1842274	19373
T08E11.1	T08E11.1	II	1836515	1840295	D	Y	KR314	1823064	1825029	1934453	1937196	109425
T08E11.2	math-39	II	1832356	1833503	D	Y	CB4853	1823064	1825029	1934411	1934453	109383
T08E11.2	math-39	II	1832356	1833503	D	Y	CB4856	1819944	1820301	1842836	1843613	22536
T08E11.2	math-39	II	1832356	1833503	D	Y	CB4858	1823064	1825029	1934411	1937196	109383
T08E11.2	math-39	II	1832356	1833503	D	Y	JU258	1823064	1825029	1855280	1860255	30252
T08E11.2	math-39	II	1832356	1833503	D	Y	JU322	1822140	1822245	1841617	1842274	19373
T08E11.2	math-39	II	1832356	1833503	D	Y	KR314	1823064	1825029	1934453	1937196	109425
T08E11.3	math-40	II	1830650	1831824	D	Y	CB4853	1823064	1825029	1934411	1934453	109383
T08E11.3	math-40	II	1830650	1831824	D	Y	CB4856	1819944	1820301	1842836	1843613	22536
T08E11.3	math-40	II	1830650	1831824	D	Y	CB4858	1823064	1825029	1934411	1937196	109383
T08E11.3	math-40	II	1830650	1831824	D	Y	JU258	1823064	1825029	1855280	1860255	30252

T08E11.3	math-40	II	1830650	1831824	D	Y	JU322	1822140	1822245	1841617	1842274	19373
T08E11.3	math-40	II	1830650	1831824	D	Y	KR314	1823064	1825029	1934453	1937196	109425
T08E11.4	math-41	II	1824632	1830198	D	N	CB4853	1823064	1825029	1934411	1934453	109383
T08E11.4	math-41	II	1824632	1830198	D	Y	CB4856	1819944	1820301	1842836	1843613	22536
T08E11.4	math-41	II	1824632	1830198	D	N	CB4858	1823064	1825029	1934411	1937196	109383
T08E11.4	math-41	II	1824632	1830198	D	N	JU258	1823064	1825029	1855280	1860255	30252
T08E11.4	math-41	II	1824632	1830198	D	Y	JU322	1822140	1822245	1841617	1842274	19373
T08E11.4	math-41	II	1824632	1830198	D	N	KR314	1823064	1825029	1934453	1937196	109425
T08E11.5	T08E11.5	II	1821029	1823042	D	N	CB4853	1766357	1801449	1822360	1822666	20912
T08E11.5	T08E11.5	II	1821029	1823042	D	Y	CB4856	1819944	1820301	1842836	1843613	22536
T08E11.5	T08E11.5	II	1821029	1823042	D	N	CB4858	1766357	1801449	1822360	1822666	20912
T08E11.5	T08E11.5	II	1821029	1823042	D	N	JU258	1750470	1750664	1822745	1822788	72082
T08E11.5	T08E11.5	II	1821029	1823042	D	N	JU322	1822140	1822245	1841617	1842274	19373
T08E11.5	T08E11.5	II	1821029	1823042	D	N	KR314	1799744	1800302	1822618	1822666	22317
T08E11.6	fbxb-10	II	1817284	1818403	D	Y	CB4853	1766357	1801449	1822360	1822666	20912
T08E11.6	fbxb-10	II	1817284	1818403	D	Y	CB4858	1766357	1801449	1822360	1822666	20912
T08E11.6	fbxb-10	II	1817284	1818403	D	Y	JU258	1750470	1750664	1822745	1822788	72082
T08E11.6	fbxb-10	II	1817284	1818403	D	Y	KR314	1799744	1800302	1822618	1822666	22317
T08E11.7	fbxa-3	II	1818961	1820005	D	Y	CB4853	1766357	1801449	1822360	1822666	20912
T08E11.7	fbxa-3	II	1818961	1820005	D	Y	CB4858	1766357	1801449	1822360	1822666	20912
T08E11.7	fbxa-3	II	1818961	1820005	D	Y	JU258	1750470	1750664	1822745	1822788	72082
T08E11.7	fbxa-3	II	1818961	1820005	D	Y	KR314	1799744	1800302	1822618	1822666	22317
T08E11.8	T08E11.8	II	1833630	1835786	D	Y	CB4853	1823064	1825029	1934411	1934453	109383
T08E11.8	T08E11.8	II	1833630	1835786	D	Y	CB4856	1819944	1820301	1842836	1843613	22536
T08E11.8	T08E11.8	II	1833630	1835786	D	Y	CB4858	1823064	1825029	1934411	1937196	109383
T08E11.8	T08E11.8	II	1833630	1835786	D	Y	JU258	1823064	1825029	1855280	1860255	30252
T08E11.8	T08E11.8	II	1833630	1835786	D	Y	JU322	1822140	1822245	1841617	1842274	19373
T08E11.8	T08E11.8	II	1833630	1835786	D	Y	KR314	1823064	1825029	1934453	1937196	109425
T08H10.1	T08H10.1	V	4483454	4486491	A	N	CB4854	4477553	4480754	4485583	4486325	4830
T08H10.3	T08H10.3	V	4471365	4473588	D	Y	CB4854	4454913	4471278	4477553	4480754	6276
T08H10.4	T08H10.4	V	4480418	4481532	A	N	CB4854	4477553	4480754	4485583	4486325	4830
T09E11.10	T09E11.10	I	12345809	12347255	D	N	MY2	12345894	12346085	12421673	12430881	75589
T09E11.12	T09E11.12	I	12364330	12365938	D	Y	MY2	12345894	12346085	12421673	12430881	75589
T09E11.3	T09E11.3	I	12369179	12370597	D	N	CB4856	12370046	12370413	12372095	12372444	1683
T09E11.3	T09E11.3	I	12369179	12370597	D	Y	JU258	12368252	12369129	12372570	12374007	3442
T09E11.3	T09E11.3	I	12369179	12370597	D	Y	KR314	12368252	12369129	12372095	12374007	2967
T09E11.3	T09E11.3	I	12369179	12370597	D	Y	MY2	12345894	12346085	12421673	12430881	75589
T09E11.4	T09E11.4	I	12359932	12363406	D	Y	MY2	12345894	12346085	12421673	12430881	75589
T09E11.5	T09E11.5	I	12356932	12359331	D	Y	MY2	12345894	12346085	12421673	12430881	75589
T09E11.6	T09E11.6	I	12366325	12368609	D	Y	MY2	12345894	12346085	12421673	12430881	75589
T09E11.7	T09E11.7	I	12353496	12356327	D	Y	MY2	12345894	12346085	12421673	12430881	75589
T09E11.8	T09E11.8	I	12350682	12352714	D	Y	MY2	12345894	12346085	12421673	12430881	75589
T09E11.9	T09E11.9	I	12347820	12349809	D	Y	MY2	12345894	12346085	12421673	12430881	75589
T09H2.1	cyp-34A4	V	3950051	3951948	D	Y	JU258	3922016	3922055	3958835	3958879	36781
T10B9.2	cyp-13A5	II	9798200	9800285	D	N	CB4858	9799186	9799307	9799807	9800266	501
T10C6.1	str-232	V	16014482	16015725	A	Y	AB1	16003771	16004293	16024961	16025011	20669
T10C6.2	T10C6.2	V	16012510	16014199	A	Y	AB1	16003771	16004293	16024961	16025011	20669
T10C6.3	srx-43	V	16017944	16019835	A	Y	AB1	16003771	16004293	16024961	16025011	20669
T10C6.4	srx-44	V	16020709	16022625	A	Y	AB1	16003771	16004293	16024961	16025011	20669
T10C6.5	T10C6.5	V	16023324	16024158	A	Y	AB1	16003771	16004293	16024961	16025011	20669
T10C6.6a	T10C6.6	V	16024299	16027418	A	N	AB1	16003771	16004293	16024961	16025011	20669
T10D4.1	T10D4.1	II	3150135	3152570	D	N	KR314	3149842	3150085	3151446	3152296	1362
T10D4.14	T10D4.14	II	3151327	3151779	D	N	KR314	3149842	3150085	3151446	3152296	1362
T10E10.3	T10E10.3	X	6311673	6314546	A	N	CB4853	6312640	6312772	6316856	6317197	4085
T10E10.4	T10E10.4	X	6314498	6322777	A	N	CB4853	6312640	6312772	6316856	6317197	4085
T10H4.10	cyp-34A1	V	15285581	15287901	A	Y	RW7000	15282709	15282820	15399718	15400303	116899
T10H4.11	cyp-34A2	V	15289001	15291062	A	Y	RW7000	15282709	15282820	15399718	15400303	116899
T10H4.12	cpr-3	V	15296826	15298652	A	Y	RW7000	15282709	15282820	15399718	15400303	116899
T10H4.2	str-96	V	15292466	15293941	A	Y	RW7000	15282709	15282820	15399718	15400303	116899
T10H4.9	srx-51	V	15281875	15283538	A	N	RW7000	15282709	15282820	15399718	15400303	116899

T11F1.2	T11F1.2	II	2956060	2958186	D	N	AB1	2954902	2955107	2956099	2956167	993
T11F1.2	T11F1.2	II	2956060	2958186	D	N	JU258	2954902	2955107	2956099	2956209	993
T11F1.2	T11F1.2	II	2956060	2958186	D	N	JU263	2954902	2955107	2956167	2956209	1061
T11F1.2	T11F1.2	II	2956060	2958186	D	N	KR314	2954902	2955107	2956099	2956167	993
T11F1.3	T11F1.3	II	2955477	2955929	D	Y	AB1	2954902	2955107	2956099	2956167	993
T11F1.3	T11F1.3	II	2955477	2955929	D	Y	JU258	2954902	2955107	2956099	2956209	993
T11F1.3	T11F1.3	II	2955477	2955929	D	Y	JU263	2954902	2955107	2956167	2956209	1061
T11F1.3	T11F1.3	II	2955477	2955929	D	Y	KR314	2954902	2955107	2956099	2956167	993
T11F1.8	T11F1.8	II	2948924	2956035	D	N	AB1	2954902	2955107	2956099	2956167	993
T11F1.8	T11F1.8	II	2948924	2956035	D	N	JU258	2954902	2955107	2956099	2956209	993
T11F1.8	T11F1.8	II	2948924	2956035	D	N	JU263	2954902	2955107	2956167	2956209	1061
T11F1.8	T11F1.8	II	2948924	2956035	D	N	KR314	2954902	2955107	2956099	2956167	993
T12B5.1	fbxa-51	III	962291	963513	D	Y	JU258	948244	949229	973819	976540	24591
T12B5.11	fbxa-67	III	949314	950598	D	Y	JU258	948244	949229	973819	976540	24591
T12B5.14	T12B5.14	III	954860	955282	D	Y	JU258	948244	949229	973819	976540	24591
T12B5.15	T12B5.15	III	954282	954639	D	Y	JU258	948244	949229	973819	976540	24591
T12B5.2	fbxa-54	III	956321	957782	D	Y	JU258	948244	949229	973819	976540	24591
T12B5.3	fbxa-10	III	952531	953790	D	Y	JU258	948244	949229	973819	976540	24591
T12B5.4	fbxa-11	III	950869	952042	D	Y	JU258	948244	949229	973819	976540	24591
T13B5.5	lips-11	II	1108847	1110998	D	N	KR314	1108662	1109926	1111973	1113185	2048
T13F3.4	T13F3.4	V	16265070	16265580	D	N	MY2	16265173	16265288	16265446	16266747	159
T15B7.10	T15B7.10	V	6810609	6811652	D	N	CB4856	6806430	6810628	6811365	6813874	738
T15B7.10	T15B7.10	V	6810609	6811652	D	N	JU258	6806430	6810628	6811909	6813874	1282
T15D6.1	T15D6.1	I	12371340	12372637	D	N	CB4856	12370046	12370413	12372095	12372444	1683
T15D6.1	T15D6.1	I	12371340	12372637	D	N	JU258	12368252	12369129	12372570	12374007	3442
T15D6.1	T15D6.1	I	12371340	12372637	D	N	KR314	12368252	12369129	12372095	12374007	2967
T15D6.1	T15D6.1	I	12371340	12372637	D	Y	MY2	12345894	12346085	12421673	12430881	75589
T15D6.10	T15D6.10	I	12394109	12396267	D	Y	MY2	12345894	12346085	12421673	12430881	75589
T15D6.11	T15D6.11	I	12396633	12398281	D	Y	MY2	12345894	12346085	12421673	12430881	75589
T15D6.12	T15D6.12	I	12399312	12401195	D	Y	MY2	12345894	12346085	12421673	12430881	75589
T15D6.2	gly-16	I	12379325	12381022	D	Y	MY2	12345894	12346085	12421673	12430881	75589
T15D6.3	gly-17	I	12376894	12378664	D	Y	MY2	12345894	12346085	12421673	12430881	75589
T15D6.4	T15D6.4	I	12374195	12376639	D	Y	MY2	12345894	12346085	12421673	12430881	75589
T15D6.5	T15D6.5	I	12381798	12383249	D	Y	MY2	12345894	12346085	12421673	12430881	75589
T15D6.6	nhr-77	I	12384267	12385721	D	Y	MY2	12345894	12346085	12421673	12430881	75589
T15D6.7	T15D6.7	I	12385756	12388781	D	Y	MY2	12345894	12346085	12421673	12430881	75589
T15D6.8	T15D6.8	I	12389749	12390968	D	Y	MY2	12345894	12346085	12421673	12430881	75589
T15D6.9	T15D6.9	I	12391998	12393574	D	Y	MY2	12345894	12346085	12421673	12430881	75589
T16A1.1a	math-42	II	2098872	2104150	D	N	CB4853	2103403	2103518	2138338	2138410	34821
T16A1.1a	math-42	II	2098872	2104150	D	N	CB4856	2103344	2103518	2103958	2104354	441
T16A1.1a	math-42	II	2098872	2104150	D	N	CB4858	2103403	2103518	2138338	2138410	34821
T16A1.1a	math-42	II	2098872	2104150	D	Y	JU258	2063261	2063759	2138338	2138578	74580
T16A1.1a	math-42	II	2098872	2104150	D	N	KR314	2068600	2068808	2099883	2102260	31076
T16A1.1a	math-42	II	2098872	2104150	D	N	KR314	2103403	2103518	2138338	2138410	34821
T16A1.2	T16A1.2	II	2095305	2098672	D	N	CB4853	2068600	2068808	2097680	2103041	28873
T16A1.2	T16A1.2	II	2095305	2098672	D	N	CB4858	2068600	2068808	2097680	2103041	28873
T16A1.2	T16A1.2	II	2095305	2098672	D	Y	JU258	2063261	2063759	2138338	2138578	74580
T16A1.2	T16A1.2	II	2095305	2098672	D	Y	KR314	2068600	2068808	2099883	2102260	31076
T16A1.3	T16A1.3	II	2084130	2084892	D	Y	CB4853	2068600	2068808	2097680	2103041	28873
T16A1.3	T16A1.3	II	2084130	2084892	D	N	CB4856	2079421	2081363	2084491	2084641	3129
T16A1.3	T16A1.3	II	2084130	2084892	D	Y	CB4858	2068600	2068808	2097680	2103041	28873
T16A1.3	T16A1.3	II	2084130	2084892	D	Y	JU258	2063261	2063759	2138338	2138578	74580
T16A1.3	T16A1.3	II	2084130	2084892	D	Y	KR314	2068600	2068808	2099883	2102260	31076
T16A1.4	T16A1.4	II	2081558	2082017	D	Y	CB4853	2068600	2068808	2097680	2103041	28873
T16A1.4	T16A1.4	II	2081558	2082017	D	Y	CB4856	2079421	2081363	2084491	2084641	3129
T16A1.4	T16A1.4	II	2081558	2082017	D	Y	CB4858	2068600	2068808	2097680	2103041	28873
T16A1.4	T16A1.4	II	2081558	2082017	D	Y	JU258	2063261	2063759	2138338	2138578	74580
T16A1.4	T16A1.4	II	2081558	2082017	D	N	JU322	2065424	2068458	2081749	2076295	13292
T16A1.4	T16A1.4	II	2081558	2082017	D	Y	KR314	2068600	2068808	2099883	2102260	31076
T16A1.5	T16A1.5	II	2083596	2083827	D	Y	CB4853	2068600	2068808	2097680	2103041	28873

T16A1.5	T16A1.5	II	2083596	2083827	D	Y	CB4856	2079421	2081363	2084491	2084641	3129
T16A1.5	T16A1.5	II	2083596	2083827	D	Y	CB4858	2068600	2068808	2097680	2103041	28873
T16A1.5	T16A1.5	II	2083596	2083827	D	Y	JU258	2063261	2063759	2138338	2138578	74580
T16A1.5	T16A1.5	II	2083596	2083827	D	Y	KR314	2068600	2068808	2099883	2102260	31076
T16A1.7	pqn-66	II	2085409	2090619	D	Y	CB4853	2068600	2068808	2097680	2103041	28873
T16A1.7	pqn-66	II	2085409	2090619	D	Y	CB4858	2068600	2068808	2097680	2103041	28873
T16A1.7	pqn-66	II	2085409	2090619	D	Y	JU258	2063261	2063759	2138338	2138578	74580
T16A1.7	pqn-66	II	2085409	2090619	D	Y	KR314	2068600	2068808	2099883	2102260	31076
T16A1.8	fbxb-37	II	2091744	2093850	D	Y	CB4853	2068600	2068808	2097680	2103041	28873
T16A1.8	fbxb-37	II	2091744	2093850	D	Y	CB4858	2068600	2068808	2097680	2103041	28873
T16A1.8	fbxb-37	II	2091744	2093850	D	Y	JU258	2063261	2063759	2138338	2138578	74580
T16A1.8	fbxb-37	II	2091744	2093850	D	Y	KR314	2068600	2068808	2099883	2102260	31076
T19C9.1	srbc-62	V	17217958	17218972	D	N	JU258	17214831	17217992	17222364	17225924	4373
T19C9.2	srh-112	V	17225054	17226187	D	N	CB4856	17225924	17226138	17261395	17261770	35258
T19C9.3	srh-252	V	17222354	17223373	D	N	JU258	17214831	17217992	17222364	17225924	4373
T19C9.4	srh-109	V	17219221	17220413	D	Y	JU258	17214831	17217992	17222364	17225924	4373
T19C9.5	scl-25	V	17227147	17227929	D	Y	CB4856	17225924	17226138	17261395	17261770	35258
T19C9.8	T19C9.8	V	17238389	17240624	D	Y	CB4856	17225924	17226138	17261395	17261770	35258
T19C9.8	T19C9.8	V	17238389	17240624	D	N	JU258	17229936	17238425	17347568	17348479	109144
T19H5.1	T19H5.1	II	9472611	9474253	D	N	CB4853	9472678	9472767	9472922	9472990	156
T19H5.1	T19H5.1	II	9472611	9474253	D	N	CB4858	9472678	9472767	9472922	9472990	156
T20B12.1	T20B12.1	III	7386154	7391061	A	N	JU258	7386432	7386847	7392985	7393671	6139
T20B12.9	lgc-50	III	7391610	7395208	A	N	JU258	7386432	7386847	7392985	7393671	6139
T20B6.1	T20B6.1	III	2897701	2898361	D	N	CB4854	2893435	2893534	2898174	2899381	4641
T20B6.2	T20B6.2	III	2893423	2897173	D	N	CB4854	2893435	2893534	2898174	2899381	4641
T20C4.1	srj-9	V	3432257	3433656	D	N	JU258	3246578	3247858	3433564	3441667	185707
T20C4.1	srj-9	V	3432257	3433656	D	N	MY2	3248872	3249051	3433136	3433521	184086
T20C7.2	nhr-284	V	3907778	3908366	D	Y	JU258	3906298	3906677	3917411	3917762	10735
T20D4.1	srab-20	V	3428997	3430406	D	Y	CB4856	3423993	3424096	3431378	3432257	7283
T20D4.1	srab-20	V	3428997	3430406	D	Y	JU258	3246578	3247858	3433564	3441667	185707
T20D4.1	srab-20	V	3428997	3430406	D	Y	MY2	3248872	3249051	3433136	3433521	184086
T20D4.10	T20D4.10	V	3400253	3400959	D	Y	CB4856	3319416	3319804	3423047	3423415	103244
T20D4.10	T20D4.10	V	3400253	3400959	D	Y	JU258	3246578	3247858	3433564	3441667	185707
T20D4.10	T20D4.10	V	3400253	3400959	D	Y	MY2	3248872	3249051	3433136	3433521	184086
T20D4.11	T20D4.11	V	3398691	3399387	D	Y	CB4856	3319416	3319804	3423047	3423415	103244
T20D4.11	T20D4.11	V	3398691	3399387	D	Y	JU258	3246578	3247858	3433564	3441667	185707
T20D4.11	T20D4.11	V	3398691	3399387	D	Y	MY2	3248872	3249051	3433136	3433521	184086
T20D4.12	T20D4.12	V	3397073	3397769	D	Y	CB4856	3319416	3319804	3423047	3423415	103244
T20D4.12	T20D4.12	V	3397073	3397769	D	Y	JU258	3246578	3247858	3433564	3441667	185707
T20D4.12	T20D4.12	V	3397073	3397769	D	Y	MY2	3248872	3249051	3433136	3433521	184086
T20D4.13	T20D4.13	V	3391681	3393292	D	Y	CB4856	3319416	3319804	3423047	3423415	103244
T20D4.13	T20D4.13	V	3391681	3393292	D	Y	JU258	3246578	3247858	3433564	3441667	185707
T20D4.13	T20D4.13	V	3391681	3393292	D	Y	MY2	3248872	3249051	3433136	3433521	184086
T20D4.15	T20D4.15	V	3393474	3394003	D	Y	CB4856	3319416	3319804	3423047	3423415	103244
T20D4.15	T20D4.15	V	3393474	3394003	D	Y	JU258	3246578	3247858	3433564	3441667	185707
T20D4.15	T20D4.15	V	3393474	3394003	D	Y	MY2	3248872	3249051	3433136	3433521	184086
T20D4.16	T20D4.16	V	3394387	3394686	D	Y	CB4856	3319416	3319804	3423047	3423415	103244
T20D4.16	T20D4.16	V	3394387	3394686	D	Y	JU258	3246578	3247858	3433564	3441667	185707
T20D4.16	T20D4.16	V	3394387	3394686	D	Y	MY2	3248872	3249051	3433136	3433521	184086
T20D4.17	T20D4.17	V	3395536	3395964	D	Y	CB4856	3319416	3319804	3423047	3423415	103244
T20D4.17	T20D4.17	V	3395536	3395964	D	Y	JU258	3246578	3247858	3433564	3441667	185707
T20D4.17	T20D4.17	V	3395536	3395964	D	Y	MY2	3248872	3249051	3433136	3433521	184086
T20D4.18	srab-21	V	3422947	3424439	D	N	CB4856	3319416	3319804	3423047	3423415	103244
T20D4.18	srab-21	V	3422947	3424439	D	N	CB4856	3423993	3424096	3431378	3432257	7283
T20D4.18	srab-21	V	3422947	3424439	D	Y	JU258	3246578	3247858	3433564	3441667	185707
T20D4.18	srab-21	V	3422947	3424439	D	Y	MY2	3248872	3249051	3433136	3433521	184086
T20D4.19	T20D4.19	V	3430667	3431444	D	N	CB4856	3423993	3424096	3431378	3432257	7283
T20D4.19	T20D4.19	V	3430667	3431444	D	Y	JU258	3246578	3247858	3433564	3441667	185707
T20D4.19	T20D4.19	V	3430667	3431444	D	Y	MY2	3248872	3249051	3433136	3433521	184086
T20D4.2	srab-22	V	3426374	3427731	D	Y	CB4856	3423993	3424096	3431378	3432257	7283

T20D4.2	srab-22	V	3426374	3427731	D	Y	JU258	3246578	3247858	3433564	3441667	185707
T20D4.2	srab-22	V	3426374	3427731	D	Y	MY2	3248872	3249051	3433136	3433521	184086
T20D4.20	T20D4.20	V	3401643	3402354	D	Y	CB4856	3319416	3319804	3423047	3423415	103244
T20D4.20	T20D4.20	V	3401643	3402354	D	Y	JU258	3246578	3247858	3433564	3441667	185707
T20D4.20	T20D4.20	V	3401643	3402354	D	Y	MY2	3248872	3249051	3433136	3433521	184086
T20D4.3	T20D4.3	V	3418261	3422731	D	Y	CB4856	3319416	3319804	3423047	3423415	103244
T20D4.3	T20D4.3	V	3418261	3422731	D	Y	JU258	3246578	3247858	3433564	3441667	185707
T20D4.3	T20D4.3	V	3418261	3422731	D	Y	MY2	3248872	3249051	3433136	3433521	184086
T20D4.4	T20D4.4	V	3415991	3417750	D	Y	CB4856	3319416	3319804	3423047	3423415	103244
T20D4.4	T20D4.4	V	3415991	3417750	D	Y	JU258	3246578	3247858	3433564	3441667	185707
T20D4.4	T20D4.4	V	3415991	3417750	D	Y	MY2	3248872	3249051	3433136	3433521	184086
T20D4.5	T20D4.5	V	3413342	3415814	D	Y	CB4856	3319416	3319804	3423047	3423415	103244
T20D4.5	T20D4.5	V	3413342	3415814	D	Y	JU258	3246578	3247858	3433564	3441667	185707
T20D4.5	T20D4.5	V	3413342	3415814	D	Y	MY2	3248872	3249051	3433136	3433521	184086
T20D4.6	T20D4.6	V	3410518	3412835	D	Y	CB4856	3319416	3319804	3423047	3423415	103244
T20D4.6	T20D4.6	V	3410518	3412835	D	Y	JU258	3246578	3247858	3433564	3441667	185707
T20D4.6	T20D4.6	V	3410518	3412835	D	Y	MY2	3248872	3249051	3433136	3433521	184086
T20D4.7	T20D4.7	V	3408868	3409517	D	Y	CB4856	3319416	3319804	3423047	3423415	103244
T20D4.7	T20D4.7	V	3408868	3409517	D	Y	JU258	3246578	3247858	3433564	3441667	185707
T20D4.7	T20D4.7	V	3408868	3409517	D	Y	MY2	3248872	3249051	3433136	3433521	184086
T20D4.8	T20D4.8	V	3405707	3407891	D	Y	CB4856	3319416	3319804	3423047	3423415	103244
T20D4.8	T20D4.8	V	3405707	3407891	D	Y	JU258	3246578	3247858	3433564	3441667	185707
T20D4.8	T20D4.8	V	3405707	3407891	D	Y	MY2	3248872	3249051	3433136	3433521	184086
T20D4.9	T20D4.9	V	3402909	3405193	D	Y	CB4856	3319416	3319804	3423047	3423415	103244
T20D4.9	T20D4.9	V	3402909	3405193	D	Y	JU258	3246578	3247858	3433564	3441667	185707
T20D4.9	T20D4.9	V	3402909	3405193	D	Y	MY2	3248872	3249051	3433136	3433521	184086
T20G5.1	chc-1	III	10203715	10209598	A	N	JU263	10207143	10207299	10209897	10210043	2599
T20G5.9	T20G5.9	III	10209742	10210875	A	N	JU263	10207143	10207299	10209897	10210043	2599
T20H9.2	fbxa-43	III	2257541	2258928	D	N	CB4853	2257622	2257884	2258071	2263236	188
T20H9.2	fbxa-43	III	2257541	2258928	D	N	CB4858	2257622	2257884	2258071	2263236	188
T21B4.9	srh-70	II	12516668	12517773	D	N	KR314	12517213	12517252	12517714	12519080	463
T21E8.2	pgp-7	X	10864896	10870164	D	N	MY2	10864706	10866265	10871113	10872634	4849
T23D5.1	str-27	V	15733997	15735794	D	Y	JU258	15730588	15730913	15760733	15761000	29821
T23D5.10	str-6	V	15748971	15750204	D	Y	JU258	15730588	15730913	15760733	15761000	29821
T23D5.11	str-8	V	15755005	15756368	D	Y	JU258	15730588	15730913	15760733	15761000	29821
T23D5.12	str-5	V	15753215	15754406	D	Y	JU258	15730588	15730913	15760733	15761000	29821
T23D5.2	str-38	V	15730753	15732283	D	N	JU258	15730588	15730913	15760733	15761000	29821
T23D5.3	T23D5.3	V	15732837	15733642	D	Y	JU258	15730588	15730913	15760733	15761000	29821
T23D5.6	str-18	V	15740501	15741925	D	Y	JU258	15730588	15730913	15760733	15761000	29821
T23D5.7	str-19	V	15743234	15744431	D	Y	JU258	15730588	15730913	15760733	15761000	29821
T23D5.8	T23D5.8	V	15747454	15748372	D	Y	JU258	15730588	15730913	15760733	15761000	29821
T23D5.9	str-43	V	15750906	15752123	D	Y	JU258	15730588	15730913	15760733	15761000	29821
T23F1.3	T23F1.3	V	15456041	15457420	D	N	CB4856	15451443	15455947	15456921	15457014	975
T24E12.4	srx-111	II	3757793	3759246	D	N	CB4854	3758021	3758062	3758745	3760463	684
T24E12.4	srx-111	II	3757793	3759246	D	Y	JU258	3748451	3748499	3759197	3760463	10699
T24E12.4	srx-111	II	3757793	3759246	D	N	JU322	3748451	3748499	3759029	3760463	10531
T24E12.4	srx-111	II	3757793	3759246	D	N	KR314	3748451	3748499	3759029	3759158	10531
T24E12.4	srx-111	II	3757793	3759246	D	N	MY2	3748358	3748499	3759029	3760463	10531
T24E12.5	T24E12.5	II	3753915	3757350	D	N	AB1	3748451	3748499	3757270	3757743	8772
T24E12.5	T24E12.5	II	3753915	3757350	D	N	CB4854	3748451	3748499	3757270	3757743	8772
T24E12.5	T24E12.5	II	3753915	3757350	D	N	CB4856	3748451	3748582	3757270	3757743	8689
T24E12.5	T24E12.5	II	3753915	3757350	D	Y	JU258	3748451	3748499	3759197	3760463	10699
T24E12.5	T24E12.5	II	3753915	3757350	D	N	JU263	3748538	3748582	3757270	3757743	8689
T24E12.5	T24E12.5	II	3753915	3757350	D	Y	JU322	3748451	3748499	3759029	3760463	10531
T24E12.5	T24E12.5	II	3753915	3757350	D	Y	KR314	3748451	3748499	3759029	3759158	10531
T24E12.5	T24E12.5	II	3753915	3757350	D	Y	MY2	3748358	3748499	3759029	3760463	10531
T26E3.1	clec-103	I	12683239	12684327	D	N	CB4856	12683239	12683365	12684210	12686131	846
T26E3.8	T26E3.8	I	12657820	12659366	D	N	CB4856	12655835	12657925	12659097	12661346	1173
T26H2.1	fbxb-1	V	19240318	19241588	D	N	CB4856	19240392	19240532	19244840	19247309	4309
T26H2.2	fbxb-115	V	19238385	19239813	D	N	AB1	19236277	19236316	19238797	19239126	2482

T26H2.2	fbxb-115	V	19238385	19239813	D	N	CB4853	19236277	19236316	19238797	19239126	2482
T26H2.2	fbxb-115	V	19238385	19239813	D	N	CB4854	19236277	19236316	19238797	19239126	2482
T26H2.2	fbxb-115	V	19238385	19239813	D	N	CB4856	19237263	19238432	19239757	19240268	1326
T26H2.2	fbxb-115	V	19238385	19239813	D	N	CB4858	19236277	19236316	19238797	19239126	2482
T26H2.2	fbxb-115	V	19238385	19239813	D	N	JU258	19236277	19236316	19238797	19239126	2482
T26H2.2	fbxb-115	V	19238385	19239813	D	N	JU263	19236277	19236316	19238797	19239126	2482
T26H2.2	fbxb-115	V	19238385	19239813	D	N	JU322	19236277	19236316	19238797	19239126	2482
T26H2.2	fbxb-115	V	19238385	19239813	D	N	KR314	19236277	19236316	19238797	19239126	2482
T26H2.2	fbxb-115	V	19238385	19239813	D	N	MY2	19236277	19236316	19238797	19239126	2482
T26H2.3	fbxb-2	V	19236055	19237262	D	N	AB1	19236277	19236316	19238797	19239126	2482
T26H2.3	fbxb-2	V	19236055	19237262	D	N	CB4853	19236277	19236316	19238797	19239126	2482
T26H2.3	fbxb-2	V	19236055	19237262	D	N	CB4854	19236277	19236316	19238797	19239126	2482
T26H2.3	fbxb-2	V	19236055	19237262	A	Y	CB4856	19228374	19230781	19237263	19238432	6483
T26H2.3	fbxb-2	V	19236055	19237262	D	N	CB4858	19236277	19236316	19238797	19239126	2482
T26H2.3	fbxb-2	V	19236055	19237262	D	N	JU258	19236277	19236316	19238797	19239126	2482
T26H2.3	fbxb-2	V	19236055	19237262	D	N	JU263	19236277	19236316	19238797	19239126	2482
T26H2.3	fbxb-2	V	19236055	19237262	D	N	JU322	19236277	19236316	19238797	19239126	2482
T26H2.3	fbxb-2	V	19236055	19237262	D	N	KR314	19236277	19236316	19238797	19239126	2482
T26H2.3	fbxb-2	V	19236055	19237262	D	N	MY2	19236277	19236316	19238797	19239126	2482
T26H2.4	fbxb-116	V	19233921	19235549	A	Y	CB4856	19228374	19230781	19237263	19238432	6483
T26H2.5	T26H2.5	V	19230746	19231984	A	N	CB4856	19228374	19230781	19237263	19238432	6483
T26H8.2	srx-48	V	15302347	15303701	A	Y	RW7000	15282709	15282820	15399718	15400303	116899
T26H8.4	T26H8.4	V	15305778	15306821	A	Y	RW7000	15282709	15282820	15399718	15400303	116899
T26H8.5	T26H8.5	V	15304583	15305578	A	Y	RW7000	15282709	15282820	15399718	15400303	116899
T27A1.1	T27A1.1	II	503228	506013	A	N	CB4853	503455	505645	509295	516098	3651
T27A1.1	T27A1.1	II	503228	506013	A	N	CB4858	503455	505645	509295	516098	3651
T27A1.7	srh-105	II	508244	509325	A	N	CB4853	503455	505645	509295	516098	3651
T27A1.7	srh-105	II	508244	509325	A	N	CB4858	503455	505645	509295	516098	3651
T27A8.5	T27A8.5	X	16063778	16066795	D	N	CB4853	16064112	16064229	16064368	16064904	140
T27C5.1	srh-120	V	17395642	17398002	D	Y	KR314	17360012	17371630	17489726	17490760	118097
T27C5.10	T27C5.10	V	17429669	17430134	D	Y	JU258	17417977	17418058	17499690	17506165	81633
T27C5.10	T27C5.10	V	17429669	17430134	D	Y	KR314	17360012	17371630	17489726	17490760	118097
T27C5.12	T27C5.12	V	17415246	17416711	D	Y	CB4856	17413671	17414106	17418524	17429281	4419
T27C5.12	T27C5.12	V	17415246	17416711	D	Y	KR314	17360012	17371630	17489726	17490760	118097
T27C5.2	srx-53	V	17400091	17401742	D	Y	KR314	17360012	17371630	17489726	17490760	118097
T27C5.5	srh-132	V	17410223	17411673	D	Y	KR314	17360012	17371630	17489726	17490760	118097
T27C5.7	clec-244	V	17413514	17414717	D	N	CB4856	17413671	17414106	17418524	17429281	4419
T27C5.7	clec-244	V	17413514	17414717	D	Y	KR314	17360012	17371630	17489726	17490760	118097
T27C5.8	T27C5.8	V	17417125	17418359	D	Y	CB4856	17413671	17414106	17418524	17429281	4419
T27C5.8	T27C5.8	V	17417125	17418359	D	N	JU258	17417977	17418058	17499690	17506165	81633
T27C5.8	T27C5.8	V	17417125	17418359	D	Y	KR314	17360012	17371630	17489726	17490760	118097
T27E7.9	T27E7.9	IV	14546899	14549351	D	N	JU258	14547120	14547780	14550230	14551095	2451
T27E7.9	T27E7.9	IV	14546899	14549351	D	N	KR314	14547308	14547780	14549271	14549792	1492
T28A11.1	str-64	V	3283322	3284527	D	Y	CB4856	3276151	3276987	3286204	3286430	9218
T28A11.1	str-64	V	3283322	3284527	D	Y	JU258	3246578	3247858	3433564	3441667	185707
T28A11.1	str-64	V	3283322	3284527	D	Y	MY2	3248872	3249051	3433136	3433521	184086
T28A11.13	T28A11.13	V	3249742	3250204	D	Y	JU258	3246578	3247858	3433564	3441667	185707
T28A11.13	T28A11.13	V	3249742	3250204	D	Y	MY2	3248872	3249051	3433136	3433521	184086
T28A11.15	srt-63	V	3254601	3256150	D	Y	JU258	3246578	3247858	3433564	3441667	185707
T28A11.15	srt-63	V	3254601	3256150	D	Y	MY2	3248872	3249051	3433136	3433521	184086
T28A11.16	T28A11.16	V	3258519	3259214	D	Y	JU258	3246578	3247858	3433564	3441667	185707
T28A11.16	T28A11.16	V	3258519	3259214	D	Y	MY2	3248872	3249051	3433136	3433521	184086
T28A11.17	T28A11.17	V	3263481	3266253	D	Y	JU258	3246578	3247858	3433564	3441667	185707
T28A11.17	T28A11.17	V	3263481	3266253	D	Y	MY2	3248872	3249051	3433136	3433521	184086
T28A11.18	T28A11.18	V	3266827	3268049	D	Y	JU258	3246578	3247858	3433564	3441667	185707
T28A11.18	T28A11.18	V	3266827	3268049	D	Y	MY2	3248872	3249051	3433136	3433521	184086
T28A11.19	T28A11.19	V	3268808	3269504	D	Y	JU258	3246578	3247858	3433564	3441667	185707
T28A11.19	T28A11.19	V	3268808	3269504	D	Y	MY2	3248872	3249051	3433136	3433521	184086
T28A11.20	T28A11.20	V	3271394	3273078	D	Y	JU258	3246578	3247858	3433564	3441667	185707
T28A11.20	T28A11.20	V	3271394	3273078	D	Y	MY2	3248872	3249051	3433136	3433521	184086

T28A11.21	fbxa-64	V	3277085	3278129	D	Y	CB4856	3276151	3276987	3286204	3286430	9218
T28A11.21	fbxa-64	V	3277085	3278129	D	Y	JU258	3246578	3247858	3433564	3441667	185707
T28A11.21	fbxa-64	V	3277085	3278129	D	Y	MY2	3248872	3249051	3433136	3433521	184086
T28A11.22	T28A11.22	V	3257584	3258239	D	Y	JU258	3246578	3247858	3433564	3441667	185707
T28A11.22	T28A11.22	V	3257584	3258239	D	Y	MY2	3248872	3249051	3433136	3433521	184086
T28A11.2a	T28A11.2	V	3280377	3282641	D	Y	CB4856	3276151	3276987	3286204	3286430	9218
T28A11.2a	T28A11.2	V	3280377	3282641	D	Y	JU258	3246578	3247858	3433564	3441667	185707
T28A11.2a	T28A11.2	V	3280377	3282641	D	Y	MY2	3248872	3249051	3433136	3433521	184086
T28A11.2b	T28A11.2	V	3280377	3282641	D	Y	JU258	3246578	3247858	3433564	3441667	185707
T28A11.3	T28A11.3	V	3275381	3276109	D	Y	JU258	3246578	3247858	3433564	3441667	185707
T28A11.3	T28A11.3	V	3275381	3276109	D	Y	MY2	3248872	3249051	3433136	3433521	184086
T28A11.4	T28A11.4	V	3274053	3274373	D	Y	JU258	3246578	3247858	3433564	3441667	185707
T28A11.4	T28A11.4	V	3274053	3274373	D	Y	MY2	3248872	3249051	3433136	3433521	184086
T28A11.5	T28A11.5	V	3270552	3271239	D	Y	JU258	3246578	3247858	3433564	3441667	185707
T28A11.5	T28A11.5	V	3270552	3271239	D	Y	MY2	3248872	3249051	3433136	3433521	184086
T28A11.6	T28A11.6	V	3262570	3263232	D	Y	JU258	3246578	3247858	3433564	3441667	185707
T28A11.6	T28A11.6	V	3262570	3263232	D	Y	MY2	3248872	3249051	3433136	3433521	184086
T28A11.7	srbc-5	V	3260374	3262081	D	Y	JU258	3246578	3247858	3433564	3441667	185707
T28A11.7	srbc-5	V	3260374	3262081	D	Y	MY2	3248872	3249051	3433136	3433521	184086
T28A11.9	srj-8	V	3247814	3249139	D	N	JU258	3246578	3247858	3433564	3441667	185707
T28A11.9	srj-8	V	3247814	3249139	D	N	MY2	3248872	3249051	3433136	3433521	184086
T28B4.2	T28B4.2	X	6581961	6583546	D	N	CB4853	6569396	6581855	6583376	6583496	1522
VY10G11R.1	VY10G11R.1	IV	16469539	16470840	D	N	CB4856	16469184	16469738	16470006	16470072	269
W02A11.5	bath-34	I	12750025	12752056	D	N	CB4856	12750598	12751464	12758513	12758603	7050
W02A11.8	bath-35	I	12758513	12759458	D	N	CB4856	12750598	12751464	12758513	12758603	7050
W02A11.8	bath-35	I	12758513	12759458	D	N	CB4856	12758603	12758825	12759375	12759745	551
W02A11.8	bath-35	I	12758513	12759458	D	N	JU263	12758603	12758660	12759330	12759375	671
W02A11.8	bath-35	I	12758513	12759458	D	N	KR314	12758603	12758660	12759217	12759375	558
W02B3.5	W02B3.5	III	686231	687079	D	N	JU322	686899	687074	687377	689879	304
W03C9.5	W03C9.5	II	11966962	11967448	D	N	JU258	11966749	11967298	11967437	11968583	140
W03H1.2	elc-2	X	1777351	1778142	D	N	CB4856	1747004	1752189	1778099	1781395	25911
W03H9.1	W03H9.1	II	14147245	14150034	D	N	CB4858	14143881	14147562	14147688	14147761	127
W03H9.1	W03H9.1	II	14147245	14150034	D	N	KR314	14143881	14147562	14147688	14147761	127
W04E12.5	W04E12.5	V	19741541	19743888	D	N	CB4853	19743041	19743707	19746576	19747740	2870
W04E12.5	W04E12.5	V	19741541	19743888	D	N	CB4854	19743041	19743707	19746576	19748160	2870
W04E12.5	W04E12.5	V	19741541	19743888	D	N	CB4858	19743041	19743707	19746576	19747740	2870
W04E12.5	W04E12.5	V	19741541	19743888	D	N	JU258	19743041	19743707	19746576	19747740	2870
W04E12.5	W04E12.5	V	19741541	19743888	D	N	JU263	19742950	19743707	19746576	19747740	2870
W04E12.5	W04E12.5	V	19741541	19743888	D	N	JU322	19743041	19743707	19746576	19747740	2870
W04E12.5	W04E12.5	V	19741541	19743888	D	N	KR314	19743041	19743707	19746576	19747740	2870
W04E12.6	clec-49	V	19744376	19746601	D	N	CB4853	19743041	19743707	19746576	19747740	2870
W04E12.6	clec-49	V	19744376	19746601	D	N	CB4854	19743041	19743707	19746576	19748160	2870
W04E12.6	clec-49	V	19744376	19746601	D	N	CB4858	19743041	19743707	19746576	19747740	2870
W04E12.6	clec-49	V	19744376	19746601	D	N	JU258	19743041	19743707	19746576	19747740	2870
W04E12.6	clec-49	V	19744376	19746601	D	N	JU263	19742950	19743707	19746576	19747740	2870
W04E12.6	clec-49	V	19744376	19746601	D	N	JU322	19743041	19743707	19746576	19747740	2870
W04E12.6	clec-49	V	19744376	19746601	D	N	KR314	19743041	19743707	19746576	19747740	2870
W04G5.5	W04G5.5	I	11634603	11636832	D	N	JU258	11634791	11635184	11635773	11635999	590
W04G5.5	W04G5.5	I	11634603	11636832	D	N	JU263	11634791	11635184	11635671	11635773	488
W04G5.7	W04G5.7	I	11652779	11654244	D	N	MY2	11650104	11650201	11652919	11653030	2719
W04G5.8	W04G5.8	I	11649972	11651815	D	N	MY2	11650104	11650201	11652919	11653030	2719
W05G11.4	W05G11.4	III	35454	39461	D	N	CB4854	37830	38019	38116	38165	98
W05G11.5	btb-5	III	59371	61144	D	N	CB4856	53510	60047	61059	61748	1013
W05G11.5	btb-5	III	59371	61144	D	N	MY2	53510	60047	61059	61748	1013
W06B4.3	W06B4.3	II	4461421	4467886	A	N	CB4853	4466969	4467008	4467668	4468173	661
W06B4.3	W06B4.3	II	4461421	4467886	A	N	CB4858	4466858	4466914	4467668	4468173	755
W08D2.8	kin-21	IV	9832909	9834796	D	N	CB4856	9830400	9832966	9833145	9833223	180
W08F4.2	fbxb-34	II	587931	590099	D	N	KR314	588506	589290	589452	589555	163
Y102A5C.1	fbxa-206	V	16917231	16918447	D	Y	CB4856	16912457	16912533	16921721	16922242	9189
Y102A5C.2	Y102A5C.2	V	16918779	16919516	D	Y	CB4856	16912457	16912533	16921721	16922242	9189

Y105C5B.12a	Y105C5B.12	IV	15942222	15944415	D	N	JU258	15941399	15944081	15947436	15949714	3356
Y105C5B.13	skr-10	IV	15946748	15947399	D	Y	JU258	15941399	15944081	15947436	15949714	3356
Y110A2AL.1	Y110A2AL.1	II	2875104	2876616	D	N	JU258	2873413	2873992	2875945	2876493	1954
Y110A2AL.1	Y110A2AL.1	II	2875104	2876616	D	N	MY2	2873413	2873992	2876560	2878983	2569
Y110A2AL.3	Y110A2AL.3	II	2867073	2867778	D	N	AB1	2867097	2867381	2867740	2867892	360
Y110A2AL.4a	Y110A2AL.4	II	2839303	2840385	D	N	CB4853	2836618	2839488	2840215	2841051	728
Y110A2AL.4a	Y110A2AL.4	II	2839303	2840385	D	N	CB4858	2836618	2839488	2840215	2841051	728
Y110A2AL.4a	Y110A2AL.4	II	2839303	2840385	D	Y	JU258	2836458	2836618	2843138	2845475	6521
Y110A2AL.4a	Y110A2AL.4	II	2839303	2840385	D	Y	JU263	2836537	2836618	2843138	2845475	6521
Y110A2AL.4a	Y110A2AL.4	II	2839303	2840385	D	N	KR314	2836618	2839488	2840215	2841051	728
Y110A2AL.6	Y110A2AL.6	II	2841051	2841628	D	Y	JU258	2836458	2836618	2843138	2845475	6521
Y110A2AL.6	Y110A2AL.6	II	2841051	2841628	D	Y	JU263	2836537	2836618	2843138	2845475	6521
Y110A2AL.7	Y110A2AL.7	II	2842607	2843187	D	Y	JU258	2836458	2836618	2843138	2845475	6521
Y110A2AL.7	Y110A2AL.7	II	2842607	2843187	D	Y	JU263	2836537	2836618	2843138	2845475	6521
Y113G7A.12	Y113G7A.12	V	20139635	20142193	D	Y	AB1	20133405	20133764	20151489	20153948	17726
Y113G7A.12	Y113G7A.12	V	20139635	20142193	D	Y	CB4856	20133405	20133764	20156235	20156347	22472
Y113G7A.12	Y113G7A.12	V	20139635	20142193	D	Y	MY2	20133405	20133764	20151489	20154392	17726
Y113G7A.13	Y113G7A.13	V	20143588	20144075	D	Y	AB1	20133405	20133764	20151489	20153948	17726
Y113G7A.13	Y113G7A.13	V	20143588	20144075	D	Y	CB4856	20133405	20133764	20156235	20156347	22472
Y113G7A.13	Y113G7A.13	V	20143588	20144075	D	Y	MY2	20133405	20133764	20151489	20154392	17726
Y113G7A.14	Y113G7A.14	V	20131360	20133831	D	N	AB1	20133405	20133764	20151489	20153948	17726
Y113G7A.14	Y113G7A.14	V	20131360	20133831	D	N	CB4856	20133405	20133764	20156235	20156347	22472
Y113G7A.14	Y113G7A.14	V	20131360	20133831	D	N	MY2	20133405	20133764	20151489	20154392	17726
Y113G7B.11	Y113G7B.11	V	20209271	20210684	D	N	AB1	20199742	20202842	20209417	20209470	6576
Y113G7B.11	Y113G7B.11	V	20209271	20210684	D	N	CB4856	20201357	20204354	20209417	20209470	5064
Y113G7B.11	Y113G7B.11	V	20209271	20210684	D	N	MY2	20198012	20199703	20209417	20210875	9715
Y113G7B.12	Y113G7B.12	V	20204189	20208167	D	Y	AB1	20199742	20202842	20209417	20209470	6576
Y113G7B.12	Y113G7B.12	V	20204189	20208167	D	N	CB4853	20199163	20199821	20208038	20209470	8218
Y113G7B.12	Y113G7B.12	V	20204189	20208167	D	N	CB4854	20199287	20202954	20207967	20209417	5014
Y113G7B.12	Y113G7B.12	V	20204189	20208167	D	N	CB4856	20201357	20204354	20209417	20209470	5064
Y113G7B.12	Y113G7B.12	V	20204189	20208167	D	Y	CB4858	20199163	20199821	20208172	20209417	8352
Y113G7B.12	Y113G7B.12	V	20204189	20208167	D	Y	JU258	20198012	20199203	20208172	20209470	8970
Y113G7B.12	Y113G7B.12	V	20204189	20208167	D	Y	JU263	20199782	20202954	20208172	20209417	5219
Y113G7B.12	Y113G7B.12	V	20204189	20208167	D	Y	JU322	20198086	20199821	20208172	20209417	8352
Y113G7B.12	Y113G7B.12	V	20204189	20208167	D	Y	KR314	20199203	20199287	20208172	20209417	8886
Y113G7B.12	Y113G7B.12	V	20204189	20208167	D	Y	MY2	20198012	20199703	20209417	20210875	9715
Y113G7B.14	Y113G7B.14	V	20222223	20224217	D	Y	AB1	20219626	20220579	20224266	20229051	3688
Y113G7B.14	Y113G7B.14	V	20222223	20224217	D	Y	CB4853	20214424	20220767	20224266	20228945	3500
Y113G7B.14	Y113G7B.14	V	20222223	20224217	D	Y	CB4854	20214424	20220767	20224266	20228945	3500
Y113G7B.14	Y113G7B.14	V	20222223	20224217	D	Y	CB4856	20214424	20217225	20224266	20228945	7042
Y113G7B.14	Y113G7B.14	V	20222223	20224217	D	Y	CB4858	20214424	20220579	20224266	20228945	3688
Y113G7B.14	Y113G7B.14	V	20222223	20224217	D	Y	JU258	20219626	20220767	20227256	20228945	6490
Y113G7B.14	Y113G7B.14	V	20222223	20224217	D	Y	JU263	20219626	20220579	20224266	20228945	3688
Y113G7B.14	Y113G7B.14	V	20222223	20224217	D	Y	JU322	20214424	20220767	20227256	20228945	6490
Y113G7B.14	Y113G7B.14	V	20222223	20224217	D	Y	KR314	20214424	20217225	20224266	20228945	7042
Y113G7B.15	Y113G7B.15	V	20224354	20229129	D	N	JU258	20219626	20220767	20227256	20228945	6490
Y113G7B.15	Y113G7B.15	V	20224354	20229129	D	N	JU322	20214424	20220767	20227256	20228945	6490
Y113G7B.1a	fbxa-116	V	20183098	20184270	D	N	CB4856	20182007	20183240	20186925	20187513	3686
Y113G7B.3	fbxa-115	V	20185972	20187506	D	N	CB4856	20182007	20183240	20186925	20187513	3686
Y113G7B.6	fbxa-113	V	20198810	20199891	D	N	CB4853	20199163	20199821	20208038	20209470	8218
Y113G7B.6	fbxa-113	V	20198810	20199891	D	N	CB4858	20199163	20199821	20208172	20209417	8352
Y113G7B.6	fbxa-113	V	20198810	20199891	D	N	JU258	20198012	20199203	20208172	20209470	8970
Y113G7B.6	fbxa-113	V	20198810	20199891	D	N	JU322	20198086	20199821	20208172	20209417	8352
Y113G7B.6	fbxa-113	V	20198810	20199891	D	N	KR314	20199203	20199287	20208172	20209417	8886
Y113G7B.6	fbxa-113	V	20198810	20199891	D	N	MY2	20198012	20199703	20209417	20210875	9715
Y113G7B.7	fbxa-114	V	20197038	20198147	A	N	CB4853	20197147	20197299	20198086	20198485	788
Y113G7B.8	fbxb-59	V	20199956	20201568	D	Y	CB4853	20199163	20199821	20208038	20209470	8218
Y113G7B.8	fbxb-59	V	20199956	20201568	D	Y	CB4858	20199163	20199821	20208172	20209417	8352
Y113G7B.8	fbxb-59	V	20199956	20201568	D	Y	JU258	20198012	20199203	20208172	20209470	8970
Y113G7B.8	fbxb-59	V	20199956	20201568	D	Y	JU322	20198086	20199821	20208172	20209417	8352

Y113G7B.8	fbxb-59	V	20199956	20201568	D	Y	KR314	20199203	20199287	20208172	20209417	8886
Y113G7B.8	fbxb-59	V	20199956	20201568	D	Y	MY2	20198012	20199703	20209417	20210875	9715
Y113G7B.9	srbc-34	V	20202058	20203295	D	N	AB1	20199742	20202842	20209417	20209470	6576
Y113G7B.9	srbc-34	V	20202058	20203295	D	Y	CB4853	20199163	20199821	20208038	20209470	8218
Y113G7B.9	srbc-34	V	20202058	20203295	D	N	CB4854	20199287	20202954	20207967	20209417	5014
Y113G7B.9	srbc-34	V	20202058	20203295	D	Y	CB4858	20199163	20199821	20208172	20209417	8352
Y113G7B.9	srbc-34	V	20202058	20203295	D	Y	JU258	20198012	20199203	20208172	20209470	8970
Y113G7B.9	srbc-34	V	20202058	20203295	D	N	JU263	20199782	20202954	20208172	20209417	5219
Y113G7B.9	srbc-34	V	20202058	20203295	D	Y	JU322	20198086	20199821	20208172	20209417	8352
Y113G7B.9	srbc-34	V	20202058	20203295	D	Y	KR314	20199203	20199287	20208172	20209417	8886
Y113G7B.9	srbc-34	V	20202058	20203295	D	Y	MY2	20198012	20199703	20209417	20210875	9715
Y116A8A.7	Y116A8A.7	IV	16830167	16830718	D	Y	KR314	16827992	16829881	16832313	16832365	2433
Y116A8A.8	clec-194	IV	16831779	16832943	D	N	KR314	16827992	16829881	16832313	16832365	2433
Y116A8C.1	Y116A8C.1	IV	16900209	16902479	D	Y	JU258	16894642	16896609	16903176	16916026	6568
Y116A8C.21	clec-195	IV	17043369	17045748	A	N	KR314	17043517	17044193	17048260	17053640	4068
Y116A8C.22	Y116A8C.22	IV	17046427	17048316	A	N	KR314	17043517	17044193	17048260	17053640	4068
Y116A8C.40	Y116A8C.40	IV	17144311	17150927	D	N	JU258	17131081	17134566	17145165	17146720	10600
Y116F11B.11	Y116F11B.11	V	19873609	19875414	D	N	CB4856	19874215	19874266	19874840	19874899	575
Y116F11B.5	srw-38	V	19831830	19834730	A	N	MY2	19825429	19833649	19846181	19849102	12533
Y116F11B.6	Y116F11B.6	V	19837113	19841365	A	Y	MY2	19825429	19833649	19846181	19849102	12533
Y116F11B.7	Y116F11B.7	V	19843971	19846281	D	N	CB4853	19844745	19844874	19846181	19849102	1308
Y116F11B.7	Y116F11B.7	V	19843971	19846281	D	N	CB4854	19844745	19844874	19846181	19849102	1308
Y116F11B.7	Y116F11B.7	V	19843971	19846281	D	N	CB4856	19844011	19844267	19852445	19853040	8179
Y116F11B.7	Y116F11B.7	V	19843971	19846281	D	N	CB4858	19844745	19844874	19846181	19849102	1308
Y116F11B.7	Y116F11B.7	V	19843971	19846281	D	N	JU258	19844745	19844874	19846181	19849102	1308
Y116F11B.7	Y116F11B.7	V	19843971	19846281	D	N	JU263	19844745	19844874	19846181	19849102	1308
Y116F11B.7	Y116F11B.7	V	19843971	19846281	D	N	JU322	19844485	19844874	19846181	19849102	1308
Y116F11B.7	Y116F11B.7	V	19843971	19846281	D	N	KR314	19844485	19844745	19846181	19849102	1437
Y116F11B.7	Y116F11B.7	V	19843971	19846281	A	N	MY2	19825429	19833649	19846181	19849102	12533
Y116F11B.9a	Y116F11B.9	V	19850562	19863371	D	N	CB4856	19844011	19844267	19852445	19853040	8179
Y119D3A.1	fbxa-75	III	1303704	1305451	D	Y	JU258	1293124	1296291	1307017	1307077	10727
Y119D3A.1	fbxa-75	III	1303704	1305451	D	N	JU322	1299327	1303600	1305396	1305794	1797
Y119D3A.1	fbxa-75	III	1303704	1305451	D	Y	KR314	1293124	1295561	1307017	1308228	11457
Y119D3A.2	fbxa-28	III	1297894	1299474	D	Y	JU258	1293124	1296291	1307017	1307077	10727
Y119D3A.2	fbxa-28	III	1297894	1299474	D	Y	KR314	1293124	1295561	1307017	1308228	11457
Y119D3A.3	fbxa-35	III	1295674	1297059	D	N	JU258	1293124	1296291	1307017	1307077	10727
Y119D3A.3	fbxa-35	III	1295674	1297059	D	Y	KR314	1293124	1295561	1307017	1308228	11457
Y14H12A.1	Y14H12A.1	II	3993248	3993841	D	N	AB1	3986405	3993310	3993745	3994930	436
Y14H12A.1	Y14H12A.1	II	3993248	3993841	D	N	JU258	3986405	3993310	3993745	3994930	436
Y14H12A.1	Y14H12A.1	II	3993248	3993841	D	N	JU263	3986405	3993310	3993612	3993745	303
Y17D7A.4	cyp-33D3	V	18832291	18837161	D	N	AB1	18831174	18832291	18837055	18843848	4765
Y17D7A.4	cyp-33D3	V	18832291	18837161	D	N	JU258	18828816	18832291	18837011	18843929	4721
Y17D7A.4	cyp-33D3	V	18832291	18837161	D	N	KR314	18829798	18832437	18837055	18843929	4619
Y17D7B.4	Y17D7B.4	V	18776163	18780181	D	N	JU258	18770198	18770400	18776232	18776304	5833
Y17D7B.5	Y17D7B.5	V	18775191	18776160	D	Y	JU258	18770198	18770400	18776232	18776304	5833
Y17D7B.6	clec-256	V	18771655	18772604	D	Y	JU258	18770198	18770400	18776232	18776304	5833
Y17D7B.8	clec-257	V	18773026	18773989	D	Y	JU258	18770198	18770400	18776232	18776304	5833
Y17D7C.1	Y17D7C.1	V	18707976	18711609	D	N	JU263	18709091	18710147	18711469	18714705	1323
Y17D7C.1	Y17D7C.1	V	18707976	18711609	D	N	KR314	18708143	18709091	18711469	18714705	2379
Y17G7B.11	Y17G7B.11	II	12060240	12062534	D	N	JU258	12060986	12061698	12062238	12065018	541
Y17G7B.19	Y17G7B.19	II	12108188	12112467	D	N	CB4858	12109442	12109998	12110048	12112833	51
Y19D10B.2	Y19D10B.2	V	2299996	2303174	D	N	JU258	2296835	2299997	2300341	2300451	345
Y19D10B.2	Y19D10B.2	V	2299996	2303174	D	N	JU263	2296835	2299997	2300451	2300676	455
Y19D10B.2	Y19D10B.2	V	2299996	2303174	D	N	MY2	2296835	2299997	2300451	2300676	455
Y19D10B.6	Y19D10B.6	V	2318525	2321052	D	N	JU258	2317083	2319179	2327141	2334409	7963
Y19D10B.6	Y19D10B.6	V	2318525	2321052	D	N	JU263	2317083	2319179	2327141	2334409	7963
Y19D10B.6	Y19D10B.6	V	2318525	2321052	D	N	MY2	2317083	2319179	2327141	2334409	7963
Y19D10B.7	Y19D10B.7	V	2322038	2322539	D	Y	JU258	2317083	2319179	2327141	2334409	7963
Y19D10B.7	Y19D10B.7	V	2322038	2322539	D	Y	JU263	2317083	2319179	2327141	2334409	7963
Y19D10B.7	Y19D10B.7	V	2322038	2322539	D	Y	MY2	2317083	2319179	2327141	2334409	7963

Y20C6A.1	Y20C6A.1	V	17371512	17376705	D	N	KR314	17360012	17371630	17489726	17490760	118097
Y20C6A.2	fbxb-7	V	17377389	17379198	D	Y	KR314	17360012	17371630	17489726	17490760	118097
Y20C6A.3	Y20C6A.3	V	17382058	17383341	D	Y	KR314	17360012	17371630	17489726	17490760	118097
Y20C6A.4	Y20C6A.4	V	17380200	17381087	D	Y	KR314	17360012	17371630	17489726	17490760	118097
Y22D7AR.14	Y22D7AR.14	III	1688133	1691759	D	Y	JU263	1685189	1686234	1692191	1692250	5958
Y22D7AR.14	Y22D7AR.14	III	1688133	1691759	D	Y	JU322	1685189	1686234	1692191	1692250	5958
Y22D7AR.7	Y22D7AR.7	III	1683619	1688088	D	N	JU263	1685189	1686234	1692191	1692250	5958
Y22D7AR.7	Y22D7AR.7	III	1683619	1688088	D	N	JU322	1685189	1686234	1692191	1692250	5958
Y22D7AR.8	srd-64	III	1692070	1695095	D	N	JU263	1685189	1686234	1692191	1692250	5958
Y22D7AR.8	srd-64	III	1692070	1695095	D	N	JU322	1685189	1686234	1692191	1692250	5958
Y26D4A.10	Y26D4A.10	I	13098675	13099925	D	Y	AB1	13084717	13084921	13104191	13107776	19271
Y26D4A.13	Y26D4A.13	I	13091097	13094425	D	Y	AB1	13084717	13084921	13104191	13107776	19271
Y26D4A.14	Y26D4A.14	I	13094672	13098370	D	Y	AB1	13084717	13084921	13104191	13107776	19271
Y26D4A.17	Y26D4A.17	I	13077544	13077929	D	Y	AB1	13071050	13072099	13081538	13081763	9440
Y26D4A.21	Y26D4A.21	I	13101344	13101889	D	Y	AB1	13084717	13084921	13104191	13107776	19271
Y26D4A.3	Y26D4A.3	I	13072055	13072930	D	N	AB1	13071050	13072099	13081538	13081763	9440
Y26D4A.5	Y26D4A.5	I	13076610	13077008	D	Y	AB1	13071050	13072099	13081538	13081763	9440
Y26D4A.6	clec-108	I	13081435	13082153	D	N	AB1	13071050	13072099	13081538	13081763	9440
Y26D4A.8	Y26D4A.8	I	13083308	13085021	D	N	AB1	13084717	13084921	13104191	13107776	19271
Y26D4A.9	Y26D4A.9	I	13086671	13090978	D	Y	AB1	13084717	13084921	13104191	13107776	19271
Y27F2A.11	Y27F2A.11	II	3189333	3190949	D	Y	MY2	3180783	3180993	3196064	3196905	15072
Y27F2A.2	sri-78	II	3185153	3186785	D	Y	MY2	3180783	3180993	3196064	3196905	15072
Y27F2A.3a	sri-40	II	3177961	3180463	D	N	CB4853	3176430	3178168	3179618	3180437	1451
Y27F2A.3a	sri-40	II	3177961	3180463	D	N	CB4854	3176430	3178168	3179618	3180366	1451
Y27F2A.3a	sri-40	II	3177961	3180463	D	N	CB4856	3176430	3178168	3179618	3180366	1451
Y27F2A.3a	sri-40	II	3177961	3180463	D	N	CB4858	3176430	3178168	3179618	3180437	1451
Y27F2A.3a	sri-40	II	3177961	3180463	D	N	JU322	3176430	3178168	3179618	3180366	1451
Y27F2A.3a	sri-40	II	3177961	3180463	D	N	MY2	3176430	3178168	3179618	3180366	1451
Y27F2A.4	sri-33	II	3155162	3156844	D	N	CB4853	3155840	3156114	3158199	3158487	2086
Y27F2A.4	sri-33	II	3155162	3156844	D	N	CB4854	3155840	3156114	3158199	3158487	2086
Y27F2A.4	sri-33	II	3155162	3156844	D	N	CB4858	3155840	3156114	3158199	3158487	2086
Y27F2A.4	sri-33	II	3155162	3156844	D	N	JU322	3155840	3156114	3158199	3158487	2086
Y27F2A.4	sri-33	II	3155162	3156844	D	N	KR314	3155840	3156114	3158199	3158487	2086
Y27F2A.6	Y27F2A.6	II	3180535	3182671	D	N	MY2	3180783	3180993	3196064	3196905	15072
Y27F2A.7	sri-34	II	3157486	3159080	D	N	CB4853	3155840	3156114	3158199	3158487	2086
Y27F2A.7	sri-34	II	3157486	3159080	D	N	CB4854	3155840	3156114	3158199	3158487	2086
Y27F2A.7	sri-34	II	3157486	3159080	D	N	CB4858	3155840	3156114	3158199	3158487	2086
Y27F2A.7	sri-34	II	3157486	3159080	D	N	JU322	3155840	3156114	3158199	3158487	2086
Y27F2A.7	sri-34	II	3157486	3159080	D	N	KR314	3155840	3156114	3158199	3158487	2086
Y27F2A.7	sri-34	II	3157486	3159080	D	N	MY2	3157382	3157544	3159025	3168772	1482
Y27F2A.8	Y27F2A.8	II	3183796	3184572	D	Y	MY2	3180783	3180993	3196064	3196905	15072
Y27F2A.9	Y27F2A.9	II	3191321	3191605	D	Y	MY2	3180783	3180993	3196064	3196905	15072
Y37H2A.7	Y37H2A.7	V	18131632	18134443	D	N	MY2	18131013	18131736	18133511	18134513	1776
Y37H2B.1	Y37H2B.1	V	18226999	18228142	D	Y	CB4856	18223164	18224107	18235017	18235065	10911
Y37H2B.1	Y37H2B.1	V	18226999	18228142	D	Y	JU258	18186176	18186552	18247813	18248607	61262
Y38E10A.28	Y38E10A.28	II	12629624	12631907	D	N	JU258	12629025	12629268	12630382	12631496	1115
Y38E10A.28	Y38E10A.28	II	12629624	12631907	D	N	JU322	12629910	12630015	12630268	12630343	254
Y38E10A.28	Y38E10A.28	II	12629624	12631907	D	N	KR314	12629910	12630015	12630268	12630382	254
Y38E10A.28	Y38E10A.28	II	12629624	12631907	D	N	MY2	12629025	12629268	12630382	12631496	1115
Y38F2AR.12a	Y38F2AR.12	IV	2399558	2412408	D	N	KR314	2408582	2409680	2410880	2411709	1201
Y38H6C.11	fbxa-150	V	20518009	20519849	D	N	MY2	20518968	20519060	20519219	20519550	160
Y38H6C.5	dct-10	V	20501027	20505395	A	N	JU258	20500740	20501312	20503971	20504483	2660
Y39A3A.5	ins-16	III	2060661	2060945	D	N	JU263	2059854	2060695	2060896	2066647	202
Y39A3A.5	ins-16	III	2060661	2060945	D	N	JU322	2059854	2060695	2060896	2066647	202
Y39A3CR.8	Y39A3CR.8	III	1882217	1883006	D	N	CB4856	1882105	1882614	1883028	1883734	415
Y39A3CR.8	Y39A3CR.8	III	1882217	1883006	D	N	JU322	1882272	1882614	1883028	1883734	415
Y39G10AR.5	zeel-1	I	2342384	2350469	D	N	CB4853	2338377	2342371	2350416	2357884	8046
Y39G10AR.5	zeel-1	I	2342384	2350469	D	N	CB4856	2338455	2342298	2350416	2357884	8119
Y39G10AR.5	zeel-1	I	2342384	2350469	D	N	CB4858	2338377	2342298	2350416	2357884	8119
Y39G10AR.5	zeel-1	I	2342384	2350469	D	N	JU322	2338377	2342298	2350416	2357884	8119

Y39G10AR.5	zeel-1	I	2342384	2350469	D	N	KR314	2338494	2342298	2350416	2357884	8119
Y39G8C.2	Y39G8C.2	II	14095366	14097293	D	N	CB4854	14095628	14096204	14096428	14096607	225
Y39G8C.2	Y39G8C.2	II	14095366	14097293	D	N	CB4856	14095628	14096204	14096490	14096651	287
Y40A1A.1	Y40A1A.1	X	2000941	2002484	D	N	AB1	1991043	1997186	2002425	2014349	5240
Y40A1A.1	Y40A1A.1	X	2000941	2002484	D	N	CB4853	1991043	1997186	2002425	2014349	5240
Y40A1A.1	Y40A1A.1	X	2000941	2002484	D	N	CB4854	1991043	1997186	2002425	2014349	5240
Y40A1A.1	Y40A1A.1	X	2000941	2002484	D	N	CB4858	1991043	1997186	2002425	2014349	5240
Y40A1A.1	Y40A1A.1	X	2000941	2002484	D	N	JU263	1991043	1997186	2002378	2014349	5193
Y40A1A.1	Y40A1A.1	X	2000941	2002484	D	N	KR314	1991043	1997186	2002425	2014349	5240
Y40A1A.1	Y40A1A.1	X	2000941	2002484	D	N	MY2	1991043	1997186	2002425	2014349	5240
Y40A1A.3	Y40A1A.3	X	1995571	1998107	D	N	AB1	1991043	1997186	2002425	2014349	5240
Y40A1A.3	Y40A1A.3	X	1995571	1998107	D	N	CB4853	1991043	1997186	2002425	2014349	5240
Y40A1A.3	Y40A1A.3	X	1995571	1998107	D	N	CB4854	1991043	1997186	2002425	2014349	5240
Y40A1A.3	Y40A1A.3	X	1995571	1998107	D	N	CB4858	1991043	1997186	2002425	2014349	5240
Y40A1A.3	Y40A1A.3	X	1995571	1998107	D	N	JU263	1991043	1997186	2002378	2014349	5193
Y40A1A.3	Y40A1A.3	X	1995571	1998107	D	N	KR314	1991043	1997186	2002425	2014349	5240
Y40A1A.3	Y40A1A.3	X	1995571	1998107	D	N	MY2	1991043	1997186	2002425	2014349	5240
Y40B10A.3	srab-23	V	2033792	2035645	D	Y	CB4854	2006917	2008669	2045822	2049093	37154
Y40B10A.3	srab-23	V	2033792	2035645	D	N	CB4856	2027804	2030835	2035594	2038195	4760
Y40B10A.3	srab-23	V	2033792	2035645	D	N	JU263	2027804	2030835	2035594	2038195	4760
Y40B10A.3	srab-23	V	2033792	2035645	D	N	MY2	2027804	2030835	2035594	2038195	4760
Y40B10A.4	Y40B10A.4	V	2038195	2041416	D	Y	CB4854	2006917	2008669	2045822	2049093	37154
Y40B10A.5	Y40B10A.5	V	2044726	2045269	D	Y	CB4854	2006917	2008669	2045822	2049093	37154
Y43F11A.5	set-24	II	12114906	12130883	D	N	MY2	12128664	12129567	12130806	12137569	1240
Y43F8B.11	Y43F8B.11	V	19470658	19472436	D	N	AB1	19389840	19395538	19470658	19470740	75121
Y43F8B.11	Y43F8B.11	V	19470658	19472436	D	N	CB4853	19389791	19399677	19470658	19470740	70982
Y43F8B.11	Y43F8B.11	V	19470658	19472436	D	N	JU322	19400192	19400613	19470658	19470740	70046
Y43F8B.11	Y43F8B.11	V	19470658	19472436	D	N	KR314	19446045	19446704	19470658	19470740	23955
Y43F8B.12	Y43F8B.12	V	19469031	19469518	D	Y	AB1	19389840	19395538	19470658	19470740	75121
Y43F8B.12	Y43F8B.12	V	19469031	19469518	D	Y	CB4853	19389791	19399677	19470658	19470740	70982
Y43F8B.12	Y43F8B.12	V	19469031	19469518	D	Y	CB4854	19395932	19400613	19469469	19470658	68857
Y43F8B.12	Y43F8B.12	V	19469031	19469518	D	N	CB4856	19400192	19400613	19469168	19470658	68556
Y43F8B.12	Y43F8B.12	V	19469031	19469518	D	Y	CB4858	19395467	19400613	19469469	19470740	68857
Y43F8B.12	Y43F8B.12	V	19469031	19469518	D	Y	JU258	19389840	19390251	19469469	19480048	79219
Y43F8B.12	Y43F8B.12	V	19469031	19469518	D	Y	JU263	19398882	19400613	19469469	19470658	68857
Y43F8B.12	Y43F8B.12	V	19469031	19469518	D	Y	JU322	19400192	19400613	19470658	19470740	70046
Y43F8B.12	Y43F8B.12	V	19469031	19469518	D	Y	KR314	19446045	19446704	19470658	19470740	23955
Y43F8B.12	Y43F8B.12	V	19469031	19469518	D	Y	MY2	19389840	19390251	19469469	19470740	79219
Y43F8B.13	Y43F8B.13	V	19455981	19463723	D	Y	AB1	19389840	19395538	19470658	19470740	75121
Y43F8B.13	Y43F8B.13	V	19455981	19463723	D	Y	CB4853	19389791	19399677	19470658	19470740	70982
Y43F8B.13	Y43F8B.13	V	19455981	19463723	D	Y	CB4854	19395932	19400613	19469469	19470658	68857
Y43F8B.13	Y43F8B.13	V	19455981	19463723	D	Y	CB4856	19400192	19400613	19469168	19470658	68556
Y43F8B.13	Y43F8B.13	V	19455981	19463723	D	Y	CB4858	19395467	19400613	19469469	19470740	68857
Y43F8B.13	Y43F8B.13	V	19455981	19463723	D	Y	JU258	19389840	19390251	19469469	19480048	79219
Y43F8B.13	Y43F8B.13	V	19455981	19463723	D	Y	JU263	19398882	19400613	19469469	19470658	68857
Y43F8B.13	Y43F8B.13	V	19455981	19463723	D	Y	JU322	19400192	19400613	19470658	19470740	70046
Y43F8B.13	Y43F8B.13	V	19455981	19463723	D	Y	KR314	19446045	19446704	19470658	19470740	23955
Y43F8B.13	Y43F8B.13	V	19455981	19463723	D	Y	MY2	19389840	19390251	19469469	19470740	79219
Y43F8B.14	Y43F8B.14	V	19452226	19454995	D	Y	AB1	19389840	19395538	19470658	19470740	75121
Y43F8B.14	Y43F8B.14	V	19452226	19454995	D	Y	CB4853	19389791	19399677	19470658	19470740	70982
Y43F8B.14	Y43F8B.14	V	19452226	19454995	D	Y	CB4854	19395932	19400613	19469469	19470658	68857
Y43F8B.14	Y43F8B.14	V	19452226	19454995	D	Y	CB4856	19400192	19400613	19469168	19470658	68556
Y43F8B.14	Y43F8B.14	V	19452226	19454995	D	Y	CB4858	19395467	19400613	19469469	19470740	68857
Y43F8B.14	Y43F8B.14	V	19452226	19454995	D	Y	JU258	19389840	19390251	19469469	19480048	79219
Y43F8B.14	Y43F8B.14	V	19452226	19454995	D	Y	JU263	19398882	19400613	19469469	19470658	68857
Y43F8B.14	Y43F8B.14	V	19452226	19454995	D	Y	JU322	19400192	19400613	19470658	19470740	70046
Y43F8B.14	Y43F8B.14	V	19452226	19454995	D	Y	KR314	19446045	19446704	19470658	19470740	23955
Y43F8B.14	Y43F8B.14	V	19452226	19454995	D	Y	MY2	19389840	19390251	19469469	19470740	79219
Y43F8B.15	Y43F8B.15	V	19460782	19462066	D	Y	AB1	19389840	19395538	19470658	19470740	75121
Y43F8B.15	Y43F8B.15	V	19460782	19462066	D	Y	CB4853	19389791	19399677	19470658	19470740	70982

Y43F8B.15	Y43F8B.15	V	19460782	19462066	D	Y	CB4854	19395932	19400613	19469469	19470658	68857
Y43F8B.15	Y43F8B.15	V	19460782	19462066	D	Y	CB4856	19400192	19400613	19469168	19470658	68556
Y43F8B.15	Y43F8B.15	V	19460782	19462066	D	Y	CB4858	19395467	19400613	19469469	19470740	68857
Y43F8B.15	Y43F8B.15	V	19460782	19462066	D	Y	JU258	19389840	19390251	19469469	19480048	79219
Y43F8B.15	Y43F8B.15	V	19460782	19462066	D	Y	JU263	19398882	19400613	19469469	19470658	68857
Y43F8B.15	Y43F8B.15	V	19460782	19462066	D	Y	JU322	19400192	19400613	19470658	19470740	70046
Y43F8B.15	Y43F8B.15	V	19460782	19462066	D	Y	KR314	19446045	19446704	19470658	19470740	23955
Y43F8B.15	Y43F8B.15	V	19460782	19462066	D	Y	MY2	19389840	19390251	19469469	19470740	79219
Y43F8B.18	Y43F8B.18	V	19485953	19486693	D	Y	JU258	19480130	19480251	19492283	19494156	12033
Y43F8B.18	Y43F8B.18	V	19485953	19486693	D	Y	KR314	19480130	19480251	19492283	19494156	12033
Y43F8B.5	scl-21	V	19494725	19495650	D	N	JU258	19494741	19494879	19495575	19503098	697
Y43F8B.5	scl-21	V	19494725	19495650	D	N	KR314	19494741	19494879	19495575	19503098	697
Y43F8B.5	scl-21	V	19494725	19495650	D	N	MY2	19494741	19494879	19495575	19503098	697
Y43F8B.6	Y43F8B.6	V	19492107	19494620	D	N	JU258	19480130	19480251	19492283	19494156	12033
Y43F8B.6	Y43F8B.6	V	19492107	19494620	D	N	KR314	19480130	19480251	19492283	19494156	12033
Y43F8B.9	Y43F8B.9	V	19479167	19480371	D	N	JU258	19480130	19480251	19492283	19494156	12033
Y43F8B.9	Y43F8B.9	V	19479167	19480371	D	N	KR314	19480130	19480251	19492283	19494156	12033
Y43F8C.19	srv-3	V	19702279	19704338	D	N	MY2	19701575	19702302	19704251	19705114	1950
Y45F10A.3	Y45F10A.3	IV	13509193	13513363	A	N	AB1	13508034	13508881	13510000	13510192	1120
Y45F10A.4	seld-1	IV	13506047	13509018	A	N	AB1	13508034	13508881	13510000	13510192	1120
Y46C8AL.1	clec-73	IV	3953929	3956200	D	Y	CB4856	3952321	3953875	3957047	3957119	3173
Y46C8AL.4	clec-71	IV	3937458	3939918	D	Y	JU258	3936191	3937399	3940096	3941411	2698
Y46C8AL.5	clec-72	IV	3944801	3947358	D	N	CB4856	3941585	3947024	3948749	3948868	1726
Y46C8AL.8	clec-74	IV	3957024	3960133	D	N	CB4856	3952321	3953875	3957047	3957119	3173
Y46C8AL.9a	clec-75	IV	3960728	3964651	D	N	CB4856	3959404	3961146	3968900	3972697	7755
Y46C8AR.1	clec-76	IV	3965240	3969019	D	N	CB4856	3959404	3961146	3968900	3972697	7755
Y46D2A.1	Y46D2A.1	II	3323335	3326426	D	Y	JU258	3305367	3306370	3353651	3369831	47282
Y46D2A.1	Y46D2A.1	II	3323335	3326426	D	Y	JU322	3305367	3309390	3330254	3330712	20865
Y46D2A.1	Y46D2A.1	II	3323335	3326426	D	Y	KR314	3306283	3309390	3355966	3359208	46577
Y46D2A.2	Y46D2A.2	II	3321052	3323176	D	Y	JU258	3305367	3306370	3353651	3369831	47282
Y46D2A.2	Y46D2A.2	II	3321052	3323176	D	Y	JU322	3305367	3309390	3330254	3330712	20865
Y46D2A.2	Y46D2A.2	II	3321052	3323176	D	Y	KR314	3306283	3309390	3355966	3359208	46577
Y46D2A.3	Y46D2A.3	II	3326507	3327035	D	Y	JU258	3305367	3306370	3353651	3369831	47282
Y46D2A.3	Y46D2A.3	II	3326507	3327035	D	Y	JU322	3305367	3309390	3330254	3330712	20865
Y46D2A.3	Y46D2A.3	II	3326507	3327035	D	Y	KR314	3306283	3309390	3355966	3359208	46577
Y46E12A.2	Y46E12A.2	III	1758710	1759772	D	N	KR314	1754932	1758323	1759072	1760063	750
Y46G5A.25	snf-4	II	12832784	12835768	D	N	JU258	12834388	12835082	12835749	12836336	668
Y46G5A.39	Y46G5A.39	II	12752762	12753721	D	Y	CB4856	12749101	12749888	12757363	12760568	7476
Y46G5A.39	Y46G5A.39	II	12752762	12753721	D	Y	JU258	12746108	12746571	12757363	12760568	10793
Y46G5A.39	Y46G5A.39	II	12752762	12753721	D	Y	JU322	12749101	12749888	12757363	12760568	7476
Y46G5A.39	Y46G5A.39	II	12752762	12753721	D	Y	MY2	12746108	12746571	12757363	12760568	10793
Y46G5A.7	Y46G5A.7	II	12755126	12756103	D	N	AB1	12749888	12754432	12755646	12756261	1215
Y46G5A.7	Y46G5A.7	II	12755126	12756103	D	N	CB4853	12749888	12754432	12755646	12756261	1215
Y46G5A.7	Y46G5A.7	II	12755126	12756103	D	N	CB4854	12749888	12754432	12755646	12756261	1215
Y46G5A.7	Y46G5A.7	II	12755126	12756103	D	Y	CB4856	12749101	12749888	12757363	12760568	7476
Y46G5A.7	Y46G5A.7	II	12755126	12756103	D	Y	JU258	12746108	12746571	12757363	12760568	10793
Y46G5A.7	Y46G5A.7	II	12755126	12756103	D	Y	JU263	12749101	12754432	12757363	12760568	2932
Y46G5A.7	Y46G5A.7	II	12755126	12756103	D	Y	JU322	12749101	12749888	12757363	12760568	7476
Y46G5A.7	Y46G5A.7	II	12755126	12756103	D	N	KR314	12749888	12754432	12755646	12756261	1215
Y46G5A.7	Y46G5A.7	II	12755126	12756103	D	Y	MY2	12746108	12746571	12757363	12760568	10793
Y46G5A.8	Y46G5A.8	II	12756261	12757543	D	N	CB4856	12749101	12749888	12757363	12760568	7476
Y46G5A.8	Y46G5A.8	II	12756261	12757543	D	N	JU258	12746108	12746571	12757363	12760568	10793
Y46G5A.8	Y46G5A.8	II	12756261	12757543	D	N	JU263	12749101	12754432	12757363	12760568	2932
Y46G5A.8	Y46G5A.8	II	12756261	12757543	D	N	JU322	12749101	12749888	12757363	12760568	7476
Y46G5A.8	Y46G5A.8	II	12756261	12757543	D	N	MY2	12746108	12746571	12757363	12760568	10793
Y47H10A.1	clp-3	I	12073133	12083601	D	N	CB4856	12073961	12074000	12074883	12080404	884
Y47H10A.1	clp-3	I	12073133	12083601	D	N	JU258	12072780	12073111	12074693	12074770	1583
Y47H10A.1	clp-3	I	12073133	12083601	D	N	JU258	12074883	12080404	12085101	12086411	4698
Y47H10A.1	clp-3	I	12073133	12083601	D	N	JU263	12072780	12073111	12074693	12074770	1583
Y47H10A.1	clp-3	I	12073133	12083601	D	N	JU263	12074883	12080404	12085101	12086411	4698

Y47H10A.1	clp-3	I	12073133	12083601	D	N	KR314	12072780	12073111	12074693	12074770	1583
Y47H10A.1	clp-3	I	12073133	12083601	D	N	KR314	12074883	12080404	12085796	12086337	5393
Y47H10A.1	clp-3	I	12073133	12083601	D	N	MY2	12072780	12073111	12074693	12074770	1583
Y47H10A.1	clp-3	I	12073133	12083601	D	N	MY2	12074883	12080404	12085796	12086337	5393
Y47H10A.2	Y47H10A.2	I	12085017	12087820	D	N	JU258	12074883	12080404	12085101	12086411	4698
Y47H10A.2	Y47H10A.2	I	12085017	12087820	D	N	JU263	12074883	12080404	12085101	12086411	4698
Y47H10A.2	Y47H10A.2	I	12085017	12087820	D	N	KR314	12074883	12080404	12085796	12086337	5393
Y47H10A.2	Y47H10A.2	I	12085017	12087820	D	N	MY2	12074883	12080404	12085796	12086337	5393
Y47H9B.2	Y47H9B.2	I	11744162	11747404	D	N	CB4858	11744222	11744796	11745411	11747181	616
Y47H9C.10	fbxa-216	I	11901598	11903160	D	N	CB4856	11896805	11897641	11903091	11903854	5451
Y47H9C.10	fbxa-216	I	11901598	11903160	D	N	JU258	11896805	11897641	11903091	11903854	5451
Y47H9C.10	fbxa-216	I	11901598	11903160	D	N	JU263	11896805	11897641	11903091	11903578	5451
Y47H9C.10	fbxa-216	I	11901598	11903160	D	N	MY2	11896805	11897641	11903091	11903578	5451
Y47H9C.9	Y47H9C.9	I	11898779	11901448	D	Y	CB4856	11896805	11897641	11903091	11903854	5451
Y47H9C.9	Y47H9C.9	I	11898779	11901448	D	N	CB4858	11896805	11897641	11900122	11902248	2482
Y47H9C.9	Y47H9C.9	I	11898779	11901448	D	Y	JU258	11896805	11897641	11903091	11903854	5451
Y47H9C.9	Y47H9C.9	I	11898779	11901448	D	Y	JU263	11896805	11897641	11903091	11903578	5451
Y47H9C.9	Y47H9C.9	I	11898779	11901448	D	Y	MY2	11896805	11897641	11903091	11903578	5451
Y48D7A.1	Y48D7A.1	X	3009491	3010167	D	N	CB4856	3006483	3009498	3010048	3012409	551
Y48E1B.14a	Y48E1B.14	II	13522827	13531128	A	N	CB4853	13513407	13513515	13526754	13526822	13240
Y48E1B.14a	Y48E1B.14	II	13522827	13531128	A	N	CB4858	13513407	13513449	13526754	13527016	13306
Y49F6A.1	Y49F6A.1	II	3603381	3607378	D	N	CB4856	3604725	3604795	3606730	3606769	1936
Y49F6C.2	Y49F6C.2	II	3378956	3379507	D	N	MY2	3378382	3379217	3394898	3395853	15682
Y49F6C.5	bath-23	II	3371305	3372140	D	N	AB1	3371375	3371440	3372084	3375149	645
Y49F6C.5	bath-23	II	3371305	3372140	D	N	CB4853	3371375	3371440	3372084	3375149	645
Y49F6C.5	bath-23	II	3371305	3372140	D	N	CB4858	3371375	3371440	3372084	3375149	645
Y49F6C.5	bath-23	II	3371305	3372140	D	N	JU258	3371375	3371440	3372084	3375192	645
Y49F6C.5	bath-23	II	3371305	3372140	D	N	KR314	3371375	3371440	3372084	3375149	645
Y49F6C.6	Y49F6C.6	II	3363341	3365956	D	N	CB4856	3334109	3335696	3363341	3363381	27646
Y50D4B.5	clec-203	V	1088371	1089893	D	N	AB1	1085760	1086092	1088458	1088690	2367
Y50D4B.6	Y50D4B.6	V	1084318	1087274	D	N	AB1	1085760	1086092	1088458	1088690	2367
Y50D4B.6	Y50D4B.6	V	1084318	1087274	D	N	CB4856	1086092	1086244	1086449	1086987	206
Y51A2A.1	clec-247	V	18284807	18286405	D	N	AB1	18283066	18285026	18291314	18292896	6289
Y51A2A.1	clec-247	V	18284807	18286405	D	N	CB4853	18283066	18285026	18291314	18292896	6289
Y51A2A.1	clec-247	V	18284807	18286405	D	N	CB4854	18283066	18285026	18291314	18292896	6289
Y51A2A.1	clec-247	V	18284807	18286405	D	N	CB4856	18283066	18285026	18300599	18318725	15574
Y51A2A.1	clec-247	V	18284807	18286405	D	N	CB4858	18283066	18285026	18291314	18292896	6289
Y51A2A.1	clec-247	V	18284807	18286405	D	Y	JU258	18277830	18282879	18287431	18289554	4553
Y51A2A.1	clec-247	V	18284807	18286405	D	N	JU263	18283066	18285026	18291314	18292896	6289
Y51A2A.1	clec-247	V	18284807	18286405	D	N	JU322	18283066	18285026	18291314	18292896	6289
Y51A2A.1	clec-247	V	18284807	18286405	D	N	KR314	18283066	18285026	18291314	18292896	6289
Y51A2A.1	clec-247	V	18284807	18286405	D	Y	MY2	18277830	18282879	18287431	18289554	4553
Y51A2A.11	clec-248	V	18294324	18295827	D	Y	CB4856	18283066	18285026	18300599	18318725	15574
Y51A2A.12	Y51A2A.12	V	18292549	18292909	D	Y	CB4856	18283066	18285026	18300599	18318725	15574
Y51A2A.4	Y51A2A.4	V	18290899	18291313	D	Y	AB1	18283066	18285026	18291314	18292896	6289
Y51A2A.4	Y51A2A.4	V	18290899	18291313	D	Y	CB4853	18283066	18285026	18291314	18292896	6289
Y51A2A.4	Y51A2A.4	V	18290899	18291313	D	Y	CB4854	18283066	18285026	18291314	18292896	6289
Y51A2A.4	Y51A2A.4	V	18290899	18291313	D	Y	CB4856	18283066	18285026	18300599	18318725	15574
Y51A2A.4	Y51A2A.4	V	18290899	18291313	D	Y	CB4858	18283066	18285026	18291314	18292896	6289
Y51A2A.4	Y51A2A.4	V	18290899	18291313	D	Y	JU263	18283066	18285026	18291314	18292896	6289
Y51A2A.4	Y51A2A.4	V	18290899	18291313	D	Y	JU322	18283066	18285026	18291314	18292896	6289
Y51A2A.4	Y51A2A.4	V	18290899	18291313	D	Y	KR314	18283066	18285026	18291314	18292896	6289
Y51A2A.5	Y51A2A.5	V	18298771	18300678	D	N	CB4856	18283066	18285026	18300599	18318725	15574
Y51B9A.9	Y51B9A.9	II	9395488	9397473	D	N	JU258	9390773	9395488	9396326	9396617	839
Y51H7BR.1	fbxb-42	II	1537627	1538633	D	Y	CB4853	1534583	1535351	1548926	1549814	13576
Y51H7BR.1	fbxb-42	II	1537627	1538633	D	N	CB4856	1537134	1537855	1547066	1547149	9212
Y51H7BR.1	fbxb-42	II	1537627	1538633	D	Y	CB4858	1534521	1535351	1547819	1549474	12469
Y51H7BR.1	fbxb-42	II	1537627	1538633	D	Y	JU258	1534143	1535351	1593891	1593940	58541
Y51H7BR.1	fbxb-42	II	1537627	1538633	D	Y	KR314	1534583	1535351	1547819	1548926	12469
Y51H7BR.2	fbxb-43	II	1536265	1537281	D	Y	CB4853	1534583	1535351	1548926	1549814	13576

Y51H7BR.2	fbxb-43	II	1536265	1537281	D	N	CB4856	1534143	1534521	1536633	1537072	2113
Y51H7BR.2	fbxb-43	II	1536265	1537281	D	Y	CB4858	1534521	1535351	1547819	1549474	12469
Y51H7BR.2	fbxb-43	II	1536265	1537281	D	Y	JU258	1534143	1535351	1593891	1593940	58541
Y51H7BR.2	fbxb-43	II	1536265	1537281	D	Y	KR314	1534583	1535351	1547819	1548926	12469
Y51H7BR.3	Y51H7BR.3	II	1534467	1535169	D	N	CB4856	1534143	1534521	1536633	1537072	2113
Y53C10A.10	Y53C10A.10	I	12020748	12028288	D	N	CB4858	12026036	12027985	12028145	12028213	161
Y53F4B.20	Y53F4B.20	II	15084378	15089554	A	N	CB4854	15084797	15084844	15085167	15085798	324
Y53F4B.20	Y53F4B.20	II	15084378	15089554	A	N	CB4856	15084473	15084797	15085083	15085167	287
Y53F4B.20	Y53F4B.20	II	15084378	15089554	A	N	CB4858	15084647	15084797	15085167	15085798	371
Y53F4B.20	Y53F4B.20	II	15084378	15089554	A	N	JU258	15084473	15084797	15085167	15085798	371
Y53F4B.20	Y53F4B.20	II	15084378	15089554	A	N	JU322	15084647	15084797	15085167	15085798	371
Y53F4B.20	Y53F4B.20	II	15084378	15089554	A	N	KR314	15084585	15084647	15085167	15085798	521
Y53F4B.5	Y53F4B.5	II	14982494	14983911	D	N	CB4856	14976660	14982543	14985471	14985732	2929
Y53F4B.5	Y53F4B.5	II	14982494	14983911	D	N	KR314	14983264	14983789	14991178	14994915	7390
Y53F4B.6	Y53F4B.6	II	14985602	14986694	D	Y	KR314	14983264	14983789	14991178	14994915	7390
Y53H1A.3	clec-100	I	11234294	11235168	D	N	JU258	11233192	11234306	11235432	11235536	1127
Y53H1A.3	clec-100	I	11234294	11235168	D	N	JU263	11233192	11234306	11235067	11235536	762
Y53H1A.3	clec-100	I	11234294	11235168	D	N	KR314	11233192	11234306	11235432	11235536	1127
Y53H1B.4	Y53H1B.4	I	11300735	11301654	D	N	JU263	11299508	11300849	11301086	11306072	238
Y53H1B.4	Y53H1B.4	I	11300735	11301654	D	Y	KR314	11297160	11299293	11307482	11311827	8190
Y53H1B.6	Y53H1B.6	I	11303731	11305210	D	Y	KR314	11297160	11299293	11307482	11311827	8190
Y53H1C.3	Y53H1C.3	I	11417874	11421227	D	N	CB4858	11418288	11418354	11419944	11421108	1591
Y53H1C.3	Y53H1C.3	I	11417874	11421227	D	N	JU258	11418288	11418354	11419944	11421108	1591
Y53H1C.3	Y53H1C.3	I	11417874	11421227	D	N	JU263	11418032	11418426	11419892	11421108	1467
Y53H1C.3	Y53H1C.3	I	11417874	11421227	D	N	MY2	11418288	11418354	11419944	11421108	1591
Y54F10BM.5	fbxa-9	III	2266680	2268287	D	N	AB1	2267232	2268078	2272238	2274074	4161
Y54F10BM.5	fbxa-9	III	2266680	2268287	D	N	JU322	2267232	2268078	2272238	2274074	4161
Y54F10BM.5	fbxa-9	III	2266680	2268287	D	N	KR314	2267232	2268078	2272238	2274074	4161
Y54F10BM.7	fbxa-48	III	2266967	2272162	D	N	AB1	2267232	2268078	2272238	2274074	4161
Y54F10BM.7	fbxa-48	III	2266967	2272162	D	N	JU322	2267232	2268078	2272238	2274074	4161
Y54F10BM.7	fbxa-48	III	2266967	2272162	D	N	KR314	2267232	2268078	2272238	2274074	4161
Y55B1AR.3	Y55B1AR.3	III	602705	603819	D	N	CB4854	602753	602795	602978	603092	184
Y57A10A.7	Y57A10A.7	II	12159398	12162574	D	N	CB4853	12156753	12159441	12162276	12162869	2836
Y57A10A.7	Y57A10A.7	II	12159398	12162574	D	N	CB4854	12156753	12159441	12162276	12162869	2836
Y57A10A.7	Y57A10A.7	II	12159398	12162574	D	N	CB4858	12156753	12159441	12162276	12162869	2836
Y57A10A.7	Y57A10A.7	II	12159398	12162574	D	N	JU263	12156753	12159441	12162276	12162869	2836
Y57A10A.7	Y57A10A.7	II	12159398	12162574	D	N	KR314	12156753	12159441	12162276	12162869	2836
Y57A10A.7	Y57A10A.7	II	12159398	12162574	D	N	MY2	12156753	12159441	12162276	12162869	2836
Y57G11B.1	Y57G11B.1	IV	14549792	14551922	D	N	JU258	14547120	14547780	14550230	14551095	2451
Y57G11C.1	haf-8	IV	14693994	14699902	D	N	CB4856	14687175	14694135	14694303	14694420	169
Y59E1A.1	fbxa-40	X	1937942	1942194	D	N	CB4856	1930251	1931466	1940418	1957551	8953
Y59E9AL.5	Y59E9AL.5	IV	5221874	5222222	D	Y	CB4858	5217278	5220192	5224938	5228511	4747
Y59E9AL.6	Y59E9AL.6	IV	5224888	5225758	D	N	CB4858	5217278	5220192	5224938	5228511	4747
Y59E9AL.8	Y59E9AL.8	IV	5223309	5224280	D	Y	CB4858	5217278	5220192	5224938	5228511	4747
Y60A3A.24	Y60A3A.24	V	19974375	19977270	D	N	MY2	19971300	19971430	19977213	19978476	5784
Y60A3A.3	srh-183	V	19970998	19973708	D	N	MY2	19971300	19971430	19977213	19978476	5784
Y60C6A.1	Y60C6A.1	V	4783609	4786126	D	N	CB4856	4780205	4783636	4788086	4789599	4451
Y61B8A.1	srh-116	V	17252899	17255096	D	Y	CB4856	17225924	17226138	17261395	17261770	35258
Y61B8A.1	srh-116	V	17252899	17255096	D	Y	JU258	17229936	17238425	17347568	17348479	109144
Y61B8A.2	srh-115	V	17255722	17257162	D	Y	CB4856	17225924	17226138	17261395	17261770	35258
Y61B8A.2	srh-115	V	17255722	17257162	D	Y	JU258	17229936	17238425	17347568	17348479	109144
Y61B8A.4	fbxa-85	V	17259149	17260502	D	Y	CB4856	17225924	17226138	17261395	17261770	35258
Y61B8A.4	fbxa-85	V	17259149	17260502	D	Y	JU258	17229936	17238425	17347568	17348479	109144
Y61B8B.1	sri-70	V	17303022	17304406	D	Y	CB4856	17301021	17301143	17325589	17325981	24447
Y61B8B.1	sri-70	V	17303022	17304406	D	Y	JU258	17229936	17238425	17347568	17348479	109144
Y65B4BL.1	Y65B4BL.1	I	530569	532855	A	N	CB4856	531024	531095	532805	534461	1711
Y67D8B.1	Y67D8B.1	IV	3169942	3172606	A	N	AB1	3156042	3170029	3171898	3175680	1870
Y67D8B.1	Y67D8B.1	IV	3169942	3172606	D	N	CB4858	3156042	3170029	3171898	3175680	1870
Y68A4A.2	srz-47	V	17190803	17192306	D	N	CB4856	17190486	17190902	17197119	17197300	6218
Y68A4B.1	clec-243	V	17247293	17248983	D	Y	CB4856	17225924	17226138	17261395	17261770	35258

Y68A4B.1	clec-243	V	17247293	17248983	D	Y	JU258	17229936	17238425	17347568	17348479	109144
Y68A4B.2	clec-242	V	17243735	17245474	D	Y	CB4856	17225924	17226138	17261395	17261770	35258
Y68A4B.2	clec-242	V	17243735	17245474	D	Y	JU258	17229936	17238425	17347568	17348479	109144
Y68A4B.3	Y68A4B.3	V	17241676	17242031	D	Y	CB4856	17225924	17226138	17261395	17261770	35258
Y68A4B.3	Y68A4B.3	V	17241676	17242031	D	Y	JU258	17229936	17238425	17347568	17348479	109144
Y69A2AR.12	Y69A2AR.12	IV	2570987	2572028	D	Y	JU258	2556903	2563486	2594976	2600114	31491
Y69A2AR.13	Y69A2AR.13	IV	2569372	2570695	D	Y	JU258	2556903	2563486	2594976	2600114	31491
Y69A2AR.14	Y69A2AR.14	IV	2552730	2557103	D	N	JU322	2553746	2553855	2555752	2556161	1898
Y69A2AR.24	Y69A2AR.24	IV	2563701	2564063	D	Y	CB4856	2556903	2563486	2568445	2570796	4960
Y69A2AR.24	Y69A2AR.24	IV	2563701	2564063	D	Y	JU258	2556903	2563486	2594976	2600114	31491
Y69A2AR.25	Y69A2AR.25	IV	2565838	2568364	D	Y	CB4856	2556903	2563486	2568445	2570796	4960
Y69A2AR.25	Y69A2AR.25	IV	2565838	2568364	D	Y	JU258	2556903	2563486	2594976	2600114	31491
Y69A2AR.26	nhr-242	IV	2577116	2581580	D	Y	CB4856	2571288	2572089	2588387	2593325	16299
Y69A2AR.26	nhr-242	IV	2577116	2581580	D	Y	JU258	2556903	2563486	2594976	2600114	31491
Y69A2AR.26	nhr-242	IV	2577116	2581580	D	Y	JU322	2570796	2572089	2588387	2592669	16299
Y69A2AR.9	Y69A2AR.9	IV	2594655	2596271	D	N	CB4856	2593549	2593952	2594678	2595226	727
Y69A2AR.9	Y69A2AR.9	IV	2594655	2596271	D	N	JU258	2556903	2563486	2594976	2600114	31491
Y69A2AR.9	Y69A2AR.9	IV	2594655	2596271	D	N	JU322	2593549	2593952	2595121	2595226	1170
Y69H2.10a	Y69H2.10	V	18670280	18684325	D	N	CB4856	18675934	18678249	18684222	18686125	5974
Y6D1A.1	Y6D1A.1	II	11820500	11823534	D	N	CB4858	11820216	11820849	11821128	11821739	280
Y6D1A.1	Y6D1A.1	II	11820500	11823534	D	N	JU263	11820216	11820849	11821128	11821739	280
Y6E2A.6	srh-140	V	15722162	15723973	D	N	JU258	15722278	15723271	15725210	15725249	1940
Y6E2A.8	Y6E2A.8	V	15724823	15726676	D	N	JU258	15722278	15723271	15725210	15725249	1940
Y6G8.1	srz-45	V	17592354	17594210	D	N	CB4856	17593140	17593363	17598236	17598449	4874
Y6G8.2	Y6G8.2	V	17594316	17598700	D	N	CB4856	17593140	17593363	17598236	17598449	4874
Y71F9AL.18	pme-1	I	2907723	2924716	A	N	JU322	2913032	2915195	2916946	2921377	1752
Y71F9AL.2	Y71F9AL.2	I	2915177	2918807	A	N	JU322	2913032	2915195	2916946	2921377	1752
Y71H2AM.14a	Y71H2AM.14	III	2743764	2746643	D	N	CB4853	2744024	2744447	2745032	2745274	586
Y71H2AM.14a	Y71H2AM.14	III	2743764	2746643	D	N	CB4856	2744024	2744447	2745032	2745274	586
Y71H2AM.14a	Y71H2AM.14	III	2743764	2746643	D	N	CB4858	2744024	2744447	2745032	2745274	586
Y71H2B.1	Y71H2B.1	III	2629000	2631315	A	N	CB4853	2620549	2622026	2629081	2630074	7056
Y71H2B.2	Y71H2B.2	III	2623159	2627725	A	Y	CB4853	2620549	2622026	2629081	2630074	7056
Y71H2B.3	ppfr-3	III	2619925	2623038	A	N	CB4853	2620549	2622026	2629081	2630074	7056
Y73C8C.3	Y73C8C.3	V	3115302	3118127	D	N	CB4856	3116655	3116719	3116965	3117048	247
Y74C10AR.2	Y74C10AR.2	I	2482685	2483706	D	N	JU258	2482349	2483194	2483482	2485022	289
Y74C10AR.3	abtm-1	I	2472419	2485383	D	N	JU258	2482349	2483194	2483482	2485022	289
Y75B12A.2	Y75B12A.2	V	15109553	15110321	D	N	JU258	15105122	15109503	15110264	15111981	762
Y75B7B.2	Y75B7B.2	V	1274212	1276007	D	N	CB4856	1272795	1274592	1274909	1275518	318
Y75B8A.11	Y75B8A.11	III	12186304	12189996	D	N	KR314	12188968	12189660	12189753	12189918	94
Y75B8A.28	Y75B8A.28	III	12318988	12322783	A	N	JU263	12319250	12320776	12322654	12329345	1879
Y75B8A.31	Y75B8A.31	III	12351520	12354798	D	N	CB4853	12354378	12354638	12364170	12364243	9533
Y75B8A.31	Y75B8A.31	III	12351520	12354798	D	N	CB4854	12354378	12354638	12359445	12360263	4808
Y75B8A.31	Y75B8A.31	III	12351520	12354798	D	N	CB4856	12354378	12354638	12361074	12364170	6437
Y75B8A.31	Y75B8A.31	III	12351520	12354798	D	N	CB4858	12354378	12354638	12360216	12364170	5579
Y75B8A.31	Y75B8A.31	III	12351520	12354798	D	N	JU258	12350618	12354638	12364170	12364414	9533
Y75B8A.31	Y75B8A.31	III	12351520	12354798	D	N	JU263	12354378	12354638	12358522	12360711	3885
Y75B8A.31	Y75B8A.31	III	12351520	12354798	D	N	JU322	12354378	12354638	12359445	12364170	4808
Y75B8A.31	Y75B8A.31	III	12351520	12354798	D	N	KR314	12354378	12354638	12360216	12364170	5579
Y75B8A.31	Y75B8A.31	III	12351520	12354798	D	N	MY2	12354378	12354638	12359445	12364170	4808
Y75B8A.32	Y75B8A.32	III	12354824	12358441	D	N	AB1	12354378	12354962	12360216	12364243	5255
Y75B8A.32	Y75B8A.32	III	12354824	12358441	D	Y	CB4853	12354378	12354638	12364170	12364243	9533
Y75B8A.32	Y75B8A.32	III	12354824	12358441	D	Y	CB4854	12354378	12354638	12359445	12360263	4808
Y75B8A.32	Y75B8A.32	III	12354824	12358441	D	Y	CB4856	12354378	12354638	12361074	12364170	6437
Y75B8A.32	Y75B8A.32	III	12354824	12358441	D	Y	CB4858	12354378	12354638	12360216	12364170	5579
Y75B8A.32	Y75B8A.32	III	12354824	12358441	D	Y	JU258	12350618	12354638	12364170	12364414	9533
Y75B8A.32	Y75B8A.32	III	12354824	12358441	D	Y	JU263	12354378	12354638	12358522	12360711	3885
Y75B8A.32	Y75B8A.32	III	12354824	12358441	D	Y	JU322	12354378	12354638	12359445	12364170	4808
Y75B8A.32	Y75B8A.32	III	12354824	12358441	D	Y	KR314	12354378	12354638	12360216	12364170	5579
Y75B8A.32	Y75B8A.32	III	12354824	12358441	D	Y	MY2	12354378	12354638	12359445	12364170	4808
Y75B8A.33	Y75B8A.33	III	12361739	12367253	D	N	CB4853	12354378	12354638	12364170	12364243	9533

Y75B8A.33	Y75B8A.33	III	12361739	12367253	D	N	JU258	12350618	12354638	12364170	12364414	9533
Y75B8A.34	Y75B8A.34	III	12359445	12361128	D	N	AB1	12354378	12354962	12360216	12364243	5255
Y75B8A.34	Y75B8A.34	III	12359445	12361128	D	Y	CB4853	12354378	12354638	12364170	12364243	9533
Y75B8A.34	Y75B8A.34	III	12359445	12361128	D	N	CB4854	12354378	12354638	12359445	12360263	4808
Y75B8A.34	Y75B8A.34	III	12359445	12361128	D	N	CB4856	12354378	12354638	12361074	12364170	6437
Y75B8A.34	Y75B8A.34	III	12359445	12361128	D	N	CB4858	12354378	12354638	12360216	12364170	5579
Y75B8A.34	Y75B8A.34	III	12359445	12361128	D	Y	JU258	12350618	12354638	12364170	12364414	9533
Y75B8A.34	Y75B8A.34	III	12359445	12361128	D	N	JU322	12354378	12354638	12359445	12364170	4808
Y75B8A.34	Y75B8A.34	III	12359445	12361128	D	N	KR314	12354378	12354638	12360216	12364170	5579
Y75B8A.34	Y75B8A.34	III	12359445	12361128	D	N	MY2	12354378	12354638	12359445	12364170	4808
Y75D11A.1	Y75D11A.1	X	1756413	1757784	D	Y	CB4856	1747004	1752189	1778099	1781395	25911
Y75D11A.1	Y75D11A.1	X	1756413	1757784	D	Y	JU322	1747004	1752189	1774025	1777351	21837
Y7A5A.8	Y7A5A.8	X	15821343	15825059	D	Y	CB4853	15804045	15821293	15825060	15826599	3768
Y7A5A.8	Y7A5A.8	X	15821343	15825059	D	Y	MY2	15804045	15821293	15825060	15826599	3768
Y7A9C.7	srz-72	IV	16299111	16300232	D	Y	CB4856	16280625	16286565	16345044	16347072	58480
Y7A9C.8	srz-76	IV	16288073	16289270	D	Y	CB4856	16280625	16286565	16345044	16347072	58480
Y7A9C.9	srz-75	IV	16286615	16287785	D	Y	CB4856	16280625	16286565	16345044	16347072	58480
Y7A9C.9	srz-75	IV	16286615	16287785	D	N	KR314	16286565	16286760	16287718	16288188	959
Y82E9BL.1	Y82E9BL.1	III	1354987	1355410	D	Y	JU258	1345721	1346343	1409647	1413962	63305
Y82E9BL.1	Y82E9BL.1	III	1354987	1355410	D	N	JU263	1353444	1355120	1357839	1358000	2720
Y82E9BL.1	Y82E9BL.1	III	1354987	1355410	D	N	JU322	1353444	1355120	1358000	1359570	2881
Y82E9BL.1	Y82E9BL.1	III	1354987	1355410	D	Y	KR314	1345721	1346940	1357785	1357839	10846
Y82E9BL.13	fbxa-79	III	1305794	1308239	D	N	JU258	1293124	1296291	1307017	1307077	10727
Y82E9BL.13	fbxa-79	III	1305794	1308239	D	N	KR314	1293124	1295561	1307017	1308228	11457
Y82E9BL.14	fbxa-80	III	1313348	1315208	D	Y	JU258	1310994	1311955	1316567	1319093	4613
Y82E9BL.14	fbxa-80	III	1313348	1315208	D	Y	KR314	1312055	1313010	1316567	1319093	3558
Y82E9BL.15	fbxa-19	III	1315254	1316655	D	N	JU258	1310994	1311955	1316567	1319093	4613
Y82E9BL.15	fbxa-19	III	1315254	1316655	D	N	KR314	1312055	1313010	1316567	1319093	3558
Y82E9BL.16	fbxa-20	III	1311218	1313112	D	N	JU258	1310994	1311955	1316567	1319093	4613
Y82E9BL.16	fbxa-20	III	1311218	1313112	D	N	KR314	1312055	1313010	1316567	1319093	3558
Y82E9BL.2	Y82E9BL.2	III	1351206	1353808	D	Y	JU258	1345721	1346343	1409647	1413962	63305
Y82E9BL.2	Y82E9BL.2	III	1351206	1353808	D	Y	KR314	1345721	1346940	1357785	1357839	10846
Y82E9BL.3	Y82E9BL.3	III	1349742	1350826	D	Y	JU258	1345721	1346343	1409647	1413962	63305
Y82E9BL.3	Y82E9BL.3	III	1349742	1350826	D	Y	KR314	1345721	1346940	1357785	1357839	10846
Y82E9BL.4	fbxa-25	III	1347777	1349390	D	Y	JU258	1345721	1346343	1409647	1413962	63305
Y82E9BL.4	fbxa-25	III	1347777	1349390	D	Y	KR314	1345721	1346940	1357785	1357839	10846
Y82E9BL.5	Y82E9BL.5	III	1346305	1347118	D	N	JU258	1345721	1346343	1409647	1413962	63305
Y82E9BL.5	Y82E9BL.5	III	1346305	1347118	D	N	KR314	1345721	1346940	1357785	1357839	10846
Y82E9BR.10	Y82E9BR.10	III	1375235	1375581	D	Y	JU258	1345721	1346343	1409647	1413962	63305
Y82E9BR.10	Y82E9BR.10	III	1375235	1375581	D	Y	KR314	1362368	1362442	1406114	1410803	43673
Y82E9BR.11	Y82E9BR.11	III	1376935	1377993	D	Y	JU258	1345721	1346343	1409647	1413962	63305
Y82E9BR.11	Y82E9BR.11	III	1376935	1377993	D	Y	KR314	1362368	1362442	1406114	1410803	43673
Y82E9BR.12	fbxa-138	III	1379130	1383572	D	N	AB1	1380609	1380678	1409647	1410803	28970
Y82E9BR.12	fbxa-138	III	1379130	1383572	D	N	CB4853	1380609	1381364	1409647	1410803	28284
Y82E9BR.12	fbxa-138	III	1379130	1383572	D	N	CB4858	1380609	1381364	1405478	1410803	24115
Y82E9BR.12	fbxa-138	III	1379130	1383572	D	Y	JU258	1345721	1346343	1409647	1413962	63305
Y82E9BR.12	fbxa-138	III	1379130	1383572	D	Y	KR314	1362368	1362442	1406114	1410803	43673
Y82E9BR.12	fbxa-138	III	1379130	1383572	D	N	MY2	1380609	1380678	1406114	1409608	25437
Y82E9BR.13	Y82E9BR.13	III	1390442	1391842	D	Y	AB1	1380609	1380678	1409647	1410803	28970
Y82E9BR.13	Y82E9BR.13	III	1390442	1391842	D	Y	CB4853	1380609	1381364	1409647	1410803	28284
Y82E9BR.13	Y82E9BR.13	III	1390442	1391842	D	Y	CB4858	1380609	1381364	1405478	1410803	24115
Y82E9BR.13	Y82E9BR.13	III	1390442	1391842	D	Y	JU258	1345721	1346343	1409647	1413962	63305
Y82E9BR.13	Y82E9BR.13	III	1390442	1391842	D	Y	KR314	1362368	1362442	1406114	1410803	43673
Y82E9BR.13	Y82E9BR.13	III	1390442	1391842	D	Y	MY2	1380609	1380678	1406114	1409608	25437
Y82E9BR.14a	Y82E9BR.14	III	1409301	1414110	D	N	AB1	1380609	1380678	1409647	1410803	28970
Y82E9BR.14a	Y82E9BR.14	III	1409301	1414110	D	N	CB4853	1380609	1381364	1409647	1410803	28284
Y82E9BR.14a	Y82E9BR.14	III	1409301	1414110	D	N	JU258	1345721	1346343	1409647	1413962	63305
Y82E9BR.2	Y82E9BR.2	III	1439086	1443180	D	N	MY2	1439086	1439221	1441977	1442300	2757
Y82E9BR.20	Y82E9BR.20	III	1374142	1376532	D	Y	JU258	1345721	1346343	1409647	1413962	63305
Y82E9BR.20	Y82E9BR.20	III	1374142	1376532	D	Y	KR314	1362368	1362442	1406114	1410803	43673

Y82E9BR.21	Y82E9BR.21	III	1404782	1406220	D	Y	AB1	1380609	1380678	1409647	1410803	28970
Y82E9BR.21	Y82E9BR.21	III	1404782	1406220	D	Y	CB4853	1380609	1381364	1409647	1410803	28284
Y82E9BR.21	Y82E9BR.21	III	1404782	1406220	D	N	CB4858	1380609	1381364	1405478	1410803	24115
Y82E9BR.21	Y82E9BR.21	III	1404782	1406220	D	Y	JU258	1345721	1346343	1409647	1413962	63305
Y82E9BR.21	Y82E9BR.21	III	1404782	1406220	D	N	KR314	1362368	1362442	1406114	1410803	43673
Y82E9BR.21	Y82E9BR.21	III	1404782	1406220	D	N	MY2	1380609	1380678	1406114	1409608	25437
Y82E9BR.22	Y82E9BR.22	III	1406544	1406753	D	Y	AB1	1380609	1380678	1409647	1410803	28970
Y82E9BR.22	Y82E9BR.22	III	1406544	1406753	D	Y	CB4853	1380609	1381364	1409647	1410803	28284
Y82E9BR.22	Y82E9BR.22	III	1406544	1406753	D	Y	JU258	1345721	1346343	1409647	1413962	63305
Y82E9BR.23	Y82E9BR.23	III	1401956	1403309	D	Y	AB1	1380609	1380678	1409647	1410803	28970
Y82E9BR.23	Y82E9BR.23	III	1401956	1403309	D	Y	CB4853	1380609	1381364	1409647	1410803	28284
Y82E9BR.23	Y82E9BR.23	III	1401956	1403309	D	Y	CB4858	1380609	1381364	1405478	1410803	24115
Y82E9BR.23	Y82E9BR.23	III	1401956	1403309	D	Y	JU258	1345721	1346343	1409647	1413962	63305
Y82E9BR.23	Y82E9BR.23	III	1401956	1403309	D	Y	KR314	1362368	1362442	1406114	1410803	43673
Y82E9BR.23	Y82E9BR.23	III	1401956	1403309	D	Y	MY2	1380609	1380678	1406114	1409608	25437
Y82E9BR.4	Y82E9BR.4	III	1399366	1401295	D	Y	AB1	1380609	1380678	1409647	1410803	28970
Y82E9BR.4	Y82E9BR.4	III	1399366	1401295	D	Y	CB4853	1380609	1381364	1409647	1410803	28284
Y82E9BR.4	Y82E9BR.4	III	1399366	1401295	D	Y	CB4858	1380609	1381364	1405478	1410803	24115
Y82E9BR.4	Y82E9BR.4	III	1399366	1401295	D	Y	JU258	1345721	1346343	1409647	1413962	63305
Y82E9BR.4	Y82E9BR.4	III	1399366	1401295	D	Y	KR314	1362368	1362442	1406114	1410803	43673
Y82E9BR.4	Y82E9BR.4	III	1399366	1401295	D	Y	MY2	1380609	1380678	1406114	1409608	25437
Y82E9BR.5	Y82E9BR.5	III	1387457	1388127	D	Y	AB1	1380609	1380678	1409647	1410803	28970
Y82E9BR.5	Y82E9BR.5	III	1387457	1388127	D	Y	CB4853	1380609	1381364	1409647	1410803	28284
Y82E9BR.5	Y82E9BR.5	III	1387457	1388127	D	Y	CB4858	1380609	1381364	1405478	1410803	24115
Y82E9BR.5	Y82E9BR.5	III	1387457	1388127	D	Y	JU258	1345721	1346343	1409647	1413962	63305
Y82E9BR.5	Y82E9BR.5	III	1387457	1388127	D	Y	KR314	1362368	1362442	1406114	1410803	43673
Y82E9BR.5	Y82E9BR.5	III	1387457	1388127	D	Y	MY2	1380609	1380678	1406114	1409608	25437
Y82E9BR.6	Y82E9BR.6	III	1381435	1381617	D	Y	AB1	1380609	1380678	1409647	1410803	28970
Y82E9BR.6	Y82E9BR.6	III	1381435	1381617	D	Y	CB4853	1380609	1381364	1409647	1410803	28284
Y82E9BR.6	Y82E9BR.6	III	1381435	1381617	D	Y	CB4858	1380609	1381364	1405478	1410803	24115
Y82E9BR.6	Y82E9BR.6	III	1381435	1381617	D	Y	JU258	1345721	1346343	1409647	1413962	63305
Y82E9BR.6	Y82E9BR.6	III	1381435	1381617	D	Y	KR314	1362368	1362442	1406114	1410803	43673
Y82E9BR.6	Y82E9BR.6	III	1381435	1381617	D	Y	MY2	1380609	1380678	1406114	1409608	25437
Y82E9BR.7	Y82E9BR.7	III	1360497	1362666	D	Y	JU258	1345721	1346343	1409647	1413962	63305
Y82E9BR.7	Y82E9BR.7	III	1360497	1362666	D	N	KR314	1359570	1360039	1362124	1362185	2086
Y82E9BR.7	Y82E9BR.7	III	1360497	1362666	D	N	KR314	1362368	1362442	1406114	1410803	43673
Y82E9BR.8	Y82E9BR.8	III	1357070	1359125	D	Y	JU258	1345721	1346343	1409647	1413962	63305
Y82E9BR.8	Y82E9BR.8	III	1357070	1359125	D	N	JU263	1353444	1355120	1357839	1358000	2720
Y82E9BR.8	Y82E9BR.8	III	1357070	1359125	D	N	JU322	1353444	1355120	1358000	1359570	2881
Y82E9BR.8	Y82E9BR.8	III	1357070	1359125	D	N	KR314	1345721	1346940	1357785	1357839	10846
Y82E9BR.9	Y82E9BR.9	III	1359244	1359759	D	Y	JU258	1345721	1346343	1409647	1413962	63305
Y92H12BR.4	Y92H12BR.4	I	1433016	1433852	D	N	KR314	1424554	1432858	1433769	1436306	912
Y94A7B.1	srh-292	V	17802736	17803890	D	Y	JU258	17801700	17801810	17820902	17822222	19093
Y94A7B.1	srh-292	V	17802736	17803890	D	N	MY2	17801700	17802876	17808438	17810485	5563
Y94A7B.11	Y94A7B.11	V	17802377	17802586	D	Y	JU258	17801700	17801810	17820902	17822222	19093
Y94A7B.3	srh-291	V	17805453	17808578	D	Y	JU258	17801700	17801810	17820902	17822222	19093
Y94A7B.3	srh-291	V	17805453	17808578	D	N	MY2	17801700	17802876	17808438	17810485	5563
Y94A7B.4	srh-296	V	17810320	17812761	D	N	CB4856	17811878	17812669	17833335	17836695	20667
Y94A7B.4	srh-296	V	17810320	17812761	D	Y	JU258	17801700	17801810	17820902	17822222	19093
Y94A7B.4	srh-296	V	17810320	17812761	D	N	MY2	17811918	17812669	17833335	17836695	20667
Y94A7B.5	srh-298	V	17818834	17820970	D	Y	CB4856	17811878	17812669	17833335	17836695	20667
Y94A7B.5	srh-298	V	17818834	17820970	D	N	JU258	17801700	17801810	17820902	17822222	19093
Y94A7B.5	srh-298	V	17818834	17820970	D	Y	MY2	17811918	17812669	17833335	17836695	20667
Y94A7B.6	srh-300	V	17822072	17823856	D	Y	CB4856	17811878	17812669	17833335	17836695	20667
Y94A7B.6	srh-300	V	17822072	17823856	D	Y	MY2	17811918	17812669	17833335	17836695	20667
Y94A7B.7	srh-303	V	17831617	17833385	D	N	CB4856	17811878	17812669	17833335	17836695	20667
Y94A7B.7	srh-303	V	17831617	17833385	D	N	MY2	17811918	17812669	17833335	17836695	20667
Y94A7B.8	srh-301	V	17824877	17827741	D	Y	CB4856	17811878	17812669	17833335	17836695	20667
Y94A7B.8	srh-301	V	17824877	17827741	D	Y	MY2	17811918	17812669	17833335	17836695	20667
Y94A7B.9	srh-304	V	17828577	17829950	D	Y	CB4856	17811878	17812669	17833335	17836695	20667

Y94A7B.9	srh-304	V	17828577	17829950	D	Y	MY2	17811918	17812669	17833335	17836695	20667
Y94H6A.10	Y94H6A.10	IV	2709982	2710667	D	N	AB1	2707074	2710143	2710599	2712030	457
Y94H6A.10	Y94H6A.10	IV	2709982	2710667	D	N	CB4854	2707315	2710143	2710599	2712030	457
Y94H6A.10	Y94H6A.10	IV	2709982	2710667	D	N	CB4856	2707074	2710143	2710599	2712030	457
Y94H6A.10	Y94H6A.10	IV	2709982	2710667	D	N	JU258	2707074	2710143	2710599	2712030	457
Y94H6A.10	Y94H6A.10	IV	2709982	2710667	D	N	JU322	2707074	2710143	2710599	2712030	457
Y94H6A.10	Y94H6A.10	IV	2709982	2710667	D	N	MY2	2707315	2710143	2710599	2712030	457
Y9C9A.15	srz-28	IV	4971661	4973193	D	N	JU263	4971745	4971811	4972592	4972653	782
ZC15.2	clec-262	V	20307207	20311016	D	N	MY2	20309585	20310567	20311098	20313164	532
ZC204.10	fbxb-16	II	1650822	1651583	D	Y	JU258	1645645	1645716	1660932	1663710	15217
ZC204.11	btb-13	II	1653778	1654735	D	Y	JU258	1645645	1645716	1660932	1663710	15217
ZC204.12	ZC204.12	II	1656266	1657305	D	N	CB4853	1656998	1657177	1660932	1663710	3756
ZC204.12	ZC204.12	II	1656266	1657305	D	N	CB4858	1656998	1657177	1660932	1663710	3756
ZC204.12	ZC204.12	II	1656266	1657305	D	Y	JU258	1645645	1645716	1660932	1663710	15217
ZC204.12	ZC204.12	II	1656266	1657305	D	N	JU322	1656906	1657177	1660932	1663710	3756
ZC204.12	ZC204.12	II	1656266	1657305	D	N	KR314	1656813	1657177	1660932	1663710	3756
ZC204.13	ZC204.13	II	1660477	1660991	D	N	CB4853	1656998	1657177	1660932	1663710	3756
ZC204.13	ZC204.13	II	1660477	1660991	D	N	CB4858	1656998	1657177	1660932	1663710	3756
ZC204.13	ZC204.13	II	1660477	1660991	D	N	JU258	1645645	1645716	1660932	1663710	15217
ZC204.13	ZC204.13	II	1660477	1660991	D	N	JU322	1656906	1657177	1660932	1663710	3756
ZC204.13	ZC204.13	II	1660477	1660991	D	N	KR314	1656813	1657177	1660932	1663710	3756
ZC204.3	btb-12	II	1655220	1656151	D	Y	JU258	1645645	1645716	1660932	1663710	15217
ZC204.7	fbxb-15	II	1652518	1653500	D	Y	JU258	1645645	1645716	1660932	1663710	15217
ZC204.8	fbxb-21	II	1647911	1648868	D	Y	CB4856	1646825	1647203	1649806	1650120	2604
ZC204.8	fbxb-21	II	1647911	1648868	D	Y	JU258	1645645	1645716	1660932	1663710	15217
ZC204.9	fbxb-20	II	1649203	1650172	D	N	CB4856	1646825	1647203	1649806	1650120	2604
ZC204.9	fbxb-20	II	1649203	1650172	D	Y	JU258	1645645	1645716	1660932	1663710	15217
ZC239.10	sri-53	II	3201199	3202614	D	Y	JU258	3195432	3196064	3249275	3250319	53212
ZC239.12	sdz-35	II	3207863	3208746	D	Y	CB4856	3205302	3205711	3210365	3212314	4655
ZC239.12	sdz-35	II	3207863	3208746	D	Y	JU258	3195432	3196064	3249275	3250319	53212
ZC239.12	sdz-35	II	3207863	3208746	D	Y	KR314	3205302	3205711	3210365	3212314	4655
ZC239.13	ZC239.13	II	3222401	3222822	D	Y	CB4856	3221413	3221670	3223403	3223933	1734
ZC239.13	ZC239.13	II	3222401	3222822	D	Y	JU258	3195432	3196064	3249275	3250319	53212
ZC239.13	ZC239.13	II	3222401	3222822	D	Y	KR314	3221413	3221454	3223403	3223933	1950
ZC239.14	ZC239.14	II	3221389	3222005	D	N	CB4856	3221413	3221670	3223403	3223933	1734
ZC239.14	ZC239.14	II	3221389	3222005	D	Y	JU258	3195432	3196064	3249275	3250319	53212
ZC239.14	ZC239.14	II	3221389	3222005	D	N	KR314	3221413	3221454	3223403	3223933	1950
ZC239.15	ZC239.15	II	3223540	3224638	D	Y	JU258	3195432	3196064	3249275	3250319	53212
ZC239.16	ZC239.16	II	3224765	3225280	D	N	CB4856	3224933	3224997	3237080	3240493	12084
ZC239.16	ZC239.16	II	3224765	3225280	D	Y	JU258	3195432	3196064	3249275	3250319	53212
ZC239.16	ZC239.16	II	3224765	3225280	D	N	KR314	3224933	3224997	3237080	3241072	12084
ZC239.17	ZC239.17	II	3225457	3225963	D	Y	CB4856	3224933	3224997	3237080	3240493	12084
ZC239.17	ZC239.17	II	3225457	3225963	D	Y	JU258	3195432	3196064	3249275	3250319	53212
ZC239.17	ZC239.17	II	3225457	3225963	D	Y	KR314	3224933	3224997	3237080	3241072	12084
ZC239.17	ZC239.17	II	3225457	3225963	D	Y	MY2	3224933	3225304	3237080	3240493	11777
ZC239.19	sri-50	II	3203710	3205538	D	Y	JU258	3195432	3196064	3249275	3250319	53212
ZC239.2	ZC239.2	II	3217902	3218985	D	Y	CB4856	3214750	3215044	3219521	3221413	4478
ZC239.2	ZC239.2	II	3217902	3218985	D	Y	JU258	3195432	3196064	3249275	3250319	53212
ZC239.2	ZC239.2	II	3217902	3218985	D	N	KR314	3214750	3215044	3218194	3218364	3151
ZC239.20	ZC239.20	II	3206015	3206536	D	Y	CB4856	3205302	3205711	3210365	3212314	4655
ZC239.20	ZC239.20	II	3206015	3206536	D	Y	JU258	3195432	3196064	3249275	3250319	53212
ZC239.20	ZC239.20	II	3206015	3206536	D	Y	KR314	3205302	3205711	3210365	3212314	4655
ZC239.21	ZC239.21	II	3206934	3207383	D	Y	CB4856	3205302	3205711	3210365	3212314	4655
ZC239.21	ZC239.21	II	3206934	3207383	D	Y	JU258	3195432	3196064	3249275	3250319	53212
ZC239.21	ZC239.21	II	3206934	3207383	D	Y	KR314	3205302	3205711	3210365	3212314	4655
ZC239.3	ZC239.3	II	3216076	3216595	D	Y	CB4856	3214750	3215044	3219521	3221413	4478
ZC239.3	ZC239.3	II	3216076	3216595	D	Y	JU258	3195432	3196064	3249275	3250319	53212
ZC239.3	ZC239.3	II	3216076	3216595	D	Y	KR314	3214750	3215044	3218194	3218364	3151
ZC239.4	ZC239.4	II	3214447	3215415	D	N	CB4856	3214750	3215044	3219521	3221413	4478
ZC239.4	ZC239.4	II	3214447	3215415	D	Y	JU258	3195432	3196064	3249275	3250319	53212

ZC239.4	ZC239.4	II	3214447	3215415	D	N	KR314	3214750	3215044	3218194	3218364	3151
ZC239.5	ZC239.5	II	3212366	3213147	D	N	CB4856	3212611	3212650	3212936	3213000	287
ZC239.5	ZC239.5	II	3212366	3213147	D	Y	JU258	3195432	3196064	3249275	3250319	53212
ZC239.5	ZC239.5	II	3212366	3213147	D	N	KR314	3212611	3212650	3212818	3213000	169
ZC239.6	ZC239.6	II	3209473	3211371	D	N	CB4856	3205302	3205711	3210365	3212314	4655
ZC239.6	ZC239.6	II	3209473	3211371	D	Y	JU258	3195432	3196064	3249275	3250319	53212
ZC239.6	ZC239.6	II	3209473	3211371	D	N	KR314	3205302	3205711	3210365	3212314	4655
ZC239.7	gcy-15	II	3192415	3196870	D	N	JU258	3195432	3196064	3249275	3250319	53212
ZC239.7	gcy-15	II	3192415	3196870	D	N	MY2	3180783	3180993	3196064	3196905	15072
ZC239.8	sri-51	II	3197608	3199093	D	Y	JU258	3195432	3196064	3249275	3250319	53212
ZC239.9	sri-48	II	3199613	3200901	D	Y	JU258	3195432	3196064	3249275	3250319	53212
ZC449.6	hsd-3	X	5009473	5011191	D	N	CB4853	5007002	5009473	5010139	5010720	667
ZC449.6	hsd-3	X	5009473	5011191	D	N	CB4853	5007002	5009473	5010139	5010720	667
ZC482.2	ZC482.2	III	12747779	12750807	A	N	CB4856	12748262	12748866	12753529	12755256	4664
ZC482.7	ZC482.7	III	12750989	12753514	A	Y	CB4856	12748262	12748866	12753529	12755256	4664
ZC53.4	ZC53.4	X	1925745	1937684	D	N	CB4856	1930251	1931466	1940418	1957551	8953
ZC53.7	rgs-9	X	1915260	1917072	D	N	JU258	1915609	1915784	1915872	1915994	89
ZK1037.1	ZK1037.1	V	15311056	15312512	A	Y	RW7000	15282709	15282820	15399718	15400303	116899
ZK1037.10	wrt-7	V	15334758	15337481	A	Y	RW7000	15282709	15282820	15399718	15400303	116899
ZK1037.11	srz-10	V	15307554	15309039	A	Y	RW7000	15282709	15282820	15399718	15400303	116899
ZK1037.13	ZK1037.13	V	15318937	15320529	D	N	CB4856	15319833	15319874	15320155	15323829	282
ZK1037.13	ZK1037.13	V	15318937	15320529	D	N	JU258	15319662	15319833	15320155	15323829	323
ZK1037.13	ZK1037.13	V	15318937	15320529	A	Y	RW7000	15282709	15282820	15399718	15400303	116899
ZK1037.3	srt-22	V	15314644	15315903	A	Y	RW7000	15282709	15282820	15399718	15400303	116899
ZK1037.4	nhr-246	V	15316473	15318401	A	Y	RW7000	15282709	15282820	15399718	15400303	116899
ZK1037.5	nhr-247	V	15323829	15325327	A	Y	RW7000	15282709	15282820	15399718	15400303	116899
ZK1037.6	ZK1037.6	V	15325808	15327771	A	Y	RW7000	15282709	15282820	15399718	15400303	116899
ZK1037.8	srh-295	V	15330590	15332750	A	Y	RW7000	15282709	15282820	15399718	15400303	116899
ZK1037.9	srw-113	V	15333127	15334547	A	Y	RW7000	15282709	15282820	15399718	15400303	116899
ZK121.1a	glrx-21	III	5345240	5346075	A	N	CB4856	5343622	5343681	5346012	5346266	2332
ZK121.1a	glrx-21	III	5345240	5346075	A	N	KR314	5343830	5343902	5346012	5346266	2111
ZK121.1a	glrx-21	III	5345240	5346075	A	N	MY2	5343830	5344083	5346012	5346266	1930
ZK121.2	ZK121.2	III	5341648	5344204	A	N	CB4853	5343757	5343902	5344232	5345363	331
ZK121.2	ZK121.2	III	5341648	5344204	A	N	CB4854	5343681	5343757	5344232	5345363	476
ZK121.2	ZK121.2	III	5341648	5344204	A	N	CB4856	5343622	5343681	5346012	5346266	2332
ZK121.2	ZK121.2	III	5341648	5344204	A	N	CB4858	5343681	5343902	5344232	5345363	331
ZK121.2	ZK121.2	III	5341648	5344204	A	N	JU258	5343830	5343956	5344232	5345363	277
ZK121.2	ZK121.2	III	5341648	5344204	A	N	KR314	5343830	5343902	5346012	5346266	2111
ZK121.2	ZK121.2	III	5341648	5344204	A	N	MY2	5343830	5344083	5346012	5346266	1930
ZK1225.5	ZK1225.5	I	13216593	13218855	A	N	CB4854	13217734	13217859	13218754	13219072	896
ZK1240.1	ZK1240.1	II	2329356	2331793	D	Y	JU258	2264403	2265489	2338778	2338818	73290
ZK1240.2	ZK1240.2	II	2323397	2325166	D	Y	CB4856	2319800	2320359	2327320	2327435	6962
ZK1240.2	ZK1240.2	II	2323397	2325166	D	Y	JU258	2264403	2265489	2338778	2338818	73290
ZK1240.2	ZK1240.2	II	2323397	2325166	D	Y	JU263	2319800	2320359	2327571	2328734	7213
ZK1240.2	ZK1240.2	II	2323397	2325166	D	N	JU322	2318762	2320359	2323431	2323650	3073
ZK1240.3	ZK1240.3	II	2320331	2321952	D	N	CB4856	2319800	2320359	2327320	2327435	6962
ZK1240.3	ZK1240.3	II	2320331	2321952	D	Y	JU258	2264403	2265489	2338778	2338818	73290
ZK1240.3	ZK1240.3	II	2320331	2321952	D	N	JU263	2319800	2320359	2327571	2328734	7213
ZK1240.3	ZK1240.3	II	2320331	2321952	D	N	JU322	2318762	2320359	2323431	2323650	3073
ZK1240.4	ZK1240.4	II	2315085	2315623	D	Y	CB4856	2302120	2306782	2318165	2318664	11384
ZK1240.4	ZK1240.4	II	2315085	2315623	D	Y	JU258	2264403	2265489	2338778	2338818	73290
ZK1240.4	ZK1240.4	II	2315085	2315623	D	Y	JU263	2302181	2306782	2318165	2318664	11384
ZK1240.4	ZK1240.4	II	2315085	2315623	D	Y	JU322	2299972	2300284	2318165	2318664	17882
ZK1240.4	ZK1240.4	II	2315085	2315623	D	Y	KR314	2302181	2306782	2318165	2318664	11384
ZK1240.5	ZK1240.5	II	2316365	2317878	D	Y	CB4856	2302120	2306782	2318165	2318664	11384
ZK1240.5	ZK1240.5	II	2316365	2317878	D	Y	JU258	2264403	2265489	2338778	2338818	73290
ZK1240.5	ZK1240.5	II	2316365	2317878	D	Y	JU263	2302181	2306782	2318165	2318664	11384
ZK1240.5	ZK1240.5	II	2316365	2317878	D	Y	JU322	2299972	2300284	2318165	2318664	17882
ZK1240.5	ZK1240.5	II	2316365	2317878	D	Y	KR314	2302181	2306782	2318165	2318664	11384
ZK1240.6	ZK1240.6	II	2322415	2322908	D	Y	CB4856	2319800	2320359	2327320	2327435	6962

ZK1240.6	ZK1240.6	II	2322415	2322908	D	Y	JU258	2264403	2265489	2338778	2338818	73290
ZK1240.6	ZK1240.6	II	2322415	2322908	D	Y	JU263	2319800	2320359	2327571	2328734	7213
ZK1240.6	ZK1240.6	II	2322415	2322908	D	Y	JU322	2318762	2320359	2323431	2323650	3073
ZK1240.8	ZK1240.8	II	2326996	2329053	D	N	CB4856	2319800	2320359	2327320	2327435	6962
ZK1240.8	ZK1240.8	II	2326996	2329053	D	Y	JU258	2264403	2265489	2338778	2338818	73290
ZK1240.8	ZK1240.8	II	2326996	2329053	D	N	JU263	2319800	2320359	2327571	2328734	7213
ZK1240.9	ZK1240.9	II	2318222	2319745	D	Y	JU258	2264403	2265489	2338778	2338818	73290
ZK337.2	ZK337.2	I	14975406	14980586	A	N	JU258	14978290	14978806	14985108	14994760	6303
ZK337.4	tag-4	I	14984482	14985087	A	Y	JU258	14978290	14978806	14985108	14994760	6303
ZK355.5	ZK355.5	II	2934801	2937948	D	N	AB1	2933462	2934689	2935105	2935173	417
ZK355.5	ZK355.5	II	2934801	2937948	D	N	JU258	2933462	2934689	2935105	2935173	417
ZK355.5	ZK355.5	II	2934801	2937948	D	N	JU263	2933462	2934689	2935105	2935173	417
ZK355.5	ZK355.5	II	2934801	2937948	D	N	KR314	2933462	2934689	2935105	2935173	417
ZK39.3	clec-94	I	11146440	11148301	D	N	JU258	11147731	11147948	11148200	11148925	253
ZK39.3	clec-94	I	11146440	11148301	D	N	JU263	11147731	11147948	11148200	11148925	253
ZK39.3	clec-94	I	11146440	11148301	D	N	KR314	11147731	11147948	11148200	11148925	253
ZK666.11	ZK666.11	II	10483835	10484428	A	Y	CB4858	10477044	10477549	10490009	10490052	12461
ZK666.11	ZK666.11	II	10483835	10484428	A	Y	JU322	10477044	10477549	10490009	10490052	12461
ZK666.4	ZK666.4	II	10477180	10477614	A	N	CB4858	10477044	10477549	10490009	10490052	12461
ZK666.4	ZK666.4	II	10477180	10477614	A	N	JU322	10477044	10477549	10490009	10490052	12461
ZK666.5	clec-59	II	10477943	10479369	A	Y	CB4858	10477044	10477549	10490009	10490052	12461
ZK666.5	clec-59	II	10477943	10479369	A	Y	JU322	10477044	10477549	10490009	10490052	12461
ZK666.6	clec-60	II	10480073	10481738	A	Y	CB4858	10477044	10477549	10490009	10490052	12461
ZK666.6	clec-60	II	10480073	10481738	A	Y	JU322	10477044	10477549	10490009	10490052	12461
ZK666.7	clec-61	II	10488668	10490435	A	N	CB4858	10477044	10477549	10490009	10490052	12461
ZK666.7	clec-61	II	10488668	10490435	A	N	JU322	10477044	10477549	10490009	10490052	12461
ZK673.9	clec-143	II	10468272	10470176	D	N	CB4853	10468315	10468472	10468797	10468850	326
ZK673.9	clec-143	II	10468272	10470176	D	N	CB4854	10468315	10468472	10468797	10468850	326
ZK673.9	clec-143	II	10468272	10470176	D	N	JU322	10468315	10468472	10468797	10468850	326
ZK697.5	srw-103	V	1717244	1719465	D	N	JU258	1715350	1717530	1717618	1717682	89
ZK697.7	srab-25	V	1738928	1741158	A	Y	CB4856	1736658	1738928	1741109	1743667	2182
ZK697.7	srab-25	V	1738928	1741158	A	Y	CB4856	1736658	1738928	1741109	1743667	2182
ZK892.3	ZK892.3	II	10001768	10003893	D	N	MY2	10002455	10002726	10002837	10002891	112
ZK896.4	ZK896.4	IV	12880636	12883115	D	N	RW7000	12881617	12881730	12968210	12968283	86481
ZK970.6	gcy-5	II	10310321	10316140	D	N	KR314	10311824	10312319	10312904	10312943	586
ZK970.7	ZK970.7	II	10312244	10312832	D	N	KR314	10311824	10312319	10312904	10312943	586
ZK993.2	ZK993.2	I	1107584	1115503	D	N	CB4856	1109988	1110028	1115236	1128003	5209
